# Genome-wide characterization of LTR retrotransposons in the non-model deep-sea annelid *Lamellibrachia luymesi*

**DOI:** 10.1186/s12864-021-07749-1

**Published:** 2021-06-23

**Authors:** Oluchi Aroh, Kenneth M. Halanych

**Affiliations:** Department of Biological Sciences & Molette Biology Laboratory for Environmental and Climate Change Studies, College of Science and Mathematics, Auburn University, 101 Rouse Life Science Building, Auburn, AL 36849 USA

**Keywords:** Long terminal repeat retrotransposon, *Lamellibrachia luymesi*, Lophotrochozoan, Annelid

## Abstract

**Background:**

Long Terminal Repeat retrotransposons (LTR retrotransposons) are mobile genetic elements composed of a few genes between terminal repeats and, in some cases, can comprise over half of a genome’s content. Available data on LTR retrotransposons have facilitated comparative studies and provided insight on genome evolution. However, data are biased to model systems and marine organisms, including annelids, have been underrepresented in transposable elements studies. Here, we focus on genome of *Lamellibrachia luymesi*, a vestimentiferan tubeworm from deep-sea hydrocarbon seeps, to gain knowledge of LTR retrotransposons in a deep-sea annelid.

**Results:**

We characterized LTR retrotransposons present in the genome of *L. luymesi* using bioinformatic approaches and found that intact LTR retrotransposons makes up about 0.1% of *L. luymesi* genome. Previous characterization of the genome has shown that this tubeworm hosts several known LTR-retrotransposons. Here we describe and classify LTR retrotransposons in *L. luymesi* as within the Gypsy, Copia and Bel-pao superfamilies. Although, many elements fell within already recognized families (e.g., Mag, CSRN1), others formed clades distinct from previously recognized families within these superfamilies. However, approximately 19% (41) of recovered elements could not be classified. Gypsy elements were the most abundant while only 2 Copia and 2 Bel-pao elements were present. In addition, analysis of insertion times indicated that several LTR-retrotransposons were recently transposed into the genome of *L. luymesi*, these elements had identical LTR’s raising possibility of recent or ongoing retrotransposon activity.

**Conclusions:**

Our analysis contributes to knowledge on diversity of LTR-retrotransposons in marine settings and also serves as an important step to assist our understanding of the potential role of retroelements in marine organisms. We find that many LTR retrotransposons, which have been inserted in the last few million years, are similar to those found in terrestrial model species. However, several new groups of LTR retrotransposons were discovered suggesting that the representation of LTR retrotransposons may be different in marine settings. Further study would improve understanding of the diversity of retrotransposons across animal groups and environments.

**Supplementary Information:**

The online version contains supplementary material available at 10.1186/s12864-021-07749-1.

## Introduction

Retrotransposons are transposable elements that replicate via an RNA intermediate [1]. They often make up a substantial fraction of the host genome in which they reside, occupying more than 40% of the human genome [2] and more than 50% of the maize genome [3]. Retrotransposons play a role in genome evolution [4] and can ultimately impact gene expression. However, our understanding of phylogenetic diversity of retrotransposons and their role in genome evolution is largely based on model organisms such as *Drosophila melanogaster*, *Caenorhabditis elegans*, *Danio rerio*, *Mus musculus*, *Bombyx mori*, etc. Animals living in marine environments and the deep-sea have been particularly underrepresented in transposable elements studies. For this reason, we explored the genome of the deep-sea tubeworm *Lamellibrachia luymesi* (Siboglinidae, Annelida) [5] which employs chemoautotrophic endosymbionts to inhabit hydrocarbon seeps in the Gulf of Mexico.

Retrotransposons are usually classified into two categories: LTR retrotransposons and non-LTR retrotransposons. Long terminal repeat retrotransposons (LTR retrotransposons) are transposable elements that are characterized by having long terminal repeats (LTRs) flanking an internal coding region. LTR retrotransposons usually serve as a model for the study of retroviruses [6], because both are structurally similar and phylogenetically related [7]. The main distinguishing characteristic is the presence of an *envelope (env)* gene in retroviruses which is absent in LTR retrotransposons. LTR retrotransposons are classified into three super families (Copia, Gypsy and Bel-pao), which differ in the arrangement of the protein domains encoded within the *pol* gene [8]. The two most common LTR retrotransposon super-families – Copia and Gypsy, are found in almost all eukaryotic lineages sampled to date [9]. These superfamilies display different distribution, abundance and diversity based on the element type and the host taxon been considered [10].

**Fig. 1:**
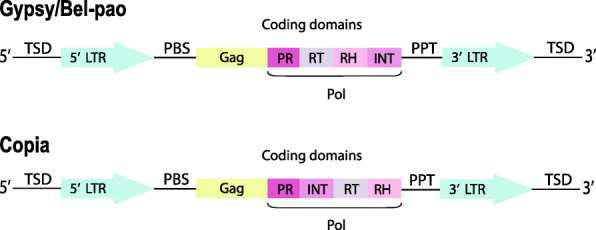
Structure of a LTR retrotransposon. Gag - group-specific antigen gene; TSD- target site duplication; PR - aspartic protease gene; RT - reverse transcriptase gene; RH - ribonuclease-H gene; INT- integrase gene; PBS - primer binding site; PPT - polypurine tract. LTR retrotransposon structure was generated using Adobe Illustrator.

LTR retrotransposons (Fig. 1) includes long terminal repeats flanking elements that range from a few hundred bases to more than 5kb and usually start with 5’TG-3’ and ends with 5’-CA3’, a target site duplication (TSD) of 4-6bp, a polypurine tract (PPT), a primer binding site (PBS) and also *gag* and *pol* genes between the two LTRs [11, 12]. The *gag* gene encodes a structural protein that is essential for assembly of viral-like particles while the *pol* gene encodes four proteins domains including a protease (PR) which cleaves the Pol polyprotein, a ribonuclease H (RH) which cleaves the RNA in the DNA-RNA hybrid, a reverse transcriptase (RT) that copies retrotransposons RNA into cDNA and an integrase (INT) which integrates the cDNA into the genome. Occasionally, an additional open reading frame (aORF) may be downstream or upstream of the *gag-pol gene,* in sense or antisense orientation [13, 14]. Those located in the sense orientation encode proteins with certain structural and functional similarities to the *env* domain of retroviruses, and hence are sometimes called env-like domains [15, 16]. The *env* domain encodes for protein that is responsible for binding the cellular receptor and facilitates the early steps in the virus-cell interaction, and drives the fusion of viral and host cellular membrane [17]. In contrast, function of the aORF located in the antisense orientation is not clearly known, however , studies carried out so far suggests that they may be playing a regulatory role in retrotransposition [16, 18, 19].

In previous reports, retroelements have been identified in marine organisms including sea urchins [20], corals endosymbionts [21] and crustaceans [22]. However, to the best of our knowledge, there has been minimal effort to characterize the LTR retrotransposons present in deep-sea (>200m) animals or in annelids. Available studies [5, 23, 24] tend to only consider transposable elements in context of their role in genome composition rather than detailed assessment of the elements and their evolution. Of particular interest, Li et al. assessed *Lamellibrachia luymesi* van der Land & Norrevang 1975; a deep-sea annelid. *L. luymesi* is a vestimentiferan tubeworm that forms bush-like aggregations at hydrocarbon seeps in the Gulf of Mexico. These animals lack a digestive tract and hosts sulfide-oxidizing, horizontally-transmitted bacterial symbionts for nutrition and growth [5, 25–27]. Their result showed that 2.52% of the genome consisted of LTR retroelements. However, the goal of the analysis was to see how much of the genome’s DNA was derived from repetitive elements using RepeatModeler [28] and RepeatMasker [29]. Their approach included altered copies such as truncated elements or solo LTR’s to gain a comprehensive view of *L. luymesi*’s genome composition rather than an exploration of the LTR retroelements biology. In the current study, we further characterized and classified LTR retrotransposons present in the genome of *Lamellibrachia luymesi* to shed light on the representation of LTR retrotransposon superfamilies, as well as augment understanding of the potential function and structure of intact elements. In addition, we also estimated insertion times of these elements to understand if they are due to recent or ancient events.

We hypothesized the possible presence of unknown LTR-retrotransposon families in marine organisms or unsampled animal lineages. This work represents an important step towards the characterization of LTR retrotransposons in marine systems (70% of the biosphere) and in unexplored animal lineages (e.g., annelids).

## Results

### Identification and classification of LTR-retrotransposon

**Fig. 2:**
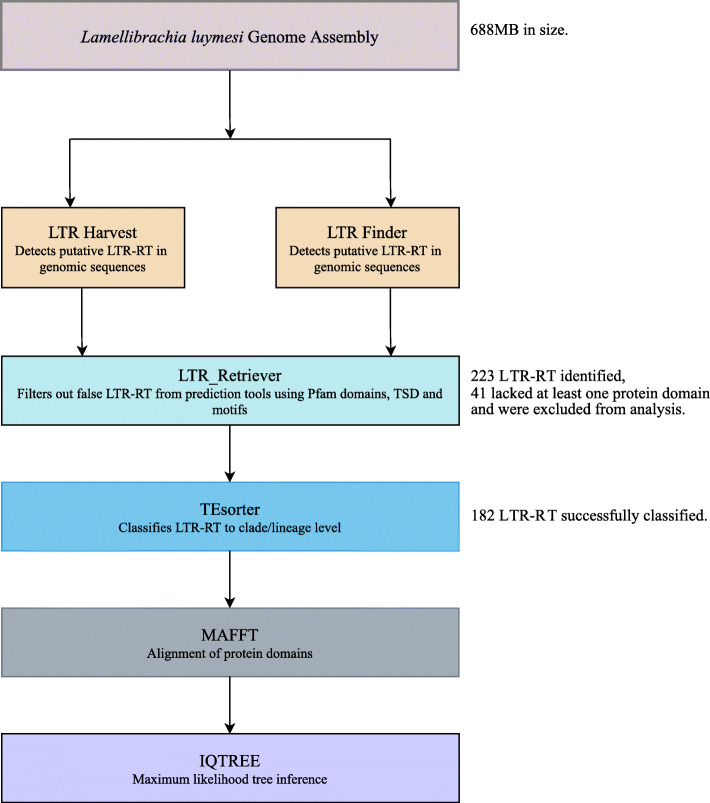
Bioinformatics pipeline for annotation of LTR retrotransposon in *L. luymesi*.

A total of 223 intact LTR retrotransposons (Supplementary Table 1, 2) were identified in the 688 Mb *L. luymesi* genome, by screening and adjustment of LTR candidates from LTRharvest and LTR_Finder using modules employed in LTR_retriever (Fig. 2). Of the 223 intact LTR-retrotransposon identified by LTR_retriever, 51 were classified as unknown, 1 was classified as Copia while 171 were classified as Gypsy.

To further classify these elements, TEsorter was used to search their internal regions against Gypsy database (GYDB). Those matching at least one domain profile in GYDB were classified. All the 171 Gypsy and 1 Copia elements classified by LTR-retriever were also classified as Gypsy and Copia respectively in TEsorter. In addition, out of the 51 classified by LTR_retriever as unknown, 7 were classified as Gypsy, 2 were classified as Bel-pao while 1 was classified as Copia in TEsorter. The rest were not classified at all. Hence, in total, TEsorter classified 182 of the 223 intact LTR retrotransposons identified by LTR-retriever (Supplementary Table 2).

Further analyses were carried out on the remaining 41 elements not classified by TEsorter. This was accomplished by manually searching the internal region of these unclassified elements against PFAM [30] and Conserved Domains Database (CDD) [31] to identify domains present within their internal region. Results showed that 24 of the elements lacked domains matching any known profiles in the databases, 10 had domains that were unrelated to LTR retrotransposons (e.g., a transmembrane receptor, coagulation-inhibition site etc.), while the remaining 8 had only RT domains (Supplementary Table 1). To further verify and classify these elements, we used REXdb-metazoan database option of TEsorter. We also performed a manual hmmscan search using GYDB hmm profiles. The REXdb- metazoan option classified these elements as LINEs (Long interspersed nuclear elements) while no match was found in the GYDB hmm profile scan. Due to the inability to accurately classify these 41 elements, they were excluded from further analysis.

Summary details of the 182 LTR retrotransposons used for downstream analysis, which includes 178 Gypsy, 2 Bel-pao and 2 Copia elements are shown in Table 1.

**Table 1:** Summary of LTR retrotransposons in *L. luymesi*

Superfamily	Structure	Total number	No. with all domains present	Average length of element (min-max)	Total length of elements in bp	Range of percentage LTR identity within Superfamily
Gypsy	Gag-PR-RT-RH-INT	178	30	5123 bp (1389-8866)	836,263	92–100%
Copia	Gag-PR-INT-RT-RH	2	0	3453 bp (2037-4869)	6906	95–99%
Bel-pao	Gag-PR-RT-RH-INT	2	2	6659 bp (6670-6648)	13,318	92–99%
Total		182			856,487	

### Structural characterization

Of the 182 identified LTR retrotransposons, 32 elements had all domains (Gag and Pol – RT, INT, RH, PR) present with the remainder having at least one domain present. For Gypsy elements, 30 out the 178 had a complete set of domains, both the Bel-pao elements had a complete set of domains and both Copia elements lacked a complete set of domains. Further analysis to describe the position of these elements in relation to coding elements showed that 26.4% of them overlapped with coding elements, 46.2% were located > 5 kb of coding elements, 10.4% were located within 5-10 kb and the remaining 17% were more than 10 kb away from coding elements.

The target site duplication flanking ends of identified LTR retrotransposons ranged from 3 to 5 bp in length, with majority of them being 5 bp in length. Palindromic motifs detected in the elements includes TGCA, TACA, TATA, TCGT, TGAA, TGAC, TGAT and TTAT, with 89% of the LTR-retrotransposons having TGCA motif. In addition, differences in length of identified LTR-retrotransposons were substantial, ranging from 1389 bp-8866 bp while the length of the LTRs ranged from 103 to 1468 bp (Supplementary Table 2).

### Estimation of insertion time

**Fig. 3:**
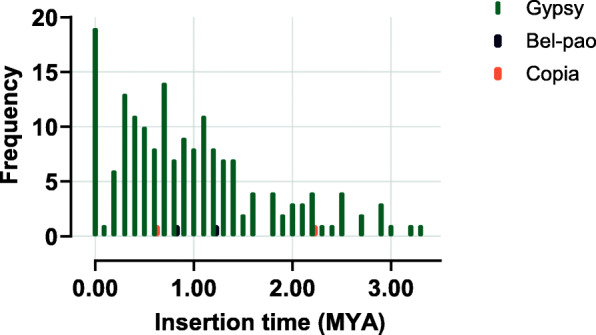
Insertion time distribution of intact LTR-RT in *L. luymesi* genome. Chart was generated using GraphPad Prism.

Insertion times of LTR retrotransposon elements in *L. luymesi* genome suggests that most elements were inserted around 1.0 million years ago (MYA; Fig. 3). The oldest observed and complete inserted retrotransposon was a Gypsy element, inserted around 2MYA. Interestingly, 50 Gypsy elements showed a 100% LTR identity, suggesting that they very recently inserted into the genome. However, calculations of insertion times used a substitution rate of 1.3 × 10^− 8^ substitution per bp per year, the LTR_retriever default based on the rice genome. Although these insertion time estimates for *L. luymesi* should be viewed with caution, decreasing the rate by two- or three-fold still suggests insertion times within the last few million years.

### Phylogenetic analysis of LTR-retrotransposons

**Fig. 4:**
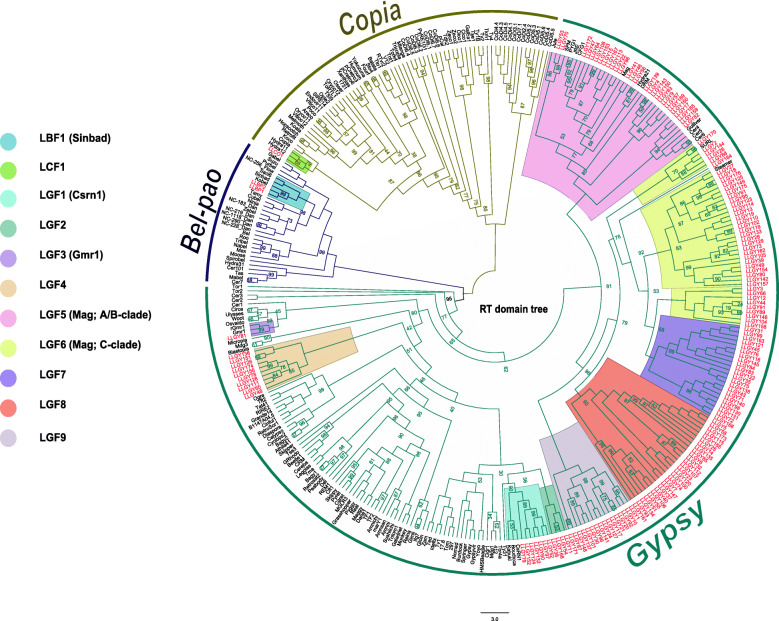
RT domain phylogenetic tree. RT phylogenetic tree was generated in IQtree with the LG + F + R6 model. Tree lines are color-coded according to the superfamily above it. Elements in red are elements identified in the genome of *L. luymesi*.

**Fig. 5:**
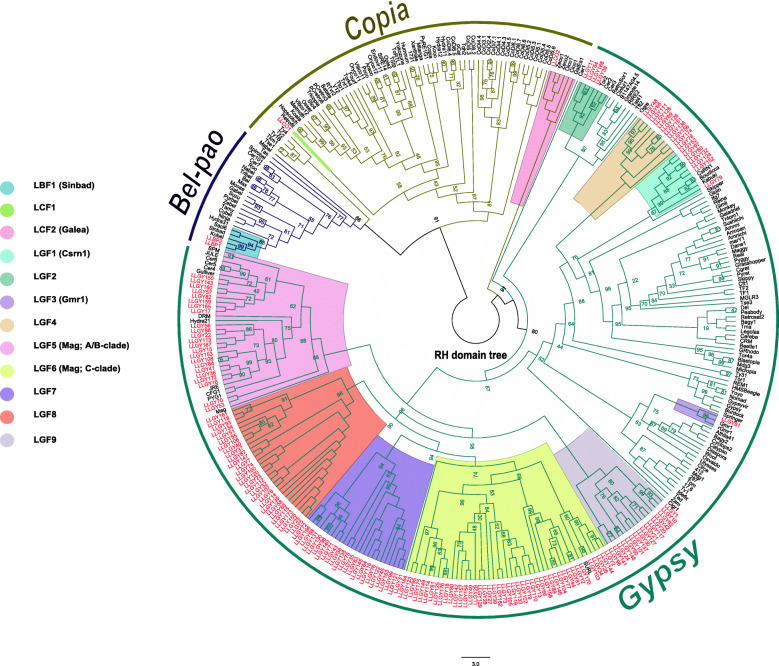
RnaseH domain phylogenetic tree. RnaseH phylogenetic tree was generated in IQtree with the LG + R7 model. Tree lines are color-coded according to the superfamily above it. Elements in red are elements identified in the genome of *L. luymesi*.

**Fig. 6:**
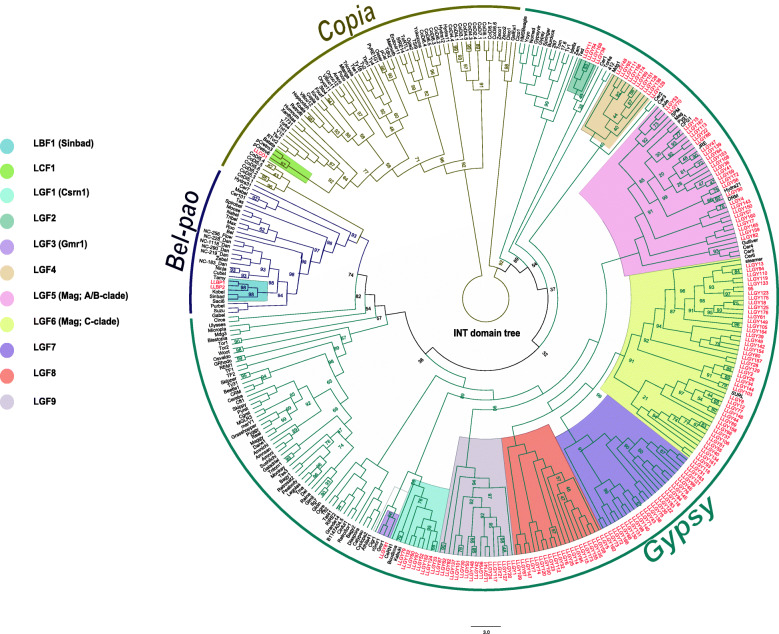
INT domain phylogenetic tree. INT phylogenetic tree was generated in IQtree with the LG + R7 model. Tree lines are color-coded according to the superfamily name above it. Elements in red are elements identified in the genome of *L. luymesi*.

Phylogenetic analysis corroborates assignments made by TEsorter. However, weak internodal support limited inferences about evolutionary relationships. Final family assignment was done by considering placements of elements with strong nodal support indicating monophyletic lineage representing gene families (Fig. 4 for RT domain, Fig. 5 for RH domain, and Fig. 6 for INT domain). Due to issues of non-concordant evolutionary histories, domains were not combined into a single phylogenetic analysis. Naming conventions based on phylogenetic analyses are described in the Methods section.

For Gypsy elements, phylogenetic analysis of the RT, RH and INT sequences showed that some elements fall into recognized families such as CSRN1 [32], Gmr1 [33] and Mag [34, 35] while others formed lineages distinct from previously recognized families. The 5 novel families were LGF2 (bootstrap value, bsv 100 in all the domain trees), LGF4 (bsv = 100, all domains), LGF7 (bsv = 94, 100, 91 in RH, RT and INT domain trees, respectively), LGF8 (bsv = 86, 93, 100 in RH, RT and INT domain trees) and LGF9 (bsv= 100, all domains). Other Gypsy elements fell within the Mag family (LGF5; bsv = 98, 100, 100 in RH, RT and INT domain trees), the Gmr1 family (LGF3; bsv = 95, 99, 100 in RH, RT and INT domain trees) and the CSRN1 family (LGF1; bsv = 99, 100, 100 in RH, RT and INT domain trees respectively). The LGF6 family was also inside the Mag family, but although this clade was monophyletic in the RH and INT trees (bsv= 74, 91 respectively), it was paraphyletic in the RT trees.

Mag elements (LGF5 and LGF6) which includes A, B and C clades where the most dominant with more than 70 elements. Elements in the 2 previously described families; CSRN1 (LGF1) and Gmr1 (LGF3), were fewer with less than 25 elements. The remaining novel families (LGF2 and LGF4) with strong bootstrap support had less than 15 elements. Three of the novel families (LFG8, LFG9 and LFG7) clustered within Mag elements, suggesting that they might be distinct lineage within the Mag radiation.

For the Copia elements, LLCO1 had all 3 domains used in tree building - RT, RH, and INT present while LLCO2 had only the RH domain (but still had GAG and PR domains not used in trees). Hence, LLCO2 was absent in INT and RT trees. In the RH tree, LLCO2 clustered within the GalEa family (LCF2) with a bootstrap value of 100. LLCO1 varied in position in the INT, RT, and RH domain tree (LCF1). In the INT and RT domain tree, this element fell within the pCetro and Hydra family respectively (bsv = 97 and 88, respectively), whereas LLCO1’s position was unsupported in the RH trees (bsv = 58).

Both Bel-pao elements (LLBP1 and LBP2) clustered within Sinbad lineage, LBF1 (bsv = 94, 100, 98 in RH, RT and INT domain trees).

## Discussion

The deep-sea annelid *Lamellibrachia luymesi* genome contained at least 182 intact LTR retrotransposons which clustered into 12 families, 6 of which appear to be novel. All three known superfamilies of LTR retrotransposons – Gypsy, Copia and Bel-pao, were recovered, although several elements could not be classified in the existing families of these superfamilies.

Generally, LTR retrotransposons are known to be more abundant in plant genomes (e.g. > 50% in *Z .mays* genome [3, 36];) than in animal genomes (e.g. only 0.02% of the genome of *C. gigas* [10];). In the genome sequencing study of *L. luymesi* done by Li et al., 2.52% of the genome were reported to be made up of LTR elements. Here, we expand this earlier effort to show that only ~0.1% of the genome is made up of intact LTR elements comprising mainly Gypsy representatives with a few Bel-pao and Copia elements. Importantly, many of these elements appear to represent families/clades new to science in addition to those that could not be classified. Our results, when compared to Li et al., indicates that most of the hits recovered by RepeatModeler and RepeatMasker are truncated, solo LTRs or nested LTR elements. However, a better understanding of LTR retrotransposon domains and a more robust database for LTR retrotransposon in non-model animals would likely allow a more accurate assessment as to the number, representation, and completeness of LTR retrotransposons in *L. luymesi*.

Comparative analysis done in eukaryotes such as crustaceans [22], fungi [9], *D. melanogaster* [37], *B. mori* [38], show that Gypsy elements were the most abundant and with a high copy number. They are also the most diversified with numerous clades and families amongst the 3 superfamilies. Examination of LTR retrotransposons in *L. luymesi* genome corroborates these observations as 97% of the elements classified were Gypsy elements. According to our phylogenetic analysis, 3 previously described families including A-clade and C-clade of the Mag family, Gmr1 and CSRN1 were present in *L. luymesi*. Mag elements have been identified in diverse organisms such as *Caenorhabditis elegans* (roundworm, [39]), *Bombyx mori* (silkworm, [40]) , *Anopheles gambiae* (mosquito, [35]) and *Xiphophorus maculate* (platyfish, [34]). In addition, a recent study shows that more than 290 Mag elements were identified in mollusc genomes [10]. Given their ubiquitous nature, Mag elements been the most common of the Gypsy elements found in *L. luymesi* is not surprising. Most of these Mag elements found are from Mag C-clade which includes SURL elements observed in marine echinoid species [20, 41]. The LGF3 family in *L. luymesi* shared same lineage with the unusual Gmr1 clade. Gmr1 elements differ from other Gypsy LTR-retroelements in that the integrase domain usually lie upstream of the reverse transcriptase domain, an arrangement mostly seen in Copia elements [33]. This clade includes elements that have been discovered in marine organisms such as the Atlantic cod *Gadus morhua* and the tunicate *Ciona intestinalis* [42, 43]. In addition, the LGF1 family clustered within the CSRN1 clade, which was first described in a trematode [32] and is characterized by the elements Kabuki [44], CSRN1 [32], and Boudicca [45]. A recent study reports that CSRN1 clade is also represented in cephalopods [10]. *L. luymesi* also contained 5 novel families of Gypsy elements, making them the most diverse group of LTR retrotransposons in *L. luymesi*.

Copia elements appear to be less abundant in animal genomes than in plant genomes [22, 36]. Here, only 2 intact Copia elements were identified in *L. luymesi*, consistent with these reports. Our phylogenetic analysis showed that these elements formed 2 distinct families, one previously described and one novel. The previously described family, GalEa, has been known to be one of the most predominant Copia retrotransposon as they are widely distributed among metazoans [10, 46]. This element was the first Copia element found in crustaceans, specifically in a deep-sea squat lobsters [46]. In a recent study [22], 29 out of 35 identified Copia elements from the deep-sea hydrothermal shrimp *Rimicaris exoculata* and other crustaceans belonged to the GalEa clade. Though, we only identified 2 Copia element in *L. luymesi*, one of them clustered within a clade found in marine metazoans, suggesting that this element may be common in marine environments. The other novel Copia element found herein did not cluster within any previously known families based on the RH domain tree (Fig. 5).

Recent studies of Bel-pao retrotransposons in metazoan genomes [47] , including mollusc genomes [10] revealed that they are more abundant than Copia elements but lesser than Gypsy elements. In our case, an equivalent number of Copia and Bel-pao elements were found in *L. luymesi* genome. To date, seven Bel/pao families have been well described, namely, Bel, Pao , Sinbad, Suzu, Tas, Flow and Dan [47]. A recent study further subdivides the Sinbad families into Sparrow and Surcourf [10]. In our study, the two Bel-pao elements clustered within the Sinbad family. Sinbad-like elements have been found in marine organism such as purple sea urchins, tunicates, pufferfish and the Atlantic salmon [48], making it a well described element in marine organisms.

The distribution of inferred insertion times of LTR retrotransposons found in *L. luymesi* suggests that current retrotransposons are recent features in the genome of this organism (Fig 3). Further analysis on the most recently transposed elements (less than 1 million years ago) showed that most of these elements had incomplete domains and are scattered across identified families. However, they all had identical LTR’s indicating that they are yet to accumulate mutations. This finding augments the fact that these elements are indeed recent in the genome of *L. luymesi*. A previous study of insertion time estimates has shown that some superfamilies of retrotransposon shows activity at different times in waves while others show activity to be linearly related to time [49], another study suggests difference in spatiality and directionality of insertions among species [50]. However, the insertion time estimates of LTR retrotransposons in *L. luymesi* indicates that Gypsy elements showed a steady activity over a long period of time (more than 3MYA). Unfortunately, we could not make the same inferences for Bel-pao and Copia elements given their limited number.

Understanding the timing of transposon activity is important because transposable elements have been known to impact gene expression, by either generating new gene copies or regulating gene activity [51]. As such, the timing of these events may offer clues as to when such animals experienced bursts of evolution. However, to infer the possible role of transposable elements more fully in the animal genomes, other types of retrotransposons such as non-LTR retrotransposons or other transposable elements needs to be identified and annotated in these organisms.

Lastly, *L. luymesi* belong to a group of animals known as Lophotrochozoans [52], a large diverse group of animals including groups such as Brachiopoda, Nemertea, Annelida, Mollusca, Phoronida etc. whose genome has been understudied in retroelements study. This and other studies e.g. [10, 53] provides a foundation of knowledge that can be built upon to understand the role of retrotransposons in non-model and marine animals.

## Materials and methods

### Genomic sequence

Assembled whole genomic sequence of the siboglinid annelid *Lamellibrachia luymesi* generated by Li et al. ([5];WGS project - SDWI01, Bio project number - PRJNA516467 and Bio sample number - SAMN10789628) was accessed from NCBI [54]. Li et al. conducted a scaffold-level assembly of the genome using Illumina paired-end and mate-pair and sequence data. The total sequence length is 688MB with an overall BUSCO genome completeness of 95%.

### Identification of LTR retrotransposons

This study focused only on intact LTR retrotransposons, solo and nested insertions without coding domains were excluded from the analysis. We defined intact LTR retrotransposon as possessing two LTRs, at least one protein domain and a pair of TSD (Target site duplication) regions.

The bioinformatics pipeline (Figure 2) used to identify LTR retrotransposon candidates in the *L. luymesi* genome included two software tools for *de-novo* prediction of LTR retrotransposons, LTRharvest genometools v1.5.10 [55] and LTR_Finder v1.07 [56]. Both programs were run to provide a more thorough search for putative LTR elements and was based on previously published approaches [9]. In addition, LTRharvest tend to have greater sensitivity whereas LTR_Finder has a lower false-positive rate [57].

To prepare data for LTRharvest, genomic scaffolds were run through Suffixerator (also part of the genometools package) with default parameters to create an enhanced suffix file which is then scanned by LTRharvest. The following LTRharvest parameters were used to obtain LTR retrotransposon candidates with TGCA motifs ‘-minlentltr 100, -maxlenltr 7000, -mintsd 4, -maxtsd 6, -similar 85, -vic 10, -seed 20, -motif TGCA, -motifmis 1.’ In contrast, to obtain LTR retrotransposon candidates without TGCA motifs, parameters were set to ‘-minlentltr 100, -maxlenltr 7000, -mintsd 4, -maxtsd 6, -similar 85, -vic 10, -seed 20’. These 2 approaches were taken to obtain a more robust putative LTR retroelements list from LTRharvest. Similarly, to obtain candidates with both TGCA and non-TGCA motifs the following parameters were used to run LTR_Finder ‘-D 15000, -d 1000, -l 100, -L 7000, -p 20, -C, -M 0.85’. In summary, parameters for both programs were set to minimum and maximum LTR length of 100 bp and 7000 bp respectively and at least 85% identity between two LTR regions.

LTR_retriever v2.8.5 [58] with default parameters was used to filter out false positives LTR candidates identified by LTRharvest and LTR_Finder. This downstream filtering was largely based on boundary mapping of LTRs, presence of TSDs and presence of palindromic motifs. The palindromic motif library employed by LTR_retriever includes – TGCA, TGCT, TACA, TACT, TGGA, TATA, TGTA, and TCCA.

### Classification of discovered LTR retrotransposons

Classification of LTR retrotransposons is dependent upon the presence and order of protein domains within the *pol* gene [11] (Fig 1). LTR_retriever based the classification of LTR retrotransposons on identification of conserved protein domains of each LTR retrotransposon candidate using profile Hidden Markov Models (pHMMs) of LTR retrotransposon domains from Pfam database [30]. Elements returning ambiguous pHMMs matches were classified as unknown.

To refine classification, we employed the program TEsorter v1.2.5 [59] which translated nucleotide sequence of LTR retrotransposon candidates in all six frames and searched these sequences against HMM profiles obtained from existing mobile elements protein databases – specifically , REXdb [14] and Gyspsy database of mobile genetic elements [60]. For each domain of a sequence, only the best hit with highest score is retained. Classification into superfamilies and families were based on hits of the *pol* and *gag* genes to curated database. Elements lacking at least one domain were not classified.

To do this step, fasta sequences of LTR retrotransposon candidates were first extracted using the call_by_seq_list.pl script from LTR_retriever package. Obtained sequences were then input into TEsorter (parameters = ‘-db gydb, -st nucl and -p 10’) for further classification.

### Naming conventions

To facilitate communication, naming conventions for LTR retrotransposons families and elements identified in this study were created. Gypsy families were designated as LGF (*Lamellibrachia* Gypsy Family), followed by a unique number (e.g., LGF1, LGF2 etc.), Copia families were designated as LCF (*Lamellibrachia* Copia Family), followed by a unique number (e.g., LCF1) while Bel-pao families were designated as LBF (*Lamellibrachia* Bel-pao Family), followed by a unique number (e.g., LBF1). For individual elements, identified LTR retrotransposons were designated as LLXY#, where LL denotes 2 letters representing *L. luymesi,* XY denotes the first two letters of the superfamily it belongs to and # denotes the element number (e.g., LLGY1 represents a Gypsy element).

### Phylogenetic analysis

Phylogenetic analysis was used to further validate family-level assignment of these elements and to access the evolutionary position of *L. luymesi* LTR retrotransposon candidates. For this purpose, amino acid sequences of INT, RT and RH domains were extracted from the LTR retrotransposon candidates following the guideline from TEsorter package. Gag and Protease (PR) sequences were excluded from analyses as they are known for their variability which prevents reliable alignments [61, 62].

To infer phylogenetic trees, amino acid sequence of INT,RH and RT from other known organisms were obtained from the GYDB database and recent studies [47, 53, 63], and aligned using MAFFT v7.407 [64] to amino acid sequence of INT, RT and RH from LTR retrotransposons found in *L. luymesi* genome. Each of the 3 domains was analyzed separately and a combined analysis was not done due to difference in taxon sampling and the fact that the domains may have distinct evolutionary histories. Maximum likelihood with bootstrap analysis was employed to construct phylogenetic trees using IQtree v1.6.12 [65] with the following parameters ‘-bb 100000, -nt AUTO, --runs 5’. The substitution model employed by IQtree for the INT domain tree was LG+R7, the RT domain tree was LG+F+R6 while the RH domain tree was LG+R7. Phylogenetic trees were mid-point rooted, visualized and edited using Figtree v1.4.2 [66].

### Estimation of insertion time

Time since initial insertion of LTR retrotransposon candidates was estimated using scripts implemented in the LTR_retriever package. Insertion time were calculated as T = K/2 μ, where K is the divergence rate measured by the Jukes-Cantor model with K = − 3/4*ln (1-d*4/3) [67] and μ is the neutral mutation which is set at 1.3 × 10^− 8^ mutations per bp per year [68].

## Supplementary Information

**Additional file 1: Table S1.** Details (size, domain present e.t.c.) of elements excluded from further analysis. **Table S2.** Details of each LTR-retrotransposons found in *Lamellibrachia luymesi* genome, including element sizes, LTR sizes and pair identity, TSD sizes and sequences,motif sequences.

**Additional file 2.** Integrase sequence alignment file.

**Additional file 3.** Ribonuclease H sequence alignment file.

**Additional file 4.** Reverse transcriptase sequence alignment file.

## Data Availability

Assembled whole genomic sequence of *Lamellibrachia luymesi* generated by Li et al. was accessed from NCBI repository (https://www.ncbi.nlm.nih.gov/bioproject/PRJNA516467/) with WGS project - SDWI01, Bio project number - PRJNA516467 and Bio sample number -SAMN10789628. All data generated or analyzed during this study are included in this published article (and its supplementary information files). Scripts for bioinformatic analyses generated herein are available at https://github.com/clavia96/LTR_retrotransposon.git.

## Abbreviations

LTR: Long Terminal Repeat
RT: Reverse Transcriptase
RH: Ribonuclease H
INT: Integrase
HMM: Hidden Markov Models
BSV: Bootstrap Value
GYDB: Gypsy Database of Mobile Genetic Elements
MYA: Million Years Ago
