# Supplementary material for: Genome-wide characterization of LTR retrotransposons in the non-model deep-sea annelid Lamellibrachia luymesi
Source: BMC Genomics. 2021 Jun 23;22:466. doi: 10.1186/s12864-021-07749-1 (PMC8220671; doi:10.1186/s12864-021-07749-1)
Supplement: Supplementary file 2 — Additional file 2. Integrase sequence alignment file. [file 12864_2021_7749_MOESM2_ESM.docx]

>NC-256_Flow

------------------------------------------VTRLIVTEYHQKLR---H

V-GV--------EHVFN-HLRER------------FWILRGRA----------EVKEC--

-----TIK-CPL-CHRRRV------QP-MTQKMSD--LPAVR------------LAGVST

PFKHVGLDY-----AGLFQ---VRVGR---NRVEKR----YVCLFTC----LHM------

--RAVH-L--------ETA----HSL---DSF-----IM-----ALRRFRARRG------

--------------NPVRIMSDN-GSNFVGAE-------RELRDALQE--LSQER-----

IADEL----SVHGV--KW---DFNPPDGPWFGGAW--EALVKSTKR--------------

------------------------------------------------------------

------------------------------------------------------------

------------------------------------------------------------

------------------------------------------------------------

------------------------------------------------------------

--------------------------------------

>NC-228_Dan

------------------------------------------ITRLLIKQYDAKLG---H

P-GP--------ERVFA-EMRRY------------YWILRGRE----------AIRRH--

-----QHS-CVE-CQRWRA------KP-NIPKMAE--LPPAR------------LRLMKP

PFFSTGVDC-----FGPFL---VKRGR----SNEKK----WGIIFKC----MTT------

--RCVH-L--------DLL----ANMD-TDSF-----LM-----ALRRMVARRG------

--------------TPSEILADQ-GTNFRGGD-------KELQTAFTA--MSPD------

LQAQL----AKQKI--QF---HYNPPNAPHFGGMW--EREIRSVKA--------------

------------------------------------------------------------

------------------------------------------------------------

------------------------------------------------------------

------------------------------------------------------------

------------------------------------------------------------

--------------------------------------

>NC-290_Dan

---------------------------------------------LLIKKYDNQLL---H

P-GP--------ERVLG-EIRRK------------YWILRGRE----------SIKRH--

-----QYN-CET-CQKWRA------KP-VIPKMAD--LPPSR------------LRLYKP

PFWSTGVDC-----FGPFT---IKIGR----RTEKR----WGIIFKC----MTT------

--SCIH-L--------DLL----ESMD-TDAF-----LM-----ALRRFVSRRG------

--------------KPFEILADR-GTNFRGGA-------TELQEAFTA--LEAS------

LKEQL----AGQEI--TF---QFNPPHAPHFGGTW--EREIRSVKT--------------

------------------------------------------------------------

------------------------------------------------------------

------------------------------------------------------------

------------------------------------------------------------

------------------------------------------------------------

--------------------------------------

>NC-219_Dan

-------------------------------------------TKLLIKETDQRLL---H

P-GS--------ERVLA-ELRRQ------------YWVLRGRE----------AIRKH--

-----QHT-CRD-CQFWRA------KP-QTPQMAD--LPSSR------------LQLYKP

PFYSTGVDC-----FGPFT---VKVGR----RQEKR----WGVLYKC----MTT------

--RCVH-L--------DLL----EQLD-TDAF-----LL-----SLRRFIARRG------

--------------KPMELLCDN-GTNFVGGD-------RELRETFNA--MAPK------

LQEQL----AEQRI--RF---RFNPPSAPHFGGTW--EREVKSVKS--------------

------------------------------------------------------------

------------------------------------------------------------

------------------------------------------------------------

------------------------------------------------------------

------------------------------------------------------------

--------------------------------------

>NC-183_Dan

----------------------------------------HHITKLLIKETDQQLL---H

P-GS--------ERVLA-ELRRQ------------YWILRGRQ----------AVRKH--

-----QHT-CQD-CQIWRA------KP-QTPRMAD--LPPCR------------LNLYKP

PFYSTGVDC-----FGPYA---VKIGR----RQEKR----WGIIYKC----LTT------

--RCVH-L--------DLL----EHMD-SDAF-----LL-----SLRRFIARRG------

--------------KPMELLCDN-GTNFIGGD-------RELRESFEA--MSPK------

LQEQL----AEQKI--SF---RHNPPNAPHFGGTW--EREIKSVKT--------------

------------------------------------------------------------

------------------------------------------------------------

------------------------------------------------------------

------------------------------------------------------------

------------------------------------------------------------

--------------------------------------

>NC-1118_Dan

---------------------------------------------------DSKLG---H

P-GA--------DRVYA-HLRRY------------YWILKGRQ----------AVRKY--

-----QRTKCLD-CVKLKG------KP-IIPKMAD--LPPSK------------LNLFKP

LFWSTGMDC-----FGPFT---VKTGR----KTGKR----WGLLFKC----QTT------

--RCVH-L--------ELL----TGLD-TDSF-----LM-----ALRRFVARRG------

--------------QPYEVICDQ-GTNFHGGE-------SELKNAFEH--LSPV------

LKEKL----WNHQI--KF---TYNPPYAPHFGGTW--EREIRSIKS--------------

------------------------------------------------------------

------------------------------------------------------------

------------------------------------------------------------

------------------------------------------------------------

------------------------------------------------------------

--------------------------------------

>JULE

-IP-------------------------------------PSYRQKVLKDLHHE-----H

P-GI--------CRMKA-LARSY------------LWWPGCDG----------DIQEL--

-----VQS-CQI-C---------------------------------------------Q

AVQRIHIDF-----A-EK---------------DKH----YFLVVIG----SHS------

--KWLE-VF-------PMT----SI-T-SH------NTI-EI--L-RRLFSSYG------

--------------LPEELVSDN-GPQLVSQ---------EFCQFLEL------------

-----------NGI--RH---TAVPAYHPASNGAA--ERSVQILKRS-------------

------------------------------------------------------------

------------------------------------------------------------

------------------------------------------------------------

------------------------------------------------------------

------------------------------------------------------------

--------------------------------------

>Pyg1

-VP-------------------------------------KPGRAKLLEFLHEG-----H

P-GI--------VRMKA-LARSH------------VWWPGIDS----------DIQDK--

-----VQA-CTE-CQ--LQ------RP-V-PPVAPL-HSW---------------DWPDR

PWSRIHVDY-----AGPF---------------LGQ----MFLVVVD----SYS------

--KWLE-VV-------PTN----SS-T-ST------VTI-AK--L-RQIFAEHG------

--------------LPDKLVSDN-GPCFISE---------EFETFLRE------------

-----------NGI--QH---VKISPHHPATNGLA--ERSVRIFK---------------

------------------------------------------------------------

------------------------------------------------------------

------------------------------------------------------------

------------------------------------------------------------

------------------------------------------------------------

--------------------------------------

>JRE

-IP-------------------------------------EQGRAGLLEQLHQS-----H

P-GM--------SRMKG-LARSY------------LWWPKLDA----------DIEAR--

-----VTN-CTV-CQ--EQ------RK-A-PVGAPL-HLW---------------EWPRQ

PWRRVHMDY-----AGPF---------------LGK----MFLILVD----AHS------

--KWIE-AY-------PIN----SA-T-TA------TTL-EY--L-RKSFSTHG------

--------------IPEMMVSDN-AQCFVSE---------ASKEFMSR------------

-----------NGI--TH---VTSAPYHPSSNGLA--ERAVQTFK---------------

------------------------------------------------------------

------------------------------------------------------------

------------------------------------------------------------

------------------------------------------------------------

------------------------------------------------------------

--------------------------------------

> steamer

-IP-------------------------------------RCMRRDILDQIH-T-----H

I-GV--------EGCLN-RARQC------------VFWPNMTS----------EIKDF--

-----IGK-CEA-CQ--SF-----------------------------------------

-----------------------------------------YLVTVD----YFS------

--NFFE-ID-------KLE----DM-T-SR------CVI-GK--L-KQHFARHG------

--------------IPNQLVSDN-AQTFKSE---------KFKQFTLQ------------

-----------WDF--EH---VTSSARYPQSNGKA--ESAVKRAK---------------

------------------------------------------------------------

------------------------------------------------------------

------------------------------------------------------------

------------------------------------------------------------

------------------------------------------------------------

--------------------------------------

>LLGY1

---------------------------------------------------HEG-----H

Q-GI--------VKTKE-LLRSK------------VWFPKMND----------MVETA--

-----VRH-CFA-CQC-TY------NG-N-PNLEPM-QMS---------------DMPPA

AWKHLSMDF-----LGPL--------P------SGE----ELMVLVD----EYS------

------------------------------------------------------------

------------------------------------------------------------

------------------------------------------------------------

------------------------------------------------------------

------------------------------------------------------------

------------------------------------------------------------

------------------------------------------------------------

------------------------------------------------------------

--------------------------------------

>LLGY2

---------------------------------------------------HEG-----H

L-GI--------EKCKR-RARDI------------LYWPNMNK----------DVYDT--

-----VSR-CDV-CQ--EY------RY-A-QQQQPL-QMH---------------ERPDR

PWAKVACDI-----F-YL---------------KQV----PYLLTVD----YYS------

--HYPE-IA-------LLS----NE-S-SR------QVI-IH--L-KSLFSRYG------

--------------IPSECISDG-GPQFASE---------EFRQFTSE------------

-----------WGI--EH---KMSSPYYPQSNGLA--ENGVKIVKRLLR-----------

---KA----ADRKEDAYLALLAYRSSP---------------LD-----CGKSPAEL---

---------------LFG-----RKIRT---RL---------------------------

------------------------------------------------------------

------------------------------------------------------------

------------------------------------------------------------

--------------------------------------

>LLGY4

------------------------------------------------------------

------------------------------------------------------------

------------------------------------------------------------

PSERVHINY-----AGSV---------------EGK----MLLIVVD----SYS------

--KWPE-VV-------IQN----CT-T-SE------ATV-NA--L-RTIFSRGG------

--------------IPHTLVSDN-GPQFKSQ---------EFKDFLDW------------

-----------LGV--LH---KPTSPYHPSSNGQA--ERFVQTVKQALKA---------M

A-SSG----ESLQVRLDKFLLAYKNAP---------------HAF----TGELPAVR---

---------------FMG-----TQLRTRLDSVK--------------------------

------------------PHNR--------------------------------------

--------------------------------R-ENKQLEK---Q---------------

------------------------------------------------------------

--------------------------------------

>LLGY5

---------------------------------------------------HAA-----H

Q-GV--------TSMTA-RANVS------------VFWPGITT----------DIARL--

-----RNS-CMD-CN--RI------SP-S-QPNAPP-TTP---------------VDPEF

PFQCICADY-----F-TY---------------KGA----HYLIIVD----RYS------

--NWPI-IK-------KTS-----G-G-AA------GLV-KS--L-REEFITYG------

--------------IAEELASDG-GPEFVAT---------ETQEFLKS------------

-----------WGV--RH---RLSSVAYPHSNCRA--EIGVKSCKRLLMH--------NT

G-PNG----ELDTPSFQRAMLQYRNTP---------------DQD----TKMSPAMI---

---------------VFG-----RSIRD---FI---------------------------

------------------PVL---------------------------------------

----------------------------------------P---GRY-------------

------------------------------------------------------------

--------------------------------------

>LLGY6

---------------------------------------------------HAA-----H

Q-GV--------SMMTA-RAESA------------VFWPGISA----------DISTT--

-----RKN-CEH-CH--RM------AP-S-QPGAPP-IPP---------------IPVVY

PFQAVCSDF-----F-VH---------------RGV----HYLVTVD----RYS------

--NWPI-IS-------QST-----G-G-AT------GLI-DH--L-RRAFVTYG------

--------------TPEELASDG-GPEFTST---------ETRSFLHR------------

-----------WGV--HH---RLSSVAFPHSNCRA--EIGVKTMKRLITD--------NT

G-PKG----ELDTDAVQIAILQYRNTP---------------DPD----TKISPAMC---

---------------VFG-----RPTRD---FI---------------------------

------------------PII---------------------------------------

----------------------------------------P---GK--------------

------------------------------------------------------------

--------------------------------------

>LLGY7

---------------------------------------------------HDS-----H

Q-GV--------DRTKR-RARQS------------VYWPGISN----------DIATT--

-----VSS-CDK-CQ--ER------LP-S-QQREPM-RAE---------------SPPSR

AFEDVSADF-----F-NY---------------KGR----DYLVYVD----RLS------

--GWPA-VI-------HFPKG--TT-T-SR------HTI-HA--C-ARLFVDLS------

--------------IPVRFRSDG-GPQFASR---------EFQQFLKR------------

-----------WDV--VA---APSTPHFAQSNGHA--ESAVKAVKKLIATTT--------

--VRG----DLDDENFQRGLLEYRNTP---------------RVG-----GLSPAQI---

---------------LFG-----HPLRSAM------------------------------

------------------------------------------------------------

------------------------------------------------------------

------------------------------------------------------------

--------------------------------------

>LLGY8

------------------------------------------------------------

------------------------------------------------------------

------------------------------------------------------------

-----------------------------------R----DYIVAVD----CYS------

--KYPE-TA-------LLE----NK-T-AS------NVI-IH--L-KSIFARHG------

--------------IPEEMMSDN-MP-FASQ----------FTNFGRD------------

-----------WGI--KI---TTSSPNFPQSNGQS--ERAVQTRKRILK-----------

---KA----DCEGRDPYVALLEYRNTP---------------VAG----ALFSPAQM---

---------------LMS-----RMLRA---KL---------------------------

------------------PARS--------------------------RLLT--------

-----PQVVS-----------------AQCQLQ-QRQDKYK---QYYDRGS-------KR

L-----------------------------------------------------------

--------------------------------------

>LLGY11

------------------------------------------------------------

------------------------------------------------------------

---------------------------------------------------------GSY

PMEFVAADL-----IGPF----TESHN------GNK----YILTIID----FCT------

--GWAE-AI-------PIP----TK-S-NQ------AVW-DA--FSNGFICRHG------

--------------VCRVLLTDH-GAEFTAL---------AFERYLSQ------------

-----------IGI--EH---RMSTPAHPQSNGKI--ERFNRTLKQMIQKAV-N----NQ

P-----------------------------------------------------------

-----------------G------------------------------------------

------------------------------------------------------------

------------------------------------------------------------

------------------------------------------------------------

--------------------------------------

>LLGY12

------------------------------------------------------------

--GA--------DKCKA-RGRTV------------LYWPGMSQ----------DIETI--

-----VGI-CHI-CL--KF------RA-S-NPKEPL-IPH---------------DVPER

PWQKVAADT-----M-TF---------------KSR----DYIVAFD----CYS------

--KYPE-IA-------LLE----KK-T-AS------NVI-IH--L-KSTFARHG------

--------------IPEEMMSDN-MP-FASQ---------EFTNFGRD------------

-----------WGL--KL---TTSSPNFPQSNGHR--ERAVQTLKRLLK-----------

---KA----DCEGRDPYVALLEYRNTP---------------VAG----ALFSPAPL---

---------------F--------SLRA---KL---------------------------

------------------PARS--------------------------RLLT--------

-----PQVVS-----------------AQC-LQ-HRQDKYK---QYYNRGS-------KR

L--TEL-------------Q-----PCSVV--------RVRH------------------

ENVWE---------------------------------

>LLGY13

---------------------------------------------------HSS-----H

Q-GI--------EATLR-RAKDS------------IYWPGMTN----------DIKQM--

-----IKS-CQA-CS--KE------KP-S-QQKETL-RSH---------------DLLSK

PWAKVGIDL-----F-TY---------------ANA----TYLIMVD----YYS------

--DFFE-FT-------KLV----DQ-R-AE------TTI-QA--C-KEQFARYG------

--------------VPQIVQSDG-GPQFISS---------EFQAFANN------------

-----------WEF--KH---SMSSPYH-QSNGKA--ESAV-------------------

------------------------------------------------------------

------------------------------------------------------------

------------------------------------------------------------

------------------------------------------------------------

------------------------------------------------------------

--------------------------------------

>LLGY14

---------------------------------------------------HAA-----H

Q-GV--------TSMTA-RANVS------------VFWPGITT----------DIARL--

-----RNS-CMD-CN--RI------SP-S-QPNAPP-TTP---------------VDPEF

PFQCICADY-----F-TY---------------KGA----HYLIIVD----RYS------

--NWPI-IK-------KTS-----G-G-AA------GLV-KS--L-LEEFITYG------

--------------IAEELASDG-GPEFVAT---------ETQEFLKS------------

-----------WGV--RH---RLSSVAYPHSNCRA--EIGVKSCKRLLMH--------NT

G-PNG----ELDTPSFQRAMLQYRNTP---------------DQD----TKMSPAMI---

---------------VFG-----RSIRD---FI---------------------------

------------------PVL---------------------------------------

----------------------------------------P---GRYT------------

------------------------------------------------------------

--------------------------------------

>LLGY15

---------------------------------------------------HNA-----H

S-GI--------VRMKA-VGRSF------------MWWPGIDS----------DIERT--

-----VNS-CDI-CR--RS------RH-K-PTEAPL-QPW---------------SFPDR

PWSRVHIDY-----AGPV---------------MGK----MILVVID----AHS------

--KWIE-AY-------TTS----GS-T-SA------ITI-SK--L-KWIFSSHG------

--------------IPDVIVSDN-ATGFVSE---------EFQSFCRR------------

-----------NGI--KH---VTSAPHHPATNGLA--ERAVGILKGGVQR---------L

Q--------GDLETRIAHFLLDYRITP---------------HTT----TGVSPAEL---

---------------LTG-----RKLRTRLDRII--------------------------

------------------PDVSGR------------------------------------

-------------------------------AI-SKQTTQK---ERHDQ--------HTQ

A--RQY-------------Q-----PGDLV--------YALM-YRGN-------------

KTNWSPGTVVTQ--TGPVSYTVRLE-------------

>LLGY17

---------------------------------------------------HQS-----H

R-GV--------VRMKT-MARLY------------VWWPNIEA----------SVEAC--

-----CKA-CNV-CA--VT------AP-A-PT-ANL-SPW---------------PLPDE

PWDRIHVDF-----AGPF---------------LGN----MWMLVMD----AYS------

--KWPS-VV-------RMS----KYPT-TE------TTI-MA--L-NILFTTWG------

--------------SPKTLVSDN-GPQFGSK---------QFEDWCRL------------

-----------NGI--VH---LTSAPFHPPSNGEA--ERLVGVFKTAMQR--------SV

G-EEG----KERDKATMGFLREYRSTP---------------NCA----TGRTPAEL---

---------------MIG-----RQVRTPLSLLQ--------------------------

------------------PSVHH-------------------------------------

------------------------------------------------------------

------------------------------------------------------------

--------------------------------------

>LLGY18

------------------------------------------------------------

------------------------------------------------------------

-----------------------------------------------------------L

QWEKVGVDL-----F-SW---------------EGR----DYQVIVD----YTS------

--NFWE-VD-------RMN----ST-T-TT------SMI-KQ--L-KSHFVRFG------

--------------IQSVVVSDN-GPQYVSE---------EFCTFAAK------------

-----------WDF--EH---QTSAPGH--------------------------------

------------------------------------------------------------

------------------------------------------------------------

------------------------------------------------------------

------------------------------------------------------------

------------------------------------------------------------

--------------------------------------

>LLGY20

---------------------------------------------------HEG-----H

L-GI--------VKTKA-LMRQK------------VWFPSMDK----------LVEAK--

-----VKS-CLA-CQ--IA------TP-V-TSREPL-QMS---------------RLPDQ

PCEEMSVDF-------AH--------V------DGE----TLLILID----DYS------

--RFPF-IE-------PVT----SE-A-AC------AVI-PK--I-DKIFAMFG------

--------------TPDVLKSDN-GPPFNGQ---------DFAKFANV------------

-----------LGF--KH---RKVTPLWPRANGEV--ERFVKTLKKCVKAAK--------

--SDG----KNWRKEMQAFLRNYRTSP---------------HAT----TGVAPSTL---

---------------FLK-----RAVRNKL------------------------------

------------------P-----------------------------------------

------------------------------------------------------------

------------------------------------------------------------

--------------------------------------

>LLGY21

---------------------------------------------------HEG-----H

L-GI--------VKTKA-LMRQK------------VWFPSMDK----------LVEAK--

-----VKS-CLA-CQ--IA------TP-V-TSREPL-QMS---------------RLPDQ

PCEEMSVDF-------AH--------V------DGE----TLLILID----DYS------

--RFPF-IE-------PVT----SE-A-AC------AVI-PK--I-DKIFAMFG------

--------------TPDVLKSDN-GPPFNGQ---------DFAKFANV------------

-----------LGF--KH---RKVTPLWPRANGEV--ERFVKTLKKCVKAAK--------

--SDG----KNWRKEMQAFLRNYRTSP---------------HAT----TGVAPSTL---

---------------FLK-----RAVRNKL------------------------------

------------------P-----------------------------------------

------------------------------------------------------------

------------------------------------------------------------

--------------------------------------

>LLGY22

---------------------------------------------------HNA-----H

S-GI--------VRMKA-VGRSL------------MWWPGIDQ----------DIEKT--

-----AKT-CDI-CM--RS------RP-R-PTEAPL-QPW---------------SFPDR

PWSRLHIDY-----AGPF---------------MGR----MMLVVID----AHS------

--KWID-AH-------LTS----GS-T-SA------ITI-SK--L-RQSFSTHG------

--------------IPDVIVSDN-ATGF--------------------------------

----------------------------------------------GVSR---------F

------------------------------------------------------------

------------------------------------------------------------

------------------------------------------------------------

------------------------------------------------------------

------------------------------------------------------------

--------------------------------------

>LLBP1

------------------------------------------------------------

----------------------G------------YWIIKARA----------TVTSY--

-----LWN-CVK-CRKMRG------GT-VTQKMAE--LPEDR-------------LEPSD

PFTYSAVDF-----FGPFF---IKEGR----SEKKK----WGVLFTC----MAS------

--RAVH-I--------ETA----NSLS-TDSF-----IN-----AYRRFVGRRG------

--------------PVRQLRSDR-GTNFVGAR-------SELEAALAE--MNDGK-----

ITAEL----LKQNC--DWVTFKMNPPHASHMGGVW--ERMIRSVRNVLSALL-----NAH

GDRLD---DEQLRTLMVEAEAVVNSRPITYP----------DTTVPDSGEPLSPS-I---

---------------LTL-----KSR----------------------------------

---------------VVLPPPGI-------------FMKEDLYCRKRWRLVQ--------

-----FLANQF----WNRWR--------TEYLL-ALQERSKW---------------NKQ

H--HNL-------------K-----TGDIV--------LVKD-ECNPR----CQWPLARV

NQIYPS----EDGLVRKVKV------------------

>LLGY23

---------------------------------------------------HSA-----H

Q-GV--------SSMES-RARSI------------VFWPGIST----------AIQET--

-----RDR-CRS-CN--KT------AP-S-QAATPP-AAL---------------DTPST

PFESVFADF-----C-DY---------------GGC----HYLVVGD----RLS------

--GWVD-IY-------KTPPGTPYS-G-AT------GLI-AC--L-RQMFATFG------

--------------VPEILSSDG-GPEFTAS---------ETSNFLSR------------

-----------WGV--HH---RISSVAFPQSNGRA--EVAVKKAKRTLMD--------NI

G-PTG----SLDNDGLLRAMLQLRNTP---------------DPD----CNVSPAEV---

---------------IFG-----RPIRDAFSFVNRCTKFENPSIRPMWREAWSAKENAMR

ARFARTSEALNAHSRALPPLVI--------------------------------------

--------------------------GARVYVQ-NQRGPHP---NKWDRSG---------

------------------------------------------------------------

-------------VVVDVG-------------------

>LLGY24

---------------------------------------------------HVG-----H

Q-GV--------VKTKM-LLREK------------VWFPAIDS----------MAERQ--

-----VKS-CLA-CQATIS------TP-M-TP-EPI-IST---------------PIPSA

PWKNLSADF-----LGPL--------P------TGE----LILVVID----DFS------

--RFPE-VE-------IVT----ST-A-AS------SVI-PK--L-DSMFARHG------

--------------IPDVLKSDN-GPPFNGS---------ELTKFAQY------------

-----------LGF--QH---KKITPIWSCANGEA--ERFMAPLMKAIRAAH--------

--VEN----RSWKQELYNFLRQYRATP---------------HCT----TGVSSAEA---

---------------LYG-----RKLN---------------------------------

------------------------------------------------------------

------------------------------------------------------------

------------------------------------------------------------

--------------------------------------

>LLGY25

---------------------------------------------------HAA-----H

Q-GV--------TSMTA-RANVS------------VFWPGITT----------DIARL--

-----RNS-CMD-CN--RI------SP-S-QPNAPP-TTP---------------VDPEF

PFQCICADY-----F-TY---------------KGA----HYLIIVD----RYS------

--NWPI-IK-------KTS-----G-G-AA------GLV-KS--L-REEFITYE------

--------------IAEKLASDG-GPEFVAT---------ETQEFLKS------------

-----------WGV--RH---RLSSVAYPHSNCRA--EIGVKSCKRLLMH--------NT

G-PNG----ELDTPSFQRAMLQYRNTP---------------DQD----TKMSPAMI---

---------------VFG-----RSIRD---FI---------------------------

------------------PVL---------------------------------------

----------------------------------------P---GRYT------------

------------------------------------------------------------

--------------------------------------

>LLGY26

---------------------------------------------------HEG-----H

L-GI--------EKCKR-RARDI------------LYWPNMNK----------DVYDT--

-----VSR-CDV-CQ--EY------RY-A-QQQQPL-QMH---------------ERPDR

PWAKVACDI-----F-YL---------------KQV----PYLLTVD----YYS------

--HYPE-IA-------LLS----NE-S-SR------QVI-IH--L-KSLFSRYG------

--------------IPSECISDG-GPQFASE---------EFRQFTSE------------

-----------WGI--EH---KMSSPYYPQSNGLA--ENGVKIVKRLLR-----------

---KA----ADRKEDAYLALLAYRSSP---------------LD-----CGKSPAEL---

---------------LFG-----RKIRT---RL---------------------------

------------------------------------------------------------

------------------------------------------------------------

------------------------------------------------------------

--------------------------------------

>LLGY28

------------------------------------------------------------

-------------------AREV------------LYWPGMSA----------EVRDY--

-----VSR-CST-CQ--TF------MP-T-QCREPL-QPH---------------ELPSR

PWEKVGGDL-----F-EL---------------AGQ----TFLIMVD----YWS------

--NYFE-IA-------ELH----KK-T-SL------SVI-AQ--F-KVQFARHG------

--------------IPSVVMTDN-GPEFASH---------EFEEFAKT------------

-----------WKF--EH---ITSSPRFPQSNGKA--ENAVKTCKALLM-----------

---KA----RKDRQDPLLALLAWRNTP---------------SEG----FNTSPVQR---

---------------LMG-----RRTRT---L----------------------------

------------------------------------------------------------

------------------------------------------------------------

------------------------------------------------------------

--------------------------------------

>LLGY30

A--------------------------------------------------HQG-----H

Q-GM--------VKTKR-LIREK------------VWFPGIDV----------LVEKR--

-----VKR-CMA-CQASTH------LP-E-SSMEPL-KMS---------------KLPEG

PWQHVDIDF-----CGPF--------P------SGD----YLLVAID----EYS------

--RFPE-VE-------ITR----ST-S-AY------STI-PK--L-DKIFSTHG------

--------------IPEVVKSDN-GPPFQSS---------EFKSFAEY------------

-----------TGF--QH---RKITPEWPQANSEV--ERFMRTLEKAIRCAI--------

--LEG----KVWKQEMYRFLRSYRATP---------------HSS----TGVSPATA---

---------------LFN-----RNIKTTL------------------------------

------------------PENKEASKSD--------------------------------

-------------------------RTMREADA-RAKSRMK---QYADKRA------KAK

S--SNL-------------Q-----PGDIV--------MMKQ----------------RR

TNKYSTPY-------QPMAYEVVARQGP---------M

>LLGY31

---------------------------------------------------HEA-----H

Q-GI--------EKTRL-RARSC------------VYWKAINR----------DIDDI--

-----VRK-CDT-CQ--QL------QR-R-QAHEPL-MQH---------------ELPTR

PWQIVGTDL-----F-VI---------------RLD----TYLLMCD----YYS------

--KFPF-VY-------RIEG---RV-T-SD------AII-SK--M-SEVFAENG------

--------------SPDKVVSDN-GGHYSSQ---------AFRNFANE------------

-----------WCF--DH---VTSSPHFPQSNGFI--ERQVQTVKSTLK-----------

---KA----AMTR-----------------------------------------------

------------------------------------------------------------

------------------------------------------------------------

------------------------------------------------------------

------------------------------------------------------------

--------------------------------------

>LLGY32

---------------------------------------------------HSA-----H

Q-GI--------SAMTA-RAESS------------VFWPGITP----------AIAAV--

-----RTN-CSD-CN--RM------AP-S-QPSAPP-TPP---------------VLPVY

PFQCVCSDF-----F-TY---------------KGN----SYLVIVD----RYS------

--NWPI-IE-------RTT-----G-G-AD------GLI-DS--L-RRSFVTYG------

--------------IPDELASDG-GPEFTST---------TTRLFLKT------------

-----------WGV--HH---RLSSVAFPHSNCRA--EIGVKTVKRLITN--------NT

G-TNG----ELDTDGVQRAILQYRNTP---------------DPD----TKLSPAMC---

---------------VFG-----RPIKD---FI---------------------------

------------------PIL---------------------------------------

----------------------------------------P---GRY-------------

------------------------------------------------------------

--------------------------------------

>LLGY33

------------------------------------------------------------

------------------------------------------------------------

------------------------------------------------------------

---------------------------------GTS----YLFTIVD----RFT------

--RWPE-AI-------PMA----DA-T-AV------SCA-RA--LLENWVPRFG------

--------------VPTDIVSDR-GRQFISG---------LWMELGKL------------

-----------LGM--QL---HHTTAYHAQSNGLV--ERFHRQLKASLKARL--------

---HD----PDWRDELPIVLLGIRCSI---------------KED----LGCTSTEL---

---------------VYG-----TTLRL--------------------------------

------------------PGEFFETTKASTEVTT-------YAL-ALLTRLR--------

-----NTM-----------------RSLQAKSM-THHG--------RQHV-------SRI

P--TAL-------------L-----TCTFV--------FVCK-DAHR----------TPL

ECPYEGPFSV----------------------------

>LLGY34

------------------------------------------------------------

---------------------------------------------------------T--

-----VSR-CDV-CQ--EY------RY-A--QQQPL-QMH---------------ERRDR

PWAKVACDI-----F-YL---------------KQF----PYLLMVD---YYYS------

--HYPE-IA-------LRS----NE-S-SR------QVI-IH--L-KSLFSRYG------

--------------IPSECISDG-GPQFASE---------EFRQFTSE------------

-----------WGI--EH---RMSSPYYPQSNGLS--EN---------------------

------------------------------------------------------------

------------------------------------------------------------

------------------------------------------------------------

------------------------------------------------------------

------------------------------------------------------------

--------------------------------------

>LLGY35

--------------------------------------------------LHAG-----H

Q-GI--------EKCKL--AKSC------------IYWDGINN----------DLEEM--

-----VKC-CPT-CQ--QL------RK-S-NAKETL-MPQ---------------EVPTR

AWQILGTDL-----F-HF---------------NDN----EYLIIAD----YYS------

--KYPF-VC-------KMPK---PC-T-SH------AFV-SV--T-KGLLSEQG------

--------------VAERIVGDN-GRHFDCV---------NYRSFAET------------

-----------WGF--DH---ITSS---PQSNGFI--ERCIQTVKNTLT-----------

---KA----RESQMDPNMAMLCLRTTP---------------IDH----SLQSPSEL---

---------------LYA-----RKLKA---N----------------------------

------------------------------------------------------------

------------------------------------------------------------

------------------------------------------------------------

--------------------------------------

>LLGY36

------------------------------------------------------------

---------------------SY------------VWWPGMDK----------DLEKM--

-----VQR-CDT-CQ--LH------NK-S-PPAAPL-HPW---------------EWPEK

PWTRIHIDY-----AGPF---------------LGK----MLLVAVD----ATS------

--KWIE-AH-------IMS----ST-T-ST------ATV-NN--L-REIFAQHG------

--------------LPEVLVSDN-A-----------------------------------

------------------------------------------------------------

------------------------------------------------------------

------------------------------------------------------------

------------------------------------------------------------

------------------------------------------------------------

------------------------------------------------------------

--------------------------------------

>LLGY37

------------------------------------------------------------

---------------------QS------------VWWPGLSK----------QIEDL--

-----VES-CDR-CA--KE------RV-N--QAEPM-IPS---------------DVPER

PWQNVGSDL-----F-EL---------------NGS----PYLLVVD----YVS------

--AFVE-IS-------KLS----ST-T-SA------SIV-NH--M-TLMFARHG------

--------------VPEVVVTDN-GPQYASD---------TFRRF-VQ------------

-----------RG-----------------------------------------------

------------------------------------------------------------

------------------------------------------------------------

------------------------------------------------------------

------------------------------------------------------------

------------------------------------------------------------

--------------------------------------

>LLGY38

------------------------------------------------------------

------------------------------------------------------------

--------------------------------------------------------IASY

PIQIIGADL-----IGPL----VESPT------GNR----YILTIID----FCT------

--GWAE-AF-------PLP----NK-T-NE------SVW-NA--FANGFICRHG------

--------------VPEVIITDN-AKEFTAF---------EFERYLSQ------------

-----------IGI--EH---RTTTPVHPQSNGKI--ERFNKSIKELIQKAV-N----NV

P--------SRWESVLNDALLAYRASV---------------STT----TGYTPYFL---

---------------MTG------------------------------------------

------------------------------------------------------------

------------------------------------------------------------

------------------------------------------------------------

--------------------------------------

>LLGY39

---------------------------------------------------HAS-----H

V-GI--------EGCLR-RAREC------------FYWPHMSQ----------DMKKF--

-----IST-CDV-CL--AH------QA-S-QQRELL-KQH---------------EEVMR

PWAKLGVDL-----C-SL---------------HGG----TLLVVCD----YFS------

--NYLE-VE-------HITG---NV-T-SR------SVI-KV--F-SSLFARHG------

--------------IPDVVVSDN-GSQFASA---------EFASLANK------------

-----------WCF--QH---VTSSPHYAQSNGKA--DNAVKTVKRLFT-----------

---KC----KEDGKSEFLTLLDWRNTP---------------SEG----MGTSPSQR---

---------------LMG-----RRCKT---LL---------------------------

------------------PMAA--------------------------PLLQ--------

-----P----------------------RHS------------------AV-------SD

S--P------------------------------------AP-LAAK-------------

AKQ-----------------------------------

>LLGY40

---------------------------------------------------HEG-----H

Q-GI--------TKCRE-RAKQS------------VWWPGLSK----------QIEDL--

-----VES-CDR-CA--KE------RV-N--QAEPM-IPS---------------DVPER

PWQKVGSDL-----F-EL---------------NGS----PYLLVVD----YLS------

--AFVE-IS-------KLS----ST-T-SA------SIV-NH--M-TSMFARHG------

--------------VPEVVVTDN-GPQYASD---------TFRRFAAA------------

-----------RDF--LH---TTSSPRFPQSNGEA--ERALKTLKCLLA-----------

---KS--------DNLYDTLLAYRSTP---------------LS-----NGYSPAEL---

---------------LMG-----RKLRT---PI---------------------------

------------------PTIP--------------------------ALLE--------

-----P------------------------------------------------------

------------------------------------------------------------

--------------------------------------

>LLGY41

---------------------------------------------------HLA-----H

P-GI--------NIMKG-LARSY------------VWWPGMDK----------DLEKM--

-----V-R-CDT-CQ--LH------NK-S-PPAAPL-HPW---------------EWPEK

PWTRIHIDY-----AGPF---------------LGK----MLLVAVD----ATS------

--KWIE-TH-------IMT----ST-T-ST------ATV-NK--L-REIFAQHG------

--------------LAEVLVS-------------------EFETFMRK------------

-----------YGI--VH---VTSAPYHPASNGLG--ERAVQTVKSGIIK----------

--TAG----DNMDVKLQRFFFDYRRTP---------------QST----TDKSPMEI---

---------------LNN-----RNMRSRLDFLH--------------------------

------------------PSLQGK------------------------------------

-------------------------------IH-KKN-----------------------

------------------------------------------------------------

--------------------------------------

>LLGY42

------------------------------------------------------------

--------------RQC-NLR---------------------------------------

---------CVP-CQ--AA------TP-R-TAYEPL-NMT---------------PLLEA

LWRDLNTDF-----YGPP--------A------TGE----YLLVIID----ECS------

--RYPV-VE-------IVR----ST-S-AN------TVI-PV--F-DKVMSVFG------

--------------IPTK------------------------------------------

------------------------------------------------------------

------------------------------------------------------------

------------------------------------------------------------

------------------------------------------------------------

------------------------------------------------------------

------------------------------------------------------------

--------------------------------------

>LLGY43

------------------------------------------------------------

------------------------------------------------------------

------------------------------------------------------------

-------YL-----F-DV---------------KGR----TFIILSD----YYS------

--KYPI-VN-------ELQA---PV-T-SA------ADT-NV--I-EDAFAMYG------

--------------RPDQIRSDN-GPQYAGQ---------HFRNF-RR------------

-----------WGI--QH---VTSSLHYAQSNGFS--ERQVRWIKSIIK-----------

---NA-------------------------------------------------------

------------------------------------------------------------

------------------------------------------------------------

------------------------------------------------------------

------------------------------------------------------------

--------------------------------------

>LLGY44

---------------------------------------------------HES-----H

Q-GA--------DKCKT-RGRTV------------LYWPGISQ----------DIETI--

-----VGR-CHI-CL--KF------RA-S-NPKEPL-IPH---------------DVPER

PRQKVAADI-----M-TL---------------KSR----DYIAAVD----CYS------

--KYPE-IA-------LLE----NK-T-AS------NVI-IH--L-KSIFARHG------

--------------IPEEMMSDN-MP-FASQ---------EFTNFGRD------------

-----------WGI--KL---TTSSPNFPQSNGQS--ERAVQTLKRILK-----------

---KA----DCEGRDPYVALLEYRNTP---------------VAG----ALFSPAQM---

---------------LMS-----RMLRA---KL---------------------------

------------------PARS--------------------------RLLT--------

-----PQVVS-----------------AQCQLQ-QRQDKYK---QYYDRGS-------KR

L--TEL-------------Q-----PGSVV--------RVRH------------------

ENVWEPAVVMRKEGH-----------------------

>LLGY45

---------------------------------------------------HEA-----H

Q-GI--------EKTRL-RARSC------------VYWKAINR----------DIDDI--

-----VRK-CDT-CQ--QL------QR-R-QAHEPL-MQH---------------ELPTR

PWQIVGTDL-----F-VI---------------RLD----TYLLMCD----YYS------

--KFPF-VY-------RIEG---RV-T-SD------AII-SK--M-SEVFAENG------

--------------SPDKVVSDN-GGHYSSQ---------AFRNFANE------------

-----------WYF--DH---VTSSPHFPQSNGFI--ERQVQTVKSTLK-----------

---KA----AMTR-----------------------------------------------

------------------------------------------------------------

------------------------------------------------------------

------------------------------------------------------------

------------------------------------------------------------

--------------------------------------

>LLGY47

------------------------------------------------------------

---------------------------------------RIDA----------EIEPK--

-----VRS-CGV-YQ--EN------SK-L-PANSNL-HPW---------------QWPGK

AWHRVHIDY-----AAPF---------------EGK----MILVIMD----AHS------

--KYID-S----------------------------------------------------

------------------------------------------------------------

------------------------------------------------------------

------------------------------------------------------------

------------------------------------------------------------

------------------------------------------------------------

------------------------------------------------------------

------------------------------------------------------------

--------------------------------------

>LLGY48

---------------------------------------------------HDGS---GH

Q-GR--------DRTLA-LVRKR------------AFWPGMSK----------DVNSY--

-----CAS-CSR-CCTAKA-------L-R-PTVKPS-MGH---------------LLADK

PLQLVAIDF-----T-LL----EKASD------GRE----NVLTMTD----AFS------

--KFTV-AI-------PTK----DQ-K-AS------TVA-KV--LTQDWFYRYG------

--------------VPRRIHSDQ-GRNFESD---------VVKALCSV------------

-----------YGI--KK---SRTTTYHPEGNGQC--ERFNRTMHDLLRSLP-P----ER

K--------RQWPRYLQELVFAYNSTP---------------HAA----TGFSPYFL---

---------------MFG-----QEPRL--------------------------------

------------------PVDELLD-----------------------------------

------------------------------------------------------------

------------------------------------------------------------

--------------------------------------

>LLGY49

------------------------------------------------------------

------------------------------------YWPRMTT----------QVKDY--

-----ISK-CDI-CL--SH------RS-A-PPREPL-QQH---------------DFVAR

PWSKIGADL-----C-QL---------------HGR----TLLVVCD----YYS------

--NFFE-VA-------RLN----TV-T-TR------SVV-RE--F-LPMFARFG------

--------------LPDVLVTDN-GPQFASA---------EFAVFVRK------------

-----------KGI--TH---LTSSPHYAQSNGKS--ENAVKTLKLIFA-----------

---KA----KQSGESEYMALLDWRNTP---------------SEG----MGTSPAQR---

---------------LMG-----RRCKT---LL---------------------------

------------------------------------------------------------

------------------------------------------------------------

------------------------------------------------------------

--------------------------------------

>LLGY50

------------------------------------------------------------

------------------------------------------------------------

------------------------------------------------------------

------------------------------------------------------------

-------VE-------IVS----ST-S-AD------VVI-PC--I-EKVFSEYG------

--------------IPDTLRTDN-GPPFNSR---------DFTQFAEQ------------

-----------LGF--RH---RKITPYLPRANAEV--ERFMRTVKKIIKTSI--------

--YER----RDWKTEMYRFLRNYRATP---------------HST----TGFPPATI---

---------------LFA-----RSMKVKL------------------------------

------------------PELHT-------------------------------------

------------------------------------------------------------

------------------------------------------------------------

--------------------------------------

>LLGY51

---------------------------------------------------HEG-----H

Q-GI--------TKCRE-RAKQS------------VWWPGLSK----------QIEDL--

-----VES-CDR-CA--KE------RV-N--QAEPM-IPS---------------DVPER

PWQKVGSDL-----F-EL---------------NGS----PYLLVVD----YLS------

--AFVE-IS-------KLS----ST-T-SA------SIV-NH--M-TSMFARHG------

--------------VPEVVVTDN-GPQYASD---------TFRRFAAA------------

-----------RGF--LH---TTSSPRFPQSNGEA--ERAVKTLKCLLA-----------

---KS--------DNPYDTLLAYRSTP---------------LS-----NGYSPAEL---

---------------LMG-----RKLRT---PI---------------------------

------------------PTIP--------------------------ALLE--------

-----PQWSH--------------LRGARKSRL-EIKNRQK---KSFDKRH---------

------------------------------------------------------------

--------------------------------------

>LLGY52

---------------------------------------------------HGLS----H

P-SI--------RTTKK-MVAAK------------FVWPGLQK----------QVGIW--

-----AKA-CLR-CQAAKV-------H-R-HTTAPL-DQF---------------TPATR

RFDHIHVDI-----VGPL----PPS-H------NYG----YLLTVVD----RFT------

--RWPE-AI-------PLV----DA-Q-TI------TCA-KA--FAFHWIARFG------

--------------VPVELTSDR-GSQFTSE---------LWAILSQL------------

-----------HGT--RL---HRTTAYHPQSNGIV--ERSHRHLKSALIARL--------

---NG----PNWIDELPWVLLGIRTVP---------------KED----LDCSSAEM---

---------------VYG-----APLTV--------------------------------

------------------PGDFLPRGQETQE-----------AA-QFLPRLR--------

-----ETV-----------------RGLAPRPP-VPHG--------TRP--------SSV

P--GTL-------------A-----NSSYV--------FVRR-DSHR----------PPL

TPPYEGPYKVLT---HGDKTFLLDYG------------

>LLGY53

---------------------------------------------------HAM-----H

P-GV--------VRMKA-IARSF------------VWWPGIDS----------DIEEM--

-----VRS-CPE-CS--KQ------RN-A-PTAAPL-MPW---------------PWATR

PWQRVHVDF-----A-EK---------------NGK----MFLVVAD----SHS------

--KWLE-VL-------LMN----ST-T-AG------STI-TE--L-RKLFSAYG------

--------------LPESVVSDN-GPQFTAE---------EFETFLKL------------

-----------NGV--KH---VLCPPYHPASNGLA--ERNVQTFKNMLAK----------

-ADPR----IPLQHRLSDILFQYRNTP---------------HSI----TGLTPAEL---

---------------FLK-----RAPRTRLTLLK--------------------------

------------------PSLQTK------------------------------------

-------------------------------VH-ERQQKEK---QHHDGPR------PVR

L--SQF-------------D-----MYQPV--------RIRN-MRGG-------------

KEKWIPGTVTKI--LGPLTYIVRLP-------------

>LLGY54

------------------------------------------------------------

------------------------------------------------------------

-------------------------------------------------------PFGSY

PMEFVAADL-----IGPF----TESHN------GNK----YILTIID----FCT------

--GWAE-AI-------PIP----TK-S-NQ------AVW-DA--FSNGFICRHG------

--------------VCRVLLTDH-GAEFTAL---------AFERYLSQ------------

-----------IGI--EH---RMSTPAHPQSNGKI--ERFNRTLKQMIQKAV-N----NQ

P--------SRWEEVLNDVLLAYRASV---------------STT----TGYTPHFL---

---------------MTG------------------------------------------

------------------------------------------------------------

------------------------------------------------------------

------------------------------------------------------------

--------------------------------------

>LLGY55

---------------------------------------------------HEG-----H

Q-GI--------TKCRE-RAKQS------------VWWPGLSK----------QIEDL--

-----VES-CDR-CA--KE------RV-N--QAEPM-IPS---------------DVPER

PWQKVGSDL-----F-EL---------------NGS----PYLLVVD----YLS------

--AFVE-IS-------KLS----ST-T-SA------SIV-NH--M-TSMFARHG------

--------------VPEVVVTDN-GPQYASD---------TFRRFAAA------------

-----------RGF--LH---TTSSPRFPQSNGEA--ERAVKTLKCLLA-----------

---KS--------DNPYDTLLAYRSTP---------------LS-----NGYSPAEL---

---------------LMG-----RKLRT---PI---------------------------

------------------PTIP--------------------------ALLE--------

-----PQWSH--------------LRGARKSRL-EIKNRQK---KSFDKR----------

------------------------------------------------------------

--------------------------------------

>LLGY56

------------------------------------------------------------

------------------------------------------------------------

----------KS-CQ--LN------QH-A-PAKAPL-HPW---------------EYTAN

PWSRLHVDF-----AGPF---------------LGH----MFLVIVD----SHT------

--KWLE-VF-------QMQ----KI-T-SR------KTV-ER--L-RSCFATHG------

--------------MPDCIVSDN-GPTFTSE---------EFREFTSA------------

-----------NGI--RH---IFTAPYHPSSNGLA--ERAVESFKEGMKR---------M

Q-----------PAPLQLCFANYRLTP---------------HST----TNRSPTEM---

---------------LLR-----RHPKSRLDLIR--------------------------

------------------PSTKSR------------------------------------

-------------------------------VK-GKQMKQK---QQHDQ--------HAK

D--HTF-------------N-----PGDRV--------LVRN-F-AP-------------

GPTWLPGELLQR--TGPVSFTAQLS-------------

>LLGY57

---------------------------------------------------HFG-----H

I-GV--------VKMKG-LARSY------------VWWPGIDN----------DIESL--

-----ARK-WQ--CQ--RV------QF-E-APTVPL-YPW---------------EWPVK

TWQRIHVDY-----AGPF---------------MGW----MFLIAVD----AHS------

--KWPE-VL-------PTT----SA-S-AE------KTV-EL--L-RDVFARYG------

--------------LPEHLHSDN-GSQFTSE---------VFRNSKKA------------

-----------NNI--RH---TFSAPHHPATNGQV--ERFVQTFKQAIRSPR--------

--GDS----GAVKRHLTKCMFAYRNAP---------------HAT----TGDSPAML---

---------------PMG-----RGLRTRLDVKR--------------------------

------------------PNTRKT------------------------------------

-------------------------------VE-IIRPRQM-----RRGE-------GNT

V--LVL-------------E-----VGDRI--------AVRN-Y-RN-------------

VQKWVPGTTKEK--RGTRSYEVLV--------------

>LLGY58

---------------------------------------------------HAA-----H

Q-GV--------TSMTA-RANVS------------VFWPGITT----------DIARL--

-----RNS-CMD-CN--RI------SP-S-QPNAPP-TTP---------------VDPEF

PFQCICADY-----F-TY---------------KGA----HYLIIVD----RYS------

--NWPI-IK-------KTS-----G-G-AA------GLV-KS--L-REEFITYG------

--------------IAEELASDG-GPEFVAT---------ETQEFLKS------------

-----------WGV--RH---RLSSVAYPHSNCRA--EIGVKSCKRLLMH--------NT

G-PNG----ELDTPSFQRAMLQYRNTP---------------DQD----TKMSPAMI---

---------------VFG-----RSIRD---FI---------------------------

------------------PVL---------------------------------------

----------------------------------------P---GRYT------------

------------------------------------------------------------

--------------------------------------

>LLGY59

---------------------------------------------------HLA-----H

P-GI--------NRMKG-LARSY------------VWWPGMDK----------DLEKM--

-----VQR-CDT-CQ--LH------NK-S-PPAAPL-HPW---------------EWPEK

PWTRIHIDY-----AGPF---------------LG-----MLLVAVD----ATS------

--KWIE-TH-------IMS----ST-T-ST------ATV-NK--L-REIFAQHG------

--------------LPEVLVSDN-AANFISE---------EFEIFNRK------------

-----------NGI--IH---VTSAPYHPASNGLG--ERAVHTVKSGIIK----------

--TAG----DNMEVKLQRFLFDYRRTP---------------QST----TIKSPMEI---

---------------LNN-----RKMRSRLDLLH--------------------------

------------------PSLQGK------------------------------------

-------------------------------IH-KKQKLMK---ETNDR--------RAH

E--RHF-------------E-----AGDSV--------YIKN-F-GP-------------

GLKWLVGTVGYV--TGPVSYTVVLG-------------

>LLGY60

---------------------------------------------------HGLS----H

P-SI--------RTTRK-MIAEK------------FVWRGLNK----------QVGAW--

-----AKS-CLR-CQAAKV-------H-R-HTASPV-ADF---------------APTTR

RFDHVHVDL-----VGPL----PPS-Q------NHR----YLFTVVD----RFT------

--RWAE-AI-------PLV----DA-Q-TT------TCA-RA--FAAHWVARFG------

--------------VPADMTSDR-GSQFTSE---------LWSVLSQL------------

-----------HGT--RL---HRTSAYHPQSNGLV--ERFHRHLKSALMARL--------

---DG----PNWLDELPWVLLGIRTAP---------------KED----LGCSSAEL---

---------------VYG-----APLTV--------------------------------

------------------PGDFIPRGQETQE-----------AA-RFLPRLR--------

-----ERV-----------------RDLAPRPS-IPHG--------TRP--------SSV

P--STL-------------A-----HSAYV--------FVRR-DSHR----------PPL

TPPYEGPYKVLT---HGEKSFVLDYG------------

>LLGY61

------------------------------------------------------------

-----------------------------------IFWPGMSH----------DVRQM--

-----ADN-CVS-CQ--EL------KP-A-NRRETL-LQH---------------EVGNV

PWEKIGCDL-----F-EI---------------SGR----NYLIVID----YFS------

--NFIE-VD-------LMT----TT-T-ST------QVV-SS--L-KKMCARFG------

--------------TPRQIVSDG-GPQFTSR---------EFEVFVKS------------

-----------WGI--DH---VTSSPNHQQANGKA--ESAVKLVKAMME-----------

---KC----VKTGSDQYLALLELRNTP---------------RQD----TNASPAQM---

---------------MFS-----RKLLS---VL---------------------------

------------------PAVSNS----------------------SKSCYD--------

-----P---------------------AKRAKR-QKSVK-----KYYDKRA-------HN

L--PQL-------------E-----PNQSV--------FFRK-LEN--------------

-DHWKKGRVVAK--HSDRAYIVNGE-------------

>LLGY62

------------------------------------------------------------

------------------RLREK------------VWWPRMDK----------DVEVF--

-----VRV-CYP-CQ--IV------GG-R-PSPEPI-RST---------------PLPQG

PWDEIAIDL-----CGPL--------P------NGE----SLLVVID----YFS------

--RWPE-VV-------WMR----NT-N-AQ------NII-KC--L-ETMFTTHG------

--------------LPYKVRSDN-GPQFVAA---------EFEGFLEY------------

-----------LRI--QH---TKGIPYWPQSNVLT-------------------------

------------------------------------------------------------

------------------------------------------------------------

------------------------------------------------------------

------------------------------------------------------------

------------------------------------------------------------

--------------------------------------

>LLGY64

---------------------------------------------------HAC-----H

P-GM--------TKMKG-LARAT------------LGWPKLDQ----------AIEDK--

-----VKS-CHM-CQ--FN------QN-A-PVKAPL-HPW---------------EWPER

PWSRIHIDH-----AGPY---------------HNQ----LWLIIVD----AHS------

--KWLD-IY-------PVS----ST-S-SQ------TTI-DM--L-RVSFSNHG------

--------------LPEMIVSDN-ATTFTSE---------QFAEFCEK------------

-----------NGI--HH---VTAARYHPASNGLA--ERAVQTFKSGFDK---------M

G---E----GSLKTKLARFLLQYRNAP---------------QGT----TGQSPAEL---

---------------LMG-----RRLRSHLDLLH--------------------------

------------------PSLSQR------------------------------------

-------------------------------VQ-RRQRYQK----QHDQ--------HAH

E--RSI-------------E-----ICDRV--------YSRN-F-SG-------------

KSDWLSGIVTEK--SGPVSYRVKLD-------------

>LLGY65

---------------------------------------------------HDKM---GH

Q-GG--------DRTSS-LAWHR------------CYWPGMHR----------EIDDY--

-----VKN-CAR-CTLAKM-------P-R-QKTHAP-MGH---------------LLASR

PLEVLAVDY-----T-QL----EKAAD------GRE----SVLVLTD----VFT------

--KFAW-AV-------PAR----DQ-K-AN------TTA-RL--LVREWFQRYG------

--------------VPQRIHSDR-GRNFESA---------TIGELCKL------------

-----------YGI--EK---SRTTAYHPEGNGQC--ERFNRTLHDLLRTLP-P----AQ

K--------RRWTEHLPELCNAYNATP---------------HAS----TGYSPHYL---

---------------LFG-----RDPWL--------------------------------

------------------PIDAFLG-----------------------------------

------------------------------------------------------------

------------------------------------------------------------

--------------------------------------

>LLGY66

------------------------------------------------------------

------------------------------------------------------------

------------------------------------------------------------

-----------------------------------H----DYLVVVD----YFS------

--KFPE-VE-------QLT----CK-T-AN------GVI-SV--L-KQIFARHG------

--------------IPETMICDN-MP-FLSH---------VMADFVTE------------

-----------I-F--EI---VTSSPRYAQSNGQS--KKFVGIVKSYMR-----------

---KA----HEEGRDFWMSLLEYRNTP---------------IT-----APYSTAQL---

---------------LMS-----RKLRD---KM---------------------------

------------------S-----------------------------------------

------------------------------------------------------------

------------------------------------------------------------

--------------------------------------

>LLGY67

------------------------------------------------------------

-------------RTKR-RARN-------------VYWPGISN----------DIATT--

-----VAS-CNK-CQ--EH--R------S-QQREPL-RSE---------------PLPKR

VFEDASADF-----F-HY---------------AGR----DFLVYMD----RLS------

--GWPV-VF-------HFPKG--TT-T-SR------NTI-YA--C-RRAFVELG------

--------------VPVRFRSDG-GPQFASR---------EFNQFLKR------------

-----------WGV--SA---APSTPHYHQSNGHV--EAAVK------------------

--------GDLDGENFQRGLLEYRNTP---------------RAG-----DVSPAQI---

---------------IFG-----HPLRS---VV---------------------------

------------------P-----------------------------------------

------------------------------------------------------------

------------------------------------------------------------

--------------------------------------

>LLGY68

---------------------------------------------------HTA-----H

L-GK--------EKTKL-LARDT------------VYWLNINK----------DIDRL--

-----VQT-CNV-CQ--EH------QS-S-QVPEPL-LQH---------------DIPYK

PWSVLGTDL-----F-EF---------------EGH----QWLIIAD----YYT------

--KYPI-IR-------QLPN---PS-P-SS------VVV-NA--T-KQIFAEFG------

--------------IPDRIVSDN-GPHFASE---------AYREFARM------------

-----------WQF--DH---ITTSPRRPQGNGFI--ERQVRTIKSLLK-----------

---KS----KQSGTDYQLALLHWRTTP---------------INA----NLASPAQL---

---------------IMG-----RRLKS---T----------------------------

------------------------------------------------------------

------------------------------------------------------------

------------------------------------------------------------

--------------------------------------

>LLGY69

----------------------------------------STMKDEVLQELHDKM---GH

Q-GI--------DRVEK-LVRSR------------FYWPNIRS----------DIQHW--

-----ISM-CER-CNLAKM-------P-H-LKVRTP-MHS---------------IVARE

PLEVIAIDF-----T-VL----EPASN------GME----NVLVMTD----VYS------

--KFTI-AV-------PTR----NQ-T-AQ------TVA-KA--LVREWFFRYG------

--------------VPCRIHSDQ-GRCFDAK---------IVTELYKI------------

-----------YAI--QK---SRTTPYHPMGNGQC--ERYNRTMHALLRTLT-P----TQ

K--------SKWPEHLPELTYAYNVTP---------------HAA----TGFSPFYL---

---------------MFS-----RVPRL--------------------------------

------------------PVDI--------------------------------------

------------------------------------------------------------

------------------------------------------------------------

--------------------------------------

>LLGY70

---------------------------------------------------HAM-----H

P-GV--------VRMKA-IARSF------------VWWPGIDS----------DIEEM--

-----VRS-CPE-CS--KQ------RN-A-PTAAPL-MPW---------------PWATR

PWQRVHVDF-----A-EK---------------NGK----MFLVVAD----SHS------

--KWLE-VL-------LMN----ST-M-AG------STI-TE--L-RKLFSAYG------

--------------LPESVVSDN-GPQFTAE---------EFETFLKL------------

-----------NGV--KH---VLCPPYHPASNGLA--ERNVQTFKNMLAK----------

-ADPR----IPLQHRLSDILFQYRNTP---------------HSI----TGLTPAEL---

---------------FLK-----RAPRTRLTLLK--------------------------

------------------PSLQTK------------------------------------

-------------------------------VH-ERQQKEK---QHHDGPR------PVR

L--SQF-------------D-----MYQPV--------RIRN-MRGG-------------

KEKWIPGTVTKI--LGPLTYIVRLP-------------

>LLGY71

------------------------------------------------------------

------------------------------------------------------------

--------------------------------------------------------VPSE

AWKVLGTDL-----F-DV---------------KGR----TFIILSD----YYS------

--KYPI-VK-------ELQA---PV-T-SA------AVT-GV--I-EDACAMFG------

--------------RPDQIRSDN-GPQYAGQ---------HFRNFCRR------------

-----------WGI--QH---VTSSPRYAQSNGFS--ERQVRWIKSIIK-----------

---KM----HQD------------------------------------------------

------------------------------------------------------------

------------------------------------------------------------

------------------------------------------------------------

------------------------------------------------------------

--------------------------------------

>LLGY72

---------------------------------------------------HTA-----H

L-GK--------EKTKL-LARDT------------VYWLNINK----------DIHRL--

-----VQT-CNV-CQ--EH------QS-S-QVPEPL-LQH---------------DIPYK

PWSVLGTDL-----F-EF---------------EGH----QWLIIAD----YYN------

--KYPI-IR-------QLPN---PS-S-SS------VVV-NA--T-KQIFAEFG------

--------------IPDKIVSDN-GPHLASE---------AYREFARM------------

-----------WQF--DH---ITNSPRRPQGNGFI--ERQVRTIKSLLK-----------

---KS----KQSGTDYQLALLQWRTTP---------------INA----NLASPAQL---

---------------IMG-----RRLKS---T----------------------------

------------------------------------------------------------

------------------------------------------------------------

------------------------------------------------------------

--------------------------------------

>LLGY73

------------------------------------------------------------

------------------------------------------------------------

------------------------------------------------------------

------------------------------------------------------------

-------------------S---ST-R-TK------CVI-YA--T-RSQFARHV------

--------------VPEVVMSDN-GPQFSCG---------EFREFAQR------------

-----------WDF--EH---IMGSWRYPQSNGHV--ERAIGTVKNLVK-----------

---KT----TEDGSDVQLALLNFRNTV---------------REG----YG---AQL---

---------------LFG-----RRCRT---LL---------------------------

------------------PI----------------------------------------

------------------------------------------------------------

------------------------------------------------------------

--------------------------------------

>LLGY74

---------------------------------------------------HSA-----H

Q-GV--------SSMES-RARSI------------VFWPGIST----------AIQET--

-----RDR-CRS-CN--KT------AP-S-QAATPP-AAL---------------DTPST

PFESVFADF-----C-DY---------------GGC----HYLVVGD----RLS------

--GWVD-IY-------KTPPGTPYS-G-AT------GLI-AC--L-RQMFATFG------

--------------VPEILSSDG-GPEFTAS---------ETSNFLSR------------

-----------WGV--HH---RISSVAFPQSNGRA--EVAVKKAKRTLMD--------NI

G-PTG----SLDNDGLLRAMLQLRNTP---------------DPD----CNVSPAEV---

---------------IFG-----RPIRDAFSFVNRCTKFENPSIRPMWREAWSAKENAMR

ARFARTSEALNAHSRALPPLVI--------------------------------------

--------------------------GARVYVQ-NQRGPHP---NKWDRSG---------

------------------------------------------------------------

-------------VVVDVG-------------------

>LLGY75

--------------------------------------------------IHAG-----H

Q-GI--------EKCKM-RARTS------------VFWNGINN----------DLEDV--

-----VKR-CAV-CQ--EH------QH-T-NPRETL-LPH---------------ELPTR

SWQILGTDL-----F-HY---------------NNS----EYLIVVD----YYS------

--KFPF-VR-------KMPT---PC-T-SH------AVV-AA--T-ADIFSEHG------

--------------VPEKVVSDN-GPHYDCV---------NYKKFAQE------------

-----------WGF--EH---VTSSPHFPQSNGFV--ERTIQTVKRTLL-----------

---KA----KESNMNPCKAMLCLRTTP---------------LDH----HLPSPSEL---

---------------LYA-----RKLK---------------------------------

------------------------------------------------------------

------------------------------------------------------------

------------------------------------------------------------

--------------------------------------

>LLGY76

--------------------------------------------------LHLA-----H

Q-GV--------EKTRL-RARSC------------VYWININC----------DIENM--

-----IQR-CDI-CQ--RE------LC-A-QPSEPL-MQH---------------EVPSR

PWQVVGTDL-----F-SI---------------GRN----NYLIIGD----YYS------

--KFPF-VE-------LIEG---RA-T-SD------MIV-KL--T-KRIFSEQG------

--------------VPDRVVSDN-GGHFDSQ---------AYKLFAKA------------

-----------WGF--EH---VTSSPHYPRSNGFV--ERQIQTIKRTLK-----------

---KA----ASARVDTDMAMLILRSTP---------------IDH----HLPSPAEM---

---------------LNA-----RKMRA---NL---------------------------

------------------PVKI--------------------------LNAH--------

-----PEKG-----------------AISERLF-ERQRQQK---VYHD------------

------------------------------------------------------------

--------------------------------------

>LLGY77

---------------------------------------------------HES-----H

Q-GA--------DKCKT-LGRTV------------LYWPGMSQ----------DIETI--

-----VGR-CHI-CL--KF------RA-S-NPKEPL-IPH---------------DVPER

PWQKVAADI-----M-TF---------------KSR----DYIVAVD----CYS------

--TYPE-IA-------LLD----NK-T-AS------NVI-IH--L-KYIFARHG------

--------------IPEEMMSDN-MP-FASQ---------EFTNFGRD------------

-----------WGI--KL---TTSSPNFPQSNGQS--ERAVQTLKRILK-----------

---KA----DCEGRDPYVALLEYRNTP---------------VAG----ALFSPAQM---

---------------LMS-----RMLRA---KL---------------------------

------------------PARS--------------------------KLLT--------

-----PQVVS-----------------AQCKLQ-QRHDKYK---QYYDRGS-------KR

------------------------------------------------------------

--------------------------------------

>LLGY79

---------------------------------------------------HDLA----H

A-GA--------RAMRR-LICDR------------FVWHGMAR----------DIRHW--

-----ART-CEA-CQRAKV-------S-K-HVVAPL-TPL---------------PMPVK

RFDSLHVDL-----VGPL----PAS-Q------GFT----YLLTIVD----RFT------

--RWPE-AI-------PLS----DI-S-AI------TCA-RA--FLYHWVSRYG------

--------------VPSTLTSDR-GRQFVSE---------LWRKTASM------------

-----------LGA--AT---NTTTSYHPQSNGLV--ERMHRTMKAALKAKL-A------

---AD----PNWVDALPLVMLGMRAAV---------------KED----LNCSAAEM---

---------------VFG-----EALRL--------------------------------

------------------PGEFFVSADGDWTA----------DP-VFVSDLR--------

-----QRI-----------------RQLRPIAP-DWHGG------QTRR--------NYV

P--REL-------------S-----TATHV--------FVRV-DVHR----------RPL

QAPYQGPFKVVE---RHEKFYKLDLG------------

>LLGY80

------------------------------------------------------------

------------------------------------YWPRMTT----------QVKDY--

-----ISK-CDI-CL--SH------RS-A-PPREPL-QQH---------------DFVAR

PWSKIGADL-----C-QL---------------HGR----TLLVVCD----YYS------

--NFFE-VA-------RLN----TV-T-TR------SVV-RE--F-LPMFARFG------

--------------LPDVLVTDN-GPQFASA---------EFAVFVRK------------

-----------KGI--TH---LTSSPHYAQSNGKS--ENAVKTLKLIFA-----------

---KA----KQSGESEYMALLDWRNTP---------------SEG----MGTSPAQR---

---------------LMG-----RRCKT---LL---------------------------

------------------------------------------------------------

------------------------------------------------------------

------------------------------------------------------------

--------------------------------------

>LLGY81

-----------------------------------------------------------H

D-SILGSHLATKKTYDR-VTSN-------------FFWPGAYD----------DVTRY--

-----CQS-CDI-CQQTVP------KS-R-CGKTPL-VGM---------------PIIGE

TFDRVAIDL-----VGPL----PMSER------KHR----WILTLVN----CAT------

---YPE-A----------K----GI-D-TI------ECA-EA--L-VNIFCRVG------

--------------IPREILSDR-G--------------------CYL------------

-----------F-------------------DSFI-------------------------

------------------------------------------------------------

------------------------------------------------------------

------------------------------------------------------------

------------------------------------------------------------

------------------------------------------------------------

--------------------------------------

>LLGY82

---------------------------------------------------HQS-----H

R-GV--------VRMKT-MARLY------------VWWPNIEA----------SVEAC--

-----CKA-CNV-CA--VT------AP-A-PT-ANL-SPW---------------PLPDE

PWDRIHVDF-----AGPF---------------LGN----MWMLVMD----AYS------

--KWPS-VV-------RMS----KYPT-TE------TTI-MA--L-NILFTTWG------

--------------SPKTLVSDN-GPQFGSK---------QFEDWCRL------------

-----------NGI--VH---LTSAPFHPPSNGEA--ERLVGVFKTAMQR--------SV

G-EEG----KERDKATMGFLREYRSTP---------------NCA----TGRTPAEL---

---------------MIG-----RQVRTPLSLLQ--------------------------

------------------PSVHH-------------------------------------

------------------------------------------------------------

------------------------------------------------------------

--------------------------------------

>LLGY83

--------------------------------------------------LHLA-----H

Q-GV--------EKTRL-RARSC------------VYWININC----------DIENM--

-----IQR-CDI-CQ--RE------LC-A-QPSEPL-MQH---------------EVPSR

PWQVVGTDL-----F-SI---------------GRN----NYLIIGD----YYS------

--KFPF-VE-------LIEG---RA-T-SD------MIV-KL--T-KRIFSEQG------

--------------VPDRVVSDN-GGHFDSQ---------AYKLFAKA------------

-----------WGF--EH---VTSSPHYPRSNGFV--ERQIQTIKRTLK-----------

---KA----ASARVDTDMAMLILRSTP---------------IDH----HLPSPAEM---

---------------LNA-----RKMRA---NL---------------------------

------------------PVKI--------------------------LNAH--------

-----PEKG-----------------AISERLF-ERQRQQK---VYHD------------

------------------------------------------------------------

--------------------------------------

>LLGY84

--------------------------------------------------LHLA-----H

Q-GV--------EKTRL-RARSC------------VYWININC----------DIENM--

-----IQR-CDI-CQ--RE------LC-A-QPSEPL-MQH---------------EVPSR

PWQVVGTDL-----F-SI---------------GRN----NYLIIGD----YYS------

--KFPF-VE-------LIEG---RA-T-SD------MIV-KL--T-KRIFSEQG------

--------------VPDRVVSDN-GGHFDSQ---------AYKLFAKA------------

-----------WGF--EH---VTSSPHYPRSNGFV--ERQIQTIKRTLK-----------

---KA----ASARVDTDMAMLILRSTP---------------IDH----HLPSPAEM---

---------------LNA-----RKMRA---NL---------------------------

------------------PVKI--------------------------LNAH--------

-----PEKG-----------------AISERLF-ERQRQQK---VYHD------------

------------------------------------------------------------

--------------------------------------

>LLGY85

---------------------------------------------------HAA-----H

Q-GV--------TSMTA-RANVS------------VFWPGITT----------DIARL--

-----RNS-CMD-CN--RI------SP-S-QPNAPP-TTP---------------VDPEF

PFQCICADY-----F-TY---------------KGA----HYLIIVD----RYS------

--NWPI-IK-------KTS-----G-G-AA------GLV-KS--L-REEFITYG------

--------------IAEELASDG-GPEFVAT---------ETQEFLKS------------

-----------WGV--RH---RLSSVAYPHSNCRA--EIGVKSCKRLLMH--------NT

G-PNG----ELDTPSFQRAMLQYRNTP---------------DQD----TKMSPAMI---

---------------VFG-----RSIRD---FI---------------------------

------------------PVL---------------------------------------

----------------------------------------P---GRYT------------

------------------------------------------------------------

--------------------------------------

>LLGY86

---------------------------------------------------HNA-----H

S-GI--------VRMKA-VGRSL------------MWWPGIDQ----------DIEKT--

-----AKT-CDI-CM--RS------RP-R-PTEAPL-QPW---------------SFPDR

PWSRLHIDY-----AGPF---------------MGR----MMLVVID----AHS------

--KWID-AH-------LTS----GS-T-SA------ITI-SK--L-RQSFSTHG------

--------------IPEVIVSDN-ATGFVSE---------EFQDFCRH------------

-----------NGI--KH---ITSAPHHPASNGLA--ERAVGIVKEGVKR---------M

H---G----GDLETKLARFLFDYRITP---------------HST----TGIAPAEL---

---------------LMH-----RQLKTRLHLIR--------------------------

------------------PDVGVK------------------------------------

-------------------------------VV-AEQTKQK---AKHDR--------HAK

V--RTF-------------Q-----PGELV--------YALR-YHGN-------------

IASWVPGTIHRQ--TGPVSYTVRLE-------------

>LLGY87

---------------------------------------------------HQG-----H

Q-GI--------VKMKQ-RLRSK------------VWWPRMDN----------DAEKI--

-----CRS-CHG-CQV-VS------QL-S-PP-EPM-QRT---------------EPPTG

PWQDVAIDI-----MGPL--------P------TGE----NLLVIVD----YYS------

--RFFE-VV-------IMQ----ST-T-TE------KII-GA--L-IPIFARYG------

--------------YPFSVKSDK-GSQFQSD---------EFKSFLLE------------

-----------HGI--EH---HTSPPLWPQANGEV--ERQNRTFLKALKVAH--------

--VEG----KGWKGELMKFLLVYRTTP---------------QVS----TGVTPAYL---

---------------IFG-----RELKTKL------------------------------

------------------PELRS-----------------------DKNILD--------

-------------------------ENVRDRDW-NHKLTAK---AYADSRR------GAM

L--NPV-------------L-----PGEQV--------LLKN-TKTS-------------

GK------------------------------------

>LLGY89

------------------------------------------------------------

------------------------------------------------------------

------------------------------------------------------------

------------------------------------------------------------

-------IA-------LLE----NK-T-AS------NVI-------KSIFARHG------

--------------IPEEMMSDN-MP-FASQ---------EFTNFGRV------------

-----------WGI--KL---TMSSPNFPQSNGQS--ERAVQTLKHILK-----------

---KA----DCEGRDPYVALLEYCNTP---------------IGG----ALFSPAQM---

---------------LMS-----RILRA---KL---------------------------

------------------P-----------------------------------------

------------------------------------------------------------

------------------------------------------------------------

--------------------------------------

>LLGY90

------------------------------------------------------------

------------------------------------------------------------

------------------------------------------------------------

-------------------------------------------IQVS----S--------

--------------------------T-TQ------VTI-EQ--L-RGLFATHG------

--------------LPETIVTDN-GTYFTSA---------EFKQFVER------------

-----------NNI--QH---ITSPAYHPSSNGLA--ERAVQLVKRGLVK----------

--LKD----GTMETRLSRYLMTYRVTP---------------HST----TGSSPNEL---

---------------LMG-----RKLRTLLDAVH--------------------------

------------------PSISSK------------------------------------

-------------------------------VF-QHQEKMT---EYYNK--------KSK

E--RCF-------------N-----PGDAI--------YVKN-HTQS-------------

APKWIPAV------------------------------

>LLGY92

------------------------------------------------------------

------------------------------------------------------------

------------------------------------------------------------

------------------------------------------------------------

----------------------------------------------------HG------

------------------------------------------------------------

----------------QK---YRITPLWPQANAQA--ECFNKPLMKALKAAS--------

--ING----LAWRTEMQRMLRAYRSTP---------------HLT----TAFTPHRL---

---------------MFG-----RDPRTKL------------------------------

------------------P-----------------------------------------

------------------------------------------------------------

------------------------------------------------------------

--------------------------------------

>LLGY93

---------------------------------------------------HEG-----H

L-GI--------SKCRA-RANTA------------VWWPGLSK----------EIYEM--

-----VST-CHT-CA--KV------HP-E--PKETL-MSA---------------SFPSR

PWERVGMDL-----F-EL---------------NGK----LYLVIVD----YYS------

--RWVE-FR-------KLT----SL-T-SE------HTI-EV--M-KEVFATHG------

--------------IPDVIMSDN-GPQFSAE---------AFLR----------------

------------------------------------------------------------

------------------------------------------------------------

------------------------------------------------------------

------------------------------------------------------------

------------------------------------------------------------

------------------------------------------------------------

--------------------------------------

>LLGY94

---------------------------------------------------HSS-----H

Q-GI--------EATLR-RARDS------------IYWPGMTN----------DIKQM--

-----IES-CQA-CS--KE------KP-S-QQKETL-RSH---------------DLLSK

PWAKVGIDL-----F-TY---------------ANA----TYLIMVD----YYS------

--DFFE-FT-------KLV----DQ-R-AE------TTI-QA--C-KEQFARYG------

--------------VPQIVQSDG-GPQFISA---------EFQAFANN------------

-----------WEF--KH---SMSSPYHSQSNGKA--ESAVKIVKNFLK-----------

---KS-------------------------------------------------------

------------------------------------------------------------

------------------------------------------------------------

------------------------------------------------------------

------------------------------------------------------------

--------------------------------------

>LLGY95

---------------------------------------------------HSS-----H

M-GI--------DSCLR-RAREC------------MYWPNMST----------DMTDY--

-----ISR-CAT-CR--EL------ET-A-SQRETL-MPH---------------DVPDR

PWAKIGTDL-----F-TC---------------NNK----EYLVTVD----YFS------

--NFFE-VD-------ELP----NT-Q-SK------TVV-AC--L-KRHFARYG------

--------------CPEVLVSDN-GPQYTSS---------EFAAFSLQ------------

-----------WDF--EH---CTSSPGHSQANGKA--ESAVKTAKKLLR-----------

---KT----AMSGGDFSMALLDLRNTP---------------TAG----MSTSPTQR---

---------------MMG-----RRART---LL---------------------------

------------------PTSR--------------------------TLLL--------

-----PSVVD--------------CRRAKHEIR-QQQNKQA---RYFNRTA-------KD

L--PHL-------------E-----EGDTV--------RIQP-FNKW-------------

GKHWRKGTVIKR--LDERSYEVET--------------

>LLGY96

---------------------------------------------------HVG-----H

Q-GV--------VKTKM-LLREK------------VWFPAIDS----------MAERQ--

-----VKS-CLA-CQATIS------TP-M-TP-EPM-IST---------------PIPSA

PWKNLSADF-----LGPL--------P------TGE----LILVVID----DFS------

--RFPE-VE-------IVT----ST-A-AS------SVI-PK--L-DSMFARHG------

--------------IPDVLKSDN-GPPFNGS---------ELTKFAQY------------

-----------LGF--QH---KKITPIWPCANGEA--ERFMAPLMKAIRAAH--------

--VEN----RSWKQELYNFLRQYRATP---------------HCT----TGVSSAEA---

---------------LYG-----RKLN---------------------------------

------------------------------------------------------------

------------------------------------------------------------

------------------------------------------------------------

--------------------------------------

>LLGY97

---------------------------------------------------HGLS----H

P-SI--------RTTKK-MVAAK------------FVWPGLQK----------QVGIW--

-----AKA-CLH-CQAAKV-------H-Q-HTTAPL--DF---------------TPATR

RFDHIHVDI-----AGPL----PPS-Q------NYR----YLLTVVD----RFT------

--RWPE-AI-------PLV----DA-Q-TI------TCT-KA--FAFHWITRFG------

--------------VPVELMSDR-GSQFTSE---------LWAILSQL------------

-----------HGT--HL---HRTIAYHPQSNGIV--ERFHRHLKSALIARL--------

---NG----PNWIDELPWVLLGIRTVP---------------KED----LDCSSAEM---

---------------VYG-----APLTV--------------------------------

------------------PGDCLPRGQETQE-----------AA-QFLPRLR--------

-----ETV-----------------RRLALRPP-IPHS--------TRQ--------SSV

P--ATL-------------A-----NSSYV--------FVRR-DSHR----------PPL

TPPYEGPYKVLT---HGDKTFLLDYG------------

>LLGY98

---------------------------------------------------HNG-----H

Q-GV--------EKTRR-LARDC------------VYWLNINK----------DVERI--

-----CKS-CET-CQ--EH------QD-A-NRRELL-IPH---------------ELPSR

PWQFIASDM-----F-EI---------------DNR----QYLLIMD----RYS------

--KYPL-VD-------EIRT---PV-T-SQ------AVT-DR--L-KRYCALFG------

--------------RPDEIMTDN-GPQYTGQ---------PFKEFVES------------

-----------WGI--NH---VMSSPHYARSNGFI--ERSVRHVKPIVK-----------

---KA----LRNGSDIQLALLNLRATP---------------VDT----NLPSPGEM---

---------------LLG-----RPLVT---LL---------------------------

------------------PS-------------------------------H--------

-----SDPGL---------------FAQRQRLE-ERRDAMK---RHHDQTS------GSD

L--PPL-------------Y-----VGQRV--------HVRS-HVD--------------

-KTW----------------------------------

>LLGY99

---------------------------------------------------HEA-----H

Q-GI--------EKTRL-RARSC------------VYWKAINR----------DIDDI--

-----VRK-CDT-CQ--QL------QR-R-QAHEPL-MQH---------------ELPTR

PWQIVGTDL-----F-VI---------------RLD----TYLLMCD----YYS------

--KFPF-VY-------RIEG---RV-T-SD------AII-SK--M-SEVFAENG------

--------------SPDKVVSDN-GGHYSSQ---------AFRNFANE------------

-----------WCF--DH---VTSSPHFPQSNGFI--ERQVQTVKSTLK-----------

---KA----AMTR-----------------------------------------------

------------------------------------------------------------

------------------------------------------------------------

------------------------------------------------------------

------------------------------------------------------------

--------------------------------------

>LLGY100

---------------------------------------------------HSA-----H

Q-GV--------SSMES-RARSI------------VFWPGIST----------AIQET--

-----RDR-CRS-CN--KT------AP-S-QAATPP-AAL---------------DTPST

PFESVFADF-----C-DY---------------GGC----HYLVVGD----RLS------

--GWVD-IY-------KTPPGTPYS-G-AT------GLI-AC--L-RQMFATFG------

--------------VPEILSSDG-GPEFTAS---------ETSNFLSR------------

-----------WGV--HH---RISSVAFPQSNGRA--EVAVKKAKRTLMD--------NI

G-PTG----SLDNDGLLRAMLQLRNTP---------------DPD----CNVSPAEV---

---------------IFG-----RPIRDAFSFVNRCTKFENPSIRPMWREAWSAKENAMR

ARFARTSEALNAHSRALPPLVI--------------------------------------

--------------------------GARVYVQ-NQRGPHP---NKWDRSG---------

------------------------------------------------------------

-------------VVVDVG-------------------

>LLGY101

A--------------------------------------------------HQG-----H

Q-GM--------VKTKR-LIREK------------VWFPGIDV----------LVEKR--

-----VKR-CMA-CQASTH------LP-E-SSMEPL-KMS---------------KLPEG

PWQHVDIDF-----CGPF--------P------SGD----YLLVAID----EYS------

--RFPE-VE-------ITR----ST-S-AF------STI-PK--L-DKIFSTHG------

--------------IPEVVKSDN-GPPFQSS---------EFKSFAEY------------

-----------TGF--QH---RKITPEWPQANSEV--ERFLRTLEKAIRCAI--------

--LEG----KVWKQEMYRFLRSYRATP---------------HSS----MGVSPATA---

---------------LFN-----RNIKTTL------------------------------

------------------PENKEASKSD--------------------------------

-------------------------RTMREADA-RAKSRMK---QYADKRA------KAK

S--SNL-------------Q-----PGDIV--------IMKQ----------------RR

TNKYSTPY-------QPKAYEVVARQGP---------M

>LLGY103

------------------------------------------------------------

------------------------------------------------------------

------------------------------------------------------------

----------------------------------GK----NYIVVTD----HLS------

--NFFE-VK-------CFI----AV-N-SR------SVI-AC--L-KDIFARHS------

--------------IPDELFSDN-GPQYAST---------EFAEFAKN------------

------------EL--VQ---STSSPHYPKTNGLA--ESSVKIVKK--------------

------------------------------------------------------------

------------------------------------------------------------

------------------------------------------------------------

------------------------------------------------------------

------------------------------------------------------------

--------------------------------------

>LLGY105

------------------------------------------------------------

-------------------AREV------------MFWPGMNH----------EVKDV--

-----ISA-CST-CN--TY------KP-D-QCREPL-LSH---------------DIPSR

PWSRVGVDL-----F-HL---------------HDQ----NYLITED----YFS------

--NFFE-VD-------KLS----VT-S-AA------QVI-TK--L-RIHFARFG------

--------------VPDKVISDN-GPQFACE---------EFKLFAKR------------

-----------WEF--EH---ITSSPRYPQSNGKV--ENAVKTAKQIMR-----------

---KA----LDDKADVYLAFLDYRNTP---------------TEA----MHTSPAQR---

---------------MFA-----RRTRT---LL---------------------------

------------------PML---------------------------------------

------------------------------------------------------------

------------------------------------------------------------

--------------------------------------

>LLGY106

---------------------------------------------------HEG-----H

Q-GI--------TKCRE-RAKQS------------VWWPGLSK----------QIEDL--

-----VES-CDR-CA--KE------RV-N--QAEPM-IPS---------------DVPER

PWQKVGSDL-----F-EL---------------NGS----PYLLVVD----YLS------

--AFVE-IS-------KLS----ST-T-SA------SIV-NH--M-TSMFARHG------

--------------VPEVVVTDN-GPQYASD---------TFRRFAAA------------

-----------RGF--LH---TTSSPRIPQSNGEA--ERAVKTLKCLLA-----------

---KS--------DNPYDTLLVYRSTP---------------LS-----NGYSPAEL---

---------------LMG-----RKLRT---PI---------------------------

------------------PTIP--------------------------ALLE--------

-----PQWSH--------------LRGARKSRL-EIKNRQK---KSFDKRH---------

------------------------------------------------------------

--------------------------------------

>LLGY108

---------------------------------------------------HAC-----H

P-GM--------TKMKG-LARAT------------LWWPKLDQ----------AIEDK--

-----VQS-CHM-CQ--VN------QN-A-PVKAPL-HPW---------------EWPER

PWSRIHIDH-----AGMY---------------HNQ----LWLIIVD----AHS------

--KWL-------------S----ST-S-SQ------TTI-DM--L-RVSFSNHG------

--------------LPEMIVSDN-ATSFTSE---------QFAESCEK------------

-----------NGI--HH---VTSAPYHPASNGLA--ERAVQTFKSGFDK---------M

G---E----GSLKTKLARFLLQYRNAP---------------QGT----TGQSPAEL---

---------------LMG------RLRSHLDLLH--------------------------

------------------PSLSQR------------------------------------

-------------------------------VQ-RRQRYQK---EQHDQ--------HAH

E--GSI-------------E-----IGDRV--------YSRN-F-SG-------------

KPDWLSGIVTEK--SGPVSCLVKLD-------------

>LLGY109

------------------------------------------------------------

---------------------------------------------------SNDIATT--

-----VAS-CNK-YQ--EH------RP-S-QQHESL-RPE---------------PLSKR

VFEDVSADF-----F-HY---------------AGR----EFLVYVD----RLS------

--GWPV-VF-------HFPKG--TT-T-SR------HTI-YA--C-RRAFVELG------

--------------VPVWFRSDG-GPQFASR---------QFNQFLKR------------

-----------WGD--SA---ATSTPHYHQSNGHA--EAAVKAMKKLIATTT--------

--VKG----DLDDENFQRGLLEYRNTP---------------RAG-----GLSPAQI---

---------------LFG-----HPLRS---VV---------------------------

------------------P-----------------------------------------

----------------------------------AHRQSFA-------------------

---SKL-------------QK----TADEQ------------------------------

--------------------------------------

>LLGY110

-----------------------------------------------------------H

Q-GI--------EATLR-RARDR------------IYWPGMTN----------DIKQM--

-----IES-CQA-CS--KQ------KP-S-QQKETL-RSH---------------DLLSK

PWAKVGIDL-----F-TY---------------ANA----TYLIMVD----YYS------

--DFFE-FT-------KLV----DQ-R-AE------TTI-QA--C-KEQFARYG------

--------------VPQIVQSDG-RPQFISA---------EFQAFANN------------

-----------WEF--KH---SMSSPYHSQSNGKA--DRQ--------------------

------------------------------------------------------------

------------------------------------------------------------

------------------------------------------------------------

------------------------------------------------------------

------------------------------------------------------------

--------------------------------------

>LLGY111

-------------------------------------------------TVHDLM---GH

Q-GG--------DRTIQ-LAWSR------------CYWPGMHR----------EIDTY--

-----VKH-CSR-CTVAKM-------P-R-RKIHAP-MGN---------------LLASR

PLEVVAIDF-----T-QL----EKAAD------GRE----SVLVMTD----VFT------

--KYTW-AV-------PTR----DH-T-AR------TTA-RV--LVREWFQRFG------

--------------VPQRIHSDR-GRNFESS---------TIRELCKL------------

-----------YGV--QK---SRTTAYHPEGNGQC--ERFNRTLHDLLRTLS-P----SQ

K--------RKWTEHLPELCTAYNATP---------------HAS----TGYSPFYL---

---------------LFG-----RDPRL--------------------------------

------------------PIDAL-------------------------------------

------------------------------------------------------------

------------------------------------------------------------

--------------------------------------

>LLGY112

---------------------------------------------------HSA-----H

Q-GI--------SAMTA-RAESS------------VFWPGITP----------AIAAV--

-----RTN-CSD-CN--RM------AP-S-QPSAPP-TPP---------------VLPVY

PFQCVCSDF-----F-TY---------------KGN----SYLVIVD----RYS------

--NWPI-IE-------RTT-----G-G-AD------GLI-DS--L-RRSFVTYG------

--------------IPDELASDG-GPEFTST---------TTRLFLKT------------

-----------WGV--HH---RLSSVAFPHSNCRA--EIGVKTVKRLITN--------NT

G-TNG----ELDTDGVQRAILQYRNTP---------------DPD----TKLSPAMC---

---------------VFG-----RPIKD---FI---------------------------

------------------PIL---------------------------------------

----------------------------------------P---GRY-------------

------------------------------------------------------------

--------------------------------------

>LLGY113

---------------------------------------------------HNA-----H

S-GI--------VRMKA-VGRSF------------MWWPGIDS----------DIERT--

-----VNS-CDM-CR--RS------RH-K-PTEAPL-QPW---------------SFPDR

PWSRVHIDY-----AGPV---------------MGK----MILVVID----AHS------

--KWIE-AY-------TTS----GS-T-SA------ITI-SK--L-KWIFSSHG------

--------------IPDVIVSDN-ATGFVSE---------EFQSFCRR------------

-----------NGI--KH---VTSAPHHPATNGLA--ERAVGILKGGVQR---------L

Q--------GDLETRIAHFLLDYRITP---------------HTT----TGVSPAEL---

---------------LTG-----RKLRTRLDRII--------------------------

------------------PDVSGR------------------------------------

-------------------------------AI-SKQTTQK---ERHDQ--------HTQ

A--RQY-------------Q-----PGDLV--------YALM-YRGN-------------

KTNWSPGTVVTQ--TGPVSYTVRLE-------------

>LLGY115

---------------------------------------------------HAA-----H

Q-GV--------TSMTA-RANVS------------VFWPGITT----------DIARL--

-----RNS-CMD-CN--RI------SP-S-QPNAPP-TTP---------------VDPEF

PFQCICADY-----F-TY---------------KGA----HYLIIVD----RYS------

--NWPI-IK-------KTS-----G-G-AA------GLV-KS--L-REEFITYG------

--------------IAEELASDG-GPEFVAT---------ETQEFLKS------------

-----------WGV--RH---RLSSVAYPHSNCRA--EIGVKSCKRLLMH--------NT

G-PNG----ELDTPSFQRAMLQYRNTP---------------DQD----TKMSPAMI---

---------------VFG-----RSIRD---FI---------------------------

------------------PVL---------------------------------------

----------------------------------------P---GRYT------------

------------------------------------------------------------

--------------------------------------

>LLGY116

--------------------------------------------------LHLA-----H

Q-GV--------EKTRL-RARSC------------VYWININC----------DIENM--

-----IQR-CDI-CQ--RE------LC-A-QPSEPL-MQH---------------EVPSR

PWQVVGTDL-----F-SI---------------GRN----NYLIIGD----YYS------

--KFPF-VE-------LIEG---RA-T-SD------MIV-KL--T-KRIFSEQG------

--------------VPDRVVSDN-GGHFDSQ---------AYKLFAKA------------

-----------WGF--EH---VTSSPHYPRSNGFV--ERQIQTIKRTLK-----------

---KA----ASARVDTDMAMLILRSTP---------------IDH----HLPSPAEM---

---------------LNA-----RKMRA---NL---------------------------

------------------PVKI--------------------------LNAH--------

-----PEKG-----------------AISERLF-ERQRQQK---VYHD------------

------------------------------------------------------------

--------------------------------------

>LLGY117

---------------------------------------------------HEG-----H

L-GI--------VKTKA-LMRQK------------VWFPSMDK----------LVEAK--

-----VKS-CLA-CQ--IA------TP-V-TSREPL-QMS---------------RLPDQ

PCEEMSVDF-------AH--------V------DGE----TLLILID----DYS------

--RFPF-IE-------PVT----SE-A-AC------AVI-PK--I-DKIFAMFG------

--------------TPDVLKSDN-GPPFNGQ---------DFAKFANV------------

-----------LGF--KH---RKVTPLWPRANGEV--ERFVKTLKKCVKAAK--------

--SDG----KNWRKEMHAFLRNYRTSP---------------HAT----TGVAPSTL---

---------------FLK-----RVVRNKL------------------------------

------------------PQ----------------------------------------

------------------------------------------------------------

------------------------------------------------------------

--------------------------------------

>LLGY118

------------------------------------------------------------

-----------------------------------VWWAGLSK----------EIYKM--

-----VST-CHT-CA--KV------RL-E--PKETL-MSA---------------SFPSR

PWERVGMDL-----F-EL---------------NGK----LYLDIVD----YYS------

--RWVE-FR-------KLT----SL-T-SE------HTI-EV--M-KEVFATHG------

--------------IPDVIMSDN-GPQFSAE---------AFAQFAKS------------

-----------YGF--TH---ITSSPRYPQANGEA--ERAVRTLKEILK-----------

---KN--------DDP--------------------------------------------

------------------------------------------------------------

------------------------------------------------------------

------------------------------------------------------------

------------------------------------------------------------

--------------------------------------

>LLGY119

------------------------------------------------------------

------------------------------------------------------------

------------------------------------------------------------

----------------------------------------HYLVLVD----YLT------

--DFID-FE-------KLL----NL-S-SE------SVI-DI--C-KRSFARFG------

--------------IPDIVQRDN-GPQFTSR---------EFDSFRIK------------

-----------WEF--SH---STSSPYRAQSNGNA--EAAVKTAKSLLK-----------

---RS----T--------------------------------------------------

------------------------------------------------------------

------------------------------------------------------------

------------------------------------------------------------

------------------------------------------------------------

--------------------------------------

>LLGY120

---------------------------------------------------HSA-----H

Q-GV--------SSMES-RARSI------------VFWPGIST----------AIQET--

-----RDR-CRS-CN--KT------AP-S-QAATPP-AAL---------------DTPST

PFESVFADF-----C-DY---------------GGC----HYLVVGD----RLS------

--GWVD-IY-------KTPPGTPYS-G-AT------GLI-AC--L-RQMFATFG------

--------------VPEILSSDG-GPEFTAS---------ETSNFLSR------------

-----------WGV--HH---RISSVAFPQSNGRA--EVAVKKAKRTLMD--------NI

G-PTG----SLDNDGLLRAMLQLRNTP---------------DPD----CNVSPAEV---

---------------IFG-----RPIRDAFSFVNRCTKFENPSIRPMWREAWSAKENAMR

ARFARTSEALNAHSRALPPLVI--------------------------------------

--------------------------GARVYVQ-NQRGPHP---NKWDRSG---------

------------------------------------------------------------

-------------VVVDVG-------------------

>LLGY121

---------------------------------------------------HEA-----H

Q-GI--------EKTRL-RARSC------------VYWKAINR----------DIDDI--

-----VRK-CDT-CQ--QL------QR-R-QAHEPL-MQH---------------ELPTR

PWQIVGTDL-----F-VI---------------RLD----TYLLMCD----YYS------

--KFPF-VY-------RIEG---RV-T-SD------AII-SK--M-SEVFAENG------

--------------SPDKVVSDN-GGHYSSQ---------AFRNFANE------------

-----------WCF--DH---VTSSPHFPQSNGFI--ERQVQTVKSTLK-----------

---KA----AMTR-----------------------------------------------

------------------------------------------------------------

------------------------------------------------------------

------------------------------------------------------------

------------------------------------------------------------

--------------------------------------

>LLGY122

---------------------------------------------------HES-----H

Q-GI--------EKTRL-RARTC------------VYWNGINR----------DIEEV--

-----VRK-CAT-CQ--EM------QR-A-QPHEPL-MPH---------------ETPTC

AWQIVGTDL-----F-MI---------------NRE----SYLIVSD----YYS------

--KFPF-VY-------AIPS---PV-T-SA------AVI-SK--M-KSLFAEQG------

--------------VPQRVVSDN-GGHFSSD---------AFKKFADQ------------

-----------WCF--DH---VTSSPHYPQSNGHI--ERHIQTVKRMLK-----------

---KI----GPRS-DIQMALLVLRATP---------------IDS----HLPSPAEL---

---------------LYG-----RRVVS---NL---------------------------

------------------PV----------------------------------------

------------------------------------------------------------

------------------------------------------------------------

--------------------------------------

>LLGY123

---------------------------------------------------HSS-----H

M-GI--------DSCLR-RAREC------------MYWPNMST----------DMTDY--

-----ISR-CAT-CR--EL------ET-A-SQRETL-MPH---------------DVPDR

PWAKIGTDL-----F-TC---------------NNK----EYLVTVD----YFS------

--NFFE-VD-------ELP----NT-Q-SK------TVV-AC--L-KRHFARYG------

--------------CPEVLVSDN-GPQYTSS---------EFAAFSLQ------------

-----------WDF--EH---CTSSPGHSQANGKA--ESAVKTAKKLLR-----------

---KT----AMSGGDFSMALLDLRNTP---------------TAG----MSTSPTQR---

---------------MMG-----RRART---LL---------------------------

------------------PTSR--------------------------TLLL--------

-----PSVVD--------------CRRAKHEIR-QQQNKQA---RYFNRTA-------KD

L--PHL-------------E-----EGDTV--------RIQP-FNKW-------------

GKHWRKGTVIKR--LDERSYEVET--------------

>LLGY124

---------------------------------------------------HGTS----H

P-SG--------RTTKR-LMNSR------------YVWHGINK----------DITAW--

-----TKT-CLA-CQRAKI-------H-R-HVSAPL-QQF---------------PTPDH

RFDSIHVDI-----VGPL----PSS-Q------GTS----YLFTIVD----RFT------

--RWPE-AI-------PMA----DA-T-AV------SCA-RA--LLENWVPRFG------

--------------VPTDIVSDR-GRQFISG---------LWMELGKL------------

-----------LGM--QL---HHTTAYHAQSNGLV--ERFHRQLKASLKARL--------

---HG----PDWRDELPIVLLGIRCSI---------------KED----LGCTSAEL---

---------------VYG-----TTLRL--------------------------------

------------------PGEFFETTKASTEVTT-------DAL-ALLTRLR--------

-----KTM-----------------RSLRAKSM-THHG--------RQHV-------SRI

P--TAL-------------L-----TCTFV--------FVRK-DAHR----------TPL

ECPYEGPFRVLE---RNDKYYTLDIRG-----------

>LLGY125

------------------------------------------------------------

--------------------------------------PNMAA----------DIRQV--

-----VEK-CEA-CR--SY------ER-S-QQKETL-ITR---------------ETPTL

QWEKVSVDL-----F-SW---------------EGR----DYQVIVD----YTS------

--NFWE-VD-------RMN----ST-T-TT------SVI-KQ--L-KSHFARFG------

--------------IPSVVVSYN-GPQYVSE---------EFCTFAAK------------

-----------WDF--EH---QTSALGHQNANRKA--EAAVKAAKLMVR-----------

---KC----KKLQTDPYLVLLEIRNTP---------------TQG----LGSSPAQR---

---------------LLN-----RRTRT---LL---------------------------

------------------------------------------------------------

------------------------------------------------------------

------------------------------------------------------------

--------------------------------------

>LLGY126

----------------------------------------STMKDEVLQELHDKM---GH

Q-GI--------DRVEK-VVRSR------------FYWPNIRS----------DIQHW--

-----ISM-CER-CNLAKM-------P-H-LKVRTP-MHS---------------IVARE

PLEVIAIDF-----T-VL----EPASN------GME----NVLVMTD----VYS------

--KFTI-AV-------PTR----NQ-T-AQ------TVA-KA--LVREWFFRYG------

--------------VPCRIHSDQ-GRCFDAK---------IVTELYKI------------

-----------YAI--QK---SRTTPYHPMGNGQC--ERYNRTMHALLRTLT-P----TQ

K--------SKWPEHLPELTYAYNVTP---------------HAA----TGFSPFYL---

---------------MFS-----RVPRL--------------------------------

------------------PVDIR-------------------------------------

------------------------------------------------------------

------------------------------------------------------------

--------------------------------------

>LLGY127

---------------------------------------------------HEG-----H

L-GI--------VKTKA-LMRQK------------VWFPSRDK----------LVKAK--

-----VKS-CLA-CQ--IA------TP-V-TSREPL-QMS---------------RLPDQ

PCEEMSVDF-------AH--------V------NRE----TLLVLID----DYS------

--RFPF-IE-------PVT----SE-A-AC------AVI-PK--I-YKIFAMFG------

--------------TPDVLKADN-GPPFNGQ---------DFAKFANV------------

-----------LGF--KH---RKVTPLWPRANGEV--ERFVKTLKKCVKAGK--------

--SDG----KNWRKEMQAFLRNYRTSP---------------HAT----TGVAPSTL---

---------------FLK-----RAVRNKL------------------------------

------------------CNVQ--------------------------------------

------------------------------------------------------------

------------------------------------------------------------

--------------------------------------

>LLGY128

------------------------------------------------ESLHDQM---GH

Q-GI--------ERTQN-LVRKR------------CYWPRMMA----------EVEEW--

-----CNA-CDR-CTLAKM-------P-T-PRIRTS-MSS---------------FLATK

PLEILAIDF-----T-VL----EPASD------GRE----NVLVMTD----VFS------

--KFTV-AI-------PTK----DQ-K-AT------TTA-KA--LVHEWFLRYG------

--------------VPSRIHSDQ-GRNFESE---------IIAGLCKT------------

-----------YGI--RK---SRTTPYRPQGNGQC--ERFNRTLHNLLRTLS-P----EK

K--------RHWPRYLPEVLYAYNATS---------------HSS----TGVSPFYL---

---------------MFG-----RDARL--------------------------------

------------------PIDVLLGVAEEEEQTRVDWVKEHQ------TRLT--------

-----DAY-----------------KKARHRLE-QEANSRK---DFYNR--------KAK

S--APL-------------Q-----VGDRV--------YLRN-RKVK--------GRNKI

Q-------------------------------------

>LLGY129

------------------------------------------------------------

-------------------AREV------------LYWPGMSA----------EVRDY--

-----VSR-CST-CQ--TF------MP-T-QCREPL-QPH---------------ELPSR

PWEKVGGDL-----F-EL---------------AGQ----TFLIMVD----YWS------

--NYFE-IA-------ELH----KK-T-SL------SVI-AQ--F-KVQFARHG------

--------------IPSVVMTDN-GPEFASH---------EFEEFAKT------------

-----------WKF--EH---ITSSPRFPQSNGKA--ENAVKTCKALLM-----------

---KA----RKDRQDPLLALLAWRNTP---------------SEG----FNTSPVQR---

---------------LMG-----RRTRT---L----------------------------

------------------------------------------------------------

------------------------------------------------------------

------------------------------------------------------------

--------------------------------------

>LLGY130

------------------------------------------------------------

------------------------------------------------------------

-------------------------------RREPL-IPH---------------ELPSR

PWQFIASDM-----F-EI---------------DNR----QYLLITD----RYS------

--KYPL-VD-------EIRT---PV-T-SQ------AVT-DL--L-KTYCALFG------

--------------RPDEIMTDN-GPQYTGQ---------PFKEFVES------------

------------GI--NH---VTSSPHYAQSNGFI--ERSVKHVKPIVK-----------

---KA----LRNGSDIQLALLN--------------------------------------

------------------------------------------------------------

------------------------------------------------------------

------------------------------------------------------------

------------------------------------------------------------

--------------------------------------

>LLGY131

---------------------------------------------------HNG-----H

Q-GV--------EKTRR-LARDC------------VYWLNINK----------DVERI--

-----CKS-CET-CQ--EH------QD-A-NRREPL-IPH---------------ELSSR

PWQFIASDM-----F-EI---------------DNR----QYLLITD----RYS------

--KYPL-VD-------EIRT---PV-T-SQ------AVT-DR--L-KKYCALFG------

--------------RPDEIMTDN-GPQYTGQ---------PFKEFVES------------

-----------WGI--NH---VTSSPHYARSNGFI--ERSVRHVKPIVK-----------

---KA----LRNGSDIQLALLNLRATP---------------VNT----NLPSPGEM---

---------------LLG-----RPLVT---LL---------------------------

------------------PS-------------------------------Q--------

-----TDAGL---------------FAQRERLE-ERRDAMK---RHHDQTS------GSD

L--PPL-------------Y-----VGQRV--------RVRS-H----------------

--------------------------------------

>LLGY132

---------------------------------------------------HGLS----H

P-SI--------RTTRK-MIAEK------------FVWRGLNK----------QVGAW--

-----AKS-CLR-CQAAKV-------H-R-HTASPV-ADF---------------APTTR

RFDHVHVDL-----VGPL----PPS-Q------NHR----YLFTVVD----RFT------

--RWAE-AI-------PLV----DA-Q-TT------TCA-RA--FAAHWVARFG------

--------------VPADMTSDR-GSQFTSE---------LWSVLSQL------------

-----------HGT--RL---HRTSAYHPQSNGLV--ERFHRHLKSALMARL--------

---DG----PNWLDELPWVLLGIRTAP---------------KED----LGCSSAEL---

---------------VYG-----APLTV--------------------------------

------------------PGDFIPRGQETQE-----------AA-RFLPRLR--------

-----ERV-----------------RDLAPRPS-IPHG--------TRP--------SSV

P--STL-------------A-----HSAYV--------FVRR-DSHR----------PPL

TPPYEGPYKVLT---HGEKSFVLDYG------------

>LLGY133

------------------------------------------------------------

------------------------------------------------------------

------------------------------------------------------------

----------------------------------------HYLVLVD----YLT------

--DFID-FE-------KLL----NL-S-SE------SVI-DI--C-KRSFARFG------

--------------IPDIVQTDN-GPQFTSR---------EFDSFRIK------------

-----------WEF--SH---STSSSYRAQSNGKA--EAAVKTAKRLLK-----------

---RS----T--------------------------------------------------

------------------------------------------------------------

------------------------------------------------------------

------------------------------------------------------------

------------------------------------------------------------

--------------------------------------

>LLGY134

---------------------------------------------------HEG-----Q

Q-GI--------TKCRE-RAKQS------------VWWPGLSK----------QNEDL--

-----VES-CDR-CA--KG------RV-N--QAEPI-IPS---------------DVPER

PWQKVGSDL-----F-EL---------------NGS----PYFLIVD----YLS------

--AFVE-IS-------KLS----ST-T-SA------SIV-NH--M-TSMFARHG------

--------------VPEVVVTDN-GPQYASD---------TFRRFAAA------------

-----------RGF--LH---TTSSPRFPQSNGEA--ERAVKTLKCLLA-----------

---KS--------DNPYDTLLAYRSTR---------------LS-----NGYRPAEL---

---------------LMG-----RKLRT---PI---------------------------

------------------P-----------------------------------------

------------------------------------------------------------

------------------------------------------------------------

--------------------------------------

>LLGY135

---------------------------------------------------HAA-----H

Q-GV--------TSMTA-RANVS------------VFWPGITT----------DIARL--

-----RNS-CMD-CN--RI------SP-S-QPNAPP-TTP---------------VDPEF

PFQCICADY-----F-TY---------------KGA----HYLIIVD----RYS------

--NWPI-IK-------KTS-----G-G-AA------GLV-KS--L-REEFITYG------

--------------IAEELASDG-GPEFVAT---------ETQEFLKS------------

-----------WGV--RH---RLSSVAYPHSNCRA--EIGVKSCKRLLMH--------NT

G-PNG----ELDTPSFQRAMLQYRNTP---------------DQD----TKMSPAMI---

---------------VFG-----RSIRD---FI---------------------------

------------------PVL---------------------------------------

----------------------------------------P---GRYT------------

------------------------------------------------------------

--------------------------------------

>LLGY136

----------------------------------------STMKDEVLQELHDKM---GH

Q-GI--------DRVEK-LVRSR------------FYWQNIRS----------DIQHW--

-----ISM-CER-CNLAKM-------P-H-LEVRTP-MHS---------------IVARE

PLEVIAIDF-----T-VL----EPASN------GME----NVLVMTD----VYS------

--KFTV-AV-------PTT----NQ-T-AQ------TVA-KA--LVQEWFSRYG------

--------------VPCRIHSD--GRCFDAK---------IVTELYKI------------

-----------YAI--QK---SRTTPYHPMGNGQC--ERYNRTMHALLRTLT-P----TQ

K--------SKWPEHLQEITYAYNVIP---------------HAA----TGFSPFYL---

---------------MFS-----RVPHL--------------------------------

------------------PVNIRLH-----------------------------------

------------------------------------------------------------

------------------------------------------------------------

--------------------------------------

>LLGY137

A--------------------------------------------------HQG-----H

Q-GM--------VKTKR-LIREK------------VWFPGIDV----------LVEKR--

-----VKR-CMA-CQASTH------LP-E-SSTEPL-KMS---------------KLPEG

PWQHVDIDF-----CGPF--------P------SGD----YLLVAID----EYS------

--RFPE-VE-------ITR----ST-S-AY------STI-PK--L-DKIFSTHG------

--------------IPEVVKSDN-GPPFQSS---------EFKSFAEY------------

-----------TGF--QH---RKITPEWPQANSEV--ERFMRTLEKAIRCAI--------

--LEG----KVWKQEMYRFLRSYRATP---------------HSS----TGVSPATA---

---------------LFN-----RNIKTTL------------------------------

------------------PENKEASKSD--------------------------------

-------------------------RTMREADA-RAKSRMK---QYADKRA------KAK

S--SNL-------------Q-----PGDIV--------MMKQ----------------RR

TNKYSTPY-------QPMAYEVVARQGP---------M

>LLGY138

--------------------------------------------------IHAG-----H

Q-GI--------EKCKM-RARTS------------VFWNGINN----------DLEDV--

-----VKR-CAV-CQ--EH------QH-T-NPRETL-LPH---------------ELPTR

SWQILGTDL-----F-HY---------------NNS----EYLIVVD----YYS------

--KFPF-IR-------KMPT---PC-T-SH------AVV-AA--T-ADIFSEHG------

--------------VPEKVVSDN-GPHYDCV---------NYKKFAQE------------

-----------WGF--EH---VTSSPHFPQSNGFV--ERTIQTVKRTLL-----------

---KA----KESNMNPCKAMLCLRTTP---------------LDH----HLPSPSEL---

---------------LYA-----RKLK---------------------------------

------------------------------------------------------------

------------------------------------------------------------

------------------------------------------------------------

--------------------------------------

>LLGY139

------------------------------------------------------------

------------------------------------------S----------DIENT--

-----VQQ-CGQ-YK--EN------AQ-N-LTRAPL-RPW---------------LFPQK

PWSRVHLDH-----VGPI---------------ENK----MILVAVD----AYS------

--KWIE-P---------------------------------------KVYSSCG------

------------------------------------------------------------

------------------------------------------------------------

------------------------------------------------------------

------------------------------------------------------------

------------------------------------------------------------

------------------------------------------------------------

------------------------------------------------------------

--------------------------------------

>LLGY140

------------------------------------------------------------

------------------------------------------------------------

------------------------------------------------------------

---------------------------------------------------QKS------

--KYPI-VK-------ELQA---PF-T-SA------AVT-DV--I-EDACAMFG------

--------------RPDQIRSEN-GPQYAGQ---------HFRNFCRR------------

-----------WGI--QH---VTSSPHYAQSNGFS--ERQVRWIKSIIK-----------

---KC----IKTSE----------------------------------------------

------------------------------------------------------------

------------------------------------------------------------

------------------------------------------------------------

------------------------------------------------------------

--------------------------------------

>LLGY141

---------------------------------------------------HVG-----H

Q-GV--------VKTKM-LLREK------------VWFPAIDS----------MAERQ--

-----VKS-CLA-CQATIS------TP-M-TP-EPI-IST---------------PIPSA

PWKNLSADF-----LGPL--------P------TGE----LILVVID----DFS------

--RFPE-VE-------IVT----ST-A-AS------SVI-PK--L-DSMFARHG------

--------------IPDVLKSDN-GPPFNGS---------ELTKFAQY------------

-----------LGF--QH---KKITPIWSCANGEA--ERFMAPLMKAIRAAH--------

--VEN----RSWKQELYNFLRQYRATP---------------HCT----TGVSSAEA---

---------------LYG-----RKLN---------------------------------

------------------------------------------------------------

------------------------------------------------------------

------------------------------------------------------------

--------------------------------------

>LLGY142

------------------------------------------------------------

------------------------------------YWPRMTT----------QVKDY--

-----ISK-CDI-CL--SH------RS-A-PPREPL-QQH---------------DFVAR

PWSKIGADL-----C-QL---------------HGR----TLLVVCD----YYS------

--NFFE-VA-------RLN----TV-T-TR------SVV-RE--F-LPMFARFG------

--------------LPDVLVTDN-GPQFASA---------EFAVFVRK------------

-----------KGI--TH---LTSSPHYAQSNGKS--ENAVKTLKLIFA-----------

---KA----KQSGESEYMALLDWRNTP---------------SEG----MGTSPAQR---

---------------LMG-----RRCKT---LL---------------------------

------------------------------------------------------------

------------------------------------------------------------

------------------------------------------------------------

--------------------------------------

>LLGY143

---------------------------------------------------HTG-----H

I-GV--------VKIKG-VARSY------------VWWPGMDA----------HIESC--

-----TKA-CEL-CQ--LV------QR-N-PTNAPV-HP----------------IPSSR

PIERVHIDY-----AGPV---------------EGK----MLLIVVD----SYS------

--KWPE-VV-------IQN----ST-T-SE------ATV-NA--L-RTIFSRGG------

--------------IPHTLVSDN-GPQFNSQ---------EFK---DW------------

-----------LGV--LH---KPTSPYHPSSNGQA--ERFVQTVKQALKA---------M

A-SSG----ESLQVRLDKFLLAYRNAP---------------HAF----TGELPAVR---

---------------FMG-----RQLRTRLDSVK--------------------------

------------------PDNR--------------------------------------

--------------------------------R-ENKRLEK---QMERG--------SQN

L--RSF-------------R-----EGDMV--------WVRD-Y-HG-------------

KDKWVPGTINNK--CGPLTYQVTVS-------------

>LLGY144

------------------------------------------------------------

------------------------------------------------------------

------------------------------------------------------------

------------------------------------------------------------

----------------LLS----NE-S-SR------QVI-IH--L-KSLFSRYG------

--------------IPSGCISDG-GPQFASE---------EFRQFTSE------------

-----------WGI--EH---KMSSPYYPQSNGLA--ENGVKIVKRLLR-----------

---KA----AGRKEDAYLALLAYRASP---------------LD-----CGKSPAEL---

---------------LFG-----RKIRT---RL---------------------------

------------------P-----------------------------------------

------------------------------------------------------------

------------------------------------------------------------

--------------------------------------

>LLGY145

--------------------------------------------------LHLA-----H

Q-GV--------EKTRL-RARSC------------VYWININC----------DIENM--

-----IQR-CDI-CQ--RE------LC-A-QPSEPL-MQH---------------EVPSR

PWQVVGTDL-----F-SI---------------GRN----NYLIIGD----YYS------

--KFPF-VE-------LIEG---RA-T-SD------MIV-KL--T-KRIFSEQG------

--------------VPDRVVSDN-GGHFDSQ---------AYKLFAKA------------

-----------WGF--EH---VTSSPHYPRSNGFV--ERQIQTIKRTLK-----------

---KA----ASARVDTDMAMLILRSTP---------------IDH----HLPSPAEM---

---------------LNA-----RKMRA---NL---------------------------

------------------PVKI--------------------------LNAH--------

-----PEKG-----------------AISERLF-ERQRQQK---VYHD------------

------------------------------------------------------------

--------------------------------------

>LLGY146

---------------------------------------------------HES-----H

Q-GA--------DKCKT-RRRTV------------LYWPGMSQ----------DIETI--

-----VGR-CHI-CL--KF------RA-S-NPKEPL-IPH---------------DVPER

PWQKVAADI-----I-TF---------------KSR----DYIVAVD----CYS------

--KYPE-IA-------LLE----SK-T-AS------NVI-IH--F-KSIFARHG------

--------------IPEEMMSDN-MP-FASQ---------EFTNFGRD------------

-----------WGI--KL---TTSSPNFPQSNGQS--ARAVQTLKRVLK-----------

---RA----DCEGRDPYVALLEYRNTP---------------VAD----ALFSPAQM---

------------------------------------------------------------

------------------------------------------------------------

------------------------------------------------------------

------------------------------------------------------------

--------------------------------------

>LLGY147

---------------------------------------------------HDS-----H

Q-GV--------DRTKR-RARQS------------VYWPGISN----------DIATT--

-----VSS-CDK-CQ--ER------LP-S-QQREPM-RAE---------------SPPSR

AFEDVSADF-----F-NY---------------KGR----DYLVYVD----RLS------

--GWPA-VI-------HFPKG--TT-T-SR------HTI-HA--C-ARLFVDLS------

--------------IPVRFRSDR-GPQFASR---------EFQQFLKR------------

-----------WDV--VA---APSTPHFAQSNGHA--ESAVKAVKKLIATTT--------

--VRG----DLDDENFQRGLLEYRNTP---------------RVG-----GLSPAQI---

---------------LFG-----HPLRSAM------------------------------

------------------P-----------------------------------------

----------------------------------AHRKSFA---KEWQQTA---------

---DDY-------------D----------------------------------------

--------------------------------------

>LLGY148

------------------------------------------------------------

------------------------------------------------------------

--------------------------------------------------------LPSR

KWSHLCADF-----YGPL--------P------SGE----YLLVVLD----EYS------

--RFPE-VE-------------------AQ------TVI-PI--F-DKIFSSRG------

--------------IPEKLKTDN-GTPF------------------RV------------

-----------LMF--CQ---RS-------------------------------------

------------------------------------------------------------

------------------------------------------------------------

------------------------------------------------------------

------------------------------------------------------------

------------------------------------------------------------

--------------------------------------

>LLGY149

------------------------------------------------------------

------------------------------------------------------------

------------------------------------------------------------

------------------------------------------------------------

------------------A----TT-G-SK------AVI-TK--L-KAHFARMG------

--------------IPDTVISDN-GPQLISD---------EFANFSRT------------

-----------WGF--EH---LTTSPHHSRSNGKV--ESSVKAAKKMIK-----------

---KA----RKAGEDQ--------------------------------------------

------------------------------------------------------------

------------------------------------------------------------

------------------------------------------------------------

------------------------------------------------------------

--------------------------------------

>LLGY150

---------------------------------------------------HTG-----H

I-GV--------VKTKG-VAPSC------------VWWPGMDA----------DIESC--

-----TKA-CES-CQ--LV------QR-N-PTKAPV-HPW---------------IPSSR

PGERGHIDY-----AGPV---------------EGK----ILLIVVD----SYS------

--KWPE-VV-------IQN----ST-T-SE------ATV-NA--L-RTIFSRGG------

--------------IPHTLVSDN-GTQLKSQ---------EFKDFLNW------------

-----------LGV--LH---RHI------------------------------------

------------------------------------------------------------

------------------------------------------------------------

------------------------------------------------------------

------------------------------------------------------------

------------------------------------------------------------

--------------------------------------

>LLGY151

----------------------------------------STMKDEVLQELHDKM---GH

Q-GI--------DRVEK-LVRSR------------FYWPNIRS----------DIQHW--

-----ISM-CER-CNLAKM-------P-H-LKVRTP-MHS---------------IVARE

PLEVIAIDF-----T-VL----EPASN------GME----NVLVMTD----VYS------

--KFTI-AV-------PTR----NQ-T-AQ------TVA-KA--LVREWFFRYG------

--------------VPCRIHSDQ-GRCFDAK---------IVTELYKI------------

-----------YAI--QK---SRTTPYHPMGNGQC--ERYNRTMHALLRTLT-P----TQ

K--------SKWPEHLPELTYAYNVTP---------------HAA----TGFSPFYL---

---------------MFS-----RVPRL--------------------------------

------------------PVDIR-------------------------------------

------------------------------------------------------------

------------------------------------------------------------

--------------------------------------

>LLGY152

------------------------------------------------------------

------------------------------------------------------------

------------------------------------------------------------

------------------------------------------------------------

-----------------MA----DA-T-AV------SCA-RA--LLENWVPRFG------

--------------VPNDIVSDR-GRQFISG---------LWMELGKL------------

-----------LGM--QV---HHTTAYHAQSNGLV--ERFHRQLKASLKARL--------

---HG----PDWRDELPIVLLGIRCSI---------------KED----FGCTSAKL---

---------------VYG-----TTLRL--------------------------------

------------------PGEFFETTKASTEVTT-------DAL-TLLTRLR--------

-----KTM-----------------RSLRVKSM-THHG--------RQHV-------SRI

P--TAL-------------L-----TCTFV--------FVRK-DAHR----------TPL

ECPYEGPFRVLE---GNDKYYTLDIRG-----------

>LLGY153

---------------------------------------------------HAC-----H

P-GM--------TKMKG-LARAT------------LWWP-LDQ----------AIEDK--

-----VKS-CHM-CQ--VN------QN-A-PVKAPL-HPW---------------EWPER

PWSRTHIDH-----AGPY---------------HNQ----LWLIIVD----APS------

--KWLD-IY-------PVS----ST-S-SQ------TTI-DM--L-RVSFSNHG------

--------------LPEMIVRDN-ATSFTSE---------QFAEFCEK------------

-----------NGI--HH---VTSVPYHPASNGLA--ESAVQTFKSGFDK---------M

G---E----GSLKTKLVRFLLQYRNAP---------------QGT----TGQSPAEL---

---------------LMG-----RRLRSHLDLLH--------------------------

------------------PSLSQR------------------------------------

-------------------------------VQ-RRQRYQN---EQHDQ--------HAH

G--RSI-------------E-----IGDRV--------YSRN-F-SG-------------

KPDWLSGIVTEK--SGPVSYRLKL--------------

>LLBP2

------------------------------------------------------------

----------------------G------------YWIIKARA----------TVTSY--

-----LWN-CVK-CRKMRG------GT-VTQKMAE--LPEDR-------------LEPSD

PFTYSAVDF-----FGPFF---IKEGR----SEKKK----WGVLFTC----MAS------

--RAVH-I--------ETA----NSLS-TDSF-----IN-----AYRRFVGRRG------

--------------PVRQLRSDR-GTNFVGAR-------SELEAALAE--MNDGK-----

ITAEL----LKQNC--DWVTFKMNPPHASHMGGVW--ERMIRSVRNVLSALL-----NAH

GDRLD---DEQLRTLMVEAEAVVNSRPITYP----------DTTVPDSGEPLSPSQI---

---------------LTL-----KSR----------------------------------

---------------VVLPPPGI-------------FMKEDLYCRKRWRLVQ--------

-----FLANQF----WNRWR--------TEYLL-ALQERSKW---------------NKQ

H--HNL-------------K-----KGDIV--------LVKD-ECNPR----CQWPLARV

NQIYPS----EDGLVRKVKV------------------

>LLGY154

------------------------------------------------------------

------------------------------------YWPRMTT----------QVKDY--

-----ISK-CDI-CL--SH------RS-A-PPREPL-QQH---------------DFVAR

PWSKIGADL-----C-QL---------------HGR----TLLVVCD----YYS------

--NFFE-VA-------RLN----TV-T-TR------SVV-RE--F-LPMFARFG------

--------------LPDVLVTDN-GPQFASA---------EFAVFVRK------------

-----------KGI--TH---LTSSPHYAQSNGKS--ENAVKTLKLIFA-----------

---KA----KQSGESEYMALLDWRNTP---------------SEG----MGTSPAQR---

---------------LMG-----RRCKT---LL---------------------------

------------------------------------------------------------

------------------------------------------------------------

------------------------------------------------------------

--------------------------------------

>LLGY155

---------------------------------------------------HAA-----H

Q-GV--------TSMTA-RANVS------------VFWPGITT----------DIARL--

-----RNS-CMD-CN--RI------SP-S-QPNAPP-TTP---------------VDPEF

PFQCICADY-----F-TY---------------KGA----HYLIIVD----RYS------

--NWPI-IK-------KTS-----G-G-AA------GLV-KS--L-REEFITYG------

--------------IAEELASDG-GPEFVAT---------ETQEFLKS------------

-----------WGV--RH---RLSSVAYPHSNCRA--EIGVKSCKRLLMH--------NT

G-PNG----ELDTPSFQRAMLQYRNTP---------------DQD----TKMSPAMI---

---------------VFG-----RSIRD---FI---------------------------

------------------PVL---------------------------------------

----------------------------------------P---GRYT------------

------------------------------------------------------------

--------------------------------------

>LLGY156

---------------------------------------------------HNKQ---GH

Q-GV--------ERTEK-LVRAR------------CYWPTLHK----------DVQTW--

-----INL-CER-CTLGKF-------P-A-KKMRTP-LGR---------------LIATR

PLEVVAIDY-----T-VL----EPSSD------GRE----NVLIITD----VFT------

--KFTV-AV-------VTR----NQ-K-AE------TVA-KA--LVTQWFEPYG------

--------------IPQRIHSDN-GRNFDSV---------LISELCKL------------

-----------YGI--QR---SHTTPYHPAGNGQC--ERFNRTLHDLLRTLS-E----KK

K--------RRWAEHVHEVVNAYNLTP---------------HSS----TGYAPFYL---

---------------MFG-----RDSRQ--------------------------------

------------------PIDLLLGTGEQEVEPS-DWVSRHQ------QRLQ--------

-----EAY-----------------QLARRQLV-HEADSRK---KHYDR--------KAR

D--LPL-------------S-----FGQRV--------Y---------------------

--------------------------------------

>LLGY157

------------------------------------------------------------

------------------------------------YWPRMTT----------QVKDY--

-----ISK-CDI-CL--SH------RS-A-PPREPL-QQH---------------DFVAR

PWSKIGADL-----C-QL---------------HGR----TLLVVCD----YYS------

--NFFE-VA-------RLN----TV-T-TR------SVV-RE--F-LPMFARFG------

--------------LPDVLVTDN-GPQFASA---------EFAVFVRK------------

-----------KGI--TH---LTSSPHYAQSNGKS--ENAVKTLKLIFA-----------

---KA----KQSGESEYMALLDWRNTP---------------SDG----MGTSPAQR---

---------------LMG-----RRCKT---LL---------------------------

------------------------------------------------------------

------------------------------------------------------------

------------------------------------------------------------

--------------------------------------

>LLGY158

---------------------------------------------------HEP-----H

M-GM--------EKTKS-RARSA------------IFWPGMSK----------AIEDT--

-----VSR-CKT-CL--QF------AR-S-NQKEPM-IAH---------------KIPDG

PFRKVAMDI-----M-SF---------------KGR----DYLVVVD----YYS------

--KYPE-LA-------LLE----NK-T-SE------CVI-SH--V-KSISARHG------

--------------IPEEIVADN-QP-FGSY---------SFRQFAEN------------

-----------WGI--TV---TTSSPTYAQSNGQA--ERVVQTLKSLLK-----------

---KA----DEEGRDPYIAMLEYRNTP---------------ISG----LRYAPAQL---

---------------AMS-----RLLRS---KL---------------------------

------------------PTTK--------------------------AVLE--------

-----PRVVN-----------------AKQDLT-DRQTRFK---QDYDHGA-------TS

------------------------------------------------------------

--------------------------------------

>LLGY159

---------------------------------------------------HQS-----H

R-GV--------VRMKT-MARLY------------VWWPNIEA----------SVEAC--

-----CKA-CNV-CA--VT------AP-A-PT-ANL-SPW---------------PLPDE

PWDRIHVDF-----AGPF---------------LGN----MWMLVMD----AYS------

--KWPS-VV-------RMS----KYPT-TE------TTI-MA--L-NILFTTWG------

--------------SPKTLVSDN-GPQFGSK---------QFEDWCRL------------

-----------NGI--VH---LTSAPFHPPSNGEA--ERLVGVFKTAMQR--------SV

G-EEG----KERDKATMGFLREYRSTP---------------NCA----TGRTPAEL---

---------------MIG-----RQVRTPLSLLQ--------------------------

------------------PSVHH-------------------------------------

------------------------------------------------------------

------------------------------------------------------------

--------------------------------------

>LLGY160

---------------------------------------------------HVG-----H

I-GV--------VKMKG-LARSY------------VWWPGIDK----------CIESL--

-----ARK-CQG-CP--RV------QF-E-APTVPL-HPW---------------EWPAK

PWQRIHVDY-----AGPF---------------MGR----MFLIAVD----AHS------

--KWPE-VL-------PTV----SA-S-SE------KTV-EL--L--DVFARYG------

--------------LPEHLHSDN-GSQFTSE---------VFRNFMKA------------

-----------NNI--RH---TFSAPYHPATNGQV--ERFVQTFKQAMRSAR--------

--GDS----GAVKRHLAQFLFAYRNAP---------------HAT----TGDSPAML---

---------------LMG-----RGLRTRLDVIR--------------------------

------------------PNTRKT------------------------------------

-------------------------------VE-NHQATSI---EERRGQ-------HR-

----AF-------------E-----VGDRV--------AVRN-Y-RN-------------

DHKWVPGTIQEK--KGTRSYAVLV--------------

>LLGY161

---------------------------------------------------HDG-----H

P-GV--------VKSRE-RAKIS------------VWWPGLSD----------EISTM--

-----VEN-CSH-CQ--IG------RS-T-QSSEPL-MTA---------------PLPDR

AWSHLAADV-----C-EL---------------KGK----KYLVVIY----YYS------

--RYLE-IA-------HMT----TT-A-SR------AVI-LQ--F-KDMFARWG------

--------------LCDKITTDN-GPQFSSD---------EFRQLTND------------

-----------YTF--QH---VTSSPGFPQSNGEA--ERVVEIAKKILV-----------

---Q---------DDPSLGLMTYRATP---------------VAA----TGRSPATM---

---------------MTQ-----RE-----------------------------------

------------------------------------------------------------

------------------------------------------------------------

------------------------------------------------------------

--------------------------------------

>LLGY162

------------------------------------------------------------

------------------------------------------------------------

------------------------------------------------------------

------------------------------------------------------------

-------LM-------RQSS---ST-R-TK------CVI-DA--M-MSQFARHG------

--------------VLEVVMSDN-GPQFSCG---------EFREFAQR------------

-----------WDF--EH---ITSSPRYPQGNGQV--ERAIGTVKNLVK-----------

---KA----MEDGNDVQLALLNFRNTV---------------RDG----YSASPAQL---

---------------LFG-----RRCRT---LL---------------------------

------------------PIQR--------------------------SRLI--------

-----PKL-------------------ANDKKI-AKNAQIA---QYNK------------

------------------------------------------------------------

--------------------------------------

>LLGY163

---------------------------------------------------HEA-----H

Q-GI--------EKTRL-RARSC------------VYWKAINR----------DIDDI--

-----VRK-CDT-CQ--QL------QR-R-QAHEPL-MQH---------------ELPTR

PWQIVGTDL-----F-VI---------------RLD----TYLLMCD----YYS------

--KFPF-VY-------RIEG---RV-T-SD------AII-SK--M-SEVFAENG------

--------------SPDKVVSDN-GGHYSSQ---------AFRNFANE------------

-----------WCF--DH---VTSSPHFPQSNGFI--ERQVQTVKSTLK-----------

---KA----AMTR-----------------------------------------------

------------------------------------------------------------

------------------------------------------------------------

------------------------------------------------------------

------------------------------------------------------------

--------------------------------------

>LLGY164

--------------------------------------------------AHKG-----H

I-GI--------DATKR-RARDV------------MFWPNMNS----------DIEKA--

-----ITA-CET-CQ--SM------TY-H-QQKEPL-MSY---------------PVPNQ

PWATVGTDL-----F-EW---------------HNC----MYLITVD----SYS------

--GWYE-ID-------LLR----DT-S-TS------TII-KK--M-KTHFSRFG------

--------------IPLMVISDS-GSQYKSR---------EFKQFARD------------

-----------WSF--NH---VMSSPHFHSSNGLA--EKAVQTAKKLLE-----------

---KS----YRDGTDAHLNLLNWRNTP---------------RDA----VLGSPAQR---

---------------NIS-----RRTRT---TL---------------------------

------------------PTTQ--------------------------TLLK--------

-----PQVLN--------------QQTVQHQLS-EKRLQQK---KYAD------------

------------------------------------------------------------

--------------------------------------

>LLGY165

---------------------------------------------------HQS-----H

R-GV--------VRMKT-MARLY------------VWWPNIEA----------SVEAC--

-----CKA-CNV-CA--VT------AP-A-PT-ANL-SPW---------------PLPDE

PWDRIHVDF-----AGPF---------------LGN----MWMLVMD----AYS------

--KWPS-VV-------RMS----KYPT-TE------TTI-MA--L-NILFTTWG------

--------------SPKTLVSDN-GPQFGSK---------QFEDWCRL------------

-----------NGI--VH---LTSAPFHPPSNGEA--ERLVGVFKTAMQR--------SV

G-EEG----KERDKATMGFLREYRSTP---------------NCA----TGRTPAEL---

---------------MIG-----RQVRTPLSLLQ--------------------------

------------------PSVHH-------------------------------------

------------------------------------------------------------

------------------------------------------------------------

--------------------------------------

>LLGY166

---------------------------------------------------HTA-----H

L-GK--------EKTKL-LARDT------------VYWLNINK----------DIDRL--

-----VQT-CNV-CQ--EH------QS-S-QVPEPL-LQH---------------DIPYK

PWSVLGTDL-----F-EF---------------EGH----QWLIIAD----YYT------

--KYPI-IR-------QLPN---PS-P-SS------VVV-NA--T-KQIFAEFG------

--------------IPDRIVSDN-GPHFASE---------AYREFARM------------

-----------WQF--DH---ITTSPRRPQGNGFI--ERQVRTIKSLLK-----------

---KS----KQSGTDYQLALLHWRTTP---------------INA----NLASPAQL---

---------------IMG-----RRLKS---TI---------------------------

------------------------------------------------------------

------------------------------------------------------------

------------------------------------------------------------

--------------------------------------

>LLGY167

---------------------------------------------------HNA-----H

S-GI--------VRMKV-VGRSF------------MWWPGIDS----------DIERT--

-----VNS-CDI-CR--RS------RH-K-PTEAPL-QPW---------------SFPDR

PWSRVHIDY-----AGPV---------------MGK----MILVVID----AHS------

--KWIE-AY-------TTS----GS-T-SA------ITI-SK--L-KWIFSSHG------

--------------IPDVIVSDN-ATGFVSE---------EFQSFCRR------------

-----------NGI--KH---VTSAPHHPATNGLA--ERAVGILKGGVQR---------L

Q--------GDLETRIAHFLLDYRITP---------------HTT----TGVSPAEL---

---------------LTG-----RKLRTRLDRII--------------------------

------------------PDVSGR------------------------------------

-------------------------------AI-SKQTTQK---ERHDQ--------HTQ

A--RQY-------------Q-----PGDLV--------YALM-YRGN-------------

KTNWSPGTVVTQ--TGPVSYTVRLE-------------

>LLGY168

------------------------------------------------------------

------------------------------------------------------------

-------------------------------------------------------PFGSY

PMEFVAADL-----IGPF----TESHN------GNK----YILTIID----FCT------

--GWAE-AI-------PIP----TK-S-NQ------AVW-DA--FSNGFICRHG------

--------------VCRVLLTDH-GAEFTAL---------AFERYLSQ------------

-----------IGI--EH---RMSTPAHPQSNGKI--ERFNRTLKQMIQKAV-N----NQ

P--------SRWEEVLNDVLLAYRASV---------------STT----TGYTPHFL---

---------------MTG------------------------------------------

------------------------------------------------------------

------------------------------------------------------------

------------------------------------------------------------

--------------------------------------

>LLGY169

---------------------------------------------------HCR---FGH

L-GK--------QNMTK-LMNSD-------------LVKDMAVS---------TQTLT--

-------F-CEP-CAQGKA------HQ-QSYPKISD-TR----------------SA--D

ILDLIHADV-----CGPIN---PVSLG------GKC----YFVTFTD----DCS------

--RFVW-VR-------FIR----HK---SE-------VF-Q---KFRDLIKELEKGTG--

-------------RKLKALRSDR-GGEFLSN---------EFQQYLKR------------

-----------RGI--HH---QLTTADSPQQNGVA--ERMNRTLIEKAHAMI-----AAA

NVSKT-----FWAEAIANAAYVRNRSP---------------TSSL---RNMTPFEA---

---------------WWG------------------------------------------

------------------------------------------------------------

------------------------------------------------------------

------------------------------------------------------------

--------------------------------------

>LLGY172

------------------------------------------------------------

------------------------------------------------------------

----------DT-CQ--LH------NK-S-PPAAPL-HPW---------------EWPEK

PWTRIHIDY-----AGPF---------------LGK----MLLVAVE----ATS------

--KWIE-TH-------IMS----ST-T-ST------ATV-NK--L-REIFAQHG------

--------------LPEVLVSDN-AVNFISE---------QFETFVRK------------

-----------NGI--VH---VTSAPYHPASNGLG--ERAVHTVKSGITK----------

--TAG----DNMEVKLRRFLFDYRRTP---------------QST----TGKSPMEI---

---------------LNN-----WKMKSRLDLLH--------------------------

------------------PSLQGK------------------------------------

------------------------------------------------------------

------------------------------------------------------------

--------------------------------------

>LLGY173

---------------------------------------------------HAA-----H

Q-GV--------TSMTA-RANVS------------VFWPGITT----------DIARL--

-----RNS-CMD-CN--RI------SP-S-QPNAPP-TTP---------------VDPEF

PFQCICADY-----F-TY---------------KGA----HYLIIVD----RYS------

--NWPI-IK-------KTS-----G-G-AA------GLV-KS--L-REEFITYG------

--------------IAEELASDG-GPEFVAT---------ETQEFLKS------------

-----------WGV--RH---RLSSVAYPHSNCRA--EIGVKSCKRLLMH--------NT

G-PNG----ELDTPSFQRAMLQYRNTP---------------DQD----TKMSPAMI---

---------------VFG-----RSIRD---FI---------------------------

------------------PVL---------------------------------------

----------------------------------------P---GRYT------------

------------------------------------------------------------

--------------------------------------

>LLGY174

---------------------------------------------------HNKQ---GH

Q-GV--------ERTEK-LVRAR------------CYWPTLHK----------DVQTW--

-----INL-CER-CTLGKF-------P-A-KKMRTP-LGR---------------LIATR

PLEVVAIDY-----T-VL----EPSSD------GRE----NVLIITD----VFT------

--KFTV-AV-------VTR----NQ-K-AE------TVA-KA--LVTQWFEPYG------

--------------IPQRIHSDN-GRNFDSV---------LISELCKL------------

-----------YGI--QR---SHTTPYHPAGNGQC--ERFNRTLHDLLRTLS-E----KK

K--------RRWAEHVHEVVNAYNLTP---------------HSS----TGYAPFYL---

---------------MFG-----RDSRQ--------------------------------

------------------PIDLLLGTGEQEVEPS-DWVSRHQ------QRLQ--------

-----EAY-----------------QLARRQLV-HEADSRK---KHYDR--------KAR

D--LPL-------------S-----FGQRV--------Y---------------------

--------------------------------------

>LLGY175

--------------------------------------------------IHLG-----H

V-GI--------DGCLR-RARES------------VYWPGMNG----------EIKEY--

-----IQT-CET-CR--EF------EC-S-HTRETL-MSH---------------EVPDR

PWQKVGVDL-----F---------------------------------------------

------------------------------------------------------------

------------------------------------------------------------

------------------------------------------------------------

------------------------------------------------------------

------------------------------------------------------------

------------------------------------------------------------

------------------------------------------------------------

------------------------------------------------------------

--------------------------------------

>LLGY176

------------------------------------------------------------

-------------------AREC------------LYWPNMAA----------DIRQV--

-----VEK-CET-CR--SY------ER-S-QQKETL-ITR---------------ETPTL

QWEKVGVDL-----F-SW---------------EGR----DYQVIVD----YTS------

--NFWE-VD-------RMN----ST-T-TT------SVI-KQ--L-KSHFARFG------

--------------IPSVVVSDN-GPQYVSE---------EFCTFAAK------------

-----------WDF--EH---QTSAPGHQNANGKA--EAAVKAAKLMFR-----------

---KC----KKSQTDPYLALLEIRNTP---------------TQG----LGSSPAQR---

---------------LLN-----RRTRT---LL---------------------------

------------------P-----------------------------------------

------------------------------------------------------------

------------------------------------------------------------

--------------------------------------

>Mag

---------------------------------------------------HDP-----H

M-GI--------VKTKS-LARSY------------VWWPGID-----------EAETE--

-----CRA-CTV-CA--AV------AD-A-PSTHAP-RSW---------------PWPSR

PWSRLHLDF-----LGPI---------------GGV----TYLVVVD----SCS------

--KWIE-AI-------KMQ----RT-T-AQ------AVI-SV--L-RDLWSKFG------

--------------LPKQTVSDN-GPPFSSS---------DFQKFLIH------------

-----------NGI--KH---IYSAPYHPASNGAA--ENAVKICKRAIKKAL--------

--KQN----LNVDTALCRFLLAYRNTE---------------HAT----TGDSPANI---

---------------LQG-----RSLRMRLDNLK--------------------------

------------------PERQSR------------------------------------

-------------------------------VI-AQQERSE---QNA----------GGV

Q--RQL-------------E-----PGTKV--------WYRD-Y-RG-------------

LDKWVPGTILKQ--LGSRDYCVRSS-------------

>Gulliver

-------------------------------------------------HFHLG-----H

P-GV--------TRMKA-LMRCY------------VYWPFMDR----------SIEEY--

-----VAK-REQ-CI--EF------SR-Q-PPKADS-QPW---------------PEVSK

PWIRLHVDY-----AEPV---------------NGR----YFLVVVD----AYS------

--KWPE-IF-------AVK----TS-K-TS------ETK-FH--L-RQLFGRFG------

--------------APNILVSDN-GSQFISA---------KFADFCQR------------

------------GI--EH---IRIPPYHPQSNVQA--ERFVHIFKRALFKGG--------

--REA----VNDEEALQKFLILYRATP---------------NLIR--KDGKSLAEI---

---------------MFG-----RSMRIFSDIVH--------------------------

------------------PRDKVK------------------------------------

-------------------------------VT-SNN----------DGND------YKR

A--RDF-------------N-----VGDYA--------HASD-YRPG-------------

RPKWINGTIVAR--RGKMLYEVRVG-------------

>DRM

---------------------------------------------------HVG-----H

Q-GM--------VKMKA-LARKY------------VWWPKMDA----------ELEQV--

-----CRT-CEP-CQ--ME------QK-A-PRQVPL-HPW---------------EFPGQ

SWKRLHIDF-----AGPF---------------LGH----TFMIIVD----AYS------

--KWLE-VF-------RMP----NL-T-SQ------STI-SR--L-RRLFAAYG------

--------------LPEHIVTDN-GTQFTSE---------EFKNFMQQ------------

-----------NGI--LH---STSAPGHPATNGLA--ERYVQTFKGGIKK---------L

A-HVT----MDLEDKISLFLMQYRTTP---------------NCT----TGQSPADL---

---------------FLN-----RHVRTRLDFVH--------------------------

------------------PDVTVA------------------------------------

-------------------------------VR-RKQYLQK---FHHDK--------RSV

E--RSF-------------S-----EKDAV--------YLRN-TTGK-------------

GNKWVPGVIVKQ--TGPVSYNVQGQ-------------

>CFG1

---------------------------------------------------HEG-----H

P-GI--------VRMKA-LARSH------------VWWPGIDS----------DIQDK--

-----VQA-CTE-CQ--LQ------IP-V-PPVAPL-HSW---------------DWPDR

PWSRIHVDY-----AGPF---------------LGQ----MFLVVVD----SYS------

--KWLE-VV-------PTN----SS-T-ST------VTI-AK--L-RQIFAEHG------

--------------LPDKLVSDN-GPCFISE---------EFEIFLRE------------

-----------NGI--QH---VKISPHHPATNGLA--ERSVRIFKEGMKK---------M

G-TSG----GDTAAKLSRFLLSYRSTP---------------QTT----TGLSPAEL---

---------------LFN-----RKLRTKLDLVK--------------------------

------------------PDVRER------------------------------------

-------------------------------VV-KRQQTQK---NYHDS--------RAK

E--RVF-------------A-----EGDKV--------FVKNFY-----S----------

GPKWKSGEIVSK--NSPASYTVEGQ-------------

>Hydra21

---------------------------------------------------HSA-----H

P-GV--------VRMKA-LARSY------------VWWPNMDK----------SIEET--

-----VRR-CRL-CE--LH------QR-S-PESAPI-HHW---------------EYPSK

PWSRIHLDY-----AGPF---------------LGH----MFLIVCD----AYS------

--KWIE-AI-------VMK----NV-K-SE------NLI-EQ--L-RSVFAIHG------

--------------VPEVIVSDN-GTSFSSA---------AFAEFVKR------------

-----------NSI--RH---IFTAPYHPSSNGQA--ERMVQTFKEAMKK---------L

TAQQG----NSIETTVNRFLFSYRITP---------------HST----TGISPAEL---

---------------LMK-----RKLRSAFQGLK--------------------------

------------------PDLNNS------------------------------------

-------------------------------VK-EKQERAE---KLSNR--------KAH

L--RKF-------------D-----CGDQV--------MAKN-F-GN-------------

GPKWIPGRIIKQ--KGPVNFEILT--------------

>SPM

---------------------------------------------------HQE-----H

T-GI--------VRMKA-VARSY------------FWYPKLDA----------DIEKL--

-----SQS-CEA-CL--RM------RN-D-PPKVPF-VPW---------------SNARK

PWERVHVDF-----F-EL---------------EGK----DYLVLVD----TYS------

--KWVN-VE-------LMT----ST-T-SA------KTI-ET--L-RSWFAIFG------

--------------LPETLVSDN-GPQFTSE---------EMEVFLSK------------

-----------NGV--KH---VLVPPYHPASNGAA--ERTVQIVKRTLQKYF-LS--DKL

GRNPH----VSIRHRLDNFLFSYRTTP---------------QTV----TGVSPYEL---

---------------VFK-----FAPRTRFSLLK--------------------------

------------------PDLNTR------------------------------------

-------------------------------VT-QKQEKTR---QYHDTP-------GMK

L--RQF-------------E-----SGEQV--------MVKN-HRGG-------------

KERYVLGVIVKR--LGFYSYLVRIG-------------

>SURL

-----------------------------------------------------G-----H

L-GI--------EKCKK-RAREV------------IYWPRINA----------DIAEM--

-----VQS-CTS-CL--MY------KP-K-QQAESL-HPH---------------AVPSR

PWEKVAVDL-----F-TL---------------NKR----EYMVIVD----YYS------

--QFIE-VC-------TMT----ST-S-SK------AVI-NH--M-KAIFARHG------

--------------TPCELMSDN-GPQFASQ---------EFKSFAKE------------

-----------WDF--HH---TTSSPHYPQSNGLA--ENAVKIVKNLLL-----------

---KS----QHCGQDIHRALQVYRSSP---------------LA-----CGKSPAEL---

---------------LYN-----RRLRS---NL---------------------------

------------------PMLD--------------------------TLLD--------

-----NQQID--------------TKSVRGRKE-EQKVKQK---ERFDKHA-------HD

L--SNL-------------K-----PGDHV--------ILQD-MKT--------------

-NTWSKRGIIKSVLNRNRSYQVETE-------------

>Cer4

---------------------------------------------------HEG-----H

P-GV--------VRMKQ-KACSF------------VFWTGIDK----------DVEKL--

-----VRG-CEN-CQ--ES------AK-M-PRVAPL-RPW---------------PEPQK

AWSRVHIDF-----AGPV---------------NGH----WFLVIVD----AKS------

--KYAE-VK-------MTK----TI-S-AS------ATV-SL--L-EEVFATHG------

--------------YPELLVSDN-GTQFTSN---------QFKLMCQE------------

-----------YGM--EH---KTSAVYYPRSNGAA--ERFVDSLKRGLAKIT--------

--RSG----VVTQQALNKFLICYRNTP---------------HSAL---AGATPAEC---

---------------HFG-----RKIRTKMSLLV--------------------------

------------------PSREIVEGP---------------------------------

-------------------------------LS-DCQNRMK---FQYDSRN------AAR

A--KTF-------------E-----LGQYV--------YVRV-QKGN-------------

VWSWEHGEVIKR--LGEVLYEVQVG-------------

>Cer5

---------------------------------------------------HEG-----H

P-GI--------VQMKQ-KARAF------------VFWRGLDS----------DIEKM--

-----VRH-CNN-CQ--EN------SK-M-PRVVPL-NPW---------------PVPET

PWKRIHIDF-----AGPL---------------NGY----YLLVVVD----AKT------

--KYAE-VK-------LTR----SI-S-AV------TTI-DL--L-EEIFSIHG------

--------------YPETIISDN-GTQLTSH---------LFAQMCQS------------

-----------HGI--EH---KTSAVYYPRSNGAA--ERFVDTLKRGIAKIK--------

--GEG----SVNQQILNKFLISYRNTP---------------HSAL---NGSTPAEC---

---------------HFG-----RKIRTTMSLLM--------------------------

------------------PTDRVLKVPK--------------------------------

-------------------------------LT-QYQQNMK---HHYDLRN------GAR

A--KAF-------------Q-----VNQKV--------YVQV-HHGN-------------

KSQWKHGVIRRK--FGGVLYEVQVG-------------

>Cer6

---------------------------------------------------HDG-----H

P-GV--------VHMKQ-KARSF------------VFWRGLDS----------EIEKL--

-----VRQ-CNN-CQ--EN------AK-M-PRVVPL-KLW---------------PEPEK

PWTRIHVDF-----CGPL---------------NGQ----WLLVVVD----AKS------

--KYAE-VK-------LTR----SI-S-AM------STV-DL--M-EEIFSIHG------

--------------YPEVLVSDN-GTQFTSH---------FFKKMCES------------

-----------HGI--IH---KTSATYYPRSNGAA--ERFVDTLKRGIAKIK--------

--GEG----SVNQQILNKFLISYRNTP---------------HSAL---SGATPAEC---

---------------HFG-----RSIRTTMSLLM--------------------------

------------------PKPDANHQAD--------------------------------

-------------------------------LS-EYQKKMK---QQYDSRN------GTR

A--KHF-------------Q-----VGQQV--------YVKV-QHGN-------------

KSEWDYGVVSRK--IGSVLYEVQVG-------------

>HMSBeagle

----------------------------------------------------HNRA---H

R-NA--------VENKA-QLSER------------VYFPKMRK----------KVSAI--

-----VNQ-CLV-CKTAKY------DR-H-PTHPEI-RQT---------------PLPEY

PGQIIHIDI-----Y----------ST------ERH----LVLTAID----KFS------

--KLAM-GR-------VIK----SK-A-VE------DIR-KA--L-RDIVFYYG------

--------------VPKLIVMDN-EKSLNSA---------SIKFMLTD------------

----------QLGI--EL---YKAPPYKSTVNGQI--ERFHSTLSEIMRCL-------KG

D-GTH----RGFEELLDRAIYEYNYTV---------------HSV----TKKRPLEV---

---------------FFG-----RIATVAPEKY---------------------------

------------------------------------------------------------

--------EQ-------------ARLDNIDRLRQKQETDIE----YHNRT-------RKP

I--KTY-------------I-----KGQEI--------FVRV----------NTRLGSKL

SSRFR-----KELVKEDRSTTIL---------------

>Yoyo

----------------------------------------------------HKRA---H

R-SA--------TENKA-QILEN------------FYFPQMNS----------KINKI--

-----IKQ-CKI-CLENKY------ER-H-PSKLVL-KAT---------------PVPNY

PGHIVHIDI-----Y----------HT------NNR----VILTAVD----KFS------

--KYAQ-AR-------IVK----SR-A-TE------DIK-LP--L-QDLLTSFG------

--------------IPEKIVIDN-EKSLNSS---------SIVFMLEN------------

----------QYSI--EI---FKTPPYTSSVNGQV--ERFHSTLTEIMRCT-------KA

E-NKH----NSFEELLNRSICKYNHSI---------------HST----TKKKPIEI---

---------------FFG-----RSVYSDPSLL---------------------------

------------------------------------------------------------

--------EK-------------DRLDNIKKIVNKQEKDLT----FHNKK-------RTE

V--KAY-------------S-----SNDII--------YVKI----------NKRIGNKL

TPRYK-----KEIVLEDNGNTVT---------------

>Gypsyvir

----------------------------------------------------HNRA---H

R-AA--------QENIK-QVLRD------------YYFPNMAS----------LAKEV--

-----VAN-SRV-CTKAKY------DR-H-PKKQEL-GET---------------PIPSY

TGEMLHIDI-----F----------ST------DKK----QFLTSID----KFS------

--KFAV-VQ-------PIL----SR-T-II------DVT-SP--L-LQIVNLFP------

--------------ATKTIYCDN-EAAFNSE---------TITSMLRN------------

----------VYGI--CI---VNAAPLHSTSNGQV--ERFHSTLTEIARCL-------KL

D-KKI----SDTTELILKATIEYNKVL---------------HSV----TLEKPIEV---

-------------------------VHAAAS-----------------------------

------------------------------------------------------------

---------D-------------RRGNVKNRLIKAQQDNIK----RCNAS-------RQN

---RVF-------------E-----VGEKV--------FQKN----------NKRLGNKL

TPLCS-----EQKVEADLGTSVL---------------

>Burdock

----------------------------------------------------HNRA---H

R-AA--------QENVK-QILQY------------YFFPKMSQ----------IAATF--

-----VSN-CLV-CQKAKY------DR-H-PQKQIL-GRT---------------PIPSH

VGETLHIDI-----F----------ST------GRN----YFLTCID----KFS------

--KFAI-VQ-------PIG----SR-T-IT------DLE-PA--I-MQLMNFFP------

--------------HSKTIFCDN-EPSINSE---------SIKSLLKN------------

----------RFNV--DI---ANAPPLHSTSNGQV--ERFHSTLLEIARCL-------KL

D-SGM----NDTVNLILQATIEYNKTV---------------HSV----TNRRPIDI---

-------------------------IHSTPP-----------------------------

------------------------------------------------------------

---------E-------------LANEIVEMVNEAQEKQLR----RENVT-------RRD

---RTF-------------E-----VGETV--------MVKQ----------NNRLGNKL

TPRYR-----EELIEADLGTTVL---------------

>Nomad

----------------------------------------------------HRRA---H

R-GP--------TEIRL-QLLEK------------YYFPRMSS----------TIRLQ--

-----TSS-MSV-LQTLQV------TR-DTPNKPNL-QPT---------------PIPNY

HVQILHIDI-----F----------AL------EKR----LYLSCID----KFS------

--KFAK-LF-------HLQ----SK-A-SV------HLR-ET--L-VEALHYFT------

--------------APKVLVSDN-ERGLLCP---------TVLNYLRS------------

-----------LDI--DL---YYAPTQKSEVNGQV--ERFHSTFLEIYRCL-------KD

E-LPT----FKPVELVHIAVDRYNTSV---------------HSV----TNRKPADV---

---------------FFD-----RSSRVKLSGF---------------------------

------------------------------------------------------------

---------D-------------FRRQTLEDIKLIEYKQIRG--NMRNKN-------RDE

P--KSY-------------G-----PGDEV--------FV-A----------NKQIKTKE

KARFR-----CEKVQEDNKVTVKT--------------

>Gypsy

----------------------------------------------------HNRA---H

R-AA--------QENIK-QVLRD------------YYFPKMGS----------LAKEV--

-----VAN-CRV-CTQAKY------DR-H-PKKQEL-GET---------------PIPSY

TGEMVHIDI-----F----------ST------DRK----LFLTCID----KFS------

--KYAI-VQ-------PVV----SR-T-IV------DIT-AP--L-LQIINLFP------

--------------NIKTVYCDN-EPAFNSE---------TVTSMLKN------------

----------SFGI--DI---VNAPPLHSSSNGQV--ERFHSTLAEIARCL-------KL

D-KKT----NDTVELILRATIEYNKTV---------------HSV----TRERPIEV---

-------------------------VHPGAH-----------------------------

------------------------------------------------------------

---------E-------------RCLEIKARLVKAQQDSIG----RNNPS-------RQN

---RVF-------------E-----VGERV--------FVKN----------NKRLGNKL

TPLCT-----EQKVQADLGTSVL---------------

>297

---------------------------------------------------HEKLL---H

P-GI--------QKMTK-LFKEN------------HFFPNSQL----------LIQNI--

-----INE-CNI-CNLAKT------EH-R-NTKMPL-KIT---------------PNPEH

CREKFVVDI-----Y----------SS------EGK----HYISCID----IYS------

--KFAT-LE-------QIK----TK-D-WI------ECR-NA--L-MRIFNQLG------

--------------KPKLLKADR-DGAFSSL---------ALKRWLEE------------

-----------EEV--EL---QLNTAKNGVAD--V--ERLHKTINEKIRII-------NS

S-DDE----EVKLSKIETILYTYNQKIK--------------HDT----TGQRPAQI---

---------------F---------LYAGHPIL---------------------------

------------------------------------------------------------

------------------------------DTQKIKEKKIE----KINED-------RRE

F--NI----------------------DTN--------YRKG--------PLQK---GKL

ENPFKPT---KNVEQTDPDHYKITNRNRVTHYYKTQFK

>17.6

---------------------------------------------------HEKLL---H

P-GI--------QKTTK-LFGET------------YYFPNSQL----------LIQNI--

-----INE-CSI-CNLAKT------EH-R-NTDMPT-KTT---------------PKPEH

CREKFMIDI-----Y----------SS------EGK----HYVSCID----IYS------

--KFAT-LE-------EIK----TK-D-WI------ECK-NA--L-MRIFNQLG------

--------------KPKLLKADR-DGAFSSL---------ALKRWLES------------

-----------EEV--EL---QLNTTKTGVAD--I--ERLHKTINEKIRII-------KT

S-DDE----ETKLSKMETVLNIYNHKTK--------------HDT----TGQTPAHI---

---------------F---------LYAGQPIL---------------------------

------------------------------------------------------------

------------------------------DTQQNKENKIN----KINND-------RVE

Y--EV----------------------DTR--------YRKG--------PLQK---GKL

ENPFKPT---KNVEQTDSDHYKITNRNRITHYYKTQFK

>Tv1

---------------------------------------------------HKELL---H

P-GI--------EKTIN-WFKET------------HYFPDYQN----------L------

-----INE-CET-CNIAKT------EH-R-DTKLTF-EIT---------------PEIAN

IREKYVMDF-----Y----------IV------GDK----QFLSCID----IYS------

--KFAS-LI-------EIK----SR-D-WL------ETK-RA--I-LQVFNQMG------

--------------KPIEIKADK-DSAFMCT---------ALQLWLKS------------

-----------EAV--NI---NITTSKNGISD--V--ERFHKTVNEKLRII-------NS

D-SDV----ENKLTKFETILYTYNHKTK--------------HKT----TNRTPADI---

---------------F---------IYAGTPEY---------------------------

------------------------------------------------------------

------------------------------DTQANKEKLIN----NLNKK-------RTN

Y--EI----------------------DTR--------YKHS--------PLVK---SKT

TTPFKKT---GELRQIDDKHFEETNRGRKITHYKTKFK

>Ted

--------------------------------------------------HDGKTN---H

R-GI--------NECYL-ALSKR------------YYWPRMKD----------QITKF--

-----INE-CTI-CGQAKY------DR-N-PIRPQF-NIV---------------PPATK

PLETVHMDL-----F----------TV------QNE----KYITFID----VFT------

--KYGQ-AY-------HLR----DG-T-AI------SIL-QA--L-LRFCTHHG------

--------------LPITIVTDN-GTEFSNQ---------LFSEFVRI------------

-----------HKI--IH---HKTLPHSPSDNGNI--ERFHSTILEHIRIL-------KL

Q-HKD----EPIVNLMPYAIIGYNSSI---------------HSF----TKCRPFDL---

---------------LNG------------------------------------------

----------------------------------------------HFDPRD--------

-----PLDID-------------LTEHILQQYAQNHRQQMKQVYEIINET-------SLA

V--EYI-------------------PQQQV--------FIKN--------PLASR--QKV

APRYT-----QDTVLADLPIHIYTSKK-----------

>Zam

---------------------------------------------------HTNSN---H

R-GI--------DETFL-HLKRE------------TYFPNMKN----------KISEL--

-----IRN-CET-CLKLKY------DR-Q-PQNIVF-ETP---------------ETPSK

PLDIIHIDI-----Y----------TI------NNN----FNLTIID----KFS------

--KFAA-VY-------PIP----NR-N-GI------NCI-KA--I-KNFFSQFG------

--------------LPKKLIHDQ-GVEFCND---------IFRKFCSQ------------

-----------YNI--LL---HVTSFQQSSSNSPV--ERLHSSLTEIYRILLDTRKKHKL

P-TDH----EEI---MSETVITYNNAI---------------HST----TKHTPFEL---

---------------FNG-----RTHLFEKTII---------------------------

------------------PNNEH----------------------DYLNKLN--------

-----TFQDK-------------LYSEIKEKLSTNTQQRI----EKLNTS-------RVE

P--TTV-------------Q-----PNSTI--------FRKE----------NRR--NKL

TPRFS-----LHRT------------------------

>Idefix

---------------------------------------------------HKELL---H

P-GI--------EKTIN-LFKEE------------YYYPDSQK----------LIQTI--

-----INE-CQI-CYLAKT------EH-Q--TQMTY-ETT---------------PEIFN

TREKYMIDF-----Y----------LT------GNQ----IFLSCID----IYS------

--KFAS-LV-------ELK----SR-D-WL------EAK-RA--I-TKIFNDMG------

--------------KPQEIKADK-DSAFMCL---------ALQNWLRS------------

-----------EGV--QI---SISTSKNGISD--I--ERFHKTVNEKLRII-------GS

Q-QNV----EDRCTKFERILYIYNHKTK--------------HNS----TKRFPADI---

---------------F---------LYAGSPDF---------------------------

------------------------------------------------------------

------------------------------NVQQNKIDRIE----YLNKN-------RHD

F--EV----------------------DIK--------YRQA--------PLVK---SKI

TNPFKKT---GRIGQVDDKHFEETNRGRKIVHYKSKFK

>Tom

---------------------------------------------------HEKLL---H

P-GI--------QKMTK-LFKEN------------HYFPNSQL----------LIQNI--

-----INE-CRV-CNLAKT------EH-R-NTKMPF-KVT---------------PSPGH

CRDKFVIDI-----Y----------SS------EGK----HYLSCID----IYS------

--KFAT-LE-------QIK----TK-D-WI------ECK-NA--L-MRIFNQLG------

--------------KPTLLKADR-DGAFSSL---------ALKQWLES------------

-----------EGV--EL---QLNTAKTGVAD--V--ERLHKTINEKIRII-------NS

S-KND----EIKLGKMENILYIYNHKTR--------------HDT----TGQTPAHI---

---------------F---------LYAGQPTL---------------------------

------------------------------------------------------------

------------------------------DAQKIKEQKIN----KLNDD-------RQE

Y--DI----------------------DTK--------FRKG--------PLQK---GKL

ENPFKEN---KNVEQTDPDHYKITNRNRTTNYYKTQFK

>Springer

----------------------------------------------------HNRA---H

R-SA--------QENVK-QVLSE------------YYFPKMTK----------LASEI--

-----AAN-CKT-CAKAKY------DR-H-PKKQEL-GET---------------PVPTH

VGEILHIDI-----F----------ST------DKK----YFLTCVD----KFS------

--KFAM-VQ-------PIL----SR-T-IE------DLK-AP--L-LQLMNVFP------

--------------KAKTIYCDN-EPSLKSQ---------TIVAMLEN------------

----------HFGV--SI---SNAPPLHSVSNGQV--ERFHSTLIELARCL-------KI

D-KGI----SDTVELVLLATARYNMSI---------------HSV----INKKPAEV---

-------------------------MRADPD-----------------------------

------------------------------------------------------------

---------D-------------PHTDVQEKIKNAQILTRK----RENAS-------RQN

---RVF-------------Q-----VGDKV--------LVKS----------NRRLGNKL

TPLCE-----EKTIEADLGTTVL---------------

>Athila41

-----------------------------------------------------------H

G-SAYGGHFATFKTVSK-ILQAG------------FWWPTMFK----------DAQEF--

-----VSK-CDS-CQRKGN-------I-N-RRNEMP-QNP---------------ILEVE

IFDVWGIDF-----MGPF-----PSSY------GNK----YILVAVD----YVS------

--KWVE-AI-------ASP----TN-D-AK------VVL-KL--FKTIIFPRFG------

--------------VPRVVISDG-GKHFINK---------VFENLLKK------------

-----------HGV--K----------------QV--EISNREIKTILEKTV-G----IT

R--------KDWSAKLDDALWAYRTAF---------------KTP----IGTTPFNL---

---------------LYG-----KLCHL--------------------------------

------------------PVELEYKAMWAVKLLNFDIKTAEEKRLIQLSDLD--------

-----EIR-----------------LEAYESSK-IYKERTK---LFHDK--------KII

T--KDF-------------Q-----VGDQV--------L--L-FNSRL-----KLFPGKL

KSRWSGPFCITE-VRPYGAVTLAGK-------------

>Diaspora

-----------------------------------------------------------H

S-SAPGGHLGVQRTARK-VLDCG------------FYWPTIFK----------DAWKI--

-----CST-CEQ-CQRAGN------TL-T-WRQQMP-QQP---------------MLFCE

VFDVWGIDF-----MGPF-----PVSF------GYV----YILLAVD----YVS------

--KWVE-AK-------PTR----TN-D-AK------VVA-DF--VRSNLFCRFG------

--------------VPKAIVSDQ-GTHFCNK---------TMHALLKN------------

-----------YGV--VH---RVSTPYHPQTNGQA--EISNREIKRILEKIV-Q----PS

R--------KDWSTRLDDALWAHRTAY---------------KAP----IGMSPYRV---

---------------VFG-----KACHL--------------------------------

------------------PVEIEHKAYWAVKTCNFSMDQAGEERKLQLSELD--------

-----EIR-----------------LEAYENAK-FYKEKTK---KFHDS--------MIV

K--KDF-------------M-----VGQKV--------L--L-YNSRL-----GLMSGKL

RSKWIGPFVVTN-VFPYGTVEIKSD-------------

>Cyclops2

-----------------------------------------------------------H

N-S-YGGHYNGVRTATK-ILQSG------------FYWPTIFK----------DAHTH--

-----AQS-CDS-CQRSGG-------I-G-KRDEMS-LQN---------------IQEVE

VFDCWGIDF-----VGPF-----PPSY------GNE----YMLVA---------------

----VE-AI-------ASP----RA-D-AK------TVI-IF--LKKNIFSRFG------

--------------TPRVLISDG-GSHFCNA---------PLESILKH------------

-----------YGV--SH---RVATPYHPQANGQA--EVSNREIKRILEKTV-S----NS

K--------KEWSQKLDEALWAYRTAF---------------KAP----IGLTPFQL---

---------------VFG-----KTCHL--------------------------------

------------------PVELEHKALWALKINNFEKDLAGEKRKVQLLELE--------

-----EIH-----------------NAAYHSSC-LYKEKVK---KYHDK--------KLR

K--KEF-------------V-----PGQLV--------L--L-FNSRL-----KLFPGKL

KSKWSGPFRVKE-VNEYGAIVIEDM-------------

>Bagy2

-----------------------------------------------------------H

S-EAYRGHHARDRTAHK-VLQSG------------FYWPTLFK----------DSRKF--

-----VLS-CDE-CQRIGN-------I-S-KHHEMP-MNH---------------SLVIE

PFDVWGFDY-----MGPF-----PSSN------GYT----HILVAVD----YVT------

--KWVE-SI-------PTR----NA-D-HH------TSI-KM--LKEVIFPRFG------

--------------VPRYLMTDG-GSHFIHG---------VFCKMLVK------------

-----------YDV--NH---RVASPYHPQSSGQV--ELSNREIKLILQKTV-N----RS

R--------KNWSRKLDDALWAYRTAY---------------KNP----MGMSPYKM---

---------------VYG-----KACHL--------------------------------

------------------PLELEHKAYWAIKELNFDFKLAGEKRLFDISSLD--------

-----EWR-----------------AQAYENAK-LFKEKVK---RWHDK--------RIQ

K--REF-------------N-----VGDYV--------L--L-YNSCL-----RFFAGKL

LSKWEGLYVVEE-VYRSGAIKINNT-------------

>Calypso

-----------------------------------------------------------H

N-SPCGGHYGGDKTTTK-VLQSG------------FFWPTLFK----------DAHQN--

-----MLH-CDQ-CQRMGG-------I-S-KRNEMP-LQN---------------IMEVE

VFDCWGIDF-----VGPF-----PLSF------GNE----YILVVVD----YVS------

--KWVE-AV-------ATL----HN-D-AK------IVV-KF--LKTNIFSRFG------

--------------VPRVLISDG-STHFCNN---------KIQKVLKQ------------

-----------YNV--TH---KVASAYHPQTNGQA--EVSNKELKKILEKTM-A----ST

R--------KDWSIKLDDALWAYRTAF---------------KTP----IGLSPFQM---

---------------VYG-----KSCHL--------------------------------

------------------PVEMKYKTYWALKLLNFDEAESREQRRLQLLELE--------

-----EIK-----------------LTAYESSQ-LYKEKIK---KYHDK--------KLL

K--RDF-------------Q-----QGQQV--------L--L-FTSRL-----KLFPGKL

KSKWSRPFTIKK-VRTYGAVELCDP-------------

>Gloin

---------------------------------------------------HEGKL-GGH

Y-GV--------FMTMK-RIQQS------------FHWTGLVK----------DVQRF--

-----LVE-CQV-CQTHKT------ST-R-SPAGLL-QPL---------------PIPEK

VWEDLTMDF-----IEGL----PFSN-------GIN----VILVVVD----RLS------

--KYAN-FI-------GLR----HPFT-AA------DVA-SS--FIQEIVRLHG------

------------------YIVDR-DMIFLSN---------FWREYFKQ------------

-----------AGT--KL---KHSTSFHFETDGQI--ELTNRSLEAYLRCLA-S------

--SHP----QTW-KFLAWAELWYNTSF---------------HTS----LKTTPFQV---

---------------VYG------------------------------------------

----------------RPPPTLLKYEEHSTTN---------VDLERLLKDSD--------

-----MML-----------------GRIKDQLT-LTQQLMK---NNANKH-------RGD

V---EF-------------K-----VGDFV--------YLKL-RPYRQHSV-TRRVCQKL

ATKYYGLFEVLE-RIGKAAYRLKLQ-------------

>Ifg7

---------------------------------------------------HTSPL-GGH

S-GF--------SKTYH-KVKKE------------FFWDGLKS----------DIQKF--

-----VVE-CLV-CQN-KV------AT-I-KTPGLL-QPL---------------SIPSQ

CWEDVSMDF-----ITGL----PKSE-------GKS----VIMVVVD----RLT------

--KYAH-FC-------TLS----HPFK-AS------KVS-TA--FMT-IQKLHG------

--------------NPKIIVCDR-NLIFTGN---------FW-TLFSC------------

-----------LGT--LL---AHSSSYHPQSDGKN--EIVNKCFEGYLRCFV-S------

--DK-----TQWVKWLPLAEWWYNTSF---------------HIE----TKMTPFMT---

---------------LYG------------------------------------------

----------------YQQPSITSYL-RISKV---------QAVEHHIEHQQ--------

-----QVL-----------------QLLKDNLV-LAHNRMK---QQADQH-------RIK

R---SF-------------D-----VGDWV--------FLRL-QPYKHMSLKKAKKDNKL

SPKYYGPYKVLQ-KIGTMAYKLELP-------------

>Reina

---------------------------------------------------HDSPI-GGH

S-GF--------PVTYH-RIKKL------------FYWAGMKG----------QIKEF--

-----VQS-CEI-CTKAKA------DR-N-RYPGLL-LPL---------------PIPDQ

AWQVISLDF-----ISGL----PTSR-------RFN----CILVVVD----KFS------

--KYAH-FL-------AMS----HPFT-AL------SVA-KL--FLSQVYKLHG------

--------------LPLSIISDR-DPIFTSN---------LWQELFKL------------

-----------VGT--KL---CLSSAYHPQSDGQT--EWVNQCVEAYLRCFV-H------

--GCP----KQWSNWLSLAEFWYNTCF---------------HTA----LGQSPFEV---

---------------LYG------------------------------------------

----------------HTPSQLGLSTIEQCQS---------ADLQTYLATRQ--------

-----LML-----------------QRAKLHLQ-RAQDRMK---KQADKG-------RSE

R---VF-------------Q-----VGQRV--------FLKL-QPICQSSM-GSRLNTKL

SFRYFGSFLITK-QVNPVAYKLALP-------------

>Gimli

---------------------------------------------------HCSGV-GGH

S-GR--------DATHQ-RVKSL------------FYWKGMSK----------DIQAY--

-----IRS-CSV-CQQCKY------DT-T-ASPGLL-QPL---------------PIPNA

IWSDISMDF-----IDGL----PISF-------GKS----VILVVVD----RLT------

--KAAH-FI-------ALS----HPYS-AL------TVA-QA--FMDNIFKLHG------

--------------LPNSIVSDR-DSVFLSE---------FWRELFTL------------

-----------QGV--AL---NYSSAYHPQSDGQT--EVVNRCLETYLRCMT-S------

--DRP----GLWSRWLPLAEFWYNTSF---------------HSS----ANMSPYEA---

---------------VYG------------------------------------------

----------------QPPPQHLPYVPGESKV---------AVVAQNLQERE--------

-----KML-----------------LILKFHLL-RAQHRML---QSANKK-------RSD

R---SF-------------Q-----IGDFV--------FVKL-QPHRQGSV-VMRSNQKL

APKYYGPYKILD-TCGKVAYKLALP-------------

>Monkey

---------------------------------------------------HDSLW-AGH

P-GI--------HRTLA-LVERA------------FYWPKMGI----------NVEEY--

-----VRT-CLT-CQ-DKV------EQ-R-KPVGLL-EPL---------------PVPER

PWERISLDF-----ISSL----PPVG-------GLG----SILVVVD----RFS------

--KYAT-FI-------AAP----LHCS-AE------EAA-RL--MMKDVVKYWG------

--------------VPHNIISDQ-DARFLGR---------FWTELF-L------------

-----------LGS--KL---YFSTSLHPQMDG-T--ERINSLLEQYLRHYV-S------

--ANQ----RNWVKLLDIAQFSYNLQR---------------SST----SNKSPFEI---

---------------ITG-----QQP----------------------------------

----------------STPHTMAIGYT-GSS----------PSAYHFAKEWH--------

-----RNA-----------------DIARAYLE-KATKRMK---K-ANLG-------RRP

Q---EF-------------K-----VGDLV--------LVKL-SASL--QFFRNRVHKGL

VRKYEGPFPIIS-RVGNVSYKLQLP-------------

>Tntom1

---------------------------------------------------HDTLR-VGH

P-GE--------ECTMA-LLCRA------------YYWPQVAD----------DVTQY--

-----VKT-CLV-CQKDKS------DR-L-TQAP-V-EYL---------------NVPKR

PWESVSLDF-----ITGL----PKVG-------DLT----TILIVVD----RFS------

--KYAT-FI-------ASP----QYIL-AE------ELA-RL--FFSHVVKYWG------

--------------QPKDIVSDR-GSCFTSN---------FWTQLFKC------------

-----------LGS--KL---SHSSSFHPQSDVQT--ERFNGMLEEYICHFV-T------

--GSQ----KDWLKLLDAAQLCFNSQT---------------ISS----TNKSAFEI---

---------------ITG-----QQL----------------------------------

----------------LLPRTVNAPNM-SKS----------PAA-SFSKEWK--------

-----RNL-----------------EIVQSYL--VAQKRMK---RHADQN-------RRF

V---EY-------------Q-----VGNKV--------MVKI---PMW-YLFAGVHDPRL

LQKNIGPLSIER-HIGKVAYRVDNP-------------

>Galadriel

---------------------------------------------------YDSAW-AGH

P-GV--------ERMLA-LLSRV------------YFWPKMED----------DIEAY--

-----VKT-CHV-CQVDKT------ER-K-KEAGLL-QPL---------------PIPER

PWLSVSMDF-----ISGF----PKVD-------GKA----SIMVVVD----RFS------

--KYSV-FI-------AAP----ELCS-SE------VAA-EL--FYKHVIKYFG------

--------------VPADIVSDR-DTRFTGR---------FWTALFNM------------

-----------MGT--EL---KFSTANHPQTDGQT--ERINHLLEEYLRHYV-T------

--ASQ----RNWVELLDTAQFCYNLHK---------------SSA----TEMSPFEI---

---------------VLG-----KQP----------------------------------

----------------MTPLDVAKSKNQGKC----------PAAYRVARDRL--------

-----EML-----------------SEAQDSLR-KAQQRMK---KYADQH-------RRS

V---EF-------------S-----VGDKV--------LLKL-TPQIWKQIVSKTRHRGL

IPKYDGPFEVVK-RVGEVAYRLKLP-------------

>Del

---------------------------------------------------HRSKF-TIH

P-GS--------TKMYR-NLKIN------------FWWSGIKR----------EVVEY--

-----VSR-CLI-CQQVKA------DH-H-HHSGLL-QPL---------------PVSE-

KWEHILMDF-----IIGF----PLSKR------CHD----SIWVIVD----RFT------

--KSAH-FI-------PIH----TTIS-GK------DLA--L--YIKEIIRLHG------

--------------IPTTIVTDR-DTKFTSR---------FWGSL-KS------------

-----------LGT--EL---FFSTAFHPQTDG-S--ERTIQILEDMLRSCS-L------

--DFK----GNWEEHLPLVEFAYNNSY---------------QSS----IGMAPFEA---

---------------LYG------------------------------------------

----------------RPCRSPTCWAEIGEHH---------LIRPELIQQTT--------

-----NAI-----------------EVIKRRLK-AAQDRQK---SYTDIR-------RHP

L---EF-------------S-----VGNHI--------FLEV-SPRKGTS--YFVFKGKL

SPRYTGPFEILE-IIWPVAYRLALP-------------

>Peabody

---------------------------------------------------HRSGL-SIH

P-GA--------TKMYH-DLKKL------------FWWPGMKR----------EIASF--

-----VYS-CLT-CQKSKI------EH-Q-KPSGLI-QPL---------------AIPEW

KWDSISMDF-----VSGL----PMTIK------NFE----AIWVIVD----RLT------

--KSAH-FI-------PIR----MDYP-LE------RLA-EL--YIEKIVSLHG------

--------------IPSSIVSDR-DPRFTSK---------FWEGLHKA------------

-----------LGT--KL---RLSSAYHPQTDGQT--ERTIQSLEDLLRACV-L------

--EKG----GAWDCYLPLIEFTYNNSF---------------HSS----IGMAPFEA---

---------------LYG------------------------------------------

----------------RRCRTPLCWYESGESA---------VVGPEIVQQTT--------

-----EKI-----------------KMIQEKMR-IAQSRQK---SYHDKR-------RKS

L---EF-------------Q-----EGDHV--------FLRV-TPITGVG--RALKSKKL

TPRFIGPYQILE-RIGEVAYRIALP-------------

>Retrosat2

---------------------------------------------------HQTQY-SIH

P-GS--------TKMYQ-DLKEK------------FWWVSMRR----------EIAEF--

-----VAL-CDV-CQRVKA------EH-Q-RPAGLL-QPL---------------QIPEW

KWEEIGMDF-----ITGL----PRTSS------GHD----SIWVVVD----RLT------

--KVAH-FI-------PVQ----TTYT-GK------RLA-EL--YLARIMCLHG------

--------------VPRKIVSDR-GSQFTSK---------FWQKLQEE------------

-----------MGT--RL---NFSTAYHPQTDGQT--ERVNQILEDMLRACA-L------

--DFG----GAWDKSLPYAEFSYNNSY---------------QAS----LQMAPFKA---

---------------LYG------------------------------------------

----------------RKCRTPLFWDQTGERQ---------LFGTEVLAEAE--------

-----EKV-----------------RIIRERLR-IAQSRQK---SYADNR-------RRE

L---TF-------------E-----AGDYV--------YLRV-TPLRGVH--RFQTKGKL

APRFVGPYKILE-RRGEVAYQLELP-------------

>Bagy1

---------------------------------------------------HDSTL-TIH

P-RS--------TKMYQ-DLRQR------------FWWTRMKR----------EIAEF--

-----VAN-CDV-CRRVKA------EH-Q-RPAGTL-QPL---------------AIPEW

KWDKVSMDF-----ITGF----PKTKK------GNN----AIFVVID----RLS------

--KVAH-FL-------LVR----ESII-AS------QLA-EL--YVSRIVFLHG------

--------------VPLGINSDR-GSIFTSR---------FWESFQNA------------

-----------MGT--HL---SFSTAFHAQSSGQV--ERVNQILEDML-ACV-I------

--SFG----MNWEKCLPFAEFAYNNSY---------------QSS----LGKAPFEV---

---------------LYG------------------------------------------

----------------RRCRTPLNWSETGERQ---------LFGPDMIQDAE--------

-----E-V-----------------RIIREKLK-TAQSLQK---SQYDRH-------HKA

V---TS-------------E-----VDEKA--------YLRV-TPLKGTH--RFGIKGKL

APRYIGPFRILA-KRGVVAYQLEHP-------------

>Tma

---------------------------------------------------HASMF-SIH

P-GA--------TNMYR-DLKR-------------YYWVGMKR----------DVANW--

-----VAE-CDV-CQLVKA------EH-Q-VPGGML-QSL---------------PIPEW

KWDFITIDF-----VVGL----PVSR-------TKD----AIWVIVD----RLT------

--KSAH-FL-------AIR----KTDG-AA------VLA-KK--YVSEIVKLHG------

--------------VPVSIVSDR-DSKFTSA---------FWRAFQAE------------

-----------MGT--KV---QMSTAYHPQTYGQS--ERTIQTLEDMLRMCV-L------

--DWG----GHWADHLSLVEFAYNNSY---------------PAS----IGMAPFEA---

---------------LYE------------------------------------------

----------------RPCRTPLCLTQVGERS---------IYGADYVQETT--------

-----ERI-----------------RVLKLNMK-EAQDRQR---SYADKR-------RRE

L---EF-------------E-----VGDRV--------YLKM-AMLRGPN--RSISETKL

SPRYMGPFRIVE-RVGPVAYRLELP-------------

>Legolas

---------------------------------------------------HQSKF-SIH

P-GS--------NKMYR-DLKRY------------YHWVGMKK----------DVARW--

-----VAK-CPT-CQLVKA------EH-Q-VPSGLL-QNL---------------PIPEW

KWDHITMDF-----VTGL----PTGIKS-----KHN----AVWVVVD----RLT------

--KSAH-FM-------AIS----DKDG-AE------IIA-EK--YIDEIVRLHG------

--------------IPVSIVSDR-DTRFTSK---------FWKAFQKA------------

-----------LGT--RV---NLSTAYHPQTDGQS--ERTIQTLEDMLRACV-L------

--DWG----GNWEKYLRLVEFAYNNSF---------------QAS----IGMSPYEA---

---------------LYG------------------------------------------

----------------RACRTPLCWTPVGERR---------LFGPTIVDETT--------

-----ERM-----------------KFLKIKLK-EAQDRQK---SYANKR-------RKE

L---EF-------------Q-----VGDLV--------YLKA-MTYKGAG--RFTSRKKL

SPRYVGPYKVIE-RVGAVAYKLDLP-------------

>Cereba

---------------------------------------------------HRGMG-LGH

F-GV--------KKTED-VLATH------------FFWPKMRR----------DVERF--

-----VAR-CTT-C-KAKS------RL-N--PHGLY-MPL---------------PVPSV

PWEDISMDF-----VLGL----PRTKK------GRD----SIFVVVD----RF-------

--KMAHFFI-------PCH----KSDD-AA------DVD-DL--FFHEIIRLHG------

--------------VPNTIVSYR-DAKFLSH---------FWRHLWAK------------

-----------LGT--KL---LFSTTCHPQTNGQT--EVVNRSLSTMLRAVL-K------

--NNI----KLREECLPHIDFAYNRSL---------------HST----TKMCPFEV---

---------------VYG-----FLP----------------------------------

----------------RAPIHLL-PLPSSEKV--------NFDAKECADLIL--------

-----NMH-----------------ELTKENIE-CMNSKYK---LAGAHK-------K--

--------------------------------------WSS-------------------

--------------------------------------

>CRM

---------------------------------------------------HGGGL-MGH

F-GA--------KKTED-ILAGH------------FFWPKMRR----------DVVRL--

-----VAR-CTT-CQKAKS------RL-N--PHGLY-LPL---------------PVPSA

PWEDISMDF-----VLGL----PRTRK------GRD----SVFVVVD----RFS------

--KMAH-FI-------PCH----KTDD-AT------HIA-DL--FFREIVRLHG------

--------------VPNTIVSDR-DAKFLSH---------FWRTLWAK------------

-----------LGT--KL---LFSTTCHPQTDGQT--EVVNRTLSTMLRAVL-K------

--KNI----KMWEDCLPHIEFAYNRSL---------------HST----TKMCPFQI---

---------------VYG-----LLP----------------------------------

----------------RAPIDLM-PLPSSEKL--------NFDATRRAELML--------

-----KLH-----------------ETTKENIE-RMNARYK---FASDKG-------RKE

I---NF-------------E-----PGDLV--------WLHL-RKERF----PELRKSKL

LPRADGPFKVLE-KINDNAYRLDLP-------------

>Beetle1

---------------------------------------------------HGGGL-AGH

F-GI--------NKTVD-VLQEH------------FYWPKLAG----------DVHSV--

-----LAR-CST-CQKAKS------SF-H---QGLY-TPL---------------PVPNQ

PWEDVSMDF-----IVAL----PRTQR------QRD----AIMVVVD----RFS------

--KMAH-FI-------ACN----KTDD-AI------HVA-DL--YFKEIIRLHG------

--------------VPKTIVSDK-DVKFLSF---------FWKTLWKL------------

-----------LGT--KL---LFSTTAHPQTDGQT--EVTNRTLTTLLRTLVNK------

--KSQ----KDWDLKLAHAEFAYNRSP---------------TYA----TKCSPFEV---

---------------NYG-----VNP----------------------------------

----------------LSPIDLL-PIPVEVRQ--------SKDAEEKAKEMK--------

-----KLH-----------------QQIRARIE-KVNEAYK---VQANKH-------RKE

V---IY-------------K-----PGDLV--------WLHL-RKERF----PSRRKNKL

MPRSDGPFRVLE-KINNNAYKIELP-------------

>REM1

---------------------------------------------------HDRR----H

FHGP--------------------------------------------------------

--KLVAAT-CAL------------------------------------------------

----------------PA---------------GNAR---HGLVC---------------

-----------------------------------WIVA---------------------

--------------NPLCESTDL-------------------------------------

-----------VGIH---------------------------------------------

----------------HLVEFAINNSK---------------NRS----TGFSPFALNC-

----------------------AKQPRV--------------------------------

------------------PADLSYKS-------------RCPSADAYAKEMQ--------

-----SRL-----------------QRAREALK-RAQDRQK---ADADK--------RRR

P--VSY-------------E-----VGQDV--------LLST-ANIKF----KGVGTKKL

APKWVGPYKIVE-LIGPAAVRLALP-------------

>GRhodo

---------------------------------------------------HNAPL-GGH

F-GM--------DKTLH-ALRQT------------YIWPSMRH----------HVEHY--

-----VKS-CDA-CQKNKA------RH-H-KNFGTP-QIP---------------DIPSK

PWEWMSVDF-----CGTF----PKTKE------GND----YIAGFIC----NLV------

--REAI-LV-------PCT----KNVT-AK------QTV-KL--FVKYVMPRTG------

--------------IPERINSDR-GPQFISN---------FWKNLWKL------------

-----------LGT--EL---AFSAPYHANSNALI--ERQNKTFIENLKSYI-N------

--ARQ----DDWEDHIGPYEFAYNNSY---------------NKS----IGDTPFFL---

---------------SHG-----RQP----------------------------------

----------------AMPVASLHKTPS-------------PAAEDFIMNLQ--------

-----NRI-----------------AMARDHIR-QRQGERA---DRNVDS-------TQP

A---NY-------------K-----VGDKV--------LLST-EHYNL-----QLPSEKL

APRWLGPLEILE-VRGPNTVRIEVP-------------

>Sushiichi

---------------------------------------------------HSSCF-ACH

P-GV--------RRTAE-FVQRR------------FWWPNLQE----------DVREF--

-----VGA-CTV-CARSKA------SH-R-SPAGLL-HPL---------------PVPSR

PWSHVALDF-----VTGL----PVSQ-------GND----TILTIVD----RFS------

--KGVH-FV-------ALP----KLPS-AA------ETA-EL--LVSHVVRLHG------

--------------IPLDVVSDR-GPQFTSR---------VWQAFCKG------------

-----------IGA--TV---SLSSGYHPQSNGQA--ERANQAMEAALRCVT-T------

--SNP----ASWSKFLPWVEYSLNAME---------------SSA----TGMSPFQC---

---------------FLG-----YQPPL--------------------------------

----------------FPQQELEIAVPS---------------TRAHLRRCR--------

-----RIW-----------------KTARKAIL-RATEQSR---RSANRR-------RRP

A--PAY-------------R-----PGQKV--------WLLA-RDLPLQT--SQTSSRKL

NPRDIGPYTICS-IINPSAVHLDLP-------------

>Amnichi

---------------------------------------------------HDCKP-AGH

F-GL--------FKTMH-LILRD------------FWWPKIRK----------DVEKY--

-----VNT-CPV-CQRSKT------RR-E-KPSGLL-HPL---------------PTPSR

PWEIISADF-----ITDL----PPSC-------GFT----TILVVVD----LFT------

--KLAH-FI-------PCE----GLPT-AK------ETA-DL--FLQHVFRLHG------

--------------LPKSLVTDR-GSQFTSR---------FWKALQKL------------

-----------LGI--DS---RLSSAHHPQTDGQT--ERTNATLEQYLRCYV-N------

--YQQ----DNWASLLPLSEFAYNNGV---------------QAS----TKETPFFA---

---------------NYG-----FHPRF--------------------------------

----------------FPPVIETSEVPA---------------AEDWLQELT--------

-----AVQ-----------------QLLLQQLD-QAKEDYK---RHADKH-------RQP

G--PEI-------------K-----VGDRV--------FLST-RFLPS-----HRPCRKL

DARFIGPYPVVA-QLNPVTFKLQLP-------------

>Amnsan

---------------------------------------------------HSSKQ-AGH

P-GS--------EKTLE-LLRRL------------VWWPTIRK----------DVRDF--

-----VAA-CTV-CATTKA------SH-S-RPCGLL-HPL---------------PIPSR

PWTHLGMDF-----IVEL----PPSC-------GNT----VIWVVID----RFS------

--KMAH-FI-------PLR----KLPS-AV------ELA-HL--FIQHIFRLHG------

--------------FPVEIVSDR-GSQFVSR---------FWRSLCKS------------

-----------LGV--SL---QFSSAYHPQTNGAA--ERVNQALEQFLRNHV-S------

--LCQ----DDWSDLLPWAEFAHNNAS---------------HSS----TGRSPFLS---

---------------VYG-----QHPLA--------------------------------

----------------FPQDLLLSEVPA---------------ADDLAAHMS--------

-----VIW-----------------AATKSNLE-KSSLVHK---TFADRR-------RKP

S--PPY-------------K-----VGEKV--------WLSS-RNIRL-----KVPSPKL

GPKFLGPFSISE-VINPVAVRLQLP-------------

>Amnni

---------------------------------------------------HSSQL-TCH

P-GS--------VRTLE-FLQRR------------FWWPTIKR----------DVTMY--

-----VKA-CPT-CNQNKS------SH-C-APQGLL-HPL---------------SIPHR

PWSHISMDF-----ITGL----PSSQ-------GNT----TILVIVD----RFS------

--KAAR-FI-------PLS----KLPT-AK------ETA-EL--VINHVFRVFG------

--------------IPLDVVSDR-GPQFSSR---------FWQAFCQS------------

-----------IGA--TA---SLSSGFHPESNGQT--ERLNQDLETTLRCMA-A------

--NNP----TAWSRFIIWAEYAHNTLR---------------SSA----TGMSPFEC---

---------------QFG-----FPPPL--------------------------------

----------------FPEQEVEVAVPS---------------ALQFVRRCR--------

-----QTW-----------------RKARLKLL-KVSQQYK---HQANRR-------RRP

A--PTL-------------R-----PGQRV--------WLST-RNIPL-----RVDSRKL

SQKFIGPFKIAR-KVNPVTYKLYLP-------------

>Dane1

---------------------------------------------------HDKPT-SGH

P-GR--------ACTYE-LLARE------------YYWPNM-Y----------YVSQW--

-----IKN-CHT-CQRITP------SY-K-ARQGIL-RPL---------------PVPEH

AWQDISMDF-----ITHL----PTSQ-------GFD----SILVVVD----RLT------

--KMRH-LI-------ACQ----ATIN-AE------LVA-HL--YTQHVWRLHG------

--------------LPRTITSDR-GPQFVAE---------FWKHLNKH------------

-----------LDI--QS---LLSTAFHPETNGQT--EWVNAMLEQYLQAYI-T------

--YLQ----DNWSTWLPLAEFALNATY---------------SEA----IRTSLFFA---

---------------NYR-----FHPRM--------------------------------

---------------GFEPVPVPD-CPAS------------QDAENFAQKMQ--------

-----AIS-----------------DYVRSQMT-SAQARYE---EQSNKT-------RQP

A--R-----------------------QYKAVSV--CGWMPA-TSV-------PRPSKKL

-IGSLGPFTIKG-IINAHTYELDLP-------------

>Maggy

---------------------------------------------------HDSPV-AGH

P-GK--------AKTYD-LLSRE------------YYWPGMLH----------YVSLW--

-----VKK-CQT-CRRINP------SR-E-GHQGLL-RPL---------------PTPER

SWQHLSMDF-----ITHL----PQSN-------GHD----AILVVVD----RLT------

--KMRH-FV-------PCK----GTCN-AE------DTA-NL--YLHHVWKLHG------

--------------LPLTIVSDR-GTQFVSK---------FWKHLTTR------------

-----------LKI--DS---LLSTAHHPETDGQT--ERFNASLEQYLRAYV-A------

--YLQ----DDWESWLPLAEFTANSHK---------------SET----TGTSPFYA---

---------------TYG-----FHPRM--------------------------------

---------------GFEPVPLNQPLPAQ------------RDAEKLAARME--------

-----AIL-----------------EQARAEMT-AAQARYE---EQANRH-------RTP

A--RRL-------------T-----VGQYV--------WLDA-RNIQT-----ARPQKKL

DWKNLGPFRISE-VISPYAYRLDLP-------------

>marY1

---------------------------------------------------HDSQI-AGH

P-GR--------WKTLE-LTSRN------------YWWPQMSR----------LIGQY--

-----CRT-CDL-CLCTKV------PR-R-KPIGEL-HPL---------------PVPES

RWDVVSVDF-----VVEL----PESN-------GFD----AVMCTVD----SVG------

--KRAH-FI-------PTH----TTVS-AL------GAA-RL--YLHHVWKLHG------

--------------LPGAFLSDR-GPQFMAE---------FTRELYRL------------

-----------LGI--KL---LASTAYHPQTDGQT--ERVNQELEQYIRLFV-N------

--ERQ----DDWDDLLPLAEFGYNNHV---------------HAS----TQQTPFLL---

---------------DTG-----RHPRM--------------------------------

---------------GFEPRQTPSHIET---------------VNEFTERMR--------

-----DSL-----------------EEARAALA-KAKDDMA---RFYNQR-------HSP

T--PQY-------------K-----VGDRV--------YLDS-SDIST-----TRPSKKL

AHRFLGPFPIVK-CVGTHAYRLRLP-------------

>Cgret

---------------------------------------------------HSLPA-HGH

Q-GV--------TKTWK-RLRQQ------------YGERVTRE----------RVAIA--

-----IKD-CEV-CLKSKP------AR-H-QPYGLL-QPL---------------PVPQT

AWHSISLDF-----IVKL----PKSREPLT-GVHFD----SVLVIVD----RLT------

--KYAY-FI-------PYK----ESSN-AE------EFA-YT--FLKYIIANHG------

--------------TPKEIVSDR-DKIFTSN---------FWKSITAQ------------

-----------LGI--KQ---AMSTAFHPQTDGQT--ERTNQILETYLRAYV-N------

--YDQ----DNWVVLLPIAQFAYNSAV---------------GES----TKESPFYL---

---------------NYG------------------------------------------

----------------FEPTAY--GEPRAGPE--------AVKAVASVKQIK--------

-----DIQ-----------------ANARRELE-FVRKRMT---HFSNQR-------RIE

G--PTL-------------R-----EGDSA--------YLIR-RNIKT-----KRPSDKL

DYKKLGPFKILK-QISPVNFKLDLP-------------

>Cft1

---------------------------------------------------HDEPT-YGH

P-GT--------SKTVD-LIQRS------------FSFPQMRL----------KVLRY--

-----IKK-CVH-CQQNKA------AR-H-AKYGHL-QFR---------------TPPTK

PWDEVTMDF-----ITKL----PRSKDRVT-GQAYD----MILVMVD----RLT------

--KYAH-FI-------PAS----EIYT-AE------QLG-YL--VLDRLIRYHG------

--------------FPEVFITDR-DKLFTSN---------YWKTLMGT------------

-----------IGI--KH---KLSTAYHPETDGQT--ERTNQTLEQYLRHYI-N------

--YAQ----DNWVSLLPMAQIALNNHK---------------SET----TSTTPFMR---

---------------TLAR-T-LTYPEH--------------------------------

----------------LDPT----HRPR--------------EQSVTTETLK--------

-----EVH-----------------KEARKAIE-DAQQRLS---QRRQDE-------RKM

A--PLL-------------K-----EGDKV--------YLLT-KNLKT-----RRQTKKL

DHVKVGPFFIDK-VVGPVNYRLRLP-------------

>Pyret

---------------------------------------------------HGNKA-HGH

Q-GI--------SKTWK-RLKQH------------YNFKRTRQ----------KIRKT--

-----IKD-CEL-CTKSKS------AK-H-KFYGLL-QPL---------------PAPSK

VWQTITMDF-----IVKL----PLSEKPFT-KTKYD----SILVIMD----KFT------

--KYAY-FL-------PYK----KSSN-AE------EIAYYT--FLQIIVSNYG------

--------------LPKNIITNR-DKLFISR---------FWKSLMEQ------------

-----------LGT--NH---KLSTAFHPQTDG-T--KRTNQTLEQYLKYYV-N------

--H-K----DNWVRLLPTAQFVYNSLK---------------NEN----TKTTPFYA---

---------------NYG------------------------------------------

----------------FNPTAY--GEPRTTIT--------APRADKQASELR--------

-----QLY-----------------KKLQQELK-FVRKRIM---KYANH--------RIK

G--PSF-------------K-----KGNSV--------YLIR-RNIKT-----QRPNNKF

DFKKLGPFKISK-KISDTNYRLSLP-------------

>Skippy

---------------------------------------------------HAHPL-HGH

Q-GV--------TKTMK-RL-QE------------LGYRHFKKG---------QVEKV--

-----IKQ-CDL-CAKTKA------QR-H-KPYGQL-QPL---------------PVAQR

PWDSITMDF-----ITKL----PLSEEPST-GIFYD----SIMVIVD----RLT------

--KFSY-YL-------PYR----EATD-AE------ELS-YV--FYRHIVSIHG------

--------------LPTEILSDR-GPTFAAT---------FWQSLMAR------------

-----------LGL--NH---RLTTAFRPQVDGQT--ERMNQVLEQYLRCYI-N------

--YEQ----NDWVEKLPIAQLAYNTAY---------------NES----TKLTPAYA---

---------------NFG------------------------------------------

----------------FTPNAYHNARPEKSIN---------PAAIIKSEDMQ--------

-----DLH-----------------EYLKTELE-FVRKRMK---NYYDPK-------RLK

G--PTF-------------S-----EGDMV--------YLAT-KNIKT-----DRPSHKL

DYKFIGPYKVLQ-KISENNYKLDLP-------------

>Real

---------------------------------------------------HDSLA-TGH

P-GR--------EVTYK-ILARD------------YFWPGMTQ----------TIRRY--

-----VRN-CST-CGRSKS------WR-E-GKQGLL-KPL---------------PIPAQ

IWKEISMDF-----VEGL----PESE-------GMT----NLMVITD----RLS------

--KGTI-FV-------PLP----N-IK-TD------TVV-QK--FIERVVAYHW------

--------------LPDAITSDR-GRQFVSV---------LWTKLCEL------------

-----------LKI--NR---RLSTAYHPQTDGAT--ERMNSVWETYIRSFT-N------

--WAQ----NDWALLCPMAQIAINGRT---------------ATS----TSMS-------

-------------------------PE---------------------------------

----------------VNPLQIE-PEVGSRASNQTEGLSDVQKAQVIASKLQ--------

-----QAI-----------------ELAQASMA-ESQQEQE---RQANKT-------RRE

A--QNF-------------R-----VGDKV--------WLKLDQQYST-----GRSSKKL

DWKN-AKYTVIR-VVDSHSVELDTP-------------

>MGLR3

---------------------------------------------------HGTIA-TAH

P-GR--------NKTRR-LVAQQ------------FWWPGMSG----------MVDRY--

-----VAN-CS--CRSAKV------PR-D-KTPGLL-QPL---------------PVPDR

QWSTIVVDF-----K-SM----PKSKS------GND----NLFVMID----ALT------

--KRSW-AV-------PCT----RTAT-AK------DAA-MM--YYEGPYRIYG------

--------------LPTKVVSDR-GPQFVSD---------LIDEMSKI------------

-----------LQI--KW---KLSTAGHSQTAGQA--EIMNAYIDQRLRPHI-N------

--HFQ----DDWDKRMPAIDLVQATLP---------------HDSL---GGFSPFEI-GN

GYPAHMHFDWTQRTELKGLPTRERLTRT--------------------------------

------------------------------------------EAQAITKKLE--------

-----SYV-----------------EAARTHLQ-MAQQRMC---DQANK--------HRR

E--PDF-------------G-----VGSAVY-------IIKK-------HWVTDRPSDKL

DYPLTRCSYVIK-EKRGHSYRLELP-------------

>Grasshopper

---------------------------------------------------HESPM-VGH

S-GR--------DGTFA-ILARD------------YHWDGMAE----------HVRRF--

-----VRN-CDI-CRRTKP------SR-R-ARQGLL-QPL---------------PIPDR

FWKQISIDF-----MTDL----PGNG-------EVTP--RYLMVITD----RLS------

--KYVQ-LE-------AMH----S-MK-AE------DCA-AR--FLSSWWRFRG------

--------------FPSQIISDR-GSDWVGG---------FWTELCRQ------------

-----------TGV--EQ---LLSTSYHPETDGGT--ERANQEVQQYLRAYI-A------

--FDQ----GDWPDHLGAAQLALNNRN---------------SSV----TGTSPNKL---

---------------LLGFDI-EAVPN---------------------------------

----------------AAPPSKA-PASSPKA-----------RATRFLEHLR--------

-----EGS-----------------ELAQAAIA-YNQQRQE---AGANES-------RRP

A--ERF-------------R-----VGDEV--------FLNL-RNIRT-----NRPCRKL

DYIY-GKYRVVA-VPTPLTVTLDVP-------------

>Pyggy

---------------------------------------------------HESLS-SGH

TRGE--------RLPIKDNLPRR------------FFWAEHDR----------SIRRY--

-----VRN-CDV-CEKT-N------YR-E----KVL-RTIT-------------NTRPED

IEKEISIDF-----IKGL----PTSE-------GIT----YLIVVTN----RLS------

--KGSI-FI-------LLP----N-IK-TE------TVV-RA--FL-RVVAYYL------

--------------L-EAITSDR-G-SFVSV---------L-ERLCEI------------

-----------LKI--RR---RLLISFY-LTNGST--ERINSV-EAYTRAFI-S------

---AQ----ID-ASLCSIAQIAINSRD---------------ATL----TGVAL------

------------------FFL-QHRYN---------------------------------

----------------VDPLQLEIPQ----------------------------------

------------------------------------------------------------

------------------------------------------------------------

--------------------EAD---------------

>Tse3

---------------------------------------------------HDAAA-ASH

P-GQ--------HVHFK-ALLEN------------YYWPGMEN----------DIKRY--

-----VST-CRA-CQQNKH------PV-L-LSPGTF-HPL---------------PSGTH

RWSHINIDF-----LGGL----VLSQ-------GHD----CIMVVID----RAT------

--KMAH-FI-------PVT----KGAD-SE------TVI-DL--FIDSVLKLHG------

--------------FPVEILSDQ-DKLFTSK---------LWQRSMQR------------

-----------FKI--AT---KFTSTYNPSTDGQV--ERMNRTIMEMLRHYL-T------

--DNP----SAWVMLLPVVEFAYNNTY---------------QVS----IQTTPFFA---

---------------NYG-----YHP----------------------------------

----------------RLPGFYHLITSGGQSEKEARG-TELGDLDDRIIQQN--------

-----NIF-----------------LIIQERIA-AAQQKQA---LQYNKK-------HRH

A---EF-------------E-----VGDKVLVHQ--QAYWPG-----------YHKGLKL

HHIWYGPFPVTA-ADGA-NLTLDLP-------------

>Ty31

----------------------------------------------------HTLF-GGH

F-GV--------TVTLA-KISPI------------YYWPKLQH----------SIIQY--

-----IRT-CVQ-CQLIKS------HR-P-RLHGLL-QPL---------------PIAEG

RWLDISMDF-----VTGL----PPTSN------NLN----MILVVVD----RFS------

--KRAH-FI-------ATR----KTLD-AT------QLI-DL--LFRYIFSYHG------

--------------FPRTITSDR-DVRMTAD---------KYQELTKR------------

-----------LGI--KS---TMSSANHPQTDGQS--ERTIQTLNRLLRAYA-S------

--TNI----QNWHVYLPQIEFVYNSTP---------------TRT----LGKSPFEI---

---------------DLG------------------------------------------

----------------YLPNTPAIKSDDEVNAR-------SFTAVELAKHLK--------

-----ALT-----------------IQTKEQLE-HAQIEME---TNNNQR-------RKP

L---LL-------------N-----IGDHVLVHR--DAYFK------------KGAYMKV

QQIYVGPFRVVK-KINDNAYELDLN-------------

>Skipper

---------------------------------------------------HDTKY-SGH

H-AL--------DITYN-NIRQD------------YYFKEMFS----------IIKRY--

-----IKS-CAT-CQLN--------IN-R-KDNGIL-QSL---------------EIPFE

VWRDISIDF-----LS-L----PKTMYAIN---GFTVEVDQVCVIVC----RLS------

--KMVH-IV-------PCH----KTID-AQ------HTA-QL--LLNHVFRLHG------

--------------YPRTIVSDR-DPRFLSE---------IWERWAKT------------

-----------MDS--KL---KMTVAHRAQADGQT--ERMNREIIRILTKAS-T------

--EYG----ENWSDIIPLIEFAMNSSM---------------SKS----TKMSPFQI---

---------------VYG------------------------------------------

----------------FNPPTPVNHFNSLTKTRIP---------------MS--------

-----NIK-----------------KIVRDNIL-DAQINAP---KYYNRG-------RGD

V---IF-------------V-----VGEKVMVKRK---FFQT-------NLSKDLISHKL

ESKNCGPF-IIT-AVHGNNVTLDLV-------------

>TF2

---------------------------------------------------HEEGK-LIH

P-GI--------ELLTN-IILRR------------FTWKGIRK----------QIQEY--

-----VQN-CHT-CQINKS------RN-H-KPYGPL-QPI---------------PPSER

PWESLSMDF-----ITAL----PESS-------GYN----ALFVVVD----RFS------

--KMAI-LV-------PCT----KSIT-AE------QTA-RM--FDQRVIAYFG------

--------------NPKEIIADN-DHIFTSQ---------TWKDFAHK------------

-----------YNF--VM---KFSLPYRPQTDGQT--ERTNQTVEKLLRCVC-S------

--THP----NTWVDHISLVQQSYNNAI---------------HSA----TQMTPFEI---

---------------VHR-----YSPA---------------------------------

----------------LSPLELP---------------SFSDKTDENSQETI--------

-----QVF-----------------QTVKEHLN-TNNIKMK---KYFDMK-------IQE

I--EEF-------------Q-----PGDLVMVKRTKTGFLHK--------------SNKL

APSFAGPFYVLQ-KSGPNNYELDLP-------------

>TF1

---------------------------------------------------HEEGK-LIH

P-GI--------ELLTN-TILRR------------FTWKGLRK----------QIQEY--

-----VQN-CHT-CQINKS------RN-H-KPYGPL-HPI---------------PPPER

PWESLSMDF-----ITAL----PESS-------GYN----ALLVVVD----RFS------

--KMAI-LL-------PCT----KSIT-AE------QTA-RM--FDQRVIAYFG------

--------------NPKEIIADN-DHIFTSQ---------TWKDFAHK------------

-----------YNF--VM---KFSLPYRPQTDGQT--ERTNQTVEKLLRCVC-S------

--THP----NTWVDHISLVQQSYNNAI---------------HSA----TQMTPFEI---

---------------VHR-----YSPA---------------------------------

----------------LSPLELP---------------SFSDKTDENSQETI--------

-----QVF-----------------QTVKEHLN-TNNIKMK---KYFDMK-------IQE

T--EEF-------------Q-----PGDLVMVKRTKTGFLHK--------------SNKL

APSFAGPFYVLQ-KSGPNNYELDLP-------------

>Tor4a

---------------------------------------------------------YCH

T-GI--------DQTFS-KIAKK------------YVWPRMNM----------AIREF--

-----IRT-CDF-CQKDKP------NF-H-PNRAPV-LSF---------------RTPNG

PYEIYGFDL-----I-TL----PPTDF------GNK----YVMVMID----FFS------

--KFAY-CE-------PLK----SR-N-SS------YLL-SK--F-RNVIFKNP------

-------------FFPKMVVLDN-ARE--HS---------ELAKFMTN------------

-----------NNI--EP---HFTPPRHPSSNGQV--ENFNRTLKSRLRAKC--------

---KY----ENWDLVLQEILHDINASE---------------HSV----TKKSPFFI---

---------------QSG------------------------------------------

---------------AECPHNIFDNNYRNYNLDR--------------------------

----------------------------KTKFE-DIQKLIE---EEKNKRMA-----KFS

N--PKF-------NAY---N-----LGDLV--------LIKN-FESK-------------

KPAFLGPFKITSKSTAGTWYTVESD-------------

>Tor2

---------------------------------------------HYCKSAHDL---KGH

L-GV--------DRTAQ--FLSW------------CWWPHKMD----------DIRTY--

-----VAS-CAN-CLKQKG------YD-M-QPSRPD-RKHL--------------YRATR

PHEIIYCDF-----I-TL----PTSSRS-----GKR----HALTVMC----GFS------

--RWLQ-VY-------AVH----RC-R-SI------DAA-RG--L-MNYFLQFD------

--------------FPRTLSSDR-GRHFENE---------LLADLCKL------------

-----------LQI--RQ---NLHCAYRPESSGVI--ERCHKTLKSSLWAMVRD------

--NPR----LDWELALPSVVSAMNRST---------------NAA----TKVSPYKC---

---------------IFG-----RDPSFNGIAID--------------------------

------------NVAAANPASYA-------------------------------------

-------------------------QNTAELLD-RAHKFVKLSQEAADRDALERGKSKIQ

P--EEI-------------T-----AGHSI--------MLKR---------EVSAEAKEG

RNKWVGPYKVLA--SDGLILQIDVD-------------

>Tor1

---------------------------------------------------HLP----GH

A-RT--------TNMLGSMRDTG------------VIFPKMAK----------KIKQK--

-----IMT-CPT-CLATGS------TE-R-PRFTPI-TP----------------PKESH

PYMTVTVDL-----LGPL----PSTPT------RKK----YILAAID----NLT------

--RWIE-LR-------CIP----DK-T-AA------HVA-KA--L-MDIFFLRG------

--------------PPRAVSCDN-GREFSNT---------LLRELLAS------------

-----------FGT--CI---NYGTPYRPQGQGLI--ERANREIVKHLKGLN--------

--IEE----HKWDTFIPSIQLSMNLTY---------------HSA----LGSSPFQA---

---------------MHG---------------------------------WTL------

----------------AEPLFSKKVDGSEIQS-------------DVRLWVQ--------

-----ESGAR---------------MCAALALL-TAKQAVP---LTPIPV-------ESD

D--NAL-------------E-----PGTHV--------LLKT--------MTPPGVSAKL

YSPWKGGYVVRK-RCDHFTYLITP--------------

>Cer1

---------------------------------------------------HEGML-AGH

F-GI--------KKMWR-MVHRK------------FYWPQMRV----------CVENC--

-----VRT-CAK-CL-CAN------DH-S-KLTSSL-TPYR----------------MTF

PLEIVACDL-----M-DV----GLSVQ------GNR----YILTIID----LFT------

--KYGT-AV-------PIP----DK-K-AE------TVL-KA--FVERWAIGEG------

-------------RIPLKLLTDQ-GKEFVNG---------LFAQFTHM------------

-----------LKI--EH---ITTKGYNSRANGAV--ERFNKTIMHIMKKKT--------

--AVP----MEWDDQVVYAVYAYNNCV---------------HEN----TGETPMFL---

---------------MHG-----RDVMG--------------------------------

------------------PLEMSGEDAVGINY---------ADMDEYKHLLT--------

-----QELLK-------------VQKIAKEHAM-REQESYK---SLFDQKY------ASK

K--HRFP------------Q-----PGSRV--------LLEI-PSEKL-----GAQCPKL

VNKWSGPYRVIS--CSENSAEITPVLG-----------

>Cer2

---------------------------------------------------HSSLQ-GGH

H-NW--------KKTFR-KASVR------------YFWPDMKS----------DILRW--

-----CME-CIP-CQQRSK------PH---PSTREP-QQI---------------VVTSK

LFEKVGVDL-----CGPL----RSTAG------GHK----YYMNLIC----WFS------

--KFVV-SV-------PLT----DA-S-TD------TVV-RA--ILTEVVLKFG------

--------------TPSELVSDR-ASTFTSE---------AFRQFCKL------------

-----------VSI--QQ---HLAIPYHSKGNGAT--ERTFRTFHNMTSKYV--------

--NAA---HSDWDILLPYLTFSYNTVV---------------HST----TGETPFFL---

---------------VFG-----RDPVFAVDRIL--------------------------

--------------DPSPPKEAGKSDVKIWKE-------------HLVEILR--------

-----EAW-----------------KNTAEIAL-KAQLAYQ---KQANQ--------GAK

G--SEI-------------R-----PGDRV--------MFKN-FKSKIN------LSRKL

VKPWIGDYRVLE-VNHPKALILDLD-------------

>Cer3

---------------------------------------------------HESLG-GGH

F-GY--------RKTLH-KIKRN------------YYWPNMRS----------DVLKW--

-----TLQ-CKI-CQQKRN------PH---PSTREL-QKI---------------VITTK

VFEKVGVDL-----TGPL----RMTAS------GNK----Y---MVC----WFT------

--KFVI-SV-------PLP----NA-S-TE------TVA-DA--MMKELLLKFG------

--------------TPSQLVSDR-ASTFTSE---------AFRAFCRK------------

-----------LEI--QQ---HLAIPYHSKGNGAT--ERTFRTFHNMVSKHV--------

--NKT---HTDWDQILPYMTFVYNTTV---------------HDT----TGETPFFL---

---------------IFG-----RDPVFAIDKIM--------------------------

--------------HPSPPKEGEEVDIPAWKE-------------HLITTLR--------

----------------------LARKEAAERSL-KEQEARQ---KVANV--------GAK

G--SKI-------------V-----VGDRV--------FFQN-HKSKAN------LSRKM

VLPWIGEFEVIS-IDHPKAVIKDLE-------------

>Cigr1

---------------------------------------------------HNN---LGH

P-GF-S-RLYHFIKTRN--------------------LPFSSE----------EAKII--

-----CRQ-CKT-CAEVKP------RF-YKPASRNL-IKAM------------------R

PWERLSMDF-----KGPV--------------KGRNS---YLLIIVD----EYS------

--RFPF-VF-------PCK----RM-T-SA------VVI-QC--L-TNLFCLFG------

--------------FPAYVHSDR-GLSFISH---------EVKVFLTE------------

-----------RGI--AT---SKSTPYHPQGNSQC--ERVNQTIWRTVKLLL------HG

KLMAE----EQWEEVLPQALHAIRSLV---------------CLA----TNETPHER---

---------------FFNF---SRRSMT--------------------------------

--------------GTTLPTWLLTKGPV--------------------------------

----------------------LLRRFV-----------------------------RTK

G--DPLC--------------------DQV-------ELLDA------------------

---------------NPTYANIRHPDG-----------

>CsRN1

-------------------------------------------------HSHGFS----H

P-GI--------RATRK-LISAR------------FVWPFMNK----------DLTSW--

-----AKQ-CIA-CQRSKV-------T-R-HTNSPI-GSF---------------AVPDA

RFTHVHLDI-----VGPL----SPS-N------GFT----HILTMID----RFT------

--RWPV-PV-------PIS----DT-S-AE------TVA-FS--FLHHWVSNFG------

--------------IPSTVTTDR-GSQFQCN---------IFREFSTL------------

-----------FGF--HH---ISTAAYHPCSNGLV--ERFHRYLKAALTAHM--------

---NP----SSWSFSLPLILLAIRSTI---------------KED----LHCSPAEL---

---------------AYG-----TTLRL--------------------------------

------------------PGELVSTSGAQPES----------PV-TFVTRLK--------

-----QHM-----------------SELRATPT-RRS---------TRK--------EHI

S--TDL-------------S-----STPFV--------FVRH-DATR----------KPL

QPCYDGPFKVIE---RHSKYFVLERSG-----------

>Kabuki

---------------------------------------------------HNLS----H

P-GI--------RTTRK-MITNT------------FFWPNMNK----------DIGMW--

-----AKP-CIR-CQESKI-------Y-R-HTISDL-GNF----------------VDAG

RFEHIHVDI-----VGPL----PTSPQ------GFR----YCITIID----RCT------

--RWPE-AF-------PVH----DI-T-AD------KVA-KT--IYDGWITRFG------

--------------CPVRITSDQ-GRQFESN---------LFLKLTKL------------

-----------LGI--NK---ICTTPYHPQSNGAV--ERWHRSLKVALMTRL--------

---ND----TSWVDELSTAMFGLRASV---------------RTD----SGVSAAQL---

---------------TYG-----KNIRL--------------------------------

------------------PGDFYDVSKEENLCE---------PL-SLVNKIQ--------

-----KII-----------------KSFRPVRE-NQT---------SRS--------IFV

H--PDL-------------H-----KCSFV--------FVRD-DAVH----------KSL

KPPYDGPFRVIE---RGPKVFLVQLFD-----------

>Boudicca

-------------------------------------------------NVHNFS----H

P-GV--------RASIK-LIGER------------LCWPGMNK----------DVKEW--

-----ARS-CVS-CQKSKV-------I-R-HNKCPL-GSF---------------KTPDA

RFDHVHLDL-----VGPL----PDS-N------GYS----YLLTCVD----SFT------

--RWPE-AV-------PIK----DI-T-AE------TVA-RA--FVERWVANFG------

--------------CPSTITTDR-GRQFESG---------LFRCLTSL------------

-----------LGI--TR---FRTTAYHPQANGLV--ERFHRQLNASLSA-A--------

---NV----SQWTDALTLVLLGIRNAV---------------KAD----IG-TASQL---

---------------VYG-----TTLRL--------------------------------

------------------PGEFVDPSASSLNM----------DLNSYTSRLT--------

-----NAM-----------------RSVKPAHT-RSQ---------STD--------VFV

Q--PEL-------------R-----HSTHV--------FVRR-DSHR-----------PL

ESAYEGPFKVLQ---REPKYYVVDKNG-----------

>Osvaldo

---------------------------------------------------HDAVT-AGH

L-GS--------RKTIA-RVAAR------------YYWPGMYR----------NVRNY--

-----VQR-CEV-CQRYKP------SQ-L-QAAGQM-LTQ---------------VP-EE

PWATVCADF-----VGPL----PRSKH------GNT----MLLVFID----RFS------

--KWTE-MV-------PLR----SA-N-TA------ALQ-KA--FRERILARFGA-----

---------------PKVLITDN-GTQFTSR---------AFKNFLDE------------

-----------LGV--RH---QLTAPYTPQEN-PT--ERANRTVKTMIAQFAGS------

--DQR-----CW-DEAPELTLAVNSSV---------------SAS----TGYTAAFI---

---------------TQG-----REPRL--------------------------------

------------------PKTMFDAQTLGTGQ-------EAQSPIERAAKMR--------

-----EVL-----------------EIVRRNLE-----RAA---QDQARITIC--GGGSG

S--RLLGTKCGRRNATCPMP-----RTD-----------LQRSWHRDT----EGH--IRW

SSLYR---------------------------------

>Woot

---------------------------------------------------HQLL---GH

F-GA--------TKVYN-SMKRE------------YYWPNMYR----------TIKKR--

-----LRS-CDL-CQKTKS------SN-R-PHQGPL-TPI---------------LY-DH

IGDLVCVDF-----YGPL----PTGRL------GAS----YVFVVID----VFS------

--KFLK-LY-------PLR----KA-T-AK------IAA-K------RLIEDFSGY----

-------------IKPKCVLSDH-GTQFISN---------TWQNSLRA------------

-----------ADI--QP---TLSSIRHPESN-PS--ERVMRELGRIFRAYCRE------

--NHA-----SWVNHLSNIEDCLNYVP---------------HIS----TGFSPYEI---

---------------LYG-----RTPPN--------------------------------

------------------P---LDAVTSGL------------LPVRPPLTRE--------

-----EIH-----------------EKARENLKHHANLRQK---NQKGEVTV--------

------------------LE-----IEDWV--------LLKNKVTSDT----RTHQFAKF

MPLYSGPYKIIA-KPHPNTYQIADP-------------

>Ulysses

-----------------------------------------------------------H

--GG--------RKTLH-ALARQ------------YYWPNMAI----------QVRDY--

-----VRK-CDT-CKETKA------QN-Y-RMQVGI-GEE---------------VRTDR

PFQKLYIDF-----LGKY----PRSKR------GHA----WIFVVVD----HFS------

--KFTF-LK-------AMR----EA-T-AA------DVV-NF--LVHEVFFKFG------

--------------VPEVIHSDN-GRQFVSK---------SFDAMVQA------------

-----------FGI--TH---LRTPVYSPQSNA-A--ERVNRTVLSAIRTYL-G------

--QDH----REWDAYLPEVEVAIRNAV---------------HSAA---TGVTPFFA---

---------------VFG-----QQMYLNGSSYKLA------------------------

---------------------------------------------RKLSLAD--------

-----HSISD---------------LDAKDRLA-VIRSQVK---DHLHTAY------ERS

R--Q--------------------------------------------------------

--------------------------------------

>Circe

---------------------------------------------------HDQPT-SAH

C-GM--------AKCLE-RIRRR------------FYWPNMVI----------NVRDY--

-----IRN-CET-CQTTKY------LN-R-SKKPPM-AAQ---------------VQSDT

IFQRLYLVF-----FGPF----PRSKS------GNI----GILIILD----NFS------

--KFTF-LK-------FNT----KV------------II-SI--LRDEIFCAFG------

--------------VPETVVSDN-GTQFKSR---------DFSDFLSK------------

-----------YGV--LH---IFTGAYAPQSNG-A--ERVNRSINAALRAYI-R------

--SDH----RELDVFLSSINCSLRNSI---------------HQ-S---IGISPYQV---

---------------VFG-----KHMISHGNDYKLL------------------------

---------------------------------------------RKLNLLT--------

-----EGDVK---------------LSRTDEFQ-RIRSNIA---RHLNKAY------ETN

Q--K--------------------------------------------------------

--------------------------------------

>Gmr1

---------------------------------------------------RDALL-GAH

L-GM--------DKTRE-RVVAR------------FYWPGVRR----------DVARY--

-----CQE-CPD-CQRVRP---------RAVERSPL-IPM---------------PIIET

PFERIALDI-----VGPL----PRTSR------GHR----YLLVILD----YAT------

--RYPE-AL-------PLR----AA-T-SK------AYA-RI----SATLSRVG------

--------------LPKEILTDQ-GSCFMSR---------VVKELLKL------------

-----------LQV--SQ---LRTSVYHPQTDGLV--ERFNQTIKRMLKKSI-E------

--ADG----KNWDQLLPHVLFAIREVP---------------QAS----TGFSPFEL---

---------------LYG-----RRPRGILDLAK---------------EAW--------

------------ESHPS-PHRTTIEHVELVR-----------------DRMA--------

-----KVW-----------------PIVRDHLT-RAQQAQA---RVYNR--------GAR

V--RNF-------------R-----PGDRV--------LVLV-PTSEC----------KF

LAKWQGPYEVVE-AVGPVNYKVRQP-------------

>rGmr1

---------------------------------------------------HTHPM-AGH

L-GA--------ANTIQ-RIRDR------------FHWPGLNG----------EVKRY--

-----CQA-CPT-CQKTAP------QR---PPPSPL-IPL---------------PIIEV

PFDRIGLDL-----IGPL----PKSAR------GHE----HILVILD----YAT------

--RYPE-AI-------PLR----KA-T-SN------VIA-KE--L-FLLCSRVG------

--------------IPSEILTDQ-GTPFMSR---------LMADLCHL------------

-----------LKV--KQ---LRTSVYHPQTDGLV--ERFNQTLKRMLRRVV-A------

--EDG----RDWDLMIPYVLFGIREVP---------------QAS----TGFTPFEL---

---------------LFG-----RQPRGLLDVAR---------------QAW--------

------------EQEPA-PQRSLIEHVQDMR-----------------QRIE--------

-----RVM-----------------PLVRQHLT-EAQHAQR---RLYDR--------PAQ

A--REF-------------Q-----PGDQV--------LVLV-PTATS----------KF

LASWKGPYVVVE-KVGPANYRVRQP-------------

>RetroSor1

-----------------------------------------------------------H

S-GMCGSHIGPRALSAK-ALRQG------------FYWPTHIR----------DAEEI--

-----VKT-CKA-CQTFSP-------I-Q-SGPSAL-TQL---------------IPASW

PLQRWGMDL-----VGPM----PTAQG------GNK----FAVVAIE----YFT------

--RWIE-AK-------PLT----TI-T-SE------TIR-KF--FWQNIVCRFG------

--------------VPRLLTVDN-GKQFDSD---------NFKEFCHL------------

-----------IGT--KI---AFASVYHPESNGAV--ERANRTIFSAISKTL-L----NL

RK-------GKWVEELPRVVWSHNTTV---------------SRA----TGFTPFKL---

---------------LYG-----EEAML--------------------------------

------------------PEEIKHQSLRSMKQQLAEDE-EYC-----KETLE--------

-----SIR-----------------LEAVENIT-RYQQETK---NWRDR--------KVV

R--KDI-------------Q-----NGDLV--------LRKK-GDHP--------NAGKL

QPKWEGPYTAIQ-AGRSGSFYLKDL-------------

>Cinful1

-----------------------------------------------------------H

A-GLCGSHIGSRPLLGK-IFRQG------------FYWPKAAS----------DAAEL--

-----VQK-CEG-CQKCAR-------D-Q-KQPSSL-TQL---------------IQPIW

PLQRWGLDL-----LGPL----PPAQG------NLR----YVVVAVE----YFS------

--KWIE-AK-------PLA----TI-T-SA------TVQ-KF--FWQNIVCRFG------

--------------VPKAITVDN-GTQFDSE---------AFRDFCDQ------------

-----------IGT--KI---HFASVRHPESNGLV--ERANGIIMTGIMKSI-F----NQ

PR-------GKWPDQLTKVVWSHNTTT---------------SRS----TGFTPFKL---

---------------LFG-----DEAIT--------------------------------

------------------PEEAKTGSIRVVASAASGSEADYS---VEKDALE--------

-----GIR-----------------LQAVENIN-KYQAETI---KWRDR--------KVR

L--KNI-------------E-----PDTWC--------FRRV-ANPE--------TVGKL

QLKWDGPFLVAS-SSRPGSYRLKDM-------------

>B1147A04.5

-----------------------------------------------------------H

E-GECGSHSASRTLVGK-AFRQG------------FYWPTALN----------DAVDL--

-----VRR-CRA-CQFHAK-------Q-I-HQPAQA-LQI---------------IPLSW

PFAVWGLDI-----LGPF----RRAPG------GFE----YLYVAID----KFT------

--KWPE-AY-------PVV----KI-D-KH------SAL-KF--I-KGITARFG------

--------------VP-RIITDN-GTQFTSE---------LFGDYCED------------

-----------MGI--KL---CFASPAHPRSNGQV--ERANAEILRGLKTKT-F----DI

LKKHG----DSWIEELPAVLWANRTTP---------------SRA----TGETPFFL---

---------------VYG-----AEAVL--------------------------------

------------------PSELTLRSPRATMYCEADQDQFRR---DDLDYLE--------

-----EQR-----------------RRAALRAA-RYQQSLR---RYHQR--------HVR

A--RSL-------------C-----VDDLV--------LRRV-QTRA--------RLSKL

SPMWEGPYRVIG-VPRPGSVRLATG-------------

>Ogre

-----------------------------------------------------------H

E-GSFGTHAGGHAMAKK-LLRAG------------YYWMTMES----------DCFKY--

-----ARK-CHK-CQIYAD-------R-V-HVPPSP-LNV---------------MNSPW

PFAMWGIDM-----IGKIE---PTASN------GHR----FILVAID----YFT------

--KWVE-AA-------SYA----NI-T-KQ------VVT-RF--IKKEIICRYG------

--------------VPERIITDN-GSNLNNK---------MMKELCKD------------

-----------FKI--EH---HNSSPYRPKMNGAV--EAANKNIKKIVRKMV-V----TY

K--------D-WHEMLPFALHGYRTSV---------------RTS----TGATPYSL---

---------------VYG-----MEAVL--------------------------------

------------------PVEVEIPSLRVLLDVKLDEAEWIRTRFNELSLIE--------

-----ERR-----------------LAVVCHGQ-LYQRRMK---RAFDQ--------KVR

P--RSY-------------Q-----IGDLV--------LKRI-LPPGT-----D-NRGKW

TPNYEGPYVVKK-VFSGGALMLTTM-------------

>Grande14

-----------------------------------------------------------H

A-GQCGIHAASRTRGRK-VFRSG------------FYWPTAKN----------DAAEL--

-----VQR-CEA--QYLSK-------Q-Q-HMPAQQ-LQT---------------IPVTW

PFACWGLDM-----IGPF----KKAQG------GYT----HVLVAID----KFT------

--KWIE-FK-------PIA----SL-T-SA------KVV-EF--D-KNEDIRRE------

--------------V--------------------------------D------------

-----------KGI--TC---CL----EPMNT----------------------------

---------------YPSFAWRHS-------------------------------FLHG-

-------------LWVGG-----SGTM---------------------------------

-------------------ADLKLGAPRLIF-----------------------------

---------------------------ESIAIS-GHQAG--------HR--------CTR

T--HLF-------------S-----VGLVL--------LRRI-LTGE--------GRHKL

SPLWEGPFMVAE-VTRPGSYRLTQM-------------

>Tat41

-----------------------------------------------------------H

E-GAGGNHSGGRSLAAK-IKKHD------------QYWPTMIT----------DCIRF--

-----VAK-CEP-CQRHAS-------I-I-HSPTEA-LTT---------------STAPY

PFMRWAMDI-----VGPL-----PSSN------GKR----FMLIMTD----YFT------

--KWVE-AE-------AYN----KI-Q-AG------EVQ-RF--VWKNIICRHG------

--------------LPYEIITDN-GSQFISR---------QFEDFCAK------------

-----------WRI--RL---NKSTPRYPQGNGQA--EATNKIILDGLKKRL-E----AK

K--------GAWADELDGVL-SHRTTP---------------RRS----TGATPFSL---

---------------TYG-----IEAMA--------------------------------

------------------PAEVSVGSLR--RTMLVNNPLNNRMLLSNIDDAE--------

-----EFR-----------------DSALLRIQ-NYQQAAA---RHYNT--------KVK

P--RSF-------------T-----VGDLV--------LRKV-YENTA-----ELNAGKL

GAKWEGPYLVSK-VVRSGVYELLTM-------------

>Tft2

-----------------------------------------------------------H

L-GSRSNRTVGRHTSAK-ASR------------------TRSS----------D------

-----ISR-NRPNSDRLGA-------D-W-REPIKH-YIV----------------TGEL

PKNRWQAR----------------KNE------NRR----KILSIEE----FFS------

--KWFE-VE-------AYV----NI-K-DS------AVK-TF--IWQNIICRFG------

--------------VPYEIVTDN-GPQFISH---------EFEAFCSD------------

-----------LGI--KV---SYSTPRYPQGNGQA--EAANKTILSNLKKRL-S----HL

K--------GGWYDELQPFLWAYRTTP---------------RRS----TGETPFSL---

---------------VYR-----MEAVV--------------------------------

------------------PAELNVPGLR--RTAPLNEEENSAMLHDSLDTIN--------

-----ERR-----------------DQALIRIQ-NYQHAEA---RYYNS--------KVK

S--RPF-------------F-----VGDYV--------LKRV-FDNKK-----EEGAGKL

GINWEGPYIVIE-VVWNGVYKLKDL-------------

>RIRE2

-----------------------------------------------------------H

S-GICGNHAAARTIVGK-AYRQG------------FFWPTAVS----------DADKI--

-----VRT-CEG-CQFFAR-------Q-I-HLPAQE-LQT---------------IPLSW

PFAVWGLDM-----VGPF----KKAVG------GYT----HLFVAID----KFS------

--KWIE-AK-------PVV----TI-T-AD------NAR-DF--F-INIVHRFG------

--------------VPNRIITDN-GTQFTGG---------VFKDFCED------------

-----------FGI--KI---CYASVAHPMSNGQV--ERANGMILQGIKARV-F----DR

LKPYA----GKWVQQLPSVLWSLRTTP---------------SRA----TGQSPFFL---

---------------VYG-----AEAML--------------------------------

------------------PSEVEFESLRFRNFREERYEEDRV---DDLHRLE--------

-----EVR-----------------EAALIRSA-RYLQGLR---RYHNR--------NVR

S--RAF-------------L-----VGDLV--------LRKI-QTTR--------DRHKL

SPLWEGPFIISE-VTRPGSYRLKRE-------------

>412

---------------------------------------------------HDDPIQGGH

T-GI--------TKTLA-KVKRH------------YYWKNMSK----------YIKEY--

-----VRK-CQK-CQKAKT-------T-K-HTKTPM-TIT---------------ETPEH

AFDRVVVDT-----IGPL----PKSEN------GNEY---AVTLICD-----LT------

--KYLV-AI-------PIA----NK-S-AK------TVA-KA--IFESFILKYG------

--------------PMKTFITDM-GTEYKNS---------IITDLCKY------------

-----------LKI--KN---ITSTAHHHQTVGVV--ERSHRTLNEYIRSYI-S------

--TDK----TDWDVWLQYFVYCFNTTQ---------------SMV----HNYCPYEL---

---------------VFG-----RTSNL--------------------------------

------------------PKH-----------------------FNKLHSIE--------

-----PIYNIDDYAKESKYRLEVAYARARKLLE-AHKEKNK---ENYDL--------KVK

D--IEL-------------E-----VGDKV--------LLRN------------EVGHKL

DFKYTGPYKIES-IGDNNNITLLTN-------------

>Mdg1

---------------------------------------------------HDDPSEGGH

S-GI--------SRTLR-KMKNC------------CCWPRMTK----------AISEY--

-----VET-CLK-CQQAKT-------T-K-HTKTPL-TIT---------------ETPAT

AFDKVLIDT-----IGPL----PRSEN------GNEY---AVTIICD-----LT------

--KYLV-TV-------PIP----NK-S-AK------SVA-KA--IFENFILKYG------

--------------PMKTITTDM-GTEYKNQ---------IIDDLCKY------------

-----------MKI--KN---ITSTAHHHQTLGTV--ERSHRTFNEYVRSYI-S------

--VDK----TDWDIWIQYFTYCFNTTP---------------SVV----HEYCPYEL---

---------------VFG-----R---L--------------------------------

------------------PRQFI--------------------DFNRIDRID--------

-----PIYNMDDYSKEVKLRLEIAYRRAKNMLD-KAKADRK---IKYDR--------NIS

N--FEL-------------K-----IGDKI--------LLKN------------ETGHKL

DNNYLGPYLVSE-IGDNDNITIIGN-------------

>Blastopia

---------------------------------------------------HEV----GH

L-SL--------QKTMH-SIQQQ------------FFYFLIWEY---------KVKKL--

-----ISN-CIK-CIIHSK------KL-G-KQEGYL-NCI---------------DKGDA

PLHTLHIDH-----LGPM----DSSAK------QYK----YILATVD----AFS------

--KFVW-LF-------PTK----ST-G-QE------EVV-KR--L-TDWSNIFG------

--------------FPKRIVSDK-GTAFTSG---------AFEQFMSS------------

-----------HNV--EH---VCTTTGVARGNGQI--ERVNRLILAIISKLS-S------

--DEP----SKWYKYVPEVQKAINCHV---------------HSS----LKLSPFEV---

---------------MFG-----TKMYT--------------------------------

-----------------------------------------RVEDRLLELLQ--------

-----EEVVCQ-F-NEDRYE---MRQLVKRNIE-QAQKDYK---RNYDKK-------RRA

E--YKY-------------K-----AGDLV-------AIKRT----------QFVAGRKM

ASGYLGPYEVTG-VKDNGRYDVKKA-------------

>Micropia

---------------------------------------------------HESIM---H

L-GW--------QKTLD-KVYQY------------YWFAKMNK----------YVRKF--

-----VSN-CIT-CRSVKS------SS-G-KVQAEL-HSI---------------PKTSI

PWHTIHIDI-----TGKL----SGKSD------LKE----YVIVQID----AYT------

--KFVY-LL-------XTL----KI-D-AE------SCV-NA--M-KSSISLFG------

--------------VPDRIIADQ-GRCFTSS---------KFSEFCVS------------

-----------QKV--EL---HLIATGMSRANGQV--ERVMETLKNLLSVV---------

E-SSQ----RSWQDALGEVQLALNCTI---------------SRA----TDASPLEM---

---------------LIG-----KQARPLGLVP---------------------------

------------------PCETE----------------------CEIDL----------

--------AT-------------VRAHATENMNSLASYDKS----RFDSS-------RAA

V--DKH-------------H-----VGDYV--------LLRN----------EERHQTKL

DPKFRGPFLVTEVLEGDRYTLKSL--------------

>Mdg3

---------------------------------------------------HNE---LGH

V-GR--------DKMIE-AIMKN------------YWFPNLKQ----------KCSTH--

-----ISN-CLK-CISFSP--K------TGKTEGFL-HNI---------------PKGNK

PFEIIHIDH-----YGPVD--LARPKK-------------HILVIVD----AFT------

--KFVR-LY-------ATK----TT-N-TK------EVI-QS--L-NDYFRAYS------

--------------RPKCIISDR-GACFTSG---------DFDSFLKE------------

-----------CNV--KH---IKIATGSPQANGQV--ERINRSLGPMISKLI-E------

--PDQ---GLHWDLVLEKVEYTLNNTL---------------HRS----IKQYPSIM---

---------------LFGL-----------------------------------------

-----------------------------------------QQKGQIMDELK--------

-----EKIEEIG---------ETIEERDLESIRNKGEASQKIA-QAYNKEYVD--KKRKR

S--GVF-------------T-----KGTTS--------WLKI--------LTQQQAAKKL

IPKHKGPYVISK-VLKNDRFLLED--------------

>CoDi4.4

--------------------------------------------------PLTPWQ----

--NRA-------EAEIR-ELKKQVLWI--------MSHEGIPR----------RFWDY--

-------------VAEYVSEIR---SR-TAHPLYDL-------KG----------RTPIE

HVTGETPDI---TEWLEYRMY-------------------QPVWYLD-P-GDFPE-EKKL

LGRWLG---------------------PAH------RVG-QA--LCCWIVPQSG-RVI--

-----------ARSTVQPVSEEERGLDSFKTR------LKDFDKSVQD-FLNRGDTHVP-

----D----GGFG-----------WKANRVYAAI---NEDIDPVEKEA------------

------------------------SMP-------------------------EADEYTE-

------------------------------------------------------------

------------------------------------------------------------

-------------------------ESFDKYLS---------------------------

---AEVV-----------------------------------------------------

--------------------------------------

>CoDi3.1

----------------------------------------------TVTEPYSPWQ----

--NRC-------EHEFG-AARIHTRLV--------LETTKCPE----------QLWDY--

-------------ALAYVIFVR---NH-TARK--AL-------AW----------ITPIT

AMTGDTHDI---SEILVFEFF-------------------EPVQYFDNPDVKFPQ-NKAK

VGRWLG---------------------IAT------NVG-QA--LCYHILTDKG-TVI--

-----------TRSTVTPL--QNLDSSALQTA------LATFDATIRE-----------I

YQPSD----FALGN-------KIKAPAFRRDEAMKVARRSDDPGDGNTR-----------

------------------NRHVLYDLN-------------------------EGDDHIQL

------------------------------------------------------------

------------------------------------------------------------

----------------------DPGLTVDDFFE---------------------------

---NDSP-----------------------------------------------------

--------------------------------------

>CoDi2.4

----------------------------------------------WQSEPNHQHQ----

--NFA-------ERRIA-TIEANTNNI--------LNLSGAPD----------SAWLL--

-------------CVTYVCYVF---NH-LAHE--SL-------DN----------RTPLE

VLTGSTPDI---SVLLQFHFW-------------------EPVYYKL-ENATFPSGGTEQ

QGRFVG---------------------IAD------SVG-DA--LTYKILTHTTNRIL--

-----------HRSSVRSATI-------------------------------PGQTNLRL

T-PQD----GESG------------------------PKPINFIKSRRTE----------

----------------NKNSYAIKELP-----------------------GFTPDDLIGR

----------TF---LTD------------------------------------------

-----------T------------------------------------------------

---------------------RDDGERLKARIT---------------------------

---RKILD---------PDK-----PSDVK--------FLVE-IN---------------

--------------------------------------

>CoDi4.1

----------------------------------------------HVTEPHSQWQ----

--NRA-------EGEIR-EIKKSVRHR--------LQASRAPK----------RLWCF--

-------------CTEWVSAVR---RL-TALSLPAL-------NG----------RVATE

LLEGETPDI---SEYAQFDWY-------------------EPVWFID-PTSSFPE-PKRK

LGRWIG---------------------VAS------DVG-QA--MTFWILPKSC-SPI--

-----------ARSLVARVDPDVSCTDEFKAD------LAMLDLSIDN-KIGNNKTAEQN

K-EID----SSLGN-------LVSGPADDLFEKVA--NKEFYPLEEAA------------

------------------------EKA-------------------------EADDFTP-

------------------------------------------------------------

------------------------------------------------------------

-------------------------ESMDEYLT---------------------------

---AEVL-----------------------------------------------------

--------------------------------------

>CoDi4.5

----------------------------------------------RQTEPYSPWS----

--NSA-------ELEIR-ELKKATGRR--------LLKSGAPK----------RLWDY--

-------------CLELESLIR---SH-TAHDIFKL-------NG----------RTPEA

MMMGETPDI---SYICEFGWY-------------------DWVMFRD-EVAPYPE-PKLV

LGRYLG---------------------PSV------DVG-PA--MSARIIKANG-QVV--

-----------DRSTFRHLTADELQDEVHKAT------RDDFTKLLHE-KLGSAATFADL

TLGGD----GGRSF-------LLPTPEHIPYDDV-----PFDILEEEL------------

-------------------------MP-------------------------TP------

------------------------------------------------------------

------------------------------------------------------------

-------------------------ESGDEYVN---------------------------

---AEVM-----------------------------------------------------

--------------------------------------

>CoDi4.3

----------------------------------------------KEIEPYSPWL----

--NRA-------ETAIK-ALKRMTNTA--------MSKSQASV----------RLWDI--

-------------CLELQCLIR---SS-IAHNIYAL-------ND----------DVPNT

AVSGDTTDI---SHLCEFAWY-------------------DWVWYLD-PV-DFPE-DKRK

LGRWLG---------------------PAH------DIG-DA--MCARILARNS-QII--

-----------SRTTYSPLSTSDLNSQQ--------------VKLLQE----SFETNVRL

VMNEDDRIKNTYVF-------EVEPSEFETYSD-------HYTGDQEM------------

-------------------------MP-------------------------QADEYDH-

------------------------------------------------------------

------------------------------------------------------------

-------------------------EAFNEYIN---------------------------

---AEVM-----------------------------------------------------

--------------------------------------

>CoDi7.1

-----------------------------------------------KSVPNNQNQ----

--NQA-------ERKIQ-DVKKRTILT--------LRYGKTPLTFW--------------

---------CF--CQQFIV---DCLNH-SAHKDLNF-------------------RTPME

KMYGHTPDIS----MFRFRFW-------------------EPVWYYE-PTAKYPA-PNFL

PGRFVG-IAWDHGDAFTYKI--WTTPN-ND---WK-----QGRELVRNVVRSRH------

----------LEEKEPVVSYQDE-DLLFSKTQP-------SRTQRRRS------------

---------------------KKRNSRSDDSRGAK--ER---------------------

---------NLDGSELESLVRFDDTPPITSVDSEEQGGDKAESQS----DHCSPVEV---

------------------------------------------------------------

------------------------------------------------------------

------------------------------------------------------------

-------------------E----------------------------------------

--------------------------------------

>CoDi5.2

PMH------------------------------------------------------KGH

L-----------DQQRA-NIKST------------QLKPSALLA---------SAPHG--

-TEHDENPVPDN-------------------------PPALRSNFLY--------ADAYE

ATGKIFSDL-----TGRFV---TSSSS------GNA----YMLVVYD----YDS------

--NFIH-VE-------PMK----NR---TG-----PEIL-AAYRRAFDLFSSRG------

-------------LRPQLQRLDN-------------EASAALQQFMTD------------

-----------SKV--DF---QLVPPHLHRRNAA---ERAIRTFKNHFIAGLCS------

--TDKDFPLHLWDRLLPQAIMTLNLLR---------------GSRIN--PRLSAWAQ---

---------------VHG------------------------------------------

------------------------------------------------------------

------------------------------------------------------------

------------------------------------------------------------

--------------------------------------

>CoDi5.3

PMV------------------------------------------------------KGH

L-----------DQQRS-NLRST--------------KPKVTL----------SASVDPD

DINFDTNPVVQD-------------------------PPAARTQFLY--------ADFAE

VTGKIFTDP-----TGRFV---TTSSS------GNA----YMLVVYD----YDS------

--NFIH-VE-------AMK----NR---TG-----PEIL-SAYKRAHAMLSSKG------

-------------LRPQLQHLDN-------------EASTALQQFMSS------------

-----------VDI--DF---QLAPPHVHRRNAA---ERAIRTFKNHFIAGLCS------

--TDKNFPLHLWDHLLPQAIMTLNLLR---------------GSRIN--PNLSSWAQ---

---------------LHG------------------------------------------

------------------------------------------------------------

------------------------------------------------------------

------------------------------------------------------------

--------------------------------------

>CoDi5.1

AMV------------------------------------------------------KGH

L-----------DQQRA-NLRSTKLPP--------VGSPTTTAP---------PARSVP-

----DLDP-PNA-------------------------PPVARTHHVF--------AAHQR

VTGQIYTDQ-----PGRFL---TPSSA------GHN----DMLVLYD----YDS------

--NAIH-VE-------LMK----NK---SG-----PEIL-AAYKRAHALFTQRG------

-------------LRPQLQRLDN-------------EASAALQSFMTS------------

-----------EHV--DF---QLAPPHLHRRNAA---ERAIRTFKNHFIAGLCT------

--TNPDFPLHLWDRLLPQALITLNLLR---------------RSRIN--PKLSAHAQ---

---------------LHG------------------------------------------

------------------------------------------------------------

------------------------------------------------------------

------------------------------------------------------------

--------------------------------------

>CoDi5.4

QCE------------------------------------------------------QGH

M-----------DQRRT-GIRSTK------------------------------SSHA--

-----VPP-PDI-V-----------DTMEEP-TQA--PQNDKTN-------MVFMTIA-E

AEGQLFTDQ-----TGRFP---VTSNR------GNN----YIVLFYV----VDA------

--NFIK-SY-------PIK----SR---HR-----TELL-KAYDDVYKYLCIRG------

-------------YRPKLHRLDN-------------ETSKDVEDFIAE------------

-----------QNA--KH---QYTPPDIHRTNIA---ERMIRTWKNHMCAVRAG------

--TPKTYRLSNWCKDLEQVDMTLNMMR---------------PCTQN--PNLSAYEA---

---------------MEG------------------------------------------

------------------------------------------------------------

------------------------------------------------------------

------------------------------------------------------------

--------------------------------------

>CoDi5.5

ETQ------------------------------------------------------KGH

M-----------DRQRK-GVRSTKD------------KSTQ------------ETQLT--

--------------------------------------PMQREH-------DVFIKVY-T

QRDVIYTDQ-----TGRLP---AISSK------GNQ----YVMVLCE----IDG------

--NVIL-VE-------PMR----SK---AD-----GEMQ-KAYLRLLARLKAAK------

-------------IVPKKHVLDN-------------ECSEAMKALIKE------------

-----------T-C--QL---ELVPPGTHRRNIA---EKAIQTFKKHFIGILAG------

--TADDFPLHLWDRLLPQAEMTLNLLR---------------QANAR--PTVSAWAY---

---------------LFG------------------------------------------

------------------------------------------------------------

------------------------------------------------------------

------------------------------------------------------------

--------------------------------------

>CoDi5.6

ATD------------------------------------------------------KGH

M-----------KRLRQ-NIRST--------------RPKQ------------HSPST--

-----TAE-DNM-ANRLKQLISE--ELDANP-------PEEKMEEGNGTNVFCFAAIADK

IEGTVYVDN-----TGRFP---VRSLE------GHL----YLFVLYD----YGS------

--NAIL-VE-------ALK----TM---ES-----KEFI-AAFQKKISYLTQRG------

-------------FKPRFNVMDN-------------IVSKAVQSFLEE------------

-----------HQI--GI---QIVEPHNHRVNAA---ERAIQTFKDHFIAGPST------

--TDKDFPLQLWDQLLEQAQDSLNMLR---------------TSRVN--PRLSAYHV---

---------------LEG------------------------------------------

------------------------------------------------------------

------------------------------------------------------------

------------------------------------------------------------

--------------------------------------

>Retrofit

---------------------------------------------------HSR---LGH

P-SL--------PIVKQ-VISRN-------------NLPC-SVE---------SVNQS--

-------V-CNA-CQEAKS------HQ-LPYIRSTS-VS-------------------QF

PLELVFSDV-----WGPAP---ES-VG------RNK----YYVSFID----DFS------

--KFTW-IY-------LLK----YK---SE-------VF-E---KFKEFQALVERMFD--

-------------RKIIAMQTDWRGGRYQKLN--------SFF---AQ------------

-----------IGL--II---MCHVLTLIRQNGSA--ERKHRHIVEVGLSLL-----SYA

SMPLK-----FWDEAFVAATYLINRIP---------------SKTI---QNSTPLEK---

---------------LFN------------------------------------------

------------------------------------------------------------

------------------------------------------------------------

------------------------------------------------------------

--------------------------------------

>Koala

---------------------------------------------------HSR---LGH

P-AF--------PIVKR-IVQSH-------------KLSCLDVE---------SNNIS--

-------V-CDA-CQKAKS------HQ-LPFGLSTS-EV-------------------HS

PFELVYSDV-----WGPAP---TS-VG------GKK----YYVSFID----AYS------

--RFTW-IY-------LLK----FK---SE-------VF-E---KFHEFQNHVERFFD--

-------------RKIKTIQTDW-GGEYQKLN--------SFF---NK------------

-----------IGI--LH---HVSCPHTHQQNGSA--ERKHRHIVEVGLSLL-----AHA

FIPLK-----FWDEAFSSAVYLINRIP---------------TKVL---QYRSPLEQ---

---------------LYN------------------------------------------

------------------------------------------------------------

------------------------------------------------------------

------------------------------------------------------------

--------------------------------------

>Hopscotch

---------------------------------------------------HGR---LGH

A-SK--------PIVLR-VINQN-------------KLPC-SNE---------SPSES--

-------V-CDA-CQQGKS------HQ-LPFPKFFS-VS-------------------SN

PLELIHSDV-----WGPAS---DS-VG------AKR----YYVSFID----DYS------

--KFVW-IY-------FLK----FK---SE-------VF-E---KFKEFQSMVERQFN--

-------------RKILGMQTDW-GGEYQKLN--------SFF---KQ------------

-----------IGI--AH---QVSCPHTHQQNGAV--ERKHRHIVEIGLSLL-----AHA

SMPLK-----FWDEAFLAATYLINRLP---------------RKVI---DFDTPLAR---

---------------LFH------------------------------------------

------------------------------------------------------------

------------------------------------------------------------

------------------------------------------------------------

--------------------------------------

>Melmoth

---------------------------------------------------HRR---LGH

A-SL--------QRLDA-ISD------------------SLGTT---------RHKNKGS

------DF-CHV-CHLAKQ------RK-LSFPTSNK-VC-------------------KE

IFDLLHIDV-----WGPFS---VETVE------GYK----YFLTIVD----DHS------

--RATW-MY-------LLK----TK---SE-------VL-T---VFPAFIQQVENQYK--

-------------VKVKAVRSDN-APEL------------KFTSFYAE------------

-----------KGI--VS---FHSCPETPEQNSVV--ERKHQHILNVARALM-----FQS

QVPLS-----LWGDCVLTAVFLINRTP---------------SQLL---MNKTPYEI---

---------------LTG------------------------------------------

------------------------------------------------------------

------------------------------------------------------------

------------------------------------------------------------

--------------------------------------

>Vitico12

---------------------------------------------------HMR---LSH

V-SY--------SKLTV-MMKKS-------------MLKGLPQL----------------

---------------EGKA------HQ-LSYEESKW-KA-------------------KG

PLELIHSDV-----FGPVK---QAXLS------GMK----YMVTFID----DFS------

--RYVW-VY-------FMK----EK---SE-------TF-S---KFKEFKEMTEIEVD--

-------------KRIHCLRTDN-GXXYTSN---------EFFYFLRE------------

-----------CRV--RH---QFTCANTLQQNGVA--ERKNRHLAEICRSML-----HAK

NVPGR-----FWAEAMKTXAFVINRLP---------------QQRL---NFSSPFEK---

---------------LWN------------------------------------------

------------------------------------------------------------

------------------------------------------------------------

------------------------------------------------------------

--------------------------------------

>Oryco11

----------------------------------------------------------RE

K-RK--------RALKL-LRTKG-------------MVQGLPFI---------TLKSD--

-------P-CEG-CVFGKQ------IR-ASFPHSGA-WR----------------AS--A

PLELVHTDI-----VGKVP---TISEG------GNW----YFITFID----DYT------

--RMIW-VY-------FLK----EK---SA-------AL-E---IFKKFKAMVENQSN--

-------------RKIKVLRSDQ-GGEYISK---------EFEKYCEN------------

-----------AGI--RR---QLTAGYSTQQNGVA--ERKNRTINDMANSML-----QDK

GMPKS-----FWAEAVNTAIYILNRSP---------------TKAV---PNRTPFEA---

---------------WYG------------------------------------------

------------------------------------------------------------

------------------------------------------------------------

------------------------------------------------------------

--------------------------------------

>Vitico11

---------------------------------------------------HLR---YGH

L-NV--------KGLKL-LSKKE-------------MVFGLPKI---------DSVN---

-------V-CEG-CIYGKQ------SK-KPFPKGRS-RR----------------AS--S

CLEIIHADL-----CGPMQ---IASFG------GSR----YFLLFTD----DHS------

--RMSW-VY-------FLQ----S------------------------------------

----------------KVLRTDR-GGEFLSN---------DFKVFCEE------------

-----------EGL--HR---ELTTPYSPEQNGVV--ERKNRTVVEMARSMM-----KAK

NLSNH-----FWAEGVATAVYLLNISP---------------TKAV---LNRTPYEA---

---------------WYG------------------------------------------

------------------------------------------------------------

------------------------------------------------------------

------------------------------------------------------------

--------------------------------------

>Araco

---------------------------------------------------HLR---FGH

L-NF--------GGLEL-LSRKE-------------MVRGLPCI---------NHPNQ--

-------V-CEG-CLLGKQ------FK-MSFPKESS-SR----------------AQ--K

PLELIHTDV-----CGPIK---PKSLG------KSN----YFLLFID----DFS------

--RKTW-VY-------FLK----EK---SE-------VF-E---IFKKFKAHVEKESG--

-------------LVIKTMRSDR-GGEFTSK---------EFLKYCED------------

-----------NGI--RR---QLTVPRSPQQNGVA--ERKNRTILEMARSML-----KSK

RLPKE-----LWAEAVACAVYLLNRSP---------------TKSV---SGKTPQEA---

---------------WSG------------------------------------------

------------------------------------------------------------

------------------------------------------------------------

------------------------------------------------------------

--------------------------------------

>Poco

---------------------------------------------------HKR---LGH

F-NQ--------ETLMQ-MQKKK-------------MVQGMPKL---------EEEIT--

-------V-CSS-CQYGKQ------NR-LPFPQNKA-WR----------------AT--K

KLQLIHTDV-----AGPLK---TISLN------ESR----YYIAFID----DYT------

--RMCW-VY-------FLK----FK---TE-------VA-S---VFMRFKNWIENQSG--

-------------HRIQVVRSDN-GTEYTSN---------KFAKFCHD------------

-----------AGI--EH---QYTTPYTPQQNGVS--ERKNRTIMEMARCLL-----FEK

DLPKK-----FSCIGCQQEHY------------------------------KTRLHM---

---------------KLG------------------------------------------

------------------------------------------------------------

---------------------------------------------------------MVT

------------------------------------------------------------

--------------------------------------

>Oryco12

---------------------------------------------------HAR---YGH

L-NF--------PALRK-LVQQE-------------MVRGLPLL---------QQVTQ--

-------V-CDG-CLLGKQ------RR-AAFPAQSK-YC----------------TN--E

HLVLVHGDL-----CRPIE---PATPA------GNR----YFLLLVD----DMS------

--RYMW-LT-------LIR----SK---DE-------AA-N---AIKHFQARAVVETG--

-------------RKLHALRTDR-GGEFTSI---------EFGEYYAN------------

-----------LGV--GR---KLTVPYSPQQNGVV--ERRNQTIVATARSMM-----KAK

GVPGR-----FWGEAMSTTVFLLNRSP---------------KKSL---DNQTPYEA---

---------------WYG------------------------------------------

------------------------------------------------------------

------------------------------------------------------------

------------------------------------------------------------

--------------------------------------

>Endovir11

---------------------------------------------------HQR---LGH

M-NA--------RSMSK-LVNKE-------------MVRGVPEL---------KHIEK--

------IV-CGA-CNQGKQ------IR-VQHKRVEG-IQ----------------TT--Q

VLDLIHMDL-----MGPMQ---TESIA------GKR----YVFVLVD----DFS------

--RYAW-VR-------FIR----EK---SE-------TA-N---SFKILALQLKNEKK--

-------------MGIKQIRSDR-GGEFMNE---------AFNSFCES------------

-----------QGI--FH---QYSAPRTPQSNGVV--ERKNRTLQEMARAMI-----HGN

GVPEK-----FWAEAISTACYVINRVY---------------VRLG---SDKTPYEI---

---------------WKG------------------------------------------

------------------------------------------------------------

------------------------------------------------------------

------------------------------------------------------------

--------------------------------------

>SIRE14

---------------------------------------------------HQR---FGH

L-HL--------RGMKK-IIDKG-------------AVRGIPNL---------KIEEG--

------RI-CGE-CQIGKQ------VK-MSHQKLQH-QT----------------TS--R

VLELLHMDL-----MGPMQ---VESLG------GKR----YAYVVVD----DFS------

--RFTW-VK-------FIR----EK---SE-------TF-E---VFKELSLRLQREKD--

-------------CVIKRIRSDH-GREFENS---------RLTEFCTS------------

-----------EGI--TH---EFSAAITPQQNGIV--ERKNRTLQEAARVML-----HAK

ELPYN-----LWAEAMNTACYIHNRVT---------------LRRG---TPTTLYEI---

---------------WKG------------------------------------------

------------------------------------------------------------

------------------------------------------------------------

------------------------------------------------------------

--------------------------------------

>Opie2

---------------------------------------------------HRR---LAH

V-GM--------NQLSK-LSKRD-------------LVVGLKDV---------KFEKDKL

---------CSA-CQAGKQ------VA-CSHPTKSI-MS----------------TS--R

PLELLHMEF-----FGPTT---YKSIG------GNS----HCLVIVD----DYS------

--RYTW-MF-------FLH----DK---SI-------VA-E---LFKKFAKRGQNEFN--

-------------CTLVKIRSDN-GSKFKNT---------NIEDYCDD------------

-----------LGI--KH---ELSATYSPQQNGVV--EMKNRTLIEMARTML-----DEY

GVSDS-----FWAEAINTACHATNRLY---------------LHRL---LKKTSYEL---

---------------IVG------------------------------------------

------------------------------------------------------------

------------------------------------------------------------

------------------------------------------------------------

--------------------------------------

>TSI9

---------------------------------------------------HRR---LGH

L-SF--------DLLCR-LSSMD-------------LIDGLPKL---------KFEKDLI

---------CAP-CKHGKM------VA-ASHAPVTQ-----------------VMTR--R

PGELLHMDI-----VGPAR---VRSAG------GKW----YVLVVVD----DFS------

--RYSW-VF-------FLE----SK---DD-------AF-S---HVHDLVLKLKNELS--

------------NNAVRAIRSDN-GTEFKNS---------RMKVFCAE------------

-----------HGL--DH---QFSSPYVPPQNGVV--ERKNRTLVEMARTML-----DEH

KTPRR-----FWAEAINTACYVANRIF---------------LRAF---LKKTSYEL---

---------------RFG------------------------------------------

------------------------------------------------------------

------------------------------------------------------------

------------------------------------------------------------

--------------------------------------

>ToRTL1

---------------------------------------------------HRR---LGH

V-SL--------SLLNK-LISKD-------------LVRGLPKM---------KFAENKI

---------CEA-CVKGKQ------IR-SSFKPKNQ-VT----------------SS--R

TLELLHMDL-----CGPLK---VQSRN------GKK----YILVIVD----DYS------

--RYTW-TR-------FLR----SK---AE-------TA-D---ELVVFFKMIQRQKQLM

NWWCSSKIQTKLNQVVCSIRSDH-GTEFENS---------TLDRFCME------------

-----------NGT--SH---NFSAPRTPQQNGVV--ERKNRNLVNIARTMI-----IES

NLPQS-----FWAEAVNTACHVTNRCL---------------IRAV---LNKTPYEL---

---------------LNN------------------------------------------

------------------------------------------------------------

------------------------------------------------------------

------------------------------------------------------------

--------------------------------------

>Fourf

----------------------------------------------------SR---FCH

A-SF--------GCLMR-LANIN-------------LIPKFNLV---------KKSK---

---------CHV-CVESKQ------PR-KPHKAAEA-RS-------------------LA

PLELVHSDL-----CEMNG---ILTKG------GKR----YFLTFID----DST------

--RFCY-VY-------LLK----TK---DE-------AF-N---YFKAYKAEVENQLE--

-------------RKIKRLRSDR-GGEFFSN---------LFDEFCVE------------

-----------HGI--IH---ERTPPFSPQSNGIA--ERKNRTLTDLVNAML-----STA

GLSKA-----WWGEAILIACHVLNRVP---------------TKNK---E-ITPFEE---

---------------WEK------------------------------------------

------------------------------------------------------------

------------------------------------------------------------

------------------------------------------------------------

--------------------------------------

>Batata

---------------------------------------------------HKR---LGH

M-SV--------KGIDY-LAKKS-------------KLSGVKEA---------KLDK---

---------CVH-CLAGKQ------RR-VSFMSHPP-TR----------------KS--E

PLDLIHSDV-----CGPMK---VRSLG------GAS----YFVTFID----DYS------

--RKLW-VY-------TLK----HK---SD-------VL-G---VFKEFHALVERQTG--

-------------KKLKCIRTDN-GGEYCG----------PFDEYCRR------------

-----------YGI--RH---QKTPPKIPQLNGLA--ERMNRTIMERVRCML-----DDA

KLPSS-----FWAEAVSTAVHVINLSP---------------VIAL---KNEVPDKV---

---------------WCG------------------------------------------

------------------------------------------------------------

------------------------------------------------------------

------------------------------------------------------------

--------------------------------------

>Sto4

---------------------------------------------------HYR---LGH

I-SR--------GRIES-LVKEQ-------------ILHPLDFT---------DLEQ---

---------CRG-CIKGKF------AK-QI--KKDA-KH----------------ST--R

VLEIIHTDI-----CGPFP---VRTVD------GFN----SFITFTD----DYS------

--RYGY-IY-------PIK----ER---SE-------AL-D---KFKQFKAEVENQHD--

-------------LKIKIVRSDR-GGEYYGRHTEYGQVPGPFARFLRE------------

-----------NGI--VA---QYSTPGEPQQNGVA--ERRNRTLMDMVRSML-----SYS

NLPLG-----LWMEALKTAMHILNRVP---------------SKSV---A-RTPYEL---

---------------WIG------------------------------------------

------------------------------------------------------------

------------------------------------------------------------

------------------------------------------------------------

--------------------------------------

>Tork4

---------------------------------------------------HMR---LGH

M-GE--------RGMQI-LSKED-------------LLAGHKVK---------SLEF---

---------CEH-CVFGKL------HR-NKFPK-AI-HR----------------TK--G

TLDYIHSDC-----WGPCR---VESLG------GCR----FFVSIID----DYS------

--RMTW-VY-------MMK----HK---SE-------AF-Q---KFKEWKILMENQTG--

-------------KKIKRLRTDN-GLEFCWS---------EFDQFCKD------------

-----------EGI--AR---HRTVRNTPQQNGVA--ERMNQTLLERARCML-----SNA

GLDRR-----FWAEAVSTACYLINRGP---------------HTGI---QCKTPMEM---

---------------WSG------------------------------------------

------------------------------------------------------------

------------------------------------------------------------

------------------------------------------------------------

--------------------------------------

>Tto1

----------------------------------------------------RR---LGH

M-SE--------KSMAR-LVKKN-------------ALPGLNQI---------QLKK---

---------CAD-CLAGKQ------NR-VSFKRFPP-SR----------------RQ--N

VLDLVHSDV-----CGPFK----KSLG------GAR----YFVTFID----DHS------

--RKTW-VY-------TLK----TK---DQ-------VF-Q---VFKQFLTLVERETG--

-------------KKLKCIRTDN-GGEYQG----------QFDAYCKE------------

-----------HGI--RH---QFTPPKTPQLNGLA--ERMNRTLIERTRCLL-----SHS

KLPKA-----FWGEALVTAAYVLNHSP---------------CVPL---QYKAPEKI---

---------------WLG------------------------------------------

------------------------------------------------------------

------------------------------------------------------------

------------------------------------------------------------

--------------------------------------

>RTvr2

---------------------------------------------------HRR---LGH

I-SE--------KGLNY-LAKKD-------------VLLGLKNV---------ELEK---

---------CYH-CMAGKQ------TR-VSFKKHPP-S-----------------KT--S

LLELVHSDV-----CGPLK---VKSFS------GAL----YFVTFID----DCS------

--RKLW-VC-------ALQ----RK---DQ-------VL-D---KFKEFHALVERQSG--

-------------KKLKRIRTDN-GGEYCG----------PFDVYCRQ------------

-----------YGI--AH---EKTPPKTPQLNGLA--ERMNRTLIERVRCML-----SEA

KFPKH-----FWGEALFTVVHVINLSL---------------AVAL---NSEVLDKI---

---------------WFS------------------------------------------

------------------------------------------------------------

------------------------------------------------------------

------------------------------------------------------------

--------------------------------------

>Tnt1

---------------------------------------------------HKR---MGH

M-SE--------KGLQI-LAKKS-------------LISYAKGT---------TVKP---

---------CDY-CLFGKQ------HR-VSFQT-SS-ER----------------KL--N

ILDLVYSDV-----CGPME---IESMG------GNK----YFVTFID----DAS------

--RKLW-VY-------ILK----TK---DQ-------VF-Q---VFQKFHALVERETG--

-------------RKLKRLRSDN-GGEYTSR---------EFEEYCSS------------

-----------HGI--RH---EKTVPGTPQHNGVA--ERMNRTIVEKVRSML-----RMA

KLPKS-----FWGEAVQTACYLINRSP---------------SVPL---AFEIPERV---

---------------WTN------------------------------------------

------------------------------------------------------------

------------------------------------------------------------

------------------------------------------------------------

--------------------------------------

>V12

---------------------------------------------------HKR---LGH

I-SE--------KGLQV-LARKK-------------FLP-VKGT---------SLLP---

---------CTH-CLSGKQ------SR-VAFRRFPS-RR----------------KP--D

ILDLVHTDV-----C-TMQ---SNTLG------GAL----YYVTFID----DHS------

--RKVW-AY-------ALK----SK---DQ-------VL-D---VFKDFHVKVERQTG--

-------------KQLKSVRADN-GGEYRG----------PFEQYCRS------------

-----------HGI--RL---EKTVPKTPQQNGVA--ERMNRTICDRIRCML-----SHA

KLPKS-----FWGEAMRTTVDLINLSP---------------SYPL---EGDIPERV---

---------------WTG------------------------------------------

------------------------------------------------------------

------------------------------------------------------------

------------------------------------------------------------

--------------------------------------

>Humnum

---------------------------------------------------HRR---LGH

V-NS--------QYLNK-M--QD-------------AVQGLTLD---------RKTDISK

------SS-CVA-CCEGKQ------SR-LPFPK-EG-SR----------------ST--R

LLHIVHSDL-----CGPME---NRSIG------GSR----YFMLFID----DFS------

--RMTY-IY-------FLK----TK---DE-------AL-K---CFQQYKAEVENQLN--

-------------CSIKILRSDN-GLEFCNS---------KFDDFLMS------------

-----------HGI--VH---HKTNPYTPEQNGLS--ERFNRTIVEKAKCLL-----FDA

GLEKR-----FWAEAAHTAVYLQNRTV---------------TTSL---NYKTPFEV---

---------------WTG------------------------------------------

------------------------------------------------------------

------------------------------------------------------------

------------------------------------------------------------

--------------------------------------

>Mtanga

-------------------------------------------------ASCKR-----H

R-DP--------NAIQR-VAREG-------------LAKGISIK---------KCDIF--

------QT-CEC-CVEGKI------AR-KPFPPITE-RQ----------------TT--R

VLDLVHTDI-----CGPMN---TVTSG------GSR----YFLTMID----DFS------

--RYTT-VY-------FLK----RK---SE-------AA-E---VIEEYVTMVHNRFG--

-------------RNPIVIRSDQ-GGEYKSK---------RLGQFYRA------------

-----------KGI--VP---QFTAGYSPQQNGVA--ERKNRTLVEMARCML-----IDA

KLGYR-----FWAEAINAAVYLQNISS---------------SRSI---E-KTPFEL---

---------------WYG------------------------------------------

------------------------------------------------------------

------------------------------------------------------------

------------------------------------------------------------

--------------------------------------

>Copia

---------------------------------------------------HER---FGH

I-SD--------GKLLE-IKRKN-------------MFSDQSLL---------NNLELSC

------EI-CEP-CLNGKQ------AR-LPFKQLKD-KTH---------------IK--R

PLFVVHSDV-----CGPIT---PVTLD------DKN----YFVIFVD----QFT------

--HYCV-TY-------LIK----YK---SD-------VF-S---MFQDFVAKSEAHFN--

-------------LKVVYLYIDN-GREYLSN---------EMRQFCVK------------

-----------KGI--SY---HLTVPHTPQLNGVS--ERMIRTITEKARTMV-----SGA

KLDKS-----FWGEAVLTATYLINRIP---------------SRALV-DSSKTPYEM---

---------------WHN------------------------------------------

------------------------------------------------------------

------------------------------------------------------------

------------------------------------------------------------

--------------------------------------

>Koco

---------------------------------------------------HER---LGH

I-SN--------GKLLE-IKRQN-------------LFSDSNLL---------KNLEITD

------GI-CES-CLSGNR------LD-CIWKS-KD-KTY---------------VK--R

PLFIIHSDV-----CGPIT---PVTLD------EKN----YYVIFVD----QFT------

--HYCV-TY-------LLK----YK---SD-------VF-S---MFRDFVAKSEAHFN--

-------------CKIVNLYIDN-GREYLSN---------EMRAFCVE------------

-----------RGI--TY---HLTVPHTPQLNGVS--ERMIRTITEKARAMV-----NGA

KLDKS-----FWGEAVLTATYLINRTP---------------SRALD-DNRKTPYEM---

---------------WHN------------------------------------------

------------------------------------------------------------

------------------------------------------------------------

------------------------------------------------------------

--------------------------------------

>Yokozuna

---------------------------------------------------HKR---LGH

A-NR--------KHLKL-LK----------------------------------------

-LPISEKP-CGI-CVEGKS------TR-LPFSTTPK-PR----------------SK--Y

IGELIHTDI-----SGPIN---IPTLT------NEV----YFHTIID----DYT------

--HFCE-VY-------LLQ----RK---SE-------AT-D---RLIEYVNRMERQIE--

-------------CKVKKIRSDN-GGEFKNE---------KLNKFCKD------------

-----------KGI--LQ---QFTLPYSPQSNGVS--ERMNRNIYNRARTLL-----IES

GLPKT-----LWGEAVRCAVYQTNRCP---------------SSNF-----QTPAEK---

---------------MFG------------------------------------------

------------------------------------------------------------

------------------------------------------------------------

------------------------------------------------------------

--------------------------------------

>Tricopia

---------------------------------------------------HRR---LGH

P-NM--------TYVTS-AIRNG-------------YLKGVEIK---------NRED---

------FE-CSV-CVKGKM------AR-TPFPK-KS-NR----------------KT--S

TLELIHSDV-----CGPMR---TQSLG------GAK----YYVEFID----DAT------

--RWCE-VR-------FLR----NK---SD-------VF-K---ATADYINLIENQIG--

-------------KSVKCLQSDN-GTEYTNK---------ELDEYLKK------------

-----------RGI--SR---RLTAPYNPEQNGVS--ERKDRTLLDTARCLL-----MES

KLPSS-----FWAEAVNTANYLRNRLP---------------TKSL---NGRTPYEA---

---------------WTG------------------------------------------

------------------------------------------------------------

------------------------------------------------------------

------------------------------------------------------------

--------------------------------------

>Hydra12

---------------------------------------------------HEI---LGH

C-NY--------EDISK-L--QN-------------VVKGMKIT---------GKIDKSN

------LN-CKI-CTQGKF------VQ-SRNREPDT-RA-------------------KA

ALELVHTDL-----AGPID---PEAKD------GFK----YALAFTD----DYS------

--GAVF-VY-------FLK----AK---SD-------TA-R---ATEKFIADTAPYGR--

---------------IKCVRSDN-GTEFTAK---------EFQSLLSK------------

-----------NGI--RH---ETSAPYSPHQNGTA--ERNWRTLFEMARCMI-----LES

GLPKM-----LWTYAVMTAAVIRNRCY----------------NSR---IHQTPYYA---

---------------LTG------------------------------------------

------------------------------------------------------------

------------------------------------------------------------

------------------------------------------------------------

--------------------------------------

>Hydra11

---------------------------------------------------HKI---MGH

C-NT--------NDIIK-L--ED-------------VAQGMKIN------------NIAK

------FD-CET-CILSKN------VN-TRNQQPDT-RA-------------------TY

PFQLVHTDL-----AGPIE---PVAIG------GFK----YVINFVD----DYS------

--SCLF-TY-------FLQ----QK---SD-------AA-K---AAEKFLADIAPYGKVK

TF--SFYNDISPSGNVKCIRSDN-GGEYLSK---------EFNELLLK------------

-----------HTI--KH---EFTSPYSPHQNGTA--ERNWRSLFDMARAMI-----IES

KLIKH-----LWTYAISTATYIRNRCY---------------VQQR---IKSTPYGL---

---------------VTG------------------------------------------

------------------------------------------------------------

------------------------------------------------------------

------------------------------------------------------------

--------------------------------------

>1731

---------------------------------------------------HKR---NGH

L-NT--------SSLQE-MVRKK-------------MVYGVEKV---------VFKPDAV

---------CKT-CMLAKI------HV-QPFPKTTR-SR----------------AE--E

LLDMIHSDL-----CGPFS---TPSLA------GSK----YFLTFID----DKS------

--RRIF-VY-------FLR----KK---DE-------VF-T---KFVEFKKLVERQTG--

-------------RKIKCIRSDN-GGEFVNN---------VFDDYLKA------------

-----------HGI--AR---QLTIPHTPQQNGVA--ERANRTLVEMARCML-----LQS

ELGEA-----LWAEAINTAVYLRNRST---------------SRAL---QSKTPMEE---

---------------WTG------------------------------------------

------------------------------------------------------------

------------------------------------------------------------

------------------------------------------------------------

--------------------------------------

>Xanthias

---------------------------------------------------HNR---FGH

L-NF--------QCLKE-IKEKE-------------LVIGMDFK---------NMSVNIN

---------CDT-CNMAKI------HV-LPFPQNSE-RA----------------TQ--S

VLELVHSDV-----CGPMN---VSSLG------GNK----YFVTFID----DYS------

--RKIF-IY-------FMH----AK---NE-------VF-D---KFKLFKSYVECQTG--

-------------KKIKALRSDN-GTEYVNR---------QFTEYLNT------------

-----------CGI--KR---QLTVPYTPQQNGVA--ERANRTIVEMAKSML-----IHA

KLEEF-----LWAEAVSTASYLRNRCP---------------SKAL---MGATPFEI---

---------------WQN------------------------------------------

------------------------------------------------------------

------------------------------------------------------------

------------------------------------------------------------

--------------------------------------

>pCretro6

---------------------------------------------------HRI---LGH

A-HL--------ASIKR-LCL-G-------------LAEGVDVD---------MTSDDQF

-------Q-CES-CVRAKQ------HV-QPFPDQAT-RT----------------PDKLK

VGEIVASDT-----WGPAQ---VRSVH------SFY----YYMSMTD----LKS------

--RFSG-VY-------FSA----QK---SGLA---KMAL-T---EFRGLIRRMSRT-G--

-------------SSIMCLRIDN-GTEFINN---------EFLQYCKS------------

-----------QGI--VV---ETTAPYSPAQNGVA--ERLNRTLRESARAML-----LAA

ELPRK-----FWPEAVSYACLIKNRLP---------------HAAL---KGMTPYQA---

---------------LTG------------------------------------------

------------------------------------------------------------

------------------------------------------------------------

------------------------------------------------------------

--------------------------------------

>pCretro3

---------------------------------------------------HCA---LGH

I-NA--------TQLQE-MYRKG-------------LVEGMDVD---------TSSDPGF

-------V-CDA-CIQAKH------SR-APFPEIAS-GT----------------VD--Q

VADLIYSDI-----WGPAR---VASLQ------GNV----YAITFTD----AKS------

--RFVA-VD-------FMK----TR---DA-------AL-D---RFQKVEQLIERQLG--

-------------RRVKVLHVDN-AKEYTEG---------KFRAYAES------------

-----------RGI--II---RTTAPYSPAQNGVA--ERLNRTLMERARAML-----IAR

SLPKF-----LWQEAWAYACYLRNRTP---------------TRAL---SGKTPYEA---

---------------LWG------------------------------------------

------------------------------------------------------------

------------------------------------------------------------

------------------------------------------------------------

--------------------------------------

>PyRE1G1

---------------------------------------------------HRR---FFH

L-GY--------NNLQR-AAK---------------MVDGLPEK---------EVETESA

----AGAI-CRP-CVEGKT------VK-APFPASNS-KT--------------------D

VMELIHVDI-----TGPFK----KSPS------GSR----YLIVLYE----DST------

--GMTL-AV-------PIR----AK---SE-------AG-R---VMRGKIPEWERRSG--

-------------KRLKRIRFDG-AKEFTTA---------KLRQWYEE------------

-----------KGI--DY---EVTLPYSPQSNGKA--ERVNRTIKERVRAAL-----SES

GLDEE-----LWAEAAVAAAYVMNRSP---------------KEG----QDVTPWEG---

---------------FTG------------------------------------------

------------------------------------------------------------

------------------------------------------------------------

------------------------------------------------------------

--------------------------------------

>Osser

---------------------------------------------------HSR---FGH

L-SY--------GNLAR-LADEE-------------MVAGLDV------------TPKKF

RE-VGAAV-CEG-CVMGKH------SR-KPFPESHS-SV----------------G---R

ALERVHMDL-----AGPMS---VESYG------GNS----YVATFLD----EYT------

--GLSV-VS-------FLK----LK---SE-------AI-A---VVKSVLTLLENQCG--

-------------QKVKEVRTDR-GREYLNA---------ELREFYGE------------

-----------RGI--VH---QTSAPYTPQQNGIA--ERLNRTLMEKVRCML-----IES

GLPSR-----GWALAMHTANYLRNRSP---------------VHGL---KG-TPFER---

---------------FYG------------------------------------------

------------------------------------------------------------

------------------------------------------------------------

------------------------------------------------------------

--------------------------------------

>CoDi6.1

---------------------------------------------------HYR---LGH

L-----------PFPRL-KLLAENGEI-----------PK-------------RLAKV--

----IPPR-CAG-CLFGAM------TK-V-PWRAKG-KQ---DTTI-----FSATKA---

-GQVVSVDQMISTQVGFI----AQLKGRLT-TQRYR----AATVFVD----HFS------

--RLKF-IY-------LMT----SL-S-SE------ETV-AAKRAFERFASNNG------

-------------VRIQQYHCDN-GR-FADK---------AFISHCEQ------------

-----------QQQ--CI---TYCGVNAHFQNGIA--EKAIRDIQEQARKQLLH---ARS

RWPEV-IHLALWPYALRMAVHLHNTVP---------------SLA----DGRSPLEV---

------------FAS---------------------------------------------

------------------------------------------------------------

------------------------------------------------------------

------------------------------------------------------------

--------------------------------------

>CoDi6.7

---------------------------------------------------HHR---LGH

A-----------SMTKI-RMLAKVGLL-----------PA-------------SLKEC--

----QIPL-CTS-CLYGKA------TR-R-PWRTKG-SS---TAST-----RSIHKVN-G

PGQCISVDQLVSTTPGYI----AQLRGRPT-LKRYH----AATVFVD----NFS------

--RLSY-VH-------VQK----GT-S-AE------ETI-QAKHAFERYARSHG------

-------------VVTKHYHADN-GI-FADN---------KFREAVKE------------

-----------DRQ--TL---SFCGVNAHFQNGIA--ERRIRELQDHARTMLIH---ATK

RWPDA-IDSHLWPYALRMANELHNHLP---------------SLR----GSVPPIEI---

------------FAQ---------------------------------------------

------------------------------------------------------------

------------------------------------------------------------

------------------------------------------------------------

--------------------------------------

>CoDi6.6

---------------------------------------------------HQR---LGH

V-----------SFPHL-QELYREHQVECDFDGC-STGPSTAANSACLANRSPSLSSC--

----DPPL-CLA-CQASKA------CR-R-PSGSKH-SRPDPDTSG-----TLSFDVS-R

PGELVSVDHYESAIRGRL----VSTRGRESETNKHC----GGTIFYD----HYS------

--SFVS-VH-------HQP----TL-G-AT------ETI-KSKRAFEIQALGCG------

-------------NVLEKFRTDN-GI-FTSK---------LWLDSLMS------------

-----------ANQ--FQ---SLSGVGAHHQNGVA--ERAIQTVTLMARSMLLH---MAL

HWPDQ-FSEDLWPFALDYSAFIHNHMP---------------KRS----QGLAPIEL---

------------FCG---------------------------------------------

------------------------------------------------------------

------------------------------------------------------------

------------------------------------------------------------

--------------------------------------

>CoDi6.4

----------------------------------------------TLEQAHKL---CGH

A-DV--------ATVKR-TAVRAGW-----------SLT---------------------

--TTTMPL-CGA-CVLAKA------QQ-KQVPKTAS-QK----------------AE--K

PGERLYVDL-----SGPYA----KSIG------GST----YWLLIVD----EVS------

--KYKW-SM-------FLT----QK---SQ-------IG-Q---KVKP-ILQWLQLLK--

-------------FTNKFIRSDN-AGENKK----------HFEKLAEE------------

-----------FLF--SL---ELTAPYTPQQNGVV--ERGFVTIRNRAYASM-----LDA

KLTPSNQGK-LWTEFVQAATVLSNNLV---------------RAG----ETKTPTEL---

---------------FFG------------------------------------------

------------------------------------------------------------

------------------------------------------------------------

------------------------------------------------------------

--------------------------------------

>CoDi6.3

---------------------------------------------------HCA---LGH

P-CE--------ATTRA-TAKAFGV-----------RL----------------------

--IGQMKP-CKD-CALSKA------KA-KKISKVPV-KR----------------AS--K

PGGRLCIDI-----SSPST----KSVG------GKC----HWLLVVD----DCT------

--DYAW-SF-------FLN----KK---SE-------TN-D---IMIALIKELKQAYD--

-------------IDVKTIRCDN-SGEN-N----------ALQRSCKQ------------

-----------EGLGITF---EYTAPNTPQQNGRV--ERRFPTLYGRVRAML-------R

DVSVSINNKRLWAEAANTATDLDNMLL---------------KQG----ETTNSFHK---

---------------FFG------------------------------------------

------------------------------------------------------------

---------------------------------------------------------K--

------------------------------------------------------------

--------------------------------------

>CoDi6.2

---------------------------------------------------HEE---LGH

P-NM--------VVTRS-TAKARHE-----------NV----------------------

--VGPIQQ-CED-CAVGKA------HQ-KRVPKQPV-AR----------------AK--N

PGERLFLDI-----SHPKQ----QSIG------GSN----DWILVAD----DAT------

--DNCW-SW-------FTR----RK---DQ-------LS-D---VIVPFVIDLKATYG--

-------------ITVKCIRCDN-SGEN-H----------LLERRCNQ------------

-----------EGLGIKF---EYTAPNTPQQNGRV--ERKFQTLYGRVRAML-----VGS

GIKQPLRNK-LWAEAANTATMLDNELV---------------KEG----ETLTSHQK---

---------------FFG------------------------------------------

------------------------------------------------------------

---------------------------------------------------------K--

------------------------------------------------------------

--------------------------------------

>CoDi6.5

---------------------------------------------------HDK---LGH

K-GE--------VLLKK-TLKHHSI-----------KV----------------------

--TGELQS-CEG-CCLAKA------KQ-KSVSKTTN-IR----------------AK--S

PGERLFVDM-----SGPFT----ATIL------GSR----YQIQVVD----DAT------

--RKGF-IG-------FVK----KR---SD-------LS-Q---WLEENVLSKLEGMQ--

-------------KQTKYLRADN-AGENKN----------PLEKLCDK------------

-----------KGM--TL---ELTAPDTPQQNGVV--ERRIAVLQQRANAMM-----MAA

DLTPEARAL-LWAEALNTANDLENISL---------------NTM----SDKSPDKL---

---------------FSN------------------------------------------

------------------------------------------------------------

------------------------------------------------------------

------------------------------------------------------------

--------------------------------------

>Tse1

---------------------------------------------------HRL---FGH

I-NI--------KTIKE-SISNK-------------LIKNISLN---------DVDWSHI

----DKFQ-CTD-CMKGKA------TK-HKHIVGSR-LKY---------------QNNYG

PFQYIHSDL-----FGPVT---GVSAT------SPS----YFISFTD----ECT------

--RFRW-VY-------PLR----TKS--AE------SIY-N---IFDHLVRQIDTQFN--

-------------TKILSFHMDR-GSEYTNT---------EMQMFFQK------------

-----------HGI--IP---IYSSTTDSSSNGVA--ERSNLTFLNDCRTLL-----VSS

HLPNS-----LWFNAVEFATLMRNAFI---------------NST----NKMSPRGK---

---------------AGL------------------------------------------

------------------------------------------------------------

------------------------------------------------------------

------------------------------------------------------------

--------------------------------------

>Ty1B

---------------------------------------------------HRM---LAH

A-NA--------QTIRY-SLKNN-------------TITYFNES---------DVDWSSA

----IDYQ-CPD-CLIGKS------TK-HRHIKGSR-LKY---------------QNSYE

PFQYLHTDI-----FGPVH---NLPKS------APS----YFISFTD----ETT------

--KFRW-VY-------PLH----DRR--ED------SIL-D---VFTTILAFIKNQFQ--

-------------ASVLVIQMDR-GSEYTNR---------TLHKFLEK------------

-----------NGI--TP---CYTTTADSRAHGVA--ERLNRTLLDDCRTQL-----QCS

GLPNH-----LWFSAIEFSTIVRNSLA---------------SPK----SKKSARQH---

---------------AGL------------------------------------------

------------------------------------------------------------

------------------------------------------------------------

------------------------------------------------------------

--------------------------------------

>Tkm1

---------------------------------------------------HNM---FGH

M-NI--------NYIRE-SFRKG-------------LIQGVKED---------DVDWTGV

----SSFQ-CQH-CMEGKA------KR-NNHYVNAR-KDY---------------TKEYL

PFEYLHTDV-----FGPVR--VQRTRT------TPR----YFIAFID----EVT------

--KYIW-TF-------PLL----HKT--AE------EVA-P---TFKEVVMLIYTQFN--

-------------TRVKTIQMDK-GSEYLNT---------KVQKFLRE------------

-----------RGI--VS---RETTVADSKANGAI--ERQHYTLLNDCRTFL-----RQA

NLRPR-----LWYHAVVYSTVMRNSLL---------------NRS----IGTSPRNR---

---------------AGM------------------------------------------

------------------------------------------------------------

------------------------------------------------------------

------------------------------------------------------------

--------------------------------------

>pCal

---------------------------------------------------HLM---SNH

M-SI--------EKILL-LQKYQGL-----------VLH---TS---------KESLQKI

------AD-CKV-CLLSNA------KQ-RSHNHHSE-RK----------------AS--R

RHERLHCDT-----LGPFR-----SEN------NKW----YLTSVID----EHT------

--GYIE-GI-------ITK----DR-----------KVK-D---LLIQRLKIWNNRFN--

--------D-----KVAYFRSDN-APEFPQPS--------DLAEF---------------

------------GI--WR---ETIAAYSPELNGLA--EVVNKLILQQIYRIV-----VTL

-GPQILK---LIYYVIQYSITMINHTP---------------RRSL---KGQTPYGC---

---------------YYQ------------------------------------------

------------------------------------------------------------

------------------------------------------------------------

------------------------------------------------------------

--------------------------------------

>Ty2

---------------------------------------------------HRM---LGH

A-NF--------RSIQK-SLKKN-------------AVTYLKES---------DIEWSNA

----STYQ-CPD-CLIGKS------TK-HRHVKGSR-LKY---------------QESYE

PFQYLHTDI-----FGPVH---HLPKS------APS----YFISFTD----EKT------

--RFQW-VY-------PLH----DRR--EE------SIL-N---VFTSILAFIKNQFN--

-------------ARVLVIQMDR-GSEYTNK---------TLHKFFTN------------

-----------RGI--TA---CYTTTADSRAHGVA--ERLNRTLLNDCRTLL-----HCS

GLPNH-----LWFSAVEFSTIIRNSLV---------------SPK----NDKSARQH---

---------------AGL------------------------------------------

------------------------------------------------------------

------------------------------------------------------------

------------------------------------------------------------

--------------------------------------

>Tdh2

---------------------------------------------------HLM---GNH

M-SL--------ESMKY-LIKSGHI-----------KMSSEITA---------SEE-ERV

------KS-CNE-CLAINS------KQ-SSHN-HTH-FT----------------AP--R

RLFRLHSDT-----LGIFS-----HRG------KKY----YITTLID----EYS------

--GYLN-TI-------WSE----HK-----------SIQ-D---LLFEKIRIWNNKFV--

--------D----ANVAFFRTDN-ALEMPTKD--------QLAEI---------------

------------GI--EK---DEIASYSPELNGIS--ERTNRSIIQFIRKAL-----LPI

QDTRTLY---LLPKIVDYVTYIRNMTP---------------VRSK---GGSCPYA----

------------------------------------------------------------

------------------------------------------------------------

------------------------------------------------------------

------------------------------------------------------------

--------------------------------------

>Ty4

---------------------------------------------------HKR---MGH

T-GI--------QQIEN-SIKHNHYE---------ESLDLIKEP---------NEFW---

---------CQT-CKISKA------TK-RNHYTGSM-NNH---------------STDHE

PGSSWCMDI-----FGPVS---SSNAD------TKR----YMLIMVD----NNT------

--RYCM-TS-------THF----NKN--AE------TIL-A---QVRKNIQYVETQFD--

-------------RKVREINSDR-GTEFTND---------QIEEYFIS------------

-----------KGI--HH---ILTSTQDHAANGRA--ERYIRTIITDATTLL-----RQS

NLRVK-----FWEYAVTSATNIRNCLE---------------HKS----TGKLPLKA---

---------------IS-------------------------------------------

------------------------------------------------------------

------------------------------------------------------------

------------------------------------------------------------

--------------------------------------

>Zeco1

-----------------------------------------------LLKLHKQ---FGH

A-SA--------DRLQR-LMHSSGNKD----KECFAILQQI-------------------

-----VDD-CEI-CKKYKR------TK----------------------------PK---

----------------PAV--------------PSI----WYLHIID----HFT------

--RFSA-GS-------IVK----TKKS-SE-------IV-NS--FIHTWISVHG------

--------------APQKLYSDN-GGEFNNQ---------EIRDMAEN------------

-----------FNI--EI---RTTAGYSPWSNGLL--ERHNQTLTEIILKVK------EN

GC--------DWHTALDWALMAKNSML-----------------------NVSPYQL---

---------------VFG------------------------------------------

------------------------------------------------------------

------------------------------------------------------------

------------------------------------------------------------

--------------------------------------

>GalEa1

-----------------------------------------------ALKLHRQ---FGH

P-TS--------MKLNK-LISDAGVKD----SGLRKAVEMV-------------------

-----FRE-CKV-CCKLKK------AK----------------------------PH---

----------------PIV---------------NC----YFLVIVD----LAT------

--RYCT-AT-------VIK----DKCA-NT-------II-KA--LFLKWIV---------

--------------NVWRTWEDT-YGQW-AL---------IMRTLGEA------------

-----------LNV--KI---MTTSAESPWSNGAC--EKLNGVIGDMVRKIM-----ADN

KC--------DLEVALAWAVSARNALT---------------NY----------------

------------------------------------------------------------

------------------------------------------------------------

------------------------------------------------------------

------------------------------------------------------------

--------------------------------------

>Cico1

-----------------------------------------------IEKLHKQ---FGH

A-SV--------DSLVQ-LLKNADNWD----KAYLSLIQNT-------------------

-----VRN-CNT-CKIHKR------PV----------------------------SR---

----------------PIV--------------PSI----YYFHMID----MFS------

--RFSS-AV-------IIK----SKCA-NV-------II-KH--FLKHWICI--------

------F------GCPKVVLSDN-GGEFNND---------EFRDMCDN------------

-----------FNI--KC---MTTAAYSPWSNGLC--ERHNLTLNEIIRKIK-----FDL

NC--------DYDVALSWALYAKNSLV---------------NN----------------

------------------------------------------------------------

------------------------------------------------------------

------------------------------------------------------------

------------------------------------------------------------

--------------------------------------

>Zeco2

---------------------------------------------------HKQ---FGH

A-SA--------DRLQR-LMHSSGNKD----KECFAILQQI-------------------

-----VDD-CEI-CKKYKR------TK---------------------PKPAVGLPMASE

YNETVAVDL---HELKPSI---------------------WYLHIID----HFT------

--RFSA-GS-------IVK----TKKS-SE-------IV-NS--FIHTWISVHG------

--------------APQKLYSDN-CGEFNNQ---------EIRDMAEN------------

-----------FNI--EI---RTTAGYSPWSNRLL--ERHNQTLTEIILKVK-----REN

GC--------DWHTALDWALMAKNSML---------------NVH-----GYSPHQL---

---------------VFG------------------------------------------

------------------------------------------------------------

------------------------------------------------------------

------------------------------------------------------------

--------------------------------------

>Olco1

VIDNDHILIANENVIQQGNTNEELGSQYEEEILAISENMTTAEKQKILMKLHQQ---FGH

A-SV--------ERLQK-LLMSSG------------------------------------

------------------------------------------------------------

----------------------------------------WYLHIVD----QFT------

--RFSA-GS-------ILT----TKKS-SE-------IV-KH--FIHAWISVHG------

--------------PPQKLFSDN-GGEFNND---------EMRDMAEN------------

-----------FNI--EI---KTTAAYSPWSNGLM--ERHNQTLTEILMKVK-----TSN

TC--------DWDTALDWALMANNTMQ---------------NVH-----GFSPYQL---

---------------VFG------------------------------------------

------------------------------------------------------------

------------------------------------------------------------

------------------------------------------------------------

--------------------------------------

>Cer7

---------------------------------------------------HGKY----H

T-NE--------QQTME-ATREK------------AWIPCLRR----------QVKKI--

-----IGK-CVK-CQRYNR------APMKYPNMAD--MPSFR-------------VRRSR

PFENTGLDY-----FGPMT---FRKED---GSTESC----WGCVLSC----ATT------

--RLTH-I--------ELV----QQCS-TKAF-----IN-----AIRRFVSERG------

--------------IPDRIVSDN-APQFLG-Q-------QILNEVSAR--ASKENALDKD

ILEYL----GNTSI--EW---SFITPYAPWQGGMY--ERMMRSIKQAIYKSI-----GKN

IL--T---LDDLETVMKEVSGIINSRPLTYV-----------TEGAGT--TICPKDF---

---------------INP-----EMR----------------------------------

---------------MTIPLQVTKDMMDE----YIPPEEINLTKQETIEALQS-------

---SVAIIEDI----WKIWN--------KTYLS-ELRETHKL---------------RMD

NK-REI-------------ND-SPFVGQVV--------LICD-PNLPR----NNWKMGKI

TETKPS----TDGILREAHL------------------

>Mabel

---------------------------------------------------HEQLY---H

A-GI--------AHTLS-NLRST------------IWIPKGRT----------EVKRI--

-----LNK-CMK-CRRWTT------KPFKLPTMAD--LPASR-------------TTRSR

PFARVGLDY-----LGPVN---IRSDN----GLTKR----WVALFTC----FTT------

--RAVH-L--------EVV----ETLS-AESF-----LH-----VFRRFTARRG------

--------------FPELILSDN-AGQFQLIF-------KII-----------VKQQLNE

FL-------AERKM--IW---KNIIPKAPWNGGVY--ERLIGLTKRAMKRAI-----GRK

LL--W---ERELITLVAEVESILNTRPLT-------------YVNFDDCIILRPIDF---

---------------ILP-----NAH----------------------------------

---------------LIMPAKNKNEMDDF----IPHKLDSREKLIQYWSNTL--------

-----KALDAF----WEIWK--------EEYLN-TLKEAQRE---------------HSS

P--RDV-------------VKRTPHEGEIV--------LLNE-PEIPR----GMWKIARI

REIKTGK----DGEVRSVSI------------------

>Tas

---------------------------------------------------HEELL---H

A-GI--------SSILA-KMREA------------YWIPRGRQ----------AIKRA--

-----LNN-CFH-CRRWKS------KPFQMPPMPP--YPPER-------------VSKHP

PFENTGVDY-----IGPFT---IRSMK----RITQN----DGSVYSL----VS-------

-----Q-L--------ELY----ISKS-PLTYPDQVSFS-----VYVRFVSRRG------

--------------LPKRMLSDN-GTQFVWAR-------SVLT----S--VSKVHQTDNA

ILDYC----AAHNI--QW---SFITPLVPWQGGIY--ERMVGPVKGSMKKTI-----GWK

RL--T---QEELQTLTTDIEAVVNCRPII-------------PLTSENTTVLRPVDF---

---------------LLP-----HGNS--SPGVFR-------------------------

--------------SLENDAREPTYRD-------PS--DIHQKLLKYWEETR--------

-----AKLDNF----WTIWR--------EEYLT-M-------------------------

------------------------------------------------------------

--------------------------------------

>Cer101

---------------------------------------------------HEING---H

L-PE--------QYTLS-ALKTM------------YWIPLCKA----------LVRSV--

-----ISN-CIK-CKKVFG------LPYPYPNTKQ--LPGCR-------------TEPSK

PFAKVGLDY-----LGPVE--YLRDDQ---ESIGKA----YVLLYTC----LVT------

--RAAV-L--------RVV----PDAT-TESY-----LM-----ALRTIFHQVG------

--------------VPSEVHSDN-AAIFKLGA-------KMTNDDIRE--GSEIDEIFTC

FL-------ASQEI--KF---IYITPWSPWQGGVY--ERIIGLLKHQIHKIC-----GDQ

KL--D---FFSLQYVVSSAQAMINNRPLVAH-----------ARSPNDMITLRPMDF---

---------------MIP-------------GV---------------------------

---------------MIETPRTPA--DS------PTTSTTETRTRAHLEKFE--------

-----SALERL----WTIWT--------FGVML-ILREVSHK---------------HKR

C--CDL----------------KPEVGDVV--------IINP-NNVSR----HRWPLALV

VQVNQSK---RDGEIRTAVVR-----------------

>Spirobel

---------------------------------------------------HLRQL---H

G-GC--------EFTLA-TLRQR------------YWILKGRR----------EVKKV--

-----IHA-CSS-CKRIES------RP-FVAKMAP--LPSDR-------------TRVTR

PFENTGLDI-----AGPFF---TR----QGKKVNKN----YICLFTC----MST------

--RAVH-L--------EVV----SEMT-APRL-----LQ-----ALRRFIARRG------

--------------RPHILQSDN-FKSF-----------KQLDKDLSQ--LVSTEMIDN-

VAREL----TSHRI--QW---NFITERAPWMGGYW--ERLIRSMKTSLKKVL-----QNS

ML--E---DEEFRTIISEVEARMNSRPLTYN-----------PDNPNNPEVLTPYHF---

---------------LTG-----THY----------------------------------

---------------TDIPEVTKD--EDE----WVPKAQSTSHLMKNWNLRQ--------

-----RLIAQW----WKRWK--------TEYVT-NLNVRQKW---------------YNS

G--NAP-------------N-----IGDIV--------LVSE-NNVPR----RNWKLGKI

VQLYPG----QDGIVRTVKV------------------

>Hydra31

---------------------------------------------------HVKLV--KH

N-GV--------KETIN-ALRSQ------------YWIPCCRQ----------LAKSV--

-----IRE-CTT-CRRIEG------RPYSYPPAPP--LPKSR-------------LNSDF

AFKSIALDY-----AGPLY---IKDIY-GDCTLNKA----WIFLFTC----CSS------

--RSIL-L--------DLV----ADCS-SSSC-----IM-----GIRRFIGRRG------

--------------VPEIIYSDN-GSQFVSTE----------------------------

TQYFA----ANHSI--KW---KFNAPSAPWWGGMF--ERLVRMTKRCLKKAL-----KNS

KS--S---YEEVRTLLSEIDMVLNNRPLTYL----------YNNQGDE--ALTPNHL---

---------------VFG-----HKL----------------------------------

---------------KLESSLSA-------------CNDIEQDVHIRNSMLS--------

-----NTLNHF----RSRFK--------SEYLT-ELREYHKS---------------KRS

NGCNGI-------------R-----VNDIV--------LIEN-DNCKR----QLWKLARV

EELLYS----DDGVVRVAKV------------------

>Ninja

---------------------------------------------------HDRMK---H

Q-NV--------DATIA-EIRTK------------FWVTKMRR----------VMRKS--

---------HLI-VQRVQV------AA-TATDAADNGTPSGR------------------

---QTGCGW-----MAIQI-----------HRTGLL----WATAGDC----VPSQ---GE

AGRLVY-VFDDKGDSPGVA----HDLS-TDSC-----II-----AIRNFVCRRG------

--------------PVYRLRNDN-GKNFVGADRE----ARRFGDVFEM----------EK

LQSEL----SSRSI--EW---VFNCPANPSEGGVW--ERMVQCVKRVLRHTL-----KEV

AP--R---DHVLESFLIEAENIVNSRPLTHL-----------PVDADQEAPLTPNDL---

---------------LKG-----VANLPDTPGLD--------------------------

---------------AECPRRVLRESSGG----LLACSETVSGGGGSWSTCLRLCAARSG

AAERSPSTRVI----WS-------------------------------------------

-------------------------SSAIL--------PWPD-ESGAR----ASWRRSTA

ELMESS------DALRCA--------------------

>Tamy

---------------------------------------------------HRRFC---H

A-NH--------ATVVN-ELRQT------------YWILSLRD----------AVKKV--

-----LHQ-CQW-CRTRKM------KP-QMPPTGD--LPVER------------LRYGSH

PFTCTAVDY-----FGPMF---VTIGR----RKEKR----WGALFTC----LTT------

--RAVH-L--------ELV----PSLS-TSSM-----IM-----ALRRMSARRG------

--------------TPRVIYSDN-GTNFIGAN-------HELREEIGK--LKKNE-----

LIDAA----NQEGI--RW---KFIPPGAPNMGGAW--ERMVRTVKTALSAIL-----NER

SP--P---EEVLHTLLTEVEHTVNSRPLTHL-----------SVNPEDEESLTPNHF---

---------------LIG-----RSC----------------------------------

----------------GSAILGT-------------FNEYNLIGKADWKTTQ--------

-----HLADHF----WKRWL--------REYVP-TLMPRKIE---------------GKE

V--RQP-------------Q-----PGDIV--------IIAD-ASLPR----NTWPRGEV

IACHPG----PDGRTRVVDV------------------

>Cubel

-----------------------------------------------------RYH---H

L-NH--------ETVVN-ELRQK------------YRIPQLRR----------ACFAV--

-----RAS-CQD-CKNRYA------RP-SPPLMAE--LPPAR------------LAAFTR

PFSYTGVDY-----FGPIS---VAVGR----RVEKR----WGVLLTC----LVT------

--RAVH-I--------EIA----HTLT-TDSC-----IL-----ALRNFMAIRG------

--------------TPLELISDQ-GTNFIGAD-------RELKKAYQK--VDQNQ-----

LIREF----TTTNT--KW---TFNPPSSPHMGGSW--ERLVQSVKKVLNQMK-----LPR

NP--S---DEVLRNTLLEISNIVNSRPLTYI-----------PVEDDNTPALTPNHF---

---------------LLG-----SSS----------------------------------

----------------GSKPLVA-------------FDNGHTALRNNWKASQ--------

-----IYANLF----WKKWV--------KEYLP-TICRRTKW---------------HQP

V--KPI-------------Q-----VGDVV--------VIAD-PDHPR----NSWPMGRV

VSTNTS----KDGQVRSAVV------------------

>Zebel

---------------------------------------------------DQRLL---H

P-GS--------ERVLA-ELRRQ------------YWVLRGRE----------AIRKH--

-----QHT-CRD-CQFWRA------KP-QTPQMAD--LPSSR------------LQLYKP

PFYSTGVDC-----FGPFT---VKVGR----RQEKR----WGVLYKC----MTT------

--RCVH-L--------DLL----EQLD-TDAF-----LL-----SLRRFIARRG------

--------------KPMELLCDN-GTNFVGGD-------RELRETFNA--MAPK------

LQEQL----AEQRI--RF---RFNPPSAPHFGGTW--EREVKSVKSALRVIL-----REQ

SV-----PEAVLQTLLVEVEGILNSKPLGYI-----------SSDVADLDPVTPNLL---

---------------LMG----RRDASL----------------------P---------

---------------------------------QVLYDSNNLLGRRRWRHSQ--------

-----VLADNF----WTAFI--------RHHLP-SLQDRQRW---------------RKD

G--KEL-------------T-----MGQVV--------LIVD-PQLPR----ALWPVGTV

SETLPG----ADGKIRTVRV------------------

>Sinbad

------------NIL------------------------------------------EGH

V-GA--------TQVMA-TVREK------------FRVLRGGV----------AMRRV--

-----IKD-CVS-CKXKER------------------PPPSN---N-----WHHYLRAEX

WCLSILIXWC----FGPIM---VKHGX---QVPQKR----YGCVFTC----LRL------

--RAVH-L--------EVA----YSFT-TDSF-----IM-----ALMRFIIRRG------

--------------YPKEIYSDN-GSNLVGAE-------RELRKCLQN--WVQER-----

IHSDL----LRKGI--DR---HFSPPAASHWGGVW--ERMIRSVRRVLGALV-----KEQ

PL--T---DECLETFMIEAERIINNRPLVPV-----------TDDSSDLDAITPAKL---

---------------LLL-----RER----------------------------------

------------------------------------------------------------

---------------YRTYR--------------SIQVFQEM------------------

-------------------E-----ASELLS-----TSFLET-------LVGICFAFAAX

IQMDPTREEHKGGXLNDMFV------------------

>Saci6

------------------------------------------------------------

-----------------------------------------GV----------KFN----

------TD-DVK-V----------------------------------------------

-------WE-----EGP-------------------------------------------

-----------------------------------------------RFLKK--------

---------------PKECWP---AVNIQGPEPH----LLELKKAMSTNVMVEES-----

T----------VGI--DW---HFSPPAASHWGGVW--ERMIRSVRRVLGALV-----KEQ

PL--T---DECLETFMIEAERIINSRPLVPV-----------TDDSSDLDAITPAKL---

---------------LLL-----REN------VTE-------------------------

----------------------------------LTNVLSNDRYSKRWKQAN--------

----YLA-QVF----WRRWS--------KEYVS-LLQRRYKW---------------TQL

E--RNI-------------R-----EGDLV--------MICS-EFSEK----NKWPLGLV

QRVLPS----KNGLVRQVEL------------------

>Kobel

---------------------------------------------------HSTVG---H

L-GR--------NSILA-KVREK------------YWIYGASQ----------LAKRV--

-----ARS-CVT-CQRYHA------AP-CEQVMAE--LPADR------------VVAEVS

PFTFCGMDY-----FAPLT---VRRGR----SEVKR----YGVIFTC----FSS------

--RAVH-L--------EIA----HSLE-TDAC-----IN-----AIRRFMARRG------

--------------PVRSIRSDN-GTNLVGSE-------KELRHALEG--LDQSR-----

MNDVL----CAEGI--EW---HFNPPAASHFGGVW--ERMIRSIRKILYSLL-----REQ

PRIVD---DETLSTLFCEAENIVNNRPLTTT-----------SSDPNDLLPLTPNML---

---------------INP-----RARALSSPGV---------------------------

------------------------------------FEKSDMYARKRWRQSQ--------

-----YLIDVF----WCRWR--------KEYLV-TLQQRPKW---------------QRK

R--RNV-------------Q-----VGDIV--------LVVD-KSVPR----NSWLMGVV

ETTFKD----KKGDVRSCRV------------------

>Purbel

----------------------------------------------------HELG---H

H-GV--------AVTTA-KTRRK------------YWILQGYR----------LAKTV--

-----KHR-CVT-CRAAEC------RR-ETQIMAN--LPSCR------------LAPFTP

PFHYTSCDY-----FGPYL---VKVGR---NKKAKH----YGIIFTC----LNT------

--RAVH-L--------EMA----TDCS-TMEF-----LQ-----ALRRFIAVRG------

--------------QPAQFLSDN-GTQFVGAE-------RELREMVRG--WSERE-----

LKDFC----AEKRV--VW---KFVTPGAPHQNGCA--EAMVKSCKFALKRAI-----GEQ

VL--T---PLELYTCFLEVANLVNERPIGRV-----------GNDPDDGGYLCPNDL---

---------------LLG-----RSS----------------------------------

----------------SKVPHGP-------------FRETR-NPRHRVEFVQ--------

-----QIVNSF----WKSWN--------RDVFP-LLVPRRKW---------------NTE

R--RNV-------------R-----VGDIA--------MLAD-ANAVR----GKWTIARV

VQVYPG----VDGTVRNVKV------------------

>Suzu

---------------------------------------------------HGWS----H

D-GV--------AGTLL-KMRCK------------AWVIKGRR----------VAQRV--

-----VDG-CVL-CRKIRA------RR-CQQVMAD--LPPER-------------TRPAA

PFQFTTVDL-----FGPYL---VKDDV-KRRVTLKT----WGVVFSC----MAC------

--RAIH-L--------DLV----NSVS-SESF-----LM-----AYQRFTAIRG------

--------------HPSKLWSDP-GTNFIGAK-------PVLQDLYQF--LESQNKAA--

LAEYA----VSRGT--EW-RWQIHPADSPHRNGAA--EAAVRVAKRALQALD-----KNT

ML--S---YSEFQTVLFTAANLANERPIEAR----------VQSKEDCIRYVSPNSL---

---------------LLG-----RAS----------------------------------

--------------------------NSG----DFNSFDFESYSYKRLQVIQ--------

-----CEVNNF----WKSWS--------QLVGP-NLFIRNKW---------------HTL

H--RNV-------------S-----EGDIV--------WVCD-QNSLR----GQY---KV

VKANPD----ARGIVRDVDV------------------

>Gabel

---------------------------------------------------HSRG----H

E-GT--------AATLL-KVRKR------------AWVIKGRR----------IAQKM--

-----IEN-CVI-CKKARA------RR-CRQVMGD--LPQER-------------TRPAA

PFEFTAVDL-----FGPYL---VKDDV-KKRVRMKV----WAVVFCC----MAS------

--RAIH-T--------ELA----NTMS-TESF-----LM-----AYQRFTAIRG------

--------------HPKKIWSDP-GTNFVGAK-------PVLEELYQF--LDGLDRPA--

VEETS----AQNGT--SW-HWEIQPADSPHRNGSA--EAAVRIVEKAFQSLG-----RES

EL--S---YSELQTTLQLTANLSNERPIDAR----------VQSCEDTVQYITPNAL---

---------------LLG-----QAS----------------------------------

--------------------------SSG----DWKTFVFTSYPYKRLQEMQ--------

-----HQVNKF----WRSWS--------QLAGP-NLFVRSKW---------------HTT

Q--RNV-------------A-----DGDIV--------WLCD-QNALR----GNFKAWEG

YKCQPR----LQRHSTGCESQSCDKLLYP---------

>Moose

---------------------------------------------------HVKAM---H

G-GI--------SSTLN-AVRDE------------IWPINGKR----------AVRKV--

-----IRN-CFR-CCRANP------QP-IIQPEGQ--LPAER-------------VTVNE

VFSCTGLDY-----CGPLY---LRPT-HRKAAPNKC----YICVFVC----MST------

--KAVH-L--------ELV----GDLS-TNSF-----LM-----ALDRFVYRRG------

--------------KPKHIYSDN-GTNFIGAK-------NELHQIYKM--LFNDSADSK-

IAKHL----AKEEI--QW---HLIPPRAPNFGGLW--EAAVKVAKTHLIRQL-----GSS

RL--S---SEEMTTVLVKIEGCMNSRPLVPL-----------SEDPNDLTALTPAHF---

---------------HIT-----NNL----------------------------------

---------------KVILEPDL--------------KEVPMNRLGRYQLLH--------

-----GYTQNF----WIHWK--------QDYLK-NLTVLHRS---------------AKQ

S--KQL-------------S-----VGDIV--------ILKD-EQLPA----VQWPLARV

VEIHPG----ADGISRVATL------------------

>Max

---------------------------------------------------HQITL---H

G-GS--------QLIVR-LIRTK------------YWIPKIKN----------LVKAV--

-----VNP-CKI-CTIYKK------RL-QTQLMGD--FPTDR-------------VSFSR

AFTYTGIDY-----AGPFE---IKNYTGRACLITKG----YVCVFVC----FST------

--KAIH-L--------EPT----SDLT-TEKF-----LA-----AFARFVARRG------

--------------CPQRVHSDN-GKTFVGAA-------ALISRDFLQ--AIKES-----

VTDAY----SHQGL--VW---RFIPPGAPHMGGLW--EAGVKSFKTLFLKST-----SVR

KY--T---FEELATLLAKIEACLNSRPLSPM-----------SEDPSDLLALTPGHF---

---------------LIG-----GPL----------------------------------

---------------LSTAEPEI--------------KGEAKSIINRWQHLK--------

-----AQHQQF----SARWK--------EEYLK-ELHKRSKW---------------QFP

T--RNL-------------Q-----ADDMV--------VVKE-DNLPP----NEWRLGRI

VSAFPG----ADERIRVVEI------------------

>Tribel

---------------------------------------------------HKQQM---H

C-GA--------QALLN-TLRQR------------YWPLRGRC----------LARQT--

-----VHK-CVR-CFRAKP------PD-SNYLMGS--LPACR-------------VTPAP

PFFRTGVDY-----AGPIM---IRNKRGRGSSLVKA----YICVFVC----LTT------

--RAIH-L--------EAV----SDLT-TQCF-----LQ-----TLRRFVARRG------

--------------KPRVIYSDN-GSNFVGAS-------SEL-QRLYN--FIRSKSNFDV

IQTSL----ANDSI--SW---IFIPANSPNWGGLW--EAGVKSVKFHLKRVL-----GNA

NL--V---FEDLCSVLCQIESILNSCPLSPL-----------SNDPNDMTPLTPGHF---

---------------LIG-----RPL----------------------------------

---------------TTIPSDNH--------------LDTPMKRLNRFEYQE--------

-----KICQDF----WQRWH--------QEYLS-YLQQRKKW---------------TQS

T--RQI-------------R-----PDDLV--------VIRD-QNLPP----MRWKMGRV

EEVYPSP---NDGVVRVASV------------------

>Nabel

---------------------------------------------------HTAHF---H

T-GI--------QNTLY-AIREN------------YWPIDGRN----------QIRKN--

-----IRK-CTI-CFRANP------QL-CQYKMRD--LPQVR-------------VTQSR

PFYNVGVDY-----CGPFF---IKEKRYRNQKFTKI----YVAIFVC----MTV------

--KAIH-I--------EVV----EDLS-TEGF-----IA-----ALRRFVSRRG------

--------------LPGTIYSDN-GTNFRGAH-------NKLNELYEL--LNSQQLKIN-

LEKFT----NSNKI--EW---HFIPPHSPNFGGLW--EISVKQFKHHFKRVA-----ADK

RF--T---LAEFNTFSIEIEAILNSRPITRI-----------SSDINDLSAITPGHF---

---------------LIG-----DSL----------------------------------

---------------KSLPEQNY--------------TKIPDNRLSAWEIMS--------

-----KLKQQF----WERWN--------KEYLN-ELNIRHNK---------------SAA

E--PKL-------------T-----KDLVV--------LIKE-DNTPP----MQWNMGII

TDVHPG----ADKIIRVVTV------------------

>Roo

---------------------------------------------------HKETL---H

G-GI--------NLMRN-YIQRK------------YWIFGLKN----------SLKKY--

-----LRE-CVT-CARYKQ------NT-AQQIMGN--LPKYR-------------VTMTF

PFLNTGIDY-----AGPYY---VKCSKNRGQKTFKG----YVAVFVC----MAT------

--KAIH-L--------EMV----SDLT-SDAF-----LA-----ALRRFIARRG------

--------------KCSNIYSDN-GTNFVGAA-------RKLDQELFN--AIQE--NIT-

IAAQL----EKDRI--DW---HFIPPAGPHFGGIW--EAGVKSMKYHLKRII-----GDT

IF--T---YEEMSTLLCQIEACLNSRPLYTI-----------VSEKDQQEVLTPGHF---

---------------LIG-----RPP----------------------------------

---------------LEIVEPME---------------DEKIGNLDRWRLIQ--------

-----KIKKDF----WVKWK--------SEYLH-TLQQRNKW---------------KKE

I--PNI-------------E-----EGQIV--------LLKD-ENCHP----ARWPLGKV

EKVHKG----NDDKVRVAKV------------------

>Bel

---------------------------------------------------HVSYL---H

T-GV--------DATFT-NLRQQ------------YWILGARN----------LVRKA--

-----VFQ-CKS-CFLQRK------GT-SNQIMGE--LPIPR-------------VQASR

CFQHTGLDY-----AGPIA---IKESKGRTPRIGKA----WFSIFVC----LTT------

--KALH-I--------EVV----SELT-TQAF-----IA-----AFQRFIARRA------

--------------KPTDLYSDN-GTTFHGGK-------KTLDDMRRL--AIQQAKDEE-

LAGFF----ANEGI--SW---HFIPPSAPHFGGMW--EAGVRSIKLHMKRIL-----GSK

AL--T---FEELSTVLTQIEAILNSRPL--C-----------PTGDNSLDPLTPAHF---

---------------LTG-----SPY----------------------------------

---------------TALPEPCR--------------LDMQVNRLERWNQLQ--------

-----AMVQGF----WKRWH--------MEYLT-SLHERTKW---------------HLE

T--ENL-------------K-----IDTLV--------VLKE-PNLPP----SKWILGRI

TAVHAG----IDNKVRVVTV------------------
