# Supplementary material for: Genome-wide characterization of LTR retrotransposons in the non-model deep-sea annelid Lamellibrachia luymesi
Source: BMC Genomics. 2021 Jun 23;22:466. doi: 10.1186/s12864-021-07749-1 (PMC8220671; doi:10.1186/s12864-021-07749-1)
Supplement: Supplementary file 3 — Additional file 3. Ribonuclease H sequence alignment file. [file 12864_2021_7749_MOESM3_ESM.docx]

>JULE

---------------------------------------LACDA-----SP---------

-----------YGVGA---------------------VI--SHIL-----E-NGEE----

--------RPVAFASRTLTDAER-------K---Y-AQIEKEALAIIFGV-------KKF

HKYLY-----------GR-K-----FT----------LVTDHKP-LLTILGPKSAVP--T

LAALR-MQRWALI-LM-A---------YNYDIEYR---R-SAD----------HA-----

NVDALSRLP----------------------------------

>PYG1

--------------------------------------VLSCDA-----SP---------

-----------YGLGA---------------------VL--SHVM-----L-DGRE----

--------RPIAYVSRTLAPAEK-------N---Y-SQLDKEGAALILGV-------RKF

HQYLF-----------GQ-K-----FV----------VYTDHKP-LLGLFKADRAIP--S

MASAR-IQRWGLL-LA-T---------YEYDLRYR---P-GTK----------NS-----

NADGLSRLA----------------------------------

>JRE

--------------------------------------VLSCDA-----SA---------

-----------YGVGA---------------------VL--AHRM-----A-DGTE----

--------RPIGFVSRTLTVAEK-------N---Y-SQLEKEGLAVVFGV-------KKF

HKYLY-----------GR-K-----FV----------ICTDHKP-LLTLLNELKAVP--Q

MVSQR-IMRWALM-LG-A---------YEYVISYR---A-GKD----------NG-----

NADALSRLP----------------------------------

> steamer

WT-------HEHDEAFKNIKTAVCNVPVLRYFDSRLNTVLQCDA-----SE---------

-----------TGLGA---------------------TL--MQ-----------EG----

--------QPVAYASRALTSTEQ-------N---Y-AQIEKELLAVVFGF-------EKF

HQFTY-----------GR-R-----VV----------V----------------------

------------------------------------------------------------

-------------------------------------------

>LLGY1

------------------------------------------------------------

-----------AGLGS---------------------IL--IQD-----------S----

--------RAIAFASRPLSDVES-------R---Y-SQTEPEALGVVWGC-------EHF

DQYLQ-----------GDPQ-----FT----------IITDHEL-FLITWKKAR------

-PPLR-IERWGLR-LQ--------------------------------------L-----

RSEIHTR--KQ----------------------------KYNR

>LLGY2

---------------------------------------VSSDA-----SK---------

-----------DGIGA---------------------VL--LQ-------ETNGEW----

--------MPVAYASRSMTAAEK-------N---Y-AQIEKEQLGVVFAC-------ERF

HVYIY-----------GR-K-----VI----------VETDHQP-LIAISKKQLCD----

-APPR-LQRLLLR-IQ-K---------YDLMLEYT---P-GKL----------LV-----

VADTLSR------------------------------------

>LLGY5

--------------------------------------CLTTDW-----SK---------

-----------EGIGF---------------------FL--LQKHC----ACPGEV----

PFCCNDGWKVTLVGSRFTHPAES-------R---Y-APIEGEALAVADAL-------ERT

RYFVL-----------GCDD-----LI----------VAVDHQP-LLKVLGDRKL-----

--EDI-KNPRLLN-LK-EKTL-----PFKFKLIHI---P-GKR----------HL-----

ATDAISR------------------------------------

>LLGY6

------------------------------------------------------------

------------------------------------------------------------

---------ITLVGSRFTHAAES-------R---Y-APIEGEALAVADAL-------DKA

RHFVL-----------GCEN-----LI----------VAVDHKP-LLKLLADRAL-----

--DDI-PNPRLRN-LK-EKTL-----RYRFRITHI---P-GMK----------NK-----

TADAMSR------------------------------------

>LLGY7

--------------------------------------ALHTDA-----SR---------

----------RKGLGY---------------------AL--LQKH-------DEKW----

--------RLVQCGSRFLTDTES-------R---Y-AMVELELLAATWAM-------KKC

RIQLL-----------GMEH-----FE----------LVVDHKP-LVTILDRHRLD---D

VDNVR-LQRLKEK-TS-L---------FTFTTRWT---K-GKD----------HC-----

IPDALSR------------------------------------

>LLGY10

--------------------------------------EIVVDA-----SP---------

-----------VGLAA---------------------LL--VQ-----------EK----

--------RVVVYGSRALSDVET-------R---Y-SQTEREALAVVWAC-------EHF

DKFIN-----------GAPQ-----FT----------VISDHKP-LETIWKKPR------

-PPLR-IERWGLC-LQ-P---------YRM------------------------------

EGNYLHRFIPG----------------------------RW--

>LLGY11

--------------------------------------KLYTDV-----CD---------

-----------YAIGG---------------------ML--VQESV------DGIE----

--------KVIQYVSHTLSPTQR-------K---W-ATIEKEAFAVVFSI-------LKL

RPYLY-----------GA-Q-----FN----------VYTDHKP-LLSLFTKAFN-----

--NTK-IQRWGVL-LA-E---------YGTTISYR---T-GRN----------NI-----

SADMLSR------------------------------------

>LLGY12

----------------------------------------QADA-----SQ---------

-----------RGLGA---------------------CL--L------------QG----

--------RPVAYASRALTSAEE-------N---Y-SQIEKEMLAVFFV-----------

------------------------------------------------------------

------------------------------------------------------------

-------------------------------------------

>LLGY13

------------------------------------------DV-----RR---------

-----------KTCGV---------------------CL---------------------

----------------TITKCEQ-------K---Y-APIELECLAIVFAC-------RKF

DQYIY-----------GHAK-----VT----------IHSDHKP-LEAIFRKSLLE----

-APKR-LQRMMIA-VQ-R---------YNVKVEYK---P-GTE----------QL-----

VADMLSR------------------------------------

>LLGY14

--------------------------------------CLTTDW-----SK---------

-----------EGIGF---------------------FL--LQKHC----ACPGEV----

PFCCNDGWKVTLVGSRFTHPAES-------R---Y-APIEGEALAVADAL-------ERT

RYFVL-----------GCDD-----LI----------VAVDHQP-LLKVLGDRKL-----

--EDI-KNPRLLN-LK-EKTL-----PFKFKLIHI---P-GKR----------HL-----

ATDAISR------------------------------------

>LLGY15

--------------------------------------VLTCDA-----SP---------

-----------YGVGA---------------------VL--AHAF-----D-DRVE----

--------RPIAYYSRSLSAAEK-------N---Y-AQIDKEGLAVIAGL-------TKF

HQYLW-----------GR-P-----FL----------IVTDHKP-LLGLFGEQKAVP--Q

MLSPR-MQRWALT-LA-A---------YEYQIVHR---P-GSS----------IP-----

QADALSR------------------------------------

>LLGY16

-----------------------------------------YDA-----SN---------

-----------YGLRV---------------------VL--SHVM-----E-DGTE----

--------RPVGFASRTLNAVEK-------N---Y-SQLDKEGAAVMFAL-------KKF

HKHLY-----------GR-S-----FE----------IITDHK----SLFGEMKQVP--T

TASAR-IQRWAVT-LC-G---------YEYNIHYK---A-GKS----------HN-----

NADCLSR------------------------------------

>LLGY17

--------------------------------------VLATDA-----SP---------

-----------YGIGA---------------------VI--SHVL-----P-DGSE----

--------EPIAFASKTLSKAER-------G---Y-AQVEKEGLSIVYGI-------RKF

NQYLS-----------GR-H-----FT----------ILTDHKP-LLTIFGPDKSLP--A

MSLQR-LQRWALL-LM-G---------HDYDIRYR---A-SAE----------HC-----

NADALSR------------------------------------

>LLGY18

--------------------------------------VVECDA-----SE---------

-----------RGLGA---------------------AL--LQ-----------NG----

--------KPIGYASRTFTSTET-------R---Y-AQIEKECLAIVFTL-------ERF

HQYTF-----------GR-R-----TI----------VHTDHKP-LEMIVKKPLYK----

-APRR-LQGMLLR-ML-Q---------YDTEVVYH---K-GKE----------MY-----

IADTLSR------------------------------------

>LLGY19

--------------------------------------VLQCDA-----ST---------

-----------QGLGA---------------------SL--ME-----------DG----

--------KPVAYVSRSLTKCEQ-------K---Y-APIELECLAIVFAC-------RKF

DQYIY-----------GHAE-----VT----------IHSDHKP-LEAIFRKSLLE----

-APKR-LQRMMLA-VQ-R---------YNVKVEYK---P-GTE----------QL-----

VADMLSR------------------------------------

>LLGY20

---------------------------------------IFVDA-----SP---------

-----------IGLGA---------------------VL--TQENA-----ISKEV----

--------TPLYFASRPLTPTES-------R---Y-PQIDREALAISWAI-------KRF

HLYAY-----------GKE------FK----------VVTDHKP-LVTLFNNPSS-----

KPTAR-IERWLMD-LL-R---------YNFTVTYQ---P-GKS-----------N-----

PADYASR------------------------------------

>LLGY21

---------------------------------------IFVDA-----SP---------

-----------IGLGA---------------------VL--TQENA-----ISKEV----

--------TPLYFASRPLTPTES-------R---Y-PQIDREALAISWAI-------KRF

HLYAY-----------GKE------FK----------VVTDHKP-LVTLFNNPSS-----

KPTAR-IERWLMD-LL-R---------YNFTVTYQ---P-GKS-----------N-----

PADYASR------------------------------------

>LLGY22

--------------------------------------VLTCDA-----SP---------

-----------YGIGS---------------------VL--AHVM-----E-DGAE----

--------KPIAYHSRSLSHAEK-------N---Y-AQIDKEGLSVIVGL-------KKF

HQYLW-----------GH-K-----FT----------IVTDHKP-LLGLFGETKAVP--Q

MLSPR-MQRWALT-LA-A---------YEYQIIHR---P-GQS----------IP-----

QADALSR------------------------------------

>LLBP1

--------------------------------------HNFSDA-----S----------

----------LTGYGQ-------------C-------SY--LRL-----MDSHDNISCAL

----------VMGKSRVGPSRFV-------------TIPRLELTAAVLSVKVG----NFL

QKELD------------YE--AIT-HY----------YWTDSKV-VLGYINN-ESR----

RFN-IFVANRVQQ-IR-SSTK-------VSAWKYV---G-TRK----------NP-----

-ADIASR------------------------------------

>LLGY23

---------------------------------------LRPDW-----SK---------

-----------LGIGY---------------------FL--SQKHC----DCATTL----

PGCCDDGWKITLAGSRFLTPTEQ-------R---Y-APIEGEALAVAWGL-------EQS

RYFTQ-----------GCDD-----LV----------VVTDHKP-LVKILGDRTL-----

--DEI-NNSRIFR-LK-QRTL-----PWHFEVTHL---P-GKT----------NT-----

AADATSR------------------------------------

>LLGY24

----------------------------------------FVDA-----SP---------

-----------LGLGA---------------------IL--TQN-----------G----

--------KVVSYASRALSDVER-------R---Y-SQTEREMLAVVWSA-------EHF

HLYLY-----------GAS------FT----------IHTDHQP-LLGIFNKSQR-----

PASAR-IERWRLR-LL-P---------YQFELKYR---P-GKDD---------RN-----

PADYISR------------------------------------

>LLGY25

--------------------------------------CLTTDW-----SK---------

-----------EGIGF---------------------FL--LQKHC----ACPGEV----

PFCCNDGWKVTLVGSRFTHPAES-------R---Y-APIEGEALAVADAL-------ERT

RYFVL-----------GCDD-----LI----------VAVDHQP-LLKVLGDRKL-----

--EDI-KNPRLLN-LK-EKTL-----PFKFKLIHI---P-GKR----------HL-----

ATDAISR------------------------------------

>LLGY26

---------------------------------------VSSDA-----SK---------

-----------DGIGA---------------------VL--LQ-------ETNGEW----

--------MPVAYASRSMTAAEK-------N---Y-AQIEKEQLGVVFAC-------ERF

HVYIY-----------GR-K-----VI----------VETDHQP-LIAISKKQLCD----

-APPR-LQRLLLR-IQ-K---------YDLMLEYT---P-GKL----------LV-----

VADTLSR------------------------------------

>LLGY27

---------------------------------------LQCYA-----SE---------

-----------GGLGD---------------------CI--LK-----------ND----

--------QPIAYASRALTDAEG-------R---Y-AQIEKEMLAIIFGL-------ERF

DQYVY-----------GR-P-----VL----------VESGHKP-LEMIHKKALQA----

-APKH-LQRMLLR-AQ-R---------YDINIVYK---K-G-H----------MY-----

TADTLSR------------------------------------

>LLGY28

--------------------------------------TIQCDA-----SE---------

-----------TGLGA---------------------VL--LQ-----------EG----

--------QPICYASRALTDTET-------R---Y-AQIEKELLAIVWSC-------DKF

DQYIY-----------GRDM-----VT----------IESDHEP-LKAVFKKDIHK----

-SPKR-LQRMCLA-LQ-K---------YNLDVQYK---K-GSL----------MY-----

ISDTLSR------------------------------------

>LLGY29

--------------------------------------TIQCDA-----SQ---------

-----------SGLGA---------------------AL--MQ-----------NG----

--------QPVAYASRALTPPET-------R---Y-AQIEKELLAIVFAC-------DRF

EAYIY-----------GRDR-----VS----------IESDHKP-LETIVLKPLSS----

-APKR-LQLMLLR-FQ-K---------YTLDVKFK---K-GEH----------MY-----

LADTLSR------------------------------------

>LLGY30

---------------------------------------LIVDA-----SP---------

-----------VGLGA---------------------IL--AQKES----EEEEGG----

--------RIVAYASRALSEVEQ-------R---Y-SQTEREALAVAWGT-------EKF

HLYIY-----------GKS------VE----------IITDHKP-LEGLFNNPRS-----

KPNAR-IERWLMR-MQDK---------YDYKVTYR---P-GKNP---------EN-----

PADYMAR------------------------------------

>LLGY31

--------------------------------------VVQVDA-----SS---------

-----------RGLGA---------------------VL--MQ-----------GG----

--------RPIAFASKSLTDCER-------R---Y-ANIEREMLAVVFGC-------ERF

HTYVY-----------GK-H-----FT----------VESDHKP-LEMIHLKNLAA----

-APQR-LQRMLLR-VQ-P---------YDFKLHYI---P-GKN----------IA-----

LADTMSR------------------------------------

>LLGY32

------------------------------------------------------------

------------------------------------------------------------

--CCPDGWRITLVGSRFTHPAES-------R---Y-APIEGEALSVADAL-------DKA

RFFVL-----------GCAD-----LI----------VAVDHKP-LLKILGDRSL-----

--DEI-ANARLRN-LK-EKTL-----RYRFRIVHI---P-GVQ----------NK-----

AADAMSR------------------------------------

>LLGY34

----------------------------------------SSDA-----SK---------

-----------DGISA---------------------VL--LQ--------TNGER----

--------MHLAYVSRSLTAAEK-------N---Y-AHIEKEHIGVVFAC-------ERF

HVYVY-----------GR-S----------------------------------------

------------------------------------------------------------

-------------------------------------------

>LLGY35

--------------------------------------VIQVDA-----SG---------

-----------KGLGA---------------------VL--LQ-----------DK----

--------KPIAYASKSLTDAEK-------R---Y-ANIERELLAVVF------------

------------------------------------------------------------

------------------------------------------------------------

-------------------------------------------

>LLGY36

--------------------------------------LLQCDA-----SN---------

-----------YGLGV---------------------VL--SHVM-----E-DGTE----

--------RPVGFASRTLKAAEK-------N---Y-SQLDKEGAAVMFAL-------KKF

HKHLY-----------GR-S-----FE----------IITDHKP-LVPLFGELKQVP--T

TASPR-IQRWAVT-LC-G---------YEYNIHYK---A-GKS----------HN-----

NADCPSR------------------------------------

>LLGY37

--------------------------------------TLSADA-----SS---------

-----------HGLGA---------------------VL--LQRQ-----P-DGKL----

--------QPVAYASRSMSSTEQ-------R---Y-AQIEKEALAKTWAC-------ERF

SDFLL-----------GK-T-----FH----------VDTDHKP-LVSLLG-QKTL---D

QLPPR-IQRFRMR-LM-R---------FNYSIAHV---A-GKD----------LI-----

TADTLSR------------------------------------

>LLGY38

--------------------------------------RLYTDA-----CQ---------

-----------YAIGG---------------------IL--VQES-----D-DGVE----

--------KVIQYVSHTLSNTQR-------K---W-ATIEKEAYAVVFCI-------EKL

RAYLF-----------GS-H-----FH----------VYTDHKP-LLSLFTKALN-----

--NTK-IQRWGIL-LA-E---------FGATISYR---S-GRH----------NI-----

RADMLSR------------------------------------

>LLGY39

--------------------------------------TLQCDA-----SQ---------

-----------SGLGA---------------------AL--TQ-----------LG----

--------QPVAFTSRALTSAGT-------R---Y-AHIEKELLSIVYAC-------EKF

DAYVY-----------GREE-----VT----------IQTDHKP-LECIFKKPLNT----

-APMR-LRHMLLR-LQ-R---------YNLKVLYH---K-GTE-----------Y-----

LADTLSR------------------------------------

>LLGY40

--------------------------------------TLSADA-----SS---------

-----------HGLGA---------------------VL--LQRQ-----P-DGKL----

--------QPVAYASRSMSSTEQ-------R---Y-AQIEKEALATTWAC-------ERF

SDFLL-----------GK-T-----FH----------VETDHKP-LVSLLG-QKTL---D

QLPPR-IQRFRMR-LM-R---------FNYSIAHV---A-GKD----------LI-----

TADTLSR------------------------------------

>LLGY41

--------------------------------------LLQCDA-----SN---------

-----------YWLGV---------------------VL--SHVM-----E-DGTE----

--------RPVGFGSRTMNAAEK-------N---Y-SKLDKEGAAVMFAL-------KKF

HKNLY-----------GR-S-----F-----------IITDHKP-FVSLFGELKQVP--T

TASPR-IQRWAVT-LC-G---------YEYNIHYK---A-GKS----------HN-----

NADCLSR------------------------------------

>LLGY42

---------------------------------------LIVDA-----SP---------

-----------VGLGA---------------------VL--VQHD------KDGHS----

--------QVIAYASRAVADVES-------P---F-S-----------------------

------------------------------------------------------------

------------------------------------------------------------

-------------------------------------------

>LLGY43

--------------------------------------TLEVDA-----SM---------

-----------KGLGA---------------------AL--VQ-----------EG----

--------KPVAFASKTLIKTQA-------N---Y-SNIEREMLALVHGV-------ESF

HAYLY-----------GR-S-----FT----------I-------------KPIAS----

-APPR-LQRMLVK-IQ-G---------YDYSVKYR---P-GKE----------MV-----

VSDALSR------------------------------------

>LLGY44

-----------------------------------------ADA-----SQ---------

-----------RGLGA---------------------CL--LQ-----------QG----

--------RPVAYASRALSSAEE-------N---Y-SQIEKEMLAICFAC-------AKF

HQCVY-----------GK-S-----IE----------VHTDRRP-LESILKKPIAK----

-ASPR-LQRMMLQ-LQ-R---------YTLIVKYI---P-GKY----------MY-----

VADTLSR------------------------------------

>LLGY45

--------------------------------------VVQVDA-----SS---------

-----------RGLGA---------------------VL--MQ-----------GG----

--------RPIAFASKSLTDCER-------R---Y-ANIEREMLAVVFGC-------ERF

HTYVY-----------GK-H-----FT----------VESDHKP-LEMIHLKNLAA----

-APQR-LQRMLLR-VQ-P---------YDFKLHYI---P-GKN----------IA-----

LADTMSR------------------------------------

>LLGY48

--------------------------------------IVETDA-----SF---------

-----------QGLGA---------------------VL--SQEH---------------

--------GVIAYASRGLRPAER-------NDANY-SSMKLELLALKWAIT------DKF

RPYLL-----------GS-K-----FT----------VYTDNNP-L-SYIQTSKL-----

--GAT-ELRWAAQ-LA-Q---------FDFSIKYR---S-GRT----------NA-----

SADALSR------------------------------------

>LLGY49

--------------------------------------TIQCDA-----SQ---------

-----------SGLGA---------------------AL--MQ-----------NG----

--------QPVAYASRALTPPET-------R---Y-AQIEKELLAIVFAC-------DRF

EAYIY-----------GRDR-----VS----------IESDHKP-LETIVLKPLSS----

-APKR-LQRMLLR-LQ-K---------YTLDVKFK---K-GEH----------MY-----

LADTLSR------------------------------------

>LLGY50

----------------------------------------YVDA-----SP---------

-----------VGLGA---------------------IL--SQHDG-----NGENQ----

--------CIIAHGSRSLTAVEQ-------R---Y-SQTEREAIAVVWAC-------EHF

HLYVY-----------GKS------VK----------VYTDHKP-LVAIYGSPKS-----

KPPAR-IERWTIR-LQ-P---------YDATVIYR---P-GSD-----------N-----

PADLLSR------------------------------------

>LLGY51

--------------------------------------TLSADA-----SS---------

-----------HGLGA---------------------VL--LQRQ-----P-DGKL----

--------QPVAYASRSMSSTEQ-------R---Y-AQIEKEALATTWAC-------ERF

SDFLL-----------GK-T-----FH----------VETDHKP-LVSLLG-QKTL---D

QLPPR-IQRFRMR-LM-R---------FNYSIAHV---A-GKD----------LI-----

TADTLSR------------------------------------

>LLGY52

--------------------------------------ALTVDA-----SG---------

-----------AAIGA---------------------VL--EQNL--------GSW----

--------KPVAFFSRKLRAAEQ-------K---Y-SAFDRELLAAYLAI-------RHF

RYFLE-----------GR-S-----FV----------LFTDHKP-LTFAISKTSD-----

PWSSR-QQRHLTY-IS-E---------FTTDVRHI---G-GKN----------NT-----

VADTLSR------------------------------------

>LLGY53

--------------------------------------ILATDA-----SS---------

-----------YGVGA---------------------VI--SHQM-----S-DGVE----

--------KPIAFASRTLSTAER-------N---Y-SQIEREALGIIFGV-------KKF

HQYLM-----------GR-S-----FT----------MRTDHRP-LTKIFGPKTGIP--S

MAAAR-MQRWALV-LA-G---------YQYDIQYI---P-SKE----------NA-----

NADMLSR------------------------------------

>LLGY54

--------------------------------------KLYTDA-----CD---------

-----------YAIGG---------------------IL--VQESV------DGIE----

--------KVIQYVSHTLSPTQR-------K---W-ATIEKEAFAVVFSI-------LKL

RPYLY-----------GA-Q-----FN----------VYTDHKP-LLSLFTKAFN-----

--NTK-IQRWGVL-LA-E---------YGTTISYR---T-GRN----------NI-----

RADMLSR------------------------------------

>LLGY55

--------------------------------------TLSADA-----SS---------

-----------HGLGA---------------------VL--LQRQ-----P-DGKL----

--------QPVAYASRSMSSTEQ-------R---Y-AQIEKEALATTWAC-------ERF

SDFLL-----------GK-T-----FH----------VETDHKP-LVSLLG-QKTL---D

QLPPR-IQRFRMR-LM-R---------FNYSIAHV---A-GKD----------LI-----

TADTLSR------------------------------------

>LLGY56

------------------------------------------------------------

------------------------------------------------------------

---------------------------------------RKEALTMIYGV-------RKF

HKYLW-----------GR-H-----FK----------IYTDHKS-LLGLLGEMKPLP--Q

NSSAR-LQRWALL-MQ-G---------YDYELIYR---P-GTN----------LA-----

NADALSR------------------------------------

>LLGY57

------------------------------------------------------------

------------------------------------------------------------

-------------------LAER-------N---Y-SQIDKEALGLVWGV-------RKF

NQYLF-----------GR-R-----LT----------LVTDHQP-LTAIFHPWKSIP--A

MTAAR-MQRYALQ-LA-A---------HDYDIVYK---S-SLK----------HA-----

NADGLLR------------------------------------

>LLGY58

--------------------------------------CLTTDW-----SK---------

-----------EGIGF---------------------FL--LQKHC----ACPGEV----

PFCCNDGWKVTLVGSRFTHPAES-------R---Y-APIEGEALAVADAL-------ERT

RYFVL-----------GCDD-----LI----------VAVDHQP-LLKVLGDRKL-----

--EDI-KNPRLLN-LK-EKTL-----PFKFKLIHI---P-GKR----------HL-----

ATDAISR------------------------------------

>LLGY60

--------------------------------------ALTVDA-----SG---------

-----------TAIGA---------------------VL--EQDL--------GSW----

--------KPVAFFSRKLRPAEQ-------K---Y-SAFDRELLAMYLAI-------RHF

RYFLE-----------GR-T-----FT----------LYTDHKP-LTFAISKVSD-----

PWSPR-QQRHLAY-IS-E---------FTTDVRHI---E-GKN----------NT-----

VADTLSR------------------------------------

>LLGY61

--------------------------------------VLQVDS-----SK---------

-----------DGLGA---------------------AL--MQ-----------NG----

--------KPIEYASRNLRSNER-------N---W-AQIEKETLALVYGL-------EKF

DQYTY-----------GR-K-----VV----------VHNDHKP-LAAILSKPLSH----

-APRR-LQSLMMR-LY-R---------YDIEFHYV---K-GVQ----------LY-----

LADTLSR------------------------------------

>LLGY64

-------------------------------------------T-----SP---------

-----------YSVGA---------------------VL--SHVM-----E-DGSE----

--------RPIGFVSRTLAPAEN-------K---Y-SQLDKEGLAIIFGI-------KKF

HQHLY-----------GR-S-----FK----------ITSDHKP-LLGLLGENKGV----

------------------------------------------------------------

-------------------------------------------

>LLGY65

--------------------------------------IVETDA-----SH---------

-----------LGLGA---------------------VL--SQEQ-------EGRR----

--------VVIAYASRRLRPPER-------Q---Y-SSMKLEMLALKWAVT------TKF

RADLY-----------GG-E-----FV----------IYTDNNP-L-KYLKTAKL-----

--GAI-EQRWAAE-LA-P---------FNFTIEYR---A-GRS----------NG-----

NADALSR------------------------------------

>LLGY66

--------------------------------------MIQADS-----SF---------

-----------KGLGA---------------------CL--LQ-----------NS----

--------QPVAYASRALTDTES-------R---Y-AQIEEELLCIVFTA-------ERF

HQYIY-----------GR-D-----ME----------VQSDHKP-LEMITRKPLHN----

-ASPR-LQVMLLR-LL-----------WKLSIRYV---K-GSK----------MY-----

IAGTLSR------------------------------------

>LLGY67

--------------------------------------ALHTDA-----SR---------

----------RKGLGY---------------------AL--LQQH-------ADKW----

---------LIQCGSRFLTDTES-------R---Y-AMVELELLAATWAM-------KKC

RIQLL-----------GLPH-----FE----------LVVDHKP-LVTILDRHRLD---D

IDNTR-LQRLKEK-TS-L---------FSFTTRWT---K-GKD----------HC-----

IPDALSR------------------------------------

>LLGY68

------------------------------------------------------------

------------------------------------------------------------

------------------------------------------------------------

-----------------K-D-----PN-------------------------KIFA----

-ATTR-LQRMLIK-LH-G---------YNFTMTHR---P-GSQ----------NQ-----

LADGLSR------------------------------------

>LLGY69

-------------------------KP----------FILETGA-----SH---------

-----------QGLGA---------------------LL--LQEQ-------DGKT----

--------RVIAHASRGLRGPEK-------NRTAY-SSMKLELLTVKWAVT------EKF

LDYLL-----------GV-Q-----FV----------IYTDNNP-L-LYIQTSSKL----

--TAA-EHHWQAE-LA-R---------FNFSIHYR---P-GRL----------NA-----

SADGLSR------------------------------------

>LLGY70

--------------------------------------ILATDA-----SS---------

-----------YGVGA---------------------VI--SHQM-----S-DGVE----

--------KPIAFASRTLSTAER-------N---Y-SQIEREALGIIFGV-------KKF

HQYLM-----------GR-S-----FT----------MRTDHRP-LTKIFGPKTGIP--S

MAAAR-MQRWALV-LA-G---------YQYDIQYI---P-SKE----------NA-----

NADMLSR------------------------------------

>LLGY71

--------------------------------------TLEVDA-----SM---------

-----------KGLGA---------------------AL--VQ-----------EG----

--------KPVAFASKTLIKTQA-------N---Y-SNIEREMLALVHGV-------ESF

HAYLY-----------GR-S-----FT----------I-------------KPIAS----

-APPR-LQRMLVK-IQ-G---------YDYSVKYR---P-GKE----------MV-----

VSDALSR------------------------------------

>LLGY72

---------------------------------------LQCDA-----SL---------

-----------RGIGA---------------------AL--LQ----P--DADGEL----

--------RPVEYASKSLTPTEQ-------R---Y-ACIERELLSIVFGM-------QRF

HTYLY-----------GR-D-----FN----------VITDHRP-LLMITNKPIAS----

-APPR-LQRMLIK-LH-G---------YNFTMTHR---P-GSQ----------NQ-----

LADGLSR------------------------------------

>LLGY73

------------------------------------------------------------

------------------------------------------------------------

------------------------------N---Y-AQIE-ELLAIVFAC-------EKF

DQYVY-----------GREK-----VH----------VQSDHKP-LEVVFRK--------

------------------------------------------------------------

-------------------------------------------

>LLGY74

---------------------------------------LRPDW-----SK---------

-----------LGIGY---------------------FL--SQKHC----DCATTL----

PGCCDDGWKITLAGSRFLTPTEQ-------R---Y-APIEGEALAVAWGL-------EQS

RYFTQ-----------GCDD-----LV----------VVTDHKP-LVKILGDRTL-----

--DEI-NNSRIFR-LK-QRTL-----PWHFEVTHL---P-GKT----------NT-----

AADATSR------------------------------------

>LLGY75

--------------------------------------TIQVDA-----SG---------

-----------RGLGA---------------------VL--LQ-----------EG----

--------RPIAYASKSLTDTEK-------R---Y-ANIERELLAVVFGA-------ERF

RTYVY-----------GK-H-----FV----------VESDHKP-LEMIQLKNLMA----

-APPR-LQRMLLR-IQ-H---------YDITIKYR---P-GKE----------LL-----

LADGLSR------------------------------------

>LLGY76

--------------------------------------VVQVDA-----SS---------

-----------RGLGA---------------------VL--LQ-----------KN----

--------KPIAFASKSLSDCER-------R---Y-VNIEREMLAVVFGC-------ERF

HTFVY-----------GK-R-----FT----------VESDHKP-LEMIHMKNLAA----

-APQR-LQRMLLR-IQ-P---------YDIVIKYR---P-GKD----------VA-----

VADLLSR------------------------------------

>LLGY77

----------------------------------------QADA-----SQ---------

-----------RGLGG---------------------CL--LQ-----------QG----

--------RPVAYASRALTSAEE-------N---Y-SQIEKEMLAICFAC-------AKV

HQYVY-----------GK-S-----IE----------VHTDHRP-LESILKKPIAK----

-ASPR-LQRVMLQ-LQ-R---------YTLEVKYI---P-GKY----------MY-----

VADT---------------------------------------

>LLGY78

--------------------------------------VLETDA-----WN---------

-----------DSIGA---------------------VL--SQVQ-------DGKS----

--------KVIAYISRRLRGGEK-------NMDNY-SSKKLELLALKWAVT------EKL

RDYLH-----------GA-H-----FT----------VYTDNNP-L-THVLTQKKL----

--PAL-EQRWVNT-LA-S---------FNFDIKYR---P-GKT----------NA-----

NADGLPR------------------------------------

>LLGY79

---------------------------------------INTDA-----SD---------

-----------TAVGA---------------------VL--QQRL-------NGVW----

--------TPISFFSRKLHAAEK-------K---Y-STFDKELLAMYLAV-------KKF

RYFIE-----------GR-K-----FT----------LFTDHKP-LTFVFNNVSD-----

KWSPR-QQRHLCL-VS-E---------FTTDIRYV---P-GAD----------NV-----

VADALSR------------------------------------

>LLGY80

--------------------------------------TIQCDA-----SQ---------

-----------SGLGA---------------------AL--MQ-----------NG----

--------QPVAYASRALTPPET-------R---Y-AQIEKELLAIVFAC-------DRF

EAYIY-----------GRDR-----VS----------IESDHKP-LETIVLKPLSS----

-APKR-LQRMLLR-LQ-K---------YTLDVKFK---K-GEH----------MY-----

LADTLSR------------------------------------

>LLGY81

--------------------------------------VLCTDA-----LD---------

-----------GGLGA---------------------VL--LQEH-------DGMN----

--------MPVMYVSRKLSEAETC----------Y-STFERECLGLFWAT-------KRL

HVYLY-----------CM-E-----FI----------LETDHQP-L-AFMNRANI-----

-SNDR-VMRWALH-MQ-M---------YRYRVRIV---K-GTD----------NT-----

TADFLSR------------------------------------

>LLGY82

--------------------------------------VLATDA-----SP---------

-----------YGIGA---------------------VI--SHVL-----P-DGSE----

--------EPIAFASKTLSKAER-------G---Y-AQVEKEGLSIVYGI-------RKF

NQYLS-----------GR-H-----FT----------ILTDHKP-LLTIFGPDKSLP--A

MSLQR-LQRWALL-LM-G---------HDYDIRYR---A-SAE----------HC-----

NADALSR------------------------------------

>LLGY83

--------------------------------------VVQVDA-----SS---------

-----------RGLGA---------------------VL--LQ-----------KN----

--------KPIAFASKSLSDCER-------R---Y-VNIEREMLAVVFGC-------ERF

HTFVY-----------GK-R-----FT----------VESDHKP-LEMIHMKNLAA----

-APQR-LQRMLLR-IQ-P---------YDIVIKYR---P-GKD----------VA-----

VADLLSR------------------------------------

>LLGY84

--------------------------------------VVQVDA-----SS---------

-----------RGLGA---------------------VL--LQ-----------KN----

--------KPIAFASKSLSDCER-------R---Y-VNIEREMLAVVFGC-------ERF

HTFVY-----------GK-R-----FT----------VESDHKP-LEMIHMKNLAA----

-APQR-LQRMLLR-IQ-P---------YDIVIKYR---P-GKD----------VA-----

VADLLSR------------------------------------

>LLGY85

--------------------------------------CLTTDW-----SK---------

-----------EGIGF---------------------FL--LQKHC----ACPGEV----

PFCCNDGWKVTLVGSRFTHPAES-------R---Y-APIEGEALAVADAL-------ERT

RYFVL-----------GCDD-----LI----------VAVDHQP-LLKVLGDRKL-----

--EDI-KNPRLLN-LK-EKTL-----PFKFKLIHI---P-GKR----------HL-----

ATDAISR------------------------------------

>LLGY86

--------------------------------------VLTCDA-----SP---------

-----------YGIGS---------------------VL--AHAM-----E-DGAE----

--------KPIAYHSRSLSHAEK-------N---Y-AQIDKEGLSVIVGL-------KKF

HQYLW-----------GH-K-----FT----------IVTDHKP-LLGLFGETKAVP--Q

MLSPR-MQRWALT-LA-A---------YEYQIIHR---P-GQS----------IP-----

QADALSR------------------------------------

>LLGY88

-------------------------KP----------VTIQTDA-----SK---------

-----------RGIGA---------------------TL--LQ-----------NG----

--------RPVAYASKSL---------------------------------------ERC

HHYAF-------------------------------------------------------

------------------------------------------------------------

-------------------------------------------

>LLGY89

----------------------------------------QADA-----SQ---------

-----------RGLGA---------------------CL--LQ-----------QG----

--------RPVAYASRALTSAEE-------N---Y-SQIEKEMLAICFAF-------VKF

HQYVY-----------GK-S-----IE----------VHTDHRP-LELILKTPIVK----

-ASPQ-LQQMMLQ-LQ-R---------YTLEVKYI---P-GKY----------MY-----

VADTLSR------------------------------------

>LLGY91

--------------------------------------TVQADA-----SQ---------

-----------RGLEA---------------------CL--LQ-----------QD----

--------RPVPYASRALTSA-E-------N---Y-SQI-KEMLAIWFAC-------AKF

HQYVY-----------GK-S-----IE----------VHTDHRP-LEPILKKPIAK----

-ASPR-LQ-MMMQ-LQ-R---------YTLEVKYI---P-GKY----------MY-----

VADTLSR------------------------------------

>LLGY92

------------------------------------------------------------

------------------------------------------QH--------SLSR----

--------RSGSYGSRALSDVET-------R---Y-SQTEREALAVVWAC-------EHF

DKFIN-----------GAPQ-----FT----------VISDHKP-LETIWKKPR------

-PPLR-IERWGLR-LQ-P---------YRMVIKYQ---P-GSD-----------N-----

PADYMSR------------------------------------

>LLGY93

--------------------------------------IIAADA-----SG---------

-----------HGLGA---------------------VL--MQVQ-----D-NGDR----

--------RPVCFASRALTAVEQ-------R---Y-AVIEKEALAATWSC-------DKF

ADYVL-----------GM-T-----FT----------LETDHKP-LVSLLS-STDL---A

KMPPR-IQRFRMC-MM-R---------YNPEVQYV---Q-GHL----------HV-----

SADALSR------------------------------------

>LLGY95

-------------------------------------LTIQCDA-----SQ---------

-----------SGLGA---------------------AL--LQ-----------EG----

--------RPVAFASRALSETET-------R---Y-AQIEKEMLAIVFAA-------ERF

HQYTF-----------GR-R-----VT----------VLSDHKP-LENIMKKPLGI----

-APKR-LQGMMLR-LQ-R---------YDVEVVYL---Q-GKQ----------MF-----

LADTLSR------------------------------------

>LLGY96

----------------------------------------FVDA-----SP---------

-----------LGLGA---------------------IL--TQN-----------G----

--------KVVSYASRALSDVER-------R---Y-SQTEREMLAVVWSA-------EHF

HLYLY-----------GAS------FT----------IHTDHQP-LLGIF-KNQR-----

PASAR-IERWRLR-LL-P---------YQFELKYR---P-GKDD---------RN-----

PADYISR------------------------------------

>LLGY97

--------------------------------------ALTVDA-----LG---------

-----------AAIGA---------------------VL--EQNL--------GSW----

--------KPVAFFSHKLRAAEQ-------K---Y-SAFDRELLAAYLAI-------RHF

RYFLE-----------GR-S-----FV----------LFTDHKP-LTFAISKTSD-----

PWSSR-QQRHLTY-IS-E---------FTTDVRHI---G-GKN----------NT-----

IADTLSR------------------------------------

>LLGY98

---------------------------------------LEVDA-----SK---------

-----------KGLGV---------------------AL--IQ-----------DE----

--------RPVAFGSKTLTECQS-------R---Y-SNIERELLAVVHGI-------QRY

HTYLY-----------GR-S-----FT----------VIT--------------------

------------------------------------------------------------

-------------------------------------------

>LLGY99

--------------------------------------VVQVDA-----SS---------

-----------RGLGA---------------------VL--MQ-----------GG----

--------RPIAFASKSLTDCER-------R---Y-ANIEREMLAVVFGC-------ERF

HTYVY-----------GK-H-----FT----------VESDHKP-LEMIHLKNLAA----

-APQR-LQRMLLR-VQ-P---------YDFKLHYI---P-GKN----------IA-----

LADTMSR------------------------------------

>LLGY100

---------------------------------------LRPDW-----SK---------

-----------LGIGY---------------------FL--SQKHC----DCATTL----

PGCCDDGWKITLAGSRFLTPTEQ-------R---Y-APIEGEALAVAWGL-------EQS

RYFTQ-----------GCDD-----LV----------VVTDHKP-LVKILGDRTL-----

--DEI-NNSRIFR-LK-QRTL-----PWHFEVTHL---P-GKT----------NT-----

AADATSR------------------------------------

>LLGY101

---------------------------------------LIVDA-----SP---------

-----------VGLGA---------------------IL--AQKES----EEEEGG----

--------RIVAYASRALSEVEQ-------R---Y-SQTEREALAVAWGT-------EKF

HLYIY-----------GKS------VE----------IITDHKP-LEGLFNNPRS-----

KPNAR-IERWLMR-MQDK---------YDYKVTYR---P-GKNP---------EN-----

PADYMSR------------------------------------

>LLGY102

--------------------------------------VLQTDA-----SS---------

-----------VGLGA---------------------VL--LQ-----------QG----

--------RPVAYASRALTDCER-------N---Y-APLELECLAIVFAT-------SKF

DQYVF-----------GHPD-----VT----------IHTDHRP-LEAILHKSLLR----

-APKR-LQAMILS-LQ-R---------YTLKVVYK---P-GTE----------QV-----

IADMQSR------------------------------------

>LLGY103

-------------------------------------VKILSDA-----SQ---------

-----------CGLDA---------------------VI--LQ-------QHDGEW----

--------QPVAYASRAMTSAET-------R---Y-AQIEKELLSIMFAC-------ERF

HQYIY-----------GQ-A-----VI----------VETDHKP-LVNLFHKSLND----

-YSLR-IQRLMIR-LQ-K---------YTVNVSYT---P-GKF----------MH-----

TADALSR------------------------------------

>LLGY104

------------------------------------------------------------

------------------------------------------------------------

----------------------R-------N---Y-SQIEKEMLAICFAC-------AKF

HQYVY-----------GK-S-----IE----------VHTDHRP-LESILKKSIAK----

-ASPR-LQRVMLQ-LQ-R---------YTLEVKYI---P-GKY----------MY-----

VAHTLSR------------------------------------

>LLGY105

--------------------------------------TIQCDA-----SQ---------

-----------SGLGA---------------------VL--LQ-----------KG----

--------KPVCYASRALTHTEE-------N---Y-AQIEKELLAIVFAC-------ERF

DQYVY-----------GR-H-----IT----------VQSDHKP-LEIITKKSMID----

-APKR-LQRMLLR-LQ-K---------YNIDVVYT---K-GKD----------ML-----

IADTLSR------------------------------------

>LLGY106

--------------------------------------TLSADA-----SS---------

-----------HGLGA---------------------VL--LQRQ-----P-DGKL----

--------QPVAYASRSMSSTEQ-------R---Y-AQIEKEALATTWAC-------ERF

SDFLL-----------GK-T-----FH----------VETDHKP-LVSLLG-QKTL---D

QLPPR-IQRFRMR-LM-R---------FNYSIAHV---A-GKD----------LI-----

TADTLSR------------------------------------

>LLGY107

------------------------------------------------------------

------------------------------------------------------------

--------ESSADGSRFTHGAES-------R---Y-APIEGEALAVVDAL-------DKV

RHFTL-----------GCSD-----LR---------------------------------

------------------------------------------------------------

-------------------------------------------

>LLGY108

--------------------------------------ILSCDA-----SP---------

-----------YGVGA---------------------VL--SHVM-----E-DGSE----

--------RPIGFVSRTLAPAEK-------K---Y-SQLDKEGLAIIFGI-------KKF

HQHLY-----------GR-S-----FK----------ITSDHKP-LLGLLGENKGVP--V

MASAR-MQRWALT-LA-A---------YEYRLVYK---G-GKD----------NG-----

NSDALSR------------------------------------

>LLGY110

--------------------------------------VLQCDA-----ST---------

-----------QGIGT---------------------SL--TQ-----------DG----

--------NPVAYVSRSLTKCEQ-------K---Y-APIELECLAIVFAC-------RKF

DQYIY-----------GHAK-----VT----------IHSDHKP-LKAIFRKSLLE----

-APKR-LQRTMLA-VQ-R---------YNVKVEYK---P-GTE----------QL-----

VADMLSR------------------------------------

>LLGY111

------------------------------------------------------------

--------------WP---------------------VI--SQDQ-------DERR----

--------VVIAYASRRLRPPER-------Q---Y-SSMKLEMLALKWAVT------TKF

RAYLY-----------GR-E-----FV----------IYTDNNP-L-KYIQTAKL-----

--GAV-EQRWAAE-LA-P---------FNFTIEYR---E-GRL----------NA-----

NADALSR------------------------------------

>LLGY112

------------------------------------------------------------

------------------------------------------------------------

--CCPDGWRITLVGSRFTHPAES-------R---Y-APIEGEALSVADAL-------DKA

RFFVL-----------GCAD-----LI----------VAVDHKP-LLKILGDRSL-----

--DEI-ANARLRN-LK-EKTL-----RYRFRIVHI---P-GVQ----------NK-----

AADAMSR------------------------------------

>LLGY113

--------------------------------------VLTCDA-----SP---------

-----------YGVGA---------------------VL--AHAF-----D-DRVE----

--------RPIAYYSRSLSAAEK-------N---Y-AQIDKEGLAVIAGL-------TKF

HQYLW-----------GR-P-----FL----------IVTDHKP-LLGLFGEQKAVP--Q

MLSPR-MQRWALT-LA-A---------YEYQIVHR---P-GSS----------IP-----

QADALSR------------------------------------

>LLGY114

--------------------------------------VLENDA-----SE---------

-----------YGLGS---------------------VL--LQ-----------DG----

--------KPVAYASRSLSSAER-------H---Y-AQIEKEMLSVLFGL-------NKF

HHYTY-----------GR-D-----VN----------VVTDHKP-LVAIRAK--------

------------------------------------------------------------

-------------------------------------------

>LLGY115

--------------------------------------CLTTDW-----SK---------

-----------EGIGF---------------------FL--LQKHC----ACPGEV----

PFCCNDGWKVTLVGSRFTHPAES-------R---Y-APIEGEALAVADAL-------ERT

RYFVL-----------GCDD-----LI----------VAVDHQP-LLKVLGDRKL-----

--EDI-KNPRLLN-LK-EKTL-----PFKFKLIHI---P-GKR----------HL-----

ATDAISR------------------------------------

>LLGY116

--------------------------------------VVQVDA-----SS---------

-----------RGLGA---------------------VL--LQ-----------KN----

--------KPIAFASKSLSDCER-------R---Y-VNIEREMLAVVFGC-------ERF

HTFVY-----------GK-R-----FT----------VESDHKP-LEMIHMKNLAA----

-APQR-LQRMLLR-IQ-P---------YDIVIKYR---P-GKD----------VA-----

VADLLSR------------------------------------

>LLGY117

---------------------------------------IFVDA-----SP---------

-----------IGLGA---------------------VL--TQENA-----ISKEV----

--------TPLYFASRPLTPTES-------R---Y-PQIDREALAISWAI-------KRF

HLYAY-----------GKE------FK----------VVTDHKP-LVTLFNNPSS-----

KPTAR-IERWLMD-LL-R---------YNFTVTYQ---P-GKS-----------N-----

PADYASR------------------------------------

>LLGY118

--------------------------------------IIAADA-----SG---------

-----------HGLGT---------------------VL--MQVQ-----D-NGDR----

--------RPVCFASRALTAVEQ-----------Y-AVIEKEALA------------DKF

ADYVL-----------GM-T-----FT----------LETDYKP-LVPLLS-STDL---A

KMPPL-IQRFRMC-MM-R---------YNPEVQYI---Q-GRR----------RI-----

VSC----------------------------------------

>LLGY119

--------------------------------------VLQTDA-----SS---------

-----------VGIGA---------------------VL--LQ-----------QG----

--------RPVAYASRALTDCER-------N---Y-APLELECLAIVFAT-------TKF

DQYVF-----------GHPD-----VT----------IHTDYRP-LEAILHKSLLR----

-APKR-LQSMILA-LQ-R---------YTLKVVYK---P-GTE----------QV-----

IADMLSR------------------------------------

>LLGY120

---------------------------------------LRPDW-----SK---------

-----------LGIGY---------------------FL--SQKHC----DCATTL----

PGCCDDGWKITLAGSRFLTPTEQ-------R---Y-APIEGEALAVAWGL-------EQS

RYFTQ-----------GCDD-----LV----------VVTDHKP-LVKILGDRTL-----

--DEI-NNSRIFR-LK-QRTL-----PWHFEVTHL---P-GKT----------NT-----

AADATSR------------------------------------

>LLGY121

--------------------------------------VVQVDA-----SS---------

-----------RGLGA---------------------VL--MQ-----------GG----

--------RPIAFASKSLTDCER-------R---Y-ANIEREMLAVVFGC-------ERF

HTYVY-----------GK-H-----FT----------VESDHKP-LEMIHLKNLAA----

-APQR-LQRMLLR-VQ-P---------YDFKLHYI---P-GKN----------IA-----

LADTMSR------------------------------------

>LLGY122

--------------------------------------VVQVDA-----SG---------

-----------RGLGA---------------------VL--LQ-----------GG----

--------KPIAFASKSLSECEK-------R---Y-ANIEREMLAVVFGC-------ERF

HTYVY-----------GT-R-----FT----------VESDHKP-LEMIHLKNLAA----

-APQR-LQRMLLR-IQ-P---------YDIQLWYR---P-GKE----------LA-----

LADTMSR------------------------------------

>LLGY123

-------------------------------------LTIQCDA-----SQ---------

-----------SGLGA---------------------AL--LQ-----------EG----

--------RPVAFASRALSETET-------R---Y-AQIEKEMLAIVFAA-------ERF

HQYTF-----------GR-R-----VT----------VLSDHKP-LENIMKKPLGI----

-APKR-LQGMMLR-LQ-R---------YDVEVVYL---Q-GKQ----------MF-----

LADTLSR------------------------------------

>LLGY124

--------------------------------------SITVDA-----SS---------

-----------TAVGG---------------------AL--EQYI-------NGQW----

--------QPLAFFSRNLKPAET-------R---Y-SAFDRELLAMYLAV-------RHF

RYFLE-----------GR-L-----FH----------IYTDHKP-ITFAFHNNAD-----

R-SPR-QTRHLSF-IA-E---------FTTDVRYI---P-GKT----------NV-----

AADMLSR------------------------------------

>LLGY125

--------------------------------------VVECDA-----SE---------

-----------RGLGA---------------------AL--LQ-----------NG----

--------KPIGYASRALTSTET-------R---Y-AQIEKECLAIVFAL-------EHF

HQYTF-----------GR-R-----TI----------VHTDHKP-LEMTVKKPLYT----

-APRR-LQGMLLR-ML-Q---------YDTEVVYH---K-GKE----------MY-----

IADTLSR------------------------------------

>LLGY126

--------------------------------------ILETDA-----SH---------

-----------QGLGA---------------------LL--LQEQ-------DGKT----

--------RVIAYASRGLRGPEK-------NRTAY-SSMKLELLAVKWAVT------EKF

RDYLL-----------GV-Q-----FV----------IYTDNNP-L-SYIQTSAKL----

--TAA-EHHWQAE-LA-R---------FNFSIHYR---P-GRL----------NA-----

SADGLSR------------------------------------

>LLGY127

---------------------------------------IFVDA-----SP---------

-----------IGLGT---------------------VL--TQENA-----ISKEV----

--------TPLYFASRPLTPT-S-------R---Y-PQIDRAALAISWAI-------KRF

HLYAY-----------GKE------FK----------VVTDHKP-LVTLFNNLSS-----

KPTAR-IERWLMD-LL-R---------YNFTVTYQ---P-GKS-----------N-----

PADCASR------------------------------------

>LLGY128

--------------------------------------VLETDA-----SF---------

-----------KGLGA---------------------VL--SQEQ-------DGRL----

--------RVIAYASRSLRGSEK-------NMENY-SSFKLELLALKWAIT------EKF

RDYLI-----------GS-T-----FT----------VYTDNNP-L-SYIQSTAKL----

--GAI-EQRWAGQ-LA-L---------FSFQIKYR---S-GRS----------NR-----

NADALSR------------------------------------

>LLGY129

--------------------------------------TIQCDA-----SE---------

-----------TGLGA---------------------VL--LQ-----------EG----

--------QPICYASRALTDTET-------R---Y-AQIEKELLAIVWSC-------DKF

DQYIY-----------GRDM-----VT----------IESDHEP-LKAVFKKDIHK----

-SPKR-LQRMCLA-LQ-K---------YNLDVQYK---K-GSL----------MY-----

ISDTLSR------------------------------------

>LLGY130

---------------------------------------LEVDA-----SK---------

-----------KGLGV---------------------AL--IQ-----------DE----

--------RPVAFGSKTLTECQS-------R---Y-SNIERELLAVVHGI-------QRY

HTYLY-----------GR-S-----FT----------LITDHKP-LVTICVNPLHA----

-APPR-LQRMLLK-IQ-G---------YYFDIVYR---P-GEQ----------MT-----

LADALSR------------------------------------

>LLGY131

---------------------------------------LEVDA-----SK---------

-----------KGLGV---------------------AL--IQ-----------DE----

--------RPVAFGSKTLTECQS-------K---Y-SNIERELLAVVHGI-------QRY

HTYLY-----------GR-S-----FT----------VITDHKP-LVTICAKPLHA----

-APPR-LQRMLLK-IQ-G---------YNFDIVYR---P-GEQ----------MT-----

LADALSR------------------------------------

>LLGY132

--------------------------------------ALTVDA-----SG---------

-----------TAIGA---------------------VL--EQDL--------GSW----

--------KPVAFFSRKLRPAEQ-------K---Y-SAFDRELLAMYLAI-------RHF

RYFLE-----------GR-T-----FT----------LYTDHKP-LTFAISKVSD-----

PWSPR-QQRHLAY-IS-E---------FTTDVRHI---E-GKN----------NT-----

VADTLSR------------------------------------

>LLGY133

--------------------------------------VLQTDS-----SS---------

-----------VGLGA---------------------VL--FQ-----------QG----

--------RPVA-ASRALTDCER-------N---Y-AP--LECLAIVFAT-------TKF

DQYIF-----------GHPY-----VT----------IHTDHRP-LEAKLHKSLLR----

-APKR-LQAMI-------------------------------------------------

-------------------------------------------

>LLGY134

---------------------------------------LSADA-----SS---------

-----------HGLGA---------------------VL--LQRQ-----P-DSKF----

--------QPVAYASRSMSSTEQ-------R---Y-TQIEKEALATTWAC-------ERF

SDFLL-----------GK-T-----FH----------IETDHKP-LVSLLG-QKTL---D

QLPPR-IQRFRMR-LM-R---------FNYSIAHV---A---D----------LI-----

TADTLSR------------------------------------

>LLGY135

--------------------------------------CLTTDW-----SK---------

-----------EGIGF---------------------FL--LQKHC----ACPGEV----

PFCCNDGWKVTLVGSRFTHPAES-------R---Y-APIEGEALAVADAL-------ERT

RYFVL-----------GCDD-----LI----------VAVDHQP-LLKVLGDRKL-----

--EDI-KNPRLLN-LK-EKTL-----PFKFKLIHI---P-GKR----------HL-----

ATDAISR------------------------------------

>LLGY136

-----------------------------------------TD-----------------

--------------GA---------------------LL--LQEQ-------DGKT----

--------RVIAYASRGLRGPEK-------NRTAY-SSMKLELLAVKWAVT------EKF

RDYLL-----------GV-Q-----FV----------IYTDNNP-L-SYIQTSAKL----

--TAA-EHHWQAE-LA-R---------FNFSIHYR---P-GRL----------NA-----

SADGLSR------------------------------------

>LLGY137

---------------------------------------LIMDA-----CP---------

-----------VGLGA---------------------IL--AQKES-------EGG----

--------RIVAYASRALSEVEQ-------R---Y-SQTEREALAVAWGT-------EKF

HLYIY-----------GKS------VE----------IITDHTP-LEGLFNNPRS-----

KPNAR-IERWLMR-MQDK---------YDYKVTYR---P-GKNP---------EN-----

PADYMAR------------------------------------

>LLGY138

--------------------------------------TIQVDA-----SG---------

-----------RGLGA---------------------VL--LQ-----------EG----

--------RPIAYASKSLTDTEK-------R---Y-ANIERELLAVVFGA-------ERF

RTYVY-----------GK-H-----FV----------VESDHKP-LEMIQLKNLMA----

-APPR-LQRMLLR-IQ-H---------YDITIKYR---P-GKE----------LL-----

LADGLSR------------------------------------

>LLGY140

------------------------------------------NP-----HK---------

-----------K------------------------------------------------

---------------------HA-------N---Y-SNIEREMLALVHGV-------ERF

HAYLY-----------GR-S-----FT----------ITTDHKP-LEMICSKPIAS----

-APPR-LQRMLVK-IQ-G---------YDYSVKSR---P-GKE----------MV-----

MSDALSR------------------------------------

>LLGY141

----------------------------------------FVDA-----SP---------

-----------LGLGA---------------------IL--TQN-----------G----

--------KVVSYASRALSDVER-------R---Y-SQTEREMLAVVWSA-------EHF

HLYLY-----------GAS------FT----------IHTDHQP-LLGIF-KSQR-----

PASAR-IERWRLR-LL-P---------YQFELKYR---P-GKDD---------RN-----

PADYISR------------------------------------

>LLGY142

--------------------------------------TIQCDA-----SQ---------

-----------SGLGA---------------------AL--MQ-----------NG----

--------QPVAYASRALTPPET-------R---Y-AQIEKELLAIVFAC-------DRF

EAYIY-----------GRDR-----VS----------IESDHKP-LETIVLKPLSS----

-APKR-LQRMLLR-LQ-K---------YTLDVKFK---K-GEH----------MY-----

LADTLSR------------------------------------

>LLGY143

------------------------------------------------------------

------------------------------------------------------------

----------------SLTRAEQ-------K---Y-SQIEREGLGIFFGV-------KRF

YPFLF-----------GR-K-----FT----------LVTDNKT-LAAIISRRMDIP--A

VAAER-IQRWAMY-LS-G---------FNYDVRYQ---S-SAQ----------NG-----

NADWLSR------------------------------------

>LLGY144

---------------------------------------VSSDA-----SK---------

-----------DGIGA---------------------VL--LQ-------ETNGEW----

--------MPVAYASRSMTAAEK-------N---Y-AQIEKEQLGVVFAC-------ERF

HVYIY-----------GR-K-----VI----------VETDHQP-LIAISKKQLCD----

-APPR-LQRLLLR-IQ-K---------YDLMLEYT---P-GKL----------LV-----

LLGY145

--------------------------------------VVQVDA-----SS---------

-----------RGLGA---------------------VL--LQ-----------KN----

--------KPIAFASKSLSDCER-------R---Y-VNIEREMLAVVFGC-------ERF

HTFVY-----------GK-R-----FT----------VESDHKP-LEMIHMKNLAA----

-APQR-LQRMLLR-IQ-P---------YDIVIKYR---P-GKD----------VA-----

VADLLSR------------------------------------

>LLGY146

-----------------------------------------ADA-----SQ---------

-----------RGLGA---------------------CL--LQ-----------QG----

--------RPVAYASRALTSAKE-------N---Y-SQIEKEMLAICFAC-------AKF

HQYVY-----------GK-S-----IE----------VHTDHRP-LESILKKPIAK----

-ASPR-LQRVMLQ-LQ-R---------YTLEVKYI---P-GKY----------MY-----

VADTISR------------------------------------

>LLGY147

--------------------------------------ALHTDA-----SR---------

----------RKGLGY---------------------AL--LQKH-------DEKW----

--------RLVQCGSRFLTDTES-------R---Y-AMVELELLAATWAM-------KKC

RIQLL-----------GMEH-----FE----------LVVDHKP-LVTILDRHRLD---D

VDNVR-LQRLKEK-TS-L---------FTFTTRWT---K-GKD----------HC-----

IPDALSR------------------------------------

>LLGY148

----------------------------------------IVDA-----SP---------

-----------VGLSG---------------------ML--VQY-------HDKKA----

--------RVMAYGSRALTAVEQ-------R---YRSQLKREALAVVWAC-------EYF

HLYIF-----------GAP------VK----------VVTDHKP-LVTLYGNPSA-----

KLPLR-LERWAMR-LL-P---------YQPIIEY------GKGC---------EN-----

PSDYLSR------------------------------------

>LLGY149

--------------------------------------VIQCDA-----SN---------

-----------YGLGN---------------------AL--LQ-----------DG----

--------RPIAYASRALTDAET-------R---Y-AIIEKEMLAVVFAL-------EKW

NQFTY-----------GR-P-----IL----------VYSDHKP-FEAITKKPLDR----

-APKH-LQGTLLR-AL-A---------YDIDVKYL---E-GKK----------MS-----

LVDTLSR------------------------------------

>LLGY150

--------------------------------------TVACDA-----SP---------

-----------VGLGV---------------------VL--SHIM-----P-DGEE----

--------QPIQFASRS-TRAEQ-------K---Y-SQIERERLGIIFGV-------KRF

YPFLF-----------GR-K-----FT----------LVTDNKP-LAAIISPRKNIP--A

VAAER-IQRWAMY-LS-G---------FTYDVRYR---S-SAQ----------NA-----

NADWLSR------------------------------------

>LLGY151

--------------------------------------ILETDA-----SH---------

-----------QGLGA---------------------LL--LQEQ-------DGKT----

--------RVIAYASRGLRGPEK-------NRTAY-SSMKLELLAVKWAVT------EKF

RDYLL-----------GV-Q-----FV----------IYTDNNP-L-SYIQTSAKL----

--TAA-EHHWQAE-LA-R---------FNFSIHYR---P-GRL----------NA-----

SADGLSR------------------------------------

>LLGY152

--------------------------------------SITVDA-----SS---------

-----------TAVGG---------------------AL--EQYI-------NGQW----

--------QPLAFFSRNLKPVET-------R---Y-SALDRELLAMYLAV-------RHF

RYFLE-----------GR-L-----FH----------IYTDHKP-ITFAFHNNAD-----

--SPR-QTRHLSF-IA-E---------FTMDVRYI---P-GKT----------NV-----

AADMLSR------------------------------------

>LLGY153

--------------------------------------ILSCDA-----SP---------

-----------YGVGA---------------------VL--SHVM-----E-DGSE----

--------RPIGFVSRTLAPAEK-------K---Y-SQLDKEGLAIIFGI-------KKF

HQHLY-----------GR-S-----FK----------ITSDHKP-LLGLLGENKGVP--V

MTSAR-MQRWALT-LA-A---------YEYRVQRR---K---------------------

-------------------------------------------

>LLBP2

--------------------------------------HNFSDA-----S----------

----------LTGYGQ-------------C-------SY--LRL-----MDSHDNISCAL

----------VMGKSRVGPSRFV-------------TIPRLELTAAVLSVKVG----NFL

QKELD------------YE--AIT-HY----------YWTDSKV-VLGYINN-ESR----

RFN-IFVANRVQQ-IR-SSTK-------VSAWKYV---G-TRK----------NP-----

-ADIASR------------------------------------

>LLGY154

--------------------------------------TIQCDA-----SQ---------

-----------SGLGA---------------------AL--MQ-----------NG----

--------QPVAYASRALTPPET-------R---Y-AQIEKELLAIVFAC-------DRF

EAYIY-----------GRDR-----VS----------IESDHKP-LETIVLKPLSS----

-APKR-LQRMLLR-LQ-K---------YTLDVKFK---K-GEH----------MY-----

LADTLSR------------------------------------

>LLGY155

--------------------------------------CLTTDW-----SK---------

-----------EGIGF---------------------FL--LQKHC----ACPGEV----

PFCCNDGWKVTLVGSRFTHPAES-------R---Y-APIEGEALAVADAL-------ERT

RYFVL-----------GCDD-----LI----------VAVDHQP-LLKVLGDRKL-----

--EDI-KNPRLLN-LK-EKTL-----PFKFKLIHI---P-GKR----------HL-----

ATDAISR------------------------------------

>LLGY156

-----------------------------------------TDA-----CD---------

-----------KGLGA---------------------VL--SQMQ-------DGKL----

--------RVIAYASRSLRGAEK-------NDVNY-SAMRLETLALKWAVT------EKF

RDYLL-----------GG-T-----FT----------VYTDNNP-L-TYLNKKVKL----

--TAV-EQRWAAS-LA-P---------FNFDIRYR---P-GHC----------NA-----

NADGLSR------------------------------------

>LLGY157

--------------------------------------TIQCDA-----SQ---------

-----------SGLGA---------------------AL--MQ-----------NG----

--------QPVAYASRALTPPET-------R---Y-AQIEKELLAIVFAC-------DRF

EAYIY-----------GRDR-----VS----------IESDHKP-LETIVLKPLSS----

-APKR-LQRMLLR-LQ-K---------YTLDVKFK---K-GEH----------MY-----

LADTLSR------------------------------------

>LLGY158

--------------------------------------TLQSDA-----SK---------

-----------SGLGA---------------------CL--LQ-----------EG----

--------QIVAYASRALVGPEL-------H---Y-AQLEKELLAVVFAT-------SRF

HQYIY-----------GK-E-----IE----------AQTDHKP-LEIIMKKPIGN----

-ATAR-VQRMMLK-LQ-R---------YEINLSYV---P-GKL----------LY-----

VADALSR------------------------------------

>LLGY159

--------------------------------------VLATDA-----SP---------

-----------YGIGA---------------------VI--SHVL-----P-DGSE----

--------EPIAFASKTLSKAER-------G---Y-AQVEKEGLSIVYGI-------RKF

NQYLS-----------GR-H-----FT----------ILTDHKP-LLTIFGPDKSLP--A

MSLQR-LQRWALL-LM-G---------HDYDIRYR---A-SAE----------HC-----

NADALSR------------------------------------

>LLGY160

---------------------------------------VACDA-----SA---------

-----------YGLGA---------------------VL--SHKM-----P-DGLE----

--------KPIAFASQTLNKAER-------N---Y-SQIDKETLGLVWGV-------RKY

NQYLF-----------GR-R-----FT----------LVTDHQP-LTAIFHPGKSIP--A

MTAAQ-MQRYALQ-LA-A---------HDYDIVYK---S-SLK----------HA-----

NADGLSR------------------------------------

>LLGY161

--------------------------------------TVSADA-----SS---------

-----------YGIGC---------------------VL--LQES-------DGKQ----

--------HPAAFCSRTLTPTEQ-------Q---Y-AHIEKECLTCVWSC-------EKY

------------------------------------------------------------

------------------------------------------------------------

-------------------------------------------

>LLGY162

--------------------------------------CIQCDA-----SD---------

-----------GGLGA---------------------RL--LQ-----------DG----

--------LPVVYASRALTATER-------N---Y-AQIEKELLAIAFAC-------EKF

DQYVY-----------GREN-----VH----------VQSDHKP-LEVIFRKPLVT----

-VPKR-LQRMLLR-LQ-R---------YSLDVTYT---R-GSE----------MY-----

IADTLSR------------------------------------

>LLGY163

--------------------------------------VVQVDA-----SS---------

-----------RGLGA---------------------VL--MQ-----------GG----

--------RPIAFASKSLTDCER-------R---Y-ANIEREMLAVVFGC-------ERF

HTYVY-----------GK-H-----FT----------VESDHKP-LEMIHLKNLAA----

-APQR-LQRMLLR-VQ-P---------YDFKLHYI---P-GKN----------IA-----

LADTMSR------------------------------------

>LLGY164

--------------------------------------TLTCDA-----SQ---------

-----------HGLGA---------------------AC--LQ-----------NG----

--------RPVAYASRALTETET-------R---Y-AQIEKELLAVVFAC-------TKF

KNYIC-----------AK-Q-----VT----------VETDHQP-LISITKKPLSA----

-APAR-LQRMLMR-LQ-S---------HDIHLVYR---K-GKD----------LV-----

LADALSR------------------------------------

>LLGY165

--------------------------------------VLATDA-----SP---------

-----------YGIGA---------------------VI--SHVL-----P-DGSE----

--------EPIAFASKTLSKAER-------G---Y-AQVEKEGLSIVYGI-------RKF

NQYLS-----------GR-H-----FT----------ILTDHKP-LLTIFGPDKSLP--A

MSLQR-LQRWALL-LM-G---------HDYDIRYR---A-SAE----------HC-----

NADALSR------------------------------------

>LLGY166

---------------------------------------LQCDA-----SL---------

-----------RGIGA---------------------AL--LQ----P--DADGEL----

--------RSVEYASKSLTPTEQ-------R---Y-ACIERELLSIVFGM-------QRF

HTYLY-----------GR-D-----FN----------VITDHRP-LLMITNKPIAS----

-APPR-LQRMLIK-LH-G---------YNFTMTHR---P-GSQ----------NQ-----

LADGLSR------------------------------------

>LLGY167

--------------------------------------VLTCDA-----SP---------

-----------YGVGA---------------------VL--AHAF-----D-DRVE----

--------RPIAYYSRSLSAAEK-------N---Y-AQIDKEGLAVIAGL-------TKF

HQYLW-----------GR-P-----FL----------IVTDHKP-LLGLFGEQKAVP--Q

MLSPR-MQRWALT-LA-A---------YEYQIVHR---P-GSS----------IP-----

QADALSR------------------------------------

>LLGY168

--------------------------------------KLYTDA-----CD---------

-----------YAIGG---------------------IL--VQESV------DGIE----

--------KVIQYVSHTLSPTQR-------K---W-ATIEKEAFAVVFSI-------LKL

RPYLY-----------GA-Q-----FN----------VYTDHKP-LLSLFTKAFN-----

--NTK-IQRWGVL-LA-E---------YGTTISYR---T-GRN----------NI-----

RADMLSR------------------------------------

>LLGY169

--------------------------------------VAYSDA-----DYAGDVN----

--------DRKSTSGF-------------V-------FL--K------------------

------NGAAISWRSKKT------------------------------------------

------------------------------------------------------------

------------------------------------------------------------

-------------------------------------------

>LLGY170

--------------------------------------TRTVDA-----SS---------

-----------TSLGA---------------------AL--LQ-----------DG----

--------GPVEYAAKALTGAQI-------N---Y-AQIEKKLQAMVFGC-------ERF

HSYIY-----------GR-E-----VT----------VETDHKP-LIGVFNKQIET----

-ASSR-IQRMMLK-LQ-R---------Y--------------------------------

--DCLQAR-----------------------------------

>LLCO2

------------------------------------------------------------

------------------------------------------F-------GENGRF----

--------SPLSWQSKRIRRVVR-------------STLAGE----ADGVDSGMFLATLF

AELT-----------TGKAKPELLPIS----------CVTDNHS-LYDAVKSAKFV----

-PDKR---------LR-----------LEITTLKS-------------------------

-------------------------------------------

>LLGY172

--------------------------------------LLQCDA-----SN---------

-----------YGLRL---------------------VF--SH-------Q-DLWE----

-----------GFAPRTLYAAEK-------N---C-LQLDKEGAAVMFAQ-------KKF

HKHLY-----------GR-S-----FE----------IITDHKP-LMSLFGELKQVP--T

TASLR-IQ----------------------------------------------------

-------------------------------------------

>LLGY173

--------------------------------------CLTTDW-----SK---------

-----------EGIGF---------------------FL--LQKHC----ACPGEV----

PFCCNDGWKVTLVGSRFTHPAES-------R---Y-APIEGEALAVADAL-------ERT

RYFVL-----------GCDD-----LI----------VAVDHQP-LLKVLGDRKL-----

--EDI-KNPRLLN-LK-EKTL-----PFKFKLIHI---P-GKR----------HL-----

ATDAISR------------------------------------

>LLGY174

-----------------------------------------TDA-----CD---------

-----------KGLGA---------------------VL--SQMQ-------DGKL----

--------RVIAYASRSLRGAEK-------NDVNY-SAMRLETLALKWAVT------EKF

RDYLL-----------GG-T-----FT----------VYTDNNP-L-TYLNKKVKL----

--TAV-EQRWAAS-LA-P---------FNFDIRYR---P-GHC----------NA-----

NADGLSR------------------------------------

>LLGY175

--------------------------------------VIQCDA-----SN---------

-----------YGLGS---------------------AL--LQ-----------DG----

--------RPIAYASRALTDAET-------R---Y-AIIEKEMLAIVFAL-------DKW

HQFTY-----------GR-P-----IL----------VNSGHKP-LEAITKK--------

------------------------------------------------------------

-------------------------------------------

>LLGY176

----------------------------------------ECDA-----SE---------

-----------RGLGA---------------------AL--LQ-----------NG----

--------KPIGYASRALTSTET-------R---Y-AQIEKECLAIVFAL-------ERF

HQYTF-----------GR-R-----TI----------VHTDHKP-LEMIVKKPLYK----

-APGR-LQGMLLR-ML-Q---------YDTEVVYH---K-GKE----------MY-----

IADTLSR------------------------------------

>Athila41

--------------------------------------EIMCDA-----SD---------

-----------YAVGA---------------------VL--GQKI-----D-KK-L----

--------HVIYYASRTLDDAQG-------R---Y-ATTEKELLAVVFAF-------EKF

RSYLV-----------G-SK-----VT----------VYTDH-A-L-RHLYAKKD-----

-TKPR-LLRWILL-LQ-E---------FDMEIVDK---K-GIE----------NG-----

AADHLSR------------------------------------

>Diaspora

--------------------------------------ELMCDA-----SN---------

-----------YALGA---------------------VL--AQKI-----D-KL-P----

--------RVIYYASRTLDAAQA-------N---Y-TTTEKELLAIVFAL-------EKF

RSYLL-----------G-TH-----II----------VYTDHAA-L-KYLLKKVD-----

-SKPR-LIRWMLW-LQ-E---------FDLEIRDR---S-GAQ----------NL-----

VADHLSR------------------------------------

>Cyclops2

--------------------------------------ELMCDA-----SN---------

-----------YAIGA---------------------VL--GQRK-----E-KK-F----

--------HAIHYASKVLMRL-I-------N---Y-ATTEKELLAIVYAL-------EKF

RSYLI-----------G-SK-----VV----------VYTNHSA-I-KYLLTKPD-----

-SKQR-LIRWILL-LQ-E---------FDVEIKDK---K-GSE----------NL-----

VADHLSR------------------------------------

>Bagy2

--------------------------------------EIMCGA-----SD---------

-----------YAVGA---------------------VL--GQRV-----D-KK-L----

--------NVIHYASKSLDRAQR-------N---Y-ATTEKEFLAVVFAC-------EKF

RSYIV-----------D-SK-----VI----------IHSDHAA-I-KYLMEKKD-----

-AKPR-LIRWVLL-LQ-E---------FDLHVVDR---K-GAD----------NP-----

VADNLSR------------------------------------

>Calypso

--------------------------------------ELMCDA-----SD---------

-----------YAIGA---------------------VL--GQRK-----G-KI-F----

--------HAIYYASKVLNDAQV-------N---Y-ATTEKEMLAIVYAL-------EKF

KSYLV-----------G-SK-----VI----------IYIDHAT-I-KYFLNKAN-----

-SKTL-LIRWILL-LQ-E---------FDLVIRDK---K-GSE----------NV-----

VANQFV-------------------------------------

>Gloin

--------------------------------------VVEYDA-----SG---------

-----------FGLGA---------------------VL--KQ-------D----H----

--------QPIAYFSYGLTAREQ-------L---K-PIYERELMTIVMAV-------QKW

RHYLL-----------G-RR-----FV----------VHTDQKS-L-KFLLEQRE-----

-VSLE-YQNWLSK-LL-N---------YTFDIIYK---P-GID----------NK-----

AADGLSR------------------------------------

>Ifg7

--------------------------------------IVECDA-----SG---------

-----------NGIGA---------------------VL--MQ-------D----E----

--------IPIAFEGHPIRGKFL-------HK----ALYEKEMLAILHAL-------KKW

RPYLM-----------GR-H-----FN----------VKTDHDS-L-KYFLEQRL-----

--SSE-EQKWVTK-ML-G---------YDFEIIYK---K-GKK----------NV-----

VANALSR------------------------------------

>Reina

--------------------------------------VVETDA-----SG---------

-----------SGIGA---------------------VL--QQ-------K----G----

--------HPIAYISKALGPKNL-------G---L-STYEKEYLAILFAV-------DHW

RPYLQ-----------H-GE-----FF----------IKTDQQS-L-THLEDQKL-----

-STIW-QQKAITK-LL-G---------LQFRIIYK---K-GVE----------NR-----

VADALSR------------------------------------

>Gimli

--------------------------------------VVETDV-----CG---------

-----------QGISA---------------------FL--MQ-------N----G----

--------HPLAYISRHLQGKQL-------H---L-SIYEKELLAVVFAI-------QKW

RHYLL-----------H-DH-----FV----------IITDQRS-L-KYLLEQRL-----

-NTPI-QQQWLPK-LL-E---------FDYEIQYK---E-GKE----------NL-----

VADALSR------------------------------------

>Monkey

--------------------------------------EVHIDA-----SD---------

-----------FAIGG---------------------VL--MQ-------E----G----

--------HPVAYESRKLNETER-------R---Y-PVHEKEMTAVIHCL-------RVW

RHYLL-----------G-SR-----FV----------LRTDNIA-L-SYFQTQKK-----

-LSPK-QARW-DF-LV-E---------FDMAMEYK---P-GKA----------NV-----

VADALSR------------------------------------

>Tntom1

--------------------------------------EVQTDA-----SDS--------

----------ILSLGG---------------------VL--LQ-------D----E----

--------HPVAYQDRILK-NAR-------H---Y-AAHENELFVVVHYL-------RLW

RHYLV-----------G-AP-----FI----------VKTDNTV-V-SHLMT-PK-----

-LNGR-QARW-EL-LA-E---------FHFNLEYR---S-GKT----------NH-----

VANALSR------------------------------------

>Galadriel

--------------------------------------EVHTDA-----SD---------

-----------KAIGG---------------------VL--VQ-------E----G----

--------HPVAFESRKLNDAEQ-------R---Y-STHEKEMVAVVHCL-------QVW

RVYLL-----------G-TR-----FV----------VRTDNVA-N-TFFKTQKK-----

-LSPK-QARWQEF-LA-E---------YDFMWEHK---P-GKH----------NQ-----

VADALSR------------------------------------

>Del

--------------------------------------VVYTDA-----SL---------

-----------AGLEG---------------------VL--MQ-------D----G----

--------RVVAYASRQLKVHEN-------N---Y-PTHDLELAVVIFIL-------KLW

RHYLY-----------G-ED-----FE----------LYCDHKS-L-KYISTQKD-----

-LNLR-Q-RWIEV-LK-D---------FDFSIFYH---P-GKA----------NV-----

VADALSR------------------------------------

>Peabody

--------------------------------------EVYCDA-----SL---------

-----------LGLGG---------------------VL--MQ-------D----K----

--------QVIAYASRQLKVHER-------N---Y-PTHDLELAAVVFVL-------KLW

RHYLY-----------G-SR-----FE----------VFSDHKS-L-KYFFDQKE-----

-LNMR-QRRWLEF-LK-D---------YDFGLNYH---P-GKA----------NV-----

VADALSR------------------------------------

>Retrosat2

--------------------------------------QVYCDA-----SR---------

-----------HGLGC---------------------VL--MQ-------E----G----

--------RVVAYASRQLRPHEG-------N---Y-PTHDLELAAVVHAL-------KIW

RHYLI-----------G-NR-----CE----------VYTDHKS-L-KYIFTQPD-----

-LNLR-QRRWLEL-IK-D---------YDMSIHYH---P-GKA----------NV-----

VADALSR------------------------------------

>Bagy1

--------------------------------------VIYCDT-----SR---------

-----------QGLGC---------------------IL--MQ-------D----R----

--------HVIAYASRQLHPHED-------N---Y-PAHDLELAAVVHAL-------KT-

-HYLL-----------G-NR-----CE----------IFTDHQS-L-KYIFTQPD-----

-LNLR-QRRWVEL-IS-D---------YDLGITYT---P-GAE----------NV-----

MGDALSR------------------------------------

>Tma

--------------------------------------MVYTDA-----SG---------

-----------VGLGC---------------------VL--MQ-------R----G----

--------KVIAYASRQLRKHEG-------N---Y-PTHDLEMAAVIFAL-------KIW

RSYLY-----------G-GN-----VQ----------VFTDHKS-L-KYIFTQPE-----

-LNLR-QRQWMEL-VA-D---------YDLEIAYH---P-GKA----------NV-----

VADALSH------------------------------------

>Legolas

--------------------------------------TVYTDA-----SI---------

-----------VGLGC---------------------VL--MQ-------K----G----

--------SVIAYASRQLRKHEK-------N---Y-PTHDLEMAAVVFFL-------KIW

RSYLY-----------G-AK-----VQ----------IYTDHKS-L-KYIFTQPE-----

-LNLR-QRRWMEL-VA-D---------YNLDIAYH---P-GKA----------NQ-----

VADALSR------------------------------------

>Cereba

--------------------------------------ELECDA-----SR---------

-----------IGLRG---------------------VL--LQ-------D----G----

--------KPVAYFSQKL------------N---Y-STYDKELYALVRT--------ETW

QHYLW-----------P-KE-----FV----------IHYDHES-L-KHIKSQAK-----

-LNRR-HAKWVEF-IE-T---------FPYVIKHK---K-GKE----------NV-----

IVDAFSH------------------------------------

>CRM

--------------------------------------ELECDA-----SG---------

-----------IGLGG---------------------VL--LQ-------E----G----

--------KPVAYFSEKLSGSVL-------N---Y-STYDKELYALVRTL-------ETW

QHYLW-----------P-KE-----FV----------IHSDHES-L-KHIRSQGK-----

-LNRR-HAKWVEF-IE-S---------FPYVIKHK---K-GKE----------NI-----

IADALSR------------------------------------

>Beetle1

--------------------------------------EVECDA-----SG---------

-----------VGIGA---------------------VL--QQ-------G----G----

--------RPIAYFSEKLNHAKL-------N---Y-STYDKEFYAIVRAL-------TYW

THYLR-----------P-AQ-----FV----------LHSDHQA-L-KYINGQHK-----

-LSSR-HAKWVEF-LQ-S---------FDFVLKYK---T-GVS----------NV-----

VADALSR------------------------------------

>REM1

--------------------------------------TVWSDA-----SV---------

-----------HGTGA---------------------VL--LQ--E---------E----

--------RPVAYTSAKFSPAEY-------N---Y-TTTDQECLGTVRAL-------EM-

--EMY-----------PR-G-----GR----------KRDTSHR-PSATCISTRATARTS

FPDAK-HGGWKSC-LD----------------TTV---A-GRLP--------NAL-----

RCHTLVRG---------------------------------SR

>GRhodo

--------------------------------------EVWADA-----SSD--------

----------NTAIGA---------------------VL--MQ-------DHGKGL----

--------QPVAYLSWVLNRQQS-------H---Y-PTFEQELLALFKAF-------EEW

QHYLL-----------PL-H-----FT----------ARTDHNG-L-KFLKTQPRL----

--NER-QFRWMAR-FA-E---------FHFDLHYR---P-GRH----------MA-----

VPDALSR------------------------------------

>Sushiichi

--------------------------------------IVEVDA-----SD---------

-----------AGIGA---------------------VL--SQRS-----EADQKI----

--------HPCAYFSRRFDPAER-------N---Y-DVGNRELLAVYGAL-------VEW

KHWLE-----------GAKHP----FL----------VWSDHKN-L-TYVRTAKRL----

--NPR-QGRWAL--FS-R---------FDFTLTFR---P-GSK----------NI-----

RADALSR------------------------------------

>Amnichi

--------------------------------------VVQADA-----SD---------

-----------VAIGA---------------------VL--LQ-------PVGDHL----

--------HPCAFYSRQLTAPER-------N---Y-TIWEKELLAIKAAF-------ETW

RHWLE-----------GAKFP----IE----------VHTDHRN-L-EHLRTARKL----

--NQR-QQRWALF-FE-R---------FNFQIHYV---T-PAQ----------TK-----

QADALSR------------------------------------

>Amnsan

--------------------------------------FIEVDA-----SD---------

-----------VGAGA---------------------IL--SQRH-----SADGKL----

--------HPCAYFSKKFSSAEQ-------N---Y-DIGNRELLAVKLAL-------EEW

RHLLE-----------GASHP----VT----------IYTDHKN-L-EFLQSLKRQ----

--NPR-QARWSLF-FS-R---------FNFVLTYR---P-GTK----------NR-----

KADALSR------------------------------------

>Amnni

--------------------------------------MVEVDA-----SD---------

-----------VGIGA---------------------VL--SQRG-----E-DNKL----

--------HPCAFLSHRLTPTER-------N---Y-HVGDRELLAVKLAL-------EEW

RHWLE-----------GAKHP----FQ----------VLTDHKN-L-EYVQQAKRL----

--NPR-QARWSLF-FN-R---------FHFTLTYR---P-GSK----------NL-----

KPDALSR------------------------------------

>Dane1

--------------------------------------ILETDA-----SD---------

-----------FVSAG---------------------VL--SQYD-----D-NGVL----

--------HPVAFFSKKHSATEC-------N---Y-EIYDKELLAIIRCF-------EEW

RPELE-----------GTPNP----IK----------VITDHRN-L-EYFMSTKLL----

--NRQ-QARWSEF-LS-C---------FNFRIVYR---P-GKQ----------GA-----

KPDTLTR------------------------------------

>Maggy

--------------------------------------ILETDA-----SD---------

-----------YVSAG---------------------IL--SQYG-----D-DGIL----

--------RPVAFFSKKHTATEC-------N---Y-EIYDKELLAIIRCF-------EEW

RPELE-----------GTSSP----VQ----------IITDHRN-L-EYFTTTKML----

--NRR-QARWAEF-LS-R---------FNFRITYR---P-GKQ----------GA-----

KPDALTR------------------------------------

>marY1

--------------------------------------RVEADS-----SD---------

-----------FAMGA---------------------VL--SQQS-----PEDQKW----

--------HPVAFYSKSLSAVER-------N---Y-EIHDKEMLAIMRAL-------EEW

RHFLE-----------GAQHK----VE----------IWTDHKN-L-EYFMTAKKL----

--NRR-QARWSLY-LS-R---------FDFSLHHR---P-GRS----------MG-----

KTDALSR------------------------------------

>Cgret

--------------------------------------EVEADA-----SD---------

-----------FALGA---------------------QL--SQRD-----S-EGRL----

--------HPCAFFSRKLHGPEL-------N---Y-QIHDKELMAIIEAF-------KEW

RPELS-----------GTIHE----VL----------VYTDHKN-L-AHFTTSKVL----

--NKR-QIRWSEF-LS-E---------FNFRIIYR---K-GSE----------NG-----

RADALSR------------------------------------

>Cft1

--------------------------------------HIETDA-----SD---------

-----------MAIGA---------------------CL--TQ-------THDGKR----

--------HPVAYYSRKMTTAEQ-------N---Y-DIHDKELLAIVAAM-------QHW

RVYVE-----------GPPK-----LT----------ILSDHKN-L-TYFTTTKEL----

--TRR-QARWSEL-LG-Q---------YKFEIKYT---P-GTE----------NG-----

PADALSR------------------------------------

>Pyret

--------------------------------------EVETDA-----SD---------

-----------YTIGG---------------------QL--NQRD-----E-KGRL----

--------HPCAFFSQKLHGPEF-------N---Y-QIYDKKFIAIIRTF-------EKW

KPQLS-----------GTKHE----ML----------IYTDHKN-L-THFTISKIL----

--NKR-QIKWSEF-LS-K---------FHFRIIYR---K-RTE----------NG-----

RADALNR------------------------------------

>Skippy

--------------------------------------ELETDA-----SD---------

-----------FALGG---------------------QI--GQRD-----D-NGVL----

--------HPIAFYSHKMHGAEL-------N---Y-PIYDKEFLAIVNCF-------KEF

RHYLR-----------GSKHP----VK----------VFTDHKN-I-AYFATTQEL----

--NRR-QLRYAEY-LC-E---------FDFTIAHC---K-GTD----------NG-----

RADAISR------------------------------------

>Real

--------------------------------------VLETDS-----SG---------

-----------FAVGG---------------------VL--SQYG-----D-DGVL----

--------RPCAYFSRKNNAHEC-------N---Y-EIHDKELLAVVRCL-------EEW

DSELR-----------SVER-----FK----------VITDHKN-L-EYFMKPRML----

--NER-QIRWSLL-LG-R---------YNMELLYR---P-GKQ----------NV-----

RADALSR------------------------------------

>MGLR3

--------------------------------------RVETDA-----SG---------

-----------GVVAG---------------------AL--LQQN-----PNTQEW----

--------HPIAFFSETMQQAEL-------N---Y-PIHDKELLAVVRAL-------KTW

RPELM-----------GTKKK----FV----------AITDHKA-L-EYFSTKRL-----

L-NSR-QAAWADF-FS-Q---------YNFEITYR---P-GSE----------NV-----

LADALTR------------------------------------

>Grasshopper

--------------------------------------RMETDC-----SG---------

-----------AALGG---------------------CL--SQKGT------DGLW----

--------RPVAFHSAKLTDAQR-------N---Y-TIHDKELLAVIACL-------KAW

DAELR-----------SVRRP----FL----------ILTDHKA-L-EYFSKPREV----

--SER-QMRWAET-LS-K---------FNYNLRFR---P-GRL----------AG-----

VPDALSR------------------------------------

>Pyggy

--------------------------------------ILECDS-----SG---------

-----------YATGG---------------------VL--SQYD-----D-EGVL----

--------RPCAYFSKKNNVHEC-------N---Y-EIHDKELLAVVRCL-------EEW

DAELR-----------SVKS-----FK----------VITDHKN-L-DVLYEAKML----

-TNVK-SV-------G-Q---------AAESIQYGDPVA-GKQ----------NV-----

RADALSR------------------------------------

>Tse3

---------------------------------------LTTDA-----SS---------

-----------TAIGA---------------------VL--ELYGK----GTLKSEL---

-------VGVVAYLSHLLRDNEL-------N---W-PIRDKELYAVIFAF-------KKW

RHYLA-----------GT-H-----II----------IKTDHHS-L-QYFKTSVLD----

-SNLR-LARWRDI-LE-E---------FDYEIQYI---K-GST----------N------

HADALSR------------------------------------

>Ty31

---------------------------------------LTTDA-----SK---------

-----------DGIGA---------------------VL--EEVD-----NKNKLV----

--------GVVGYFSKSLESAQK-------N---Y-PAGELELLGIIKAL-------HHF

RYMLH-----------GK-H-----FT----------LRTDHIS-LLSLQNKNEP-----

--ARR-VQRWLDD-LA-T---------YDFTLEYL---A-GPK----------NV-----

VADAISR------------------------------------

>Skipper

--------------------------------------HLYCDV-----SD---------

-----------KALSG---------------------VL--YQI--------QGNK----

-------FKVIWFHCRKLTDTQK-------R---Y-SIGDREFLSIIDSL-------KKF

QHLLI-----------GK-K-----VS----------IYTDHQN-LTYIINKSND----K

PFTKR-QDNYMKY-IK-E---------FDYELRHI---S-GKK----------NG-----

IADFLSR------------------------------------

>TF2

--------------------------------------LLETDA-----SD---------

-----------VAVGA---------------------VL--SQKH-----D-DDKY----

--------YPVGYYSAKMSKAQL-------N---Y-SVSDKEMLAIIKSL-------KHW

RHYLE-----------STIEP----FK----------ILTDHRN-LIGRITNESEP----

-ENKR-LARWQLF-LQ-D---------FNFEINYR---P-GSA----------NH-----

IADALSR------------------------------------

>TF1

--------------------------------------LLETDA-----SD---------

-----------VAVGA---------------------VL--SQKH-----D-DDKY----

--------YPVGYYSAKMSKAQL-------N---Y-SVSDKEMLAIIKSL-------KHW

RHYLE-----------STIEP----FK----------ILTDHRN-LIGRITNESEP----

-ENKR-LARWQLF-LQ-D---------FNFEINYR---P-GSA----------NH-----

IADALSR------------------------------------

>Tor4a

--------------------------------------VLETDA-----SD---------

-----------KGYGG---------------------VL--YICES----DLSNDSHCHN

SNC----LIPVCYNSGNFSPVQQ-------N---Y-TIVEKELLSGKLCM-------EKW

AIYLA-----------FK-K-----FQ----------WITDNSN-I-KYVRTLRT-----

-NNQK-IARWLTD-LQ-S---------FSFTISQR---P-SSK----------MK-----

ISDFLSR------------------------------------

>Tor2

--------------------------------------ILLSDA-----SE---------

----------LAA-GS---------------------VL--MQNI-------DKRQ----

--------RLIAVSSKTFSETER-------K---W-SATERE-----------------C

YSLLI-----------GCEK-----FEYYLKGPVGFIALVDHKA-LLALDKRY-------

LANSK-LKRWQTR-LA-E---------FKFTVQYV---E-GRS----------HV-----

FADMLSR------------------------------------

>Tor1

--------------------------------------IVTTDA-----SD---------

-----------TGYGG---------------------ML--SQKIG------DDPE----

--------QPLGFTSGFFRGPST-------R---W-AINEKELFAFIKTL-------EVF

HHHTY-----------GR-A-----FT----------WRTDSRC-LAFICAETNG-KQTK

RPSAK-KLRWLEK-LG-E---------YDFSISHV---S-GTSP--------EMA-----

VPDGLSR------------------------------------

>Cer1

--------------------------------------MIYTDA-----SR---------

-----------KGIGA---------------------VL--AQEG-----P-DGQQ----

--------HPIAFASKALSPAET-------R---Y-HITDLEALAMMFAL-------RRF

KTIIY-----------GT-A-----IT----------VFTDHKP-LISLLKGSPL-----

--ADR-LWRWSIE-IL-E---------FDVKIVYL---A-GKA----------NA-----

VADALSR------------------------------------

>Cer2

--------------------------------------HIFTDA-----SA---------

-----------VAQGA---------------------AL--MQTVG----EDEKD-----

-------FAAIAFISRTLADTET-------R---W-PAVHTELGAIIFAL-------RQF

RPYV------------CMSK-----II----------LHSDHKP-LRYILAKSKI-----

--NDQ-IGRWLVE-LQ-Q---------YDISIVHI---D-GKK----------NM-----

VAGCLSR------------------------------------

>Cer3

--------------------------------------HIFTDA-----SA---------

-----------VAQGA---------------------VL--MQQM-----EDSTKD----

-------YAAIAYTSRTLSDTES-------R---W-PAIQTELGAIIFAL-------RQF

RPYI------------GQSR-----TT----------IHSDHRP-PMYLLGKSKV-----

--NDN-LARWLIE-LA-Q---------YDTRIVHI---D-GKK----------NT-----

VADCLSR------------------------------------

>Cigr1

--------------------------------------QVETDA-----SD---------

-----------YAIAA---------------------VL--SQ-------S----G----

--------RPVAYMSRTLNTCER-------N---Y-PAIEKEATAVVEAV-------RKW

SHFLK-----------G-KS-----FT----------LVTDQRS-V-SFMFDQRNRG--K

IKNSK-ILMWRLE-LS-Q---------FTYDIVHR---P-GRD----------NF-----

VPDALSR------------------------------------

>CsRN1

--------------------------------------ILTTDA-----SN---------

-----------AAVGA---------------------VL--HQVV-------NNAS----

--------QPLAFSRRRCRLHKRVTVLLVVN---Y-SQF-----ALLFAI-------SG-

--TCE-----------GR-S-----FT----------IQTDHKP-LTYAFNAKPD-----

RYSPR-EIRHLDY-IS-Q---------FTTDIRYT---P-GSD----------NV-----

VADALSR------------------------------------

>Kabuki

--------------------------------------ALMTDA-----SD---------

-----------TCMGA---------------------VL--QQKV-------NGVW----

--------QPLGYFSRKLSPAEQ-------K---Y-CTYDRELLAIYKAM-------IYF

RKLFE-----------GR-P-----LT----------VYTDTSP-CVMHSVKYVK-----

TIKKRLDARGICFSLG-E---------FTT-VLQS---P-GNP----------RP-----

RASAKTI------------------------------------

>Boudicca

--------------------------------------KPLTDA-----SD---------

-----------SAIGG---------------------VL--QQWV-------NNSW----

--------QPLGFFSRRLLDAES-------R---Y-STFGRELLAMYCAV-------RHF

QHSIE-----------GR-E-----FT----------LFTDHKP-LTFSLSSSSD-----

KYSPR-ESRQLDY-IS-Q---------ITSDIQHI---SGGRD----------RP-----

IVPKHYR------------------------------------

>Osvaldo

--------------------------------------CLQTDA-----SN---------

-----------YGLGA---------------------IL--TQTS-----E-EG-E----

--------RVISYASRTLNSAER-------N---Y-SATEKECLAIIWGI-------RKL

RPYLE-----------G-YH-----FI----------VITDHMA-L-KWLNSIES-----

-PSGR-IARWALE-LQ-Q---------YDFEVRYR---K-GKQ----------NV-----

VADALSR------------------------------------

>Woot

--------------------------------------YVQTDS-----SG---------

-----------YGLGA---------------------EL--YQ----I--QEDGSR----

--------GVIAFASRSLRGPEL-------N---Y-TTTEKELLGVIFAL-------HKF

RIYIQ--------------------VT-------KIIIRTDHQA-L-KFLSRCRLL----

--SER-LTRWTLI-LG-Q---------YDYEIELV---K-GKG----------NV-----

VADILSR------------------------------------

>Ulysses

--------------------------------------FIQCDA-----SH---------

-----------YGVGA---------------------VL--FQLD-----D-EQQE----

--------RPIAFFSAKLNKHQI-------N---Y-SVTEKECLAAKLAI-------HRF

RPYVE-----------MM-P-----FT----------VITDHAS-L-QWLMSLKDL----

--SGR-LARWSLE-LQ-A---------FPFSMQYR---K-GAD----------NV-----

CR-----------------------------------------

>Circe

--------------------------------------LLIISK-----TS---------

-----------YALLL---------------------VS--FTPV-----N-SGCE----

--------RPIAFMSK--NKAQR-------N---Y-TVTELECMAVVLAI-------KKF

RMYID-----------GH-S-----FK----------VVTDHSS-L-RWLMNQSDL----

--SGR-LARWAIK-LQ-G---------YSFEIEHC---K-GTE----------NV-----

VADALSR------------------------------------

>Gmr1

--------------------------------------TLQTDA-----SE---------

-----------VGLGG---------------------VL--SQAR-----N-EREE----

--------HPVTYLSRKLLPHER-------N---Y-STVEKEALAIKWAV-------NKL

TYYLL-----------G-HQ-----FV----------LVTDHAP-L-KWMATAKD-----

-TNAR-ITRWFLS-LQ-P---------FSFTVEHR---P-GRE----------HT-----

NADALSR------------------------------------

>rGmr1

--------------------------------------ILQTDA-----SD---------

-----------SGLGA---------------------VL--SQL------H-EGEE----

--------HPVVYVSRKLTTAES-------K---Y-AAVEKEALAIKWAV-------LEL

KYYLL-----------G-RS-----FT----------LVTDHAP-L-QWMASAKN-----

-TNAR-VTRWFLA-LQ-D---------FHFKVQHR---A-GAA----------HG-----

NADGLSR------------------------------------

>RetroSor1

--------------------------------------LLYVAA-----SE---------

-----------HAVSG---------------------VL--VHETSD---TKGTVQ----

--------RPVYYVSEALSGAKL-------N---Y-TEIEKIAYAVLCAS-------RKL

KHYFQ-----------SH-E-----IK----------VPTSQ-P-LGDILRNKEA-----

--SGR-IGKWAAE-LS-Q---------FDITYVPR--TS-IKS----------QA-----

LADFMAD------------------------------------

>Cinful1

--------------------------------------LLYVAA-----SH---------

-----------SAVSA---------------------AL--VQEKLD---GQTRKQ----

--------VPVYFVSEVLSISKK-------N---Y-TELEKVLYVVLMAS-------RKL

RHYFQ-----------AY-N-----II----------VPSSQ-P-LKDIMRNREA-----

--TGR-IGKWAAE-LN-E---------FCIEYVHR--SS-IQS----------QA-----

LADFIAD------------------------------------

>B1147A04.5

------------------------------------------------------------

------------VVGTE----DECTPRGRLDKEHPRDVA--PSEEDR---PHRKVQ----

--------RPVYIVSEALRDAKT-------R---Y-PQAQKMLYAILMAS-------RKL

RHYFQ-----------AH-R-----VT----------VVTSY-P-LGQILHNREG-----

--TGR-VVKWAIE-LS-E---------FDLHFEPR--HA-IKS----------QA-----

LADFVAE------------------------------------

>Ogre

--------------------------------------IMYLSV-----TE---------

-----------NSMGC---------------------VL--GRHDES---G--RKE----

--------HAIYYLSKKFTDCET-------R---Y-SLLEKTCCALAWAA-------RRL

RQYML-----------NH-T-----TL----------LISKMDP-VKYIFEKPAL-----

--TGR-VARWQMI-LT-E---------YDIQYTSQ--KA-IKG----------SI-----

LSDYLAE------------------------------------

>Grande14

--------------------------------------LLHDSR-----GK---------

-----------HHVGSRASRRRTCLPSA-------TSCL--LHQSPG---P---------

--------------------SKK-------K---Y-PQVQKLLYAVLLTA-------RKL

RHIFD-----------DH-K-----VI----------VVTGF-P-IGDILHNKEA-----

--MGP-IAKWACE-LG-S---------HDIEFRPR--TV-IKT----------QA-----

HGFRIR-------------------------------------

>Tat41

--------------------------------------YLYIAV-----SS---------

-----------SAVSG---------------------VI--VRED-------RGEQ----

--------KPIFYVSKTLEGAEL-------K---Y-PTLEKIAYAVLISA-------RKL

RPYFQ-----------FH-S-----II----------VQTNQ-P-LRQILHSPGQ-----

--SGR-MAKWAVE-LS-E---------YDIEYQNR--TS-MKS----------QV-----

LADFIVE------------------------------------

>Tft2

--------------------------------------YLYVSV-----SD---------

-----------HAVSG---------------------VL--IRED-------RGEQ----

--------KPIYYISKSMTDPEI-------R---Y-TMMENLALAIVTSA-------RKL

RPYFQ-----------SH-P-----IE----------VLSNQ-P-HRTILHSPNQ-----

--SGR-LAKWAVE-LS-E---------YDIEYKSR--VA-MKA----------QV-----

LADFLTE------------------------------------

>RIRE2

--------------------------------------LLYVSA-----TS---------

-----------QVVST-------------------VLVV--EREEEG---HVQKVQ----

--------RPIYFVSEVLADSKT-------R---Y-PQVQKLLYGILITT-------RKL

SHYFQ-----------GH-S-----VT----------VVTSF-P-LGDILHNREA-----

--NGR-IAKWALE-LM-S---------LDISFKPR--IS-IKS----------QA-----

LADFVAE------------------------------------

>Mag

--------------------------------------VLTVDA-----SA---------

-----------RGLGA---------------------VL--AQRG-----P-GCQE----

--------RVVAYASRALTTHEL-------H---Y-SQIHKEALAIVFAV-------EKF

HQYLY-----------GR-K-----FI----------LRTDHKP-LVSIFGPNIGIP--S

AAASR-LQRWAIK-LS-A---------YDFEIEYV---R-TDK----------N------

VADALSR------------------------------------

>Gulliver

--------------------------------------AVAADT-----ST---------

-----------FGIGA---------------------VI--LHRF-----S-NESQ----

--------KAIAHASRTL-----FV----AK---Y-SQIKKEALALVFAV-------KMF

HKMLH-----------GR-K-----FT----------TQTDHKP-LLAIFGSGSAI---P

VHSAICLQRWATMLLG------------DFVLQYN---TVSAS----------MG-----

HADILSW------------------------------------

>DRM

--------------------------------------TLACDS-----SA---------

-----------YGIGA---------------------VL--QHTM-----P-TGEE----

--------RPIAYASRTLSPAEK-------K---Y-SQIEKEGLSLIFGI-------KKF

HQYLW-----------GR-K-----FK----------MVTDHKP-LLTLFGEHKSLP--T

MAAAR-IQRWAII-LS-A---------YDYHIEYC---P-SEK----------NS-----

NADGLSR------------------------------------

>CFG1

--------------------------------------VLSCDA-----SP---------

-----------YGLGA---------------------LL--SHVM-----L-DGRE----

--------RPIAYASRTLAPAKK-------H---Y-SQLDKEGAALIFGV-------RKF

HQYLF-----------GQ-K-----FV----------VYTDHKP-LLGLFKADRAIP--S

MASAR-IQRWALL-LA-T---------YEFDLRYR---P-GTK----------NS-----

NADGLSR------------------------------------

>Hydra21

--------------------------------------ILSCDA-----SP---------

-----------YGIGA---------------------VL--AHVT-----S-EGSE----

--------RPITYISRTLSAAER-------N---Y-SQIEKEALSIVFAV-------KKL

HQYLY-----------GR-Y-----FT----------LVTDHKP-LLGLLAEGKPIP--A

MTAAR-IQRWALT-LS-A---------YNYCLKYR---S-GAT----------HG-----

NADCMSR------------------------------------

>SPM

--------------------------------------VLACDA-----SP---------

-----------YGLGV---------------------VL--SHLM-----E-DGEE----

--------RPVAYASRTLTKSEQ-------N---Y-SQIEKESLAIIYGV-------TKF

HKYLY-----------GR-K-----FT----------LLTDHQA-LTIIFGSKKGIP--S

LAAAR-LQRWALI-LM-A---------HQYEIKYR---K-STE----------HA-----

NADVLSR------------------------------------

>SURL

--------------------------------------KLSADA-----SK---------

-----------NGIGA---------------------VL--LQ-------QHDENW----

--------VPIAYASRSMTDAET-------R---Y-AQIEKELLAITYAC-------ERF

HQYIY-----------GQ-Q-----VE----------VETDHKP-LIPLFVKSLGD----

-CPLR-IQRLLIR-VQ-R---------YDLKVMYT---P-GKY----------MY-----

TADTLSR------------------------------------

>Cer4

--------------------------------------IVAADA-----SK---------

-----------YGIGG---------------------VI--LHVN-----P-DGVE----

--------VPIAHFARSLTETEK-------R---Y-SQIEKEALALIYTV-------KKS

HKFVF-----------GR-R-----FK----------LQTDHRP-LLALFGDNRDLP--V

HSQNR-IVRWATT-LM-S---------YDFELSYV---A-TEK----------FA-----

KADWLSR------------------------------------

>Cer5

--------------------------------------IIAADA-----SQ---------

-----------YGIGG---------------------VL--LHQT-----P-EGHE----

--------VPVAHFARALTDTEK-------R---Y-SQIEKEAVALVYTV-------KKA

HKFIF-----------GR-K-----FL----------LQTDHKP-LLAIFGPKKDLP--V

HSQNR-LVRWATT-LM-T---------YNFELSYV---S-TSK----------IF-----

KADWLSR------------------------------------

>Cer6

--------------------------------------IVAADA-----SN---------

-----------YGIGG---------------------VL--CHVN-----P-DGVE----

--------VPIAHYARSLTATEQ-------K---Y-SQIEKEGLALIFTI-------KKA

HKFIF-----------GR-K-----FK----------LQTDHQP-LLAIFGSKKDLP--V

HSQNR-LVRWATT-LM-S---------YDFDLS-L---S-TSK----------FA-----

KADWLSR------------------------------------

>412

--------------------------------------CITTDA-----SK---------

-----------QACGA---------------------VL--TQ-------NHNGHQ----

--------LPVAYASRAFTKGES-------N---K-STTEQELAAIHWAI-------IHF

RPYIY-----------G-KH-----FT----------VKTDHRP-L-TYLFSMVN-----

-PSSK-LTRIRLE-LE-E---------YNFTVEYL---K-GKD----------NH-----

VADALSR------------------------------------

>Mdg1

--------------------------------------CITTDA-----SK---------

-----------QACGA---------------------VL--SQ-------DHNGQQ----

--------LPVAYASRSFTKGES-------N---K-STTEQELAAIHWAI-------NHF

RPYVY-----------G-RH-----FL----------VQSDHRP-L-SYLFSMRN-----

-PSSK-LTRMRLD-LE-E---------FEFTVEYL---K-GKD----------NH-----

VADALFR------------------------------------

>HMSBeagle

--------------------------------------HLTTDA-----SN---------

-----------FAVGA---------------------VL--SQ-------E----N----

--------RPISFLSRTLSKAEE-------N---Y-ATNEKEMLAIIWAL-------KKL

KIYLY-----------GKAK-----VK----------IFTDHQP-L-THSLSSWN-----

-GNAR-IKRWKAY-LE-E---------YDYEIFYK---P-GRE----------NT-----

VADALSR------------------------------------

>Yoyo

--------------------------------------ELTTDA-----SN---------

-----------YAIGA---------------------VL--SQ-------E----D----

--------RPITFISRTLTKTEE-------N---Y-AANEKEMLAIIWAL-------KSL

RNYLY-----------GSAK-----VK----------IFTDHQP-L-TYALSNKN-----

-NNSK-MKRWKAI-LE-E---------YNYELKYK---P-GKT----------NV-----

VADGLSR------------------------------------

>Gypsyvir

--------------------------------------DLTTDA-----SA---------

-----------SGIGA---------------------VL--SQ-------G----N----

--------RPITMISRALKQAEQ-------N---Y-ATNERELLAIVWAL-------GRL

QNFLY-----------GSRE-----IN----------IFTDHQP-L-TFAVSDKN-----

-TNSK-IKRWKSY-ID-Q---------HNAKMFYK---P-GKE----------NL-----

VADALSR------------------------------------

>Burdock

--------------------------------------DLTTDA-----SA---------

-----------FGLGA---------------------VL--SQ-------D----G----

--------KPVTMISRTLQDREL-------N---F-ATNERELLAIVWAL-------KSL

RNYLY-----------GVKN-----LN----------IFTDHQP-L-TYAVSDRN-----

-PNAK-IKRWKAF-ID-E---------HNAKIFYK---P-GKE----------TY-----

VADALSR------------------------------------

>Nomad

--------------------------------------HLTTDA-----SN---------

-----------WAIGA---------------------VL--SQ-------DDQGRD----

--------RPIAYISRSLNKTEE-------N---Y-ATIEKEMLAIIWSL-------DNL

RAYLY-----------GAGT-----IK----------VYTDHQP-L-TFALGNRN-----

-FNAK-LKRWKAR-IE-E---------YNCELIYK---P-GKS----------NV-----

VADALSR------------------------------------

>Gypsy

--------------------------------------DLTTDA-----SA---------

-----------SGIGA---------------------VL--SQ-------E----G----

--------RPITMISRTLKQPEQ-------N---Y-ATNERELLAIVWAL-------GKL

QNFLY-----------GSRE-----IN----------IFTDHQP-L-TFAVADRN-----

-TNAK-IKRWKSY-ID-Q---------HNAKVFYK---P-GKE----------NF-----

VADALSR------------------------------------

>297

--------------------------------------VLTTDA-----SN---------

-----------LALGA---------------------VL--SQ-------N----G----

--------HPISFISRTLNDHEL-------N---Y-SAIEKELLAIVWAT-------KTF

RHYLL-----------G-RQ-----FL----------IASDHQP-L-RWLHNLKE-----

-PGAK-LERWRVR-LS-E---------YQFKIDYI---K-GKE----------NS-----

VADALSR------------------------------------

>17.6

--------------------------------------TLTTDA-----SD---------

-----------VALGA---------------------VL--SQ-------D----G----

--------HPLSYISRTLNEHEI-------N---Y-STIEKELLAIVWAT-------KTF

RHYLL-----------G-RH-----FE----------ISSDHQP-L-SWLYRMKD-----

-PNSK-LTRWRVK-LS-E---------FDFDIKYI---K-GKE----------NC-----

VADALSR------------------------------------

>Tv1

--------------------------------------SLTTDA-----SN---------

-----------MAIGA---------------------VL--SQ-------E----H----

--------KPICYASRTLNEHEL-------N---Y-STIEKELLAIVWAT-------KYF

RSYLF-----------G-RQ-----FQ----------ILSDHRP-L-VWLNNMKE-----

-PNMK-LQRWKIK-LN-E---------FDFQIKYV---P-GKE----------NY-----

VADALSR------------------------------------

>Ted

--------------------------------------NLTTDA-----SN---------

-----------FAIGA---------------------VL--SQGPIG---S----D----

--------KPVCYASRTLNESEL-------N---Y-STIEKELLAIVWAT-------KYF

RPYLF-----------G-RK-----FK----------ILTDHKP-L-QWMMNLKD-----

-PNSR-MTRWRLR-LS-E---------YDFSVVYK---K-GKS----------NT-----

NADALSR------------------------------------

>Zam

--------------------------------------ILTTDA-----SN---------

-----------FALGA---------------------VL--SQGSLQ---N----D----

--------RPVCFASRTLSDTEV-------N---Y-STIEKEMLAIIWAV-------KYF

RPYIY-----------G-VK-----FT----------IVTDHKP-L-IWLMNFKE-----

-PNSK-IIRWRLQ-LM-E---------YNFEIIHK---K-GSQ----------NV-----

IADALSR------------------------------------

>Idefix

--------------------------------------SLTTDA-----SN---------

-----------VAIGA---------------------VL--SQ-------N----H----

--------KPVCYASRTLNEHEI-------N---Y-ATIEKELLAIVWAT-------KYF

RSYLF-----------G-RP-----FE----------VLSDHKP-L-VWLNNIKE-----

-PNMK-LQRWKIK-LN-E---------FDYKIKYL---P-GKE----------NH-----

VADALSR------------------------------------

>Tom

--------------------------------------VLTTDA-----SN---------

-----------LALGA---------------------VL--SQ-------D----N----

--------HPISFISRTLNDHEL-------N---Y-STIEKELLAIVWAT-------KTF

RHYLL-----------G-RH-----FQ----------IASDHQP-L-RWLHNLKE-----

-PNAK-LQRWRIR-LA-E---------FDFHIEYI---K-GKQ----------NS-----

IADALSR------------------------------------

>Springer

--------------------------------------DLTTDA-----SA---------

-----------YGIGA---------------------VL--SQ-------E----G----

--------RPITMISRTLSDREV-------N---Y-ATNERELLAIVWAL-------AKL

RHYLY-----------AVKE-----IN----------IFTDHQP-L-TFAVSESN-----

-PNAK-IKRWKAR-ID-E---------SGARIFYK---P-GRN----------NL-----

VADALSR------------------------------------

>Blastopia

--------------------------------------ELHTDA-----SK---------

-----------DGLGA---------------------VL--LQKF-----E--GSF----

--------HPVCFWSRKTTKAES-------N---R-HSYYLEVKAAYLAL-------KKF

RHYLL-----------GV-P-----FK----------LVTDCVA-F-KQTTKKADV----

--PRE-VGPWILY-MQ-D---------FNFQPEHR---A-GER----------MR-----

HVDFLSR------------------------------------

>Micropia

--------------------------------------ELHTDA-----SA---------

-----------CGYGA---------------------IL--LHRI-----E--SKP----

--------HVIEYFSKTTTSVES-------R---Y-HSYELETLAVVKAV-------KHF

RHYLI-----------GR-E-----FV----------VYTDCNS-L-KASRTKIDL----

--TPR-VHRWWAY-LQ-S---------FNFEIQYR---E-GKR----------MA-----

HVDFLSR------------------------------------

>Mdg3

--------------------------------------ELHCDA-----SS---------

-----------SGFGA---------------------VL--MQKK-----E-DQKW----

--------HPVSFFSKRTTDIES-------K---Y-HSFELETLAIVYSL-------RRF

RVYLH-----------WR-T-----FK----------IVTDCNS-L-ILTLSKKEL----

--NPR-IARWALE-FQ-G---------YDFEIVHR---A-GSR----------MQ-----

HVDALSR------------------------------------

>Moose

--------------------------------------HCFTDA-----S----------

----------ELAYGA-------------C-------IY--IRS-----EAEDGSIHVNL

----------LASKSRVAPLKAL-------------TIPRLELCGALLGARLH----EKV

MAAME------------IK--FVA-HR----------FWTDSTV-VLDWLNA-ESK----

TWK-TFVANRVAE-IQ-AI-R-------DAVWQHV---S-GQE----------NP-----

-ADLISR------------------------------------

>Max

--------------------------------------HGFCDA-----S----------

----------QRAYGA-------------A-------IY--IRV------EMGQKILTRL

----------LTAKTRVAPVKTV-------------SLPRLELCGAVLLTEMV----TAI

LPHMP------------SA--SSD-IR----------CWTDSTI-VLAWLRK-PAC----

NWT-TFVANRVAK-IT-QATP-------VDCWAHV---R-SEQ----------NS-----

-ADLASR------------------------------------

>Tribel

--------------------------------------HGFSDA-----S----------

----------ERAYGA-------------V-------MY--IRTR----DPSQDNWSTKL

----------LCAKSKVAPLHNL-------------SLPRLELCGALILARLF----DKV

ISSMD------------VN--FEG-HY----------LWCDSTI-VLNWLNS-PPS----

RWK-TFIANRVSE-IQ-NLTQ-------NAKWQLV---P-SND----------NP-----

-ADLLSR------------------------------------

>Nabel

--------------------------------------HGFSDA-----S----------

----------ETGYGA-------------C-------IY--MRS-----KDTFGNYKITL

----------ICSKSRVAPVKTR-------------SLPRLELCGAQLLANLY----VHT

IRSIR------------IQ--VDR-TY----------FWCDSSI-TLHWINT-APP----

TLK-TFVANRVVD-IQ-LKTE-------IHAWRHI---R-SED----------NP-----

-ADALSK------------------------------------

>Roo

--------------------------------------HGFADA-----S----------

----------EKAYAA-------------V-------VY--AKV----------GPHVNI

----------IASKSRVNPIKNR-------K-----TIPKLELCAAHLLSELI----QRL

KGSID-------------N--IME-IY----------AWSDSTI-TLAWINS-GQS----

KIK--FIKRRTDD-IR-KL-K-------NTEWNHV---K-SED----------NP-----

-ADLASR------------------------------------

>Bel

--------------------------------------HGFADA-----S----------

----------SHAYGA-------------V-------VY--ARV------AVGCSFQVTL

----------VAAKTRVAPIKPV-------------SIPRLELNAALLLSRLL----SIV

KTSLT------------IP--LFS-TS----------CWTDSEI-VLHWLSA-PPR----

RWN-TYVCNRTSE-IL-SDFP-------RSCWNHV---R-TED----------NP-----

-ADCASR------------------------------------

>Cer7

--------------------------------------IAFADA-----S----------

----------QEAIAA-------------C-------IY--VKNKY--------------

------GVNLLFGKSNVKSLKEK-----------W-TIPKLEVQALKVATDRA----LST

LTALQDG---------DIK--VTK-VI----------LFSDSEI-TLAWLRSEPGK----

KEVGILIKNRIES-IR-KTNETMLQKGVQVFFGYV---N-TLE----------NP-----

-ADLRTR------------------------------------

>Mabel

--------------------------------------HVFTDA-----S----------

----------TAAYSA-------------A-------LY--IRT---------QEREVFL

----------VFAKSRIAPIKGM-------------SIPRLELLAILIGVRMV----QFV

LKQKE------------LE--DVI-TI----------LWSDSQC-ALHWVHN-SSR----

LLP-TFIQNRVEE-IR-KA---------KIHFRYI---P-SEQ----------NP-----

-ADIATK------------------------------------

>Spirobel

--------------------------------------HAFGDA-----S----------

----------ETAYGA-------------V-------VY--IVV-----KKEDYSSISNI

----------VMAKSRVAPLKKM-------------TLPRLELMAAQMAAKLM----TFV

KEALK------------IR--IDR-LT----------CWTDSKI-TLYWIKS-ISK----

RWK-PFIQNRVEN-IQ-QLVE-------PSQWRHC---P-TNS----------NP-----

-ADILSR------------------------------------

>Tas

--------------------------------------HCFTDA-----S----------

----------KTAYAS-------------A-------IY--IRT------EMNSEIECHL

----------LICKTRLEPLKGS-------------TIPHLELMGALIGTRLL----EYT

SQQLQ------------LE--SAD-KY----------IWTDSQC-TLQRIRS-SDV----

HQKDRFVENRLKE-IR-KT---------DARFENG---S-SLQ----------IPIRPIF

VREVVTS------------------------------------

>Hydra31

--------------------------------------HGFSDA-----S----------

----------VKSFGC-------------C-------VY--LRF------FNANFSRASL

----------IASKSRVAPLGKN-------------TMPRLELSATLLLAKLL----ASI

YDQLIS----------IYN--ISN-IV----------YWTDSTN-CLHWIFN-TNN----

TYE-QFVQNRLNK-IR-ELTL-------ICNWNYI---E-SFR----------NP-----

-ADIISR------------------------------------

>Cer101

--------------------------------------IMFSDA-----S----------

----------TDIYAT-------------A-------VY--VQY-----SYKSRKPVTRL

----------LTSKSKIRSAKNE-------M---W-TIPKLELLGIECSSNLA----RTI

IAELGTL---------ATK--IKK-VR----------LFTDRAC-ALYWILS-HKT----

TR--VWVANRVSQ-IQ-ANQKILEECGIETSLHHC---P-TKE----------NP-----

-ADLATR------------------------------------

>Ninja

--------------------------------------HVFVDA-----S----------

----------QAAFAA-------------V-------AY--WRV-----TYEDDDVQVSF

----------VSAKTKCAPMRTM-------------TIPRLELQAAVLGTRLM----NTV

KQEHS------------VV--ITD-LL----------LWTDSKT-VLRWIGS-THR----

RYK-QFVGNRVAE-IL-ESSK-------VSQWRWV---P-TAD----------NA-----

-ADDATR------------------------------------

>Tamy

--------------------------------------HTFVDA-----S----------

----------ETAYAA-------------A-------CY--WRA-------ETEVIHVAL

----------IAGKARVSPAKPV-------------TIPRLELQAALLGARLA----RTV

TEEID------------LQ--VTD-RY----------FWSDSST-VLQWLKA-DPR----

RFK-PFVAHRLAE-IK-DLSK-------PHEWRWV---P-TRD----------NP-----

-ADIATR------------------------------------

>Cubel

--------------------------------------HTFVDA-----S----------

----------TNGCAT-------------V-------AF--LRF------EEEDRIECAF

----------VAGKTRVAPNKLT-------------SIPRLEIDAGTMGVRLA----QKI

MEGLR------------IV--IHQ-RF----------FWTDARD-VLCWLHA-DHR----

NYS-QFVGFRVAE-IL-EKSN-------LAEWNYC---P-SKL----------NP-----

-ADDGTK------------------------------------

>Zebel

--------------------------------------HIFCDA-----S----------

----------ERAYGS-------------V-------AY--LRA-----SDDQGHVAVTF

----------VLARSRVAPRKCL-------------SMPRLELSAALTGAQLA----QVI

QNELT------------LP--IDS-VT----------LWSDSTT-VLYWLTA-ESC----

RYK-VFVGGRVAE-IQ-TLTE-------TAEWRYV---D-SAN----------NP-----

-ADHITR------------------------------------

>Sinbad

------------------------------------------------------------

------------------------------------------------------------

------------------------------------------------------------

------------------------------------------------------------

------------------LTK-------VDQWRFV---P-SKE----------NI-----

-ADFASR------------------------------------

>Saci6

------------------------------------------------------------

------------------------------------------------------------

------------------------------------------------------------

--ELD------------IK--FAE-VK----------FWTDSTI-VLHYIRN-EKS----

QFK-TFIANRIST-IH-SLTK-------VDQWRFV---P-SKE----------NI-----

-ADFASR------------------------------------

>Kobel

--------------------------------------HAFADA-----S----------

----------EIGYGC-------------V-------IY--LRQ-----INSSENIHCSF

----------VFAKARVTPLKKI-------------TIPRLELTAATLAVRLV----SIV

QRELD------------FK--IDK-AI----------YWTDSTA-VLRYIRN-DRA----

RYH-TFVANRVQV-IR-EATV-------PEQWHHV---D-TKR----------NP-----

-ADLASR------------------------------------

>Purbel

--------------------------------------CIFCDA-----S----------

----------EIAYGA-------------C-------AY--LRW-----KTDDDKYEVRF

----------VSAKSKVAPLKAL-------------TIPRLELLAGVLAARMH----EAI

SNEMR------------LQ--VEK-VV----------FFTDSMI-VLQWIKS-SAR----

TYK-AFVSSRVGE-IQ-TLTN-------PADWKHI---P-GEV----------NI-----

-ADKVSR------------------------------------

>Suzu

--------------------------------------LTFSDG-----S----------

----------EHSYGA-------------V-------MY--LRW------ETDESPIIRL

----------VEAKAKLSPLDQK-------G-----EAVKAEVCGAVIASRLK----KYF

QSHSP------------IQ--VDR-WF----------HFVDSQT-VLGAIQR-ECY----

GFQ-TFFSNRIGE-IQ-TNSR-------AEDWWWV---P-GPQ----------NV-----

-ADMITR------------------------------------

>Gabel

--------------------------------------ITFSDG-----S----------

----------EHAYGA-------------V-------LY--LRW------ACNQGSMVRL

----------VESKAKLTPLDHK-------G-----EAVKSELCGAVFAAWLK----KYF

EQHGR------------IQ--VKQ-WY----------HFVDSQT-VLGAIQR-ESY----

GFQ-TFFANRIGE-IQ-GSTQ-------RQDWWWI---P-GTL----------NI-----

-ADIITR------------------------------------

>Retrofit

--------------------------------------HGYSDA-----DWAGSID----

--------DRKSTGGF-------------A-------VF--L------------------

------GSNLVSWSARKQPTVSR-------------SSTEAEYKAVANTTAEL----IWV

QTLLKEL---------GIE-SPKA-AK----------IWCDNLG-AKYLSAN-PVF----

HARTKHIEVDYHF-VR-ERVSQ-----KLLEIDFV---P-SGD----------QV-----

-ADGFTK------------------------------------

>Koala

--------------------------------------SGYSDA-----DWAGNGD----

--------DRRSTGGF-------------A-------VF--L------------------

------GNSLVSWSARKQPTVSR-------------SSTESEYKAMANATAEI----MWI

QTLLKEI---------HVS-NSPT-AR----------LWCDNLS-AKYLSSN-PIF----

HARTKHIEVDYHF-VR-ERVQQ-----KLLEVGFV---P-TGD----------QI-----

-ADGFTK------------------------------------

>Hopscotch

--------------------------------------LGPNDSTVPQHSWSIAVF----

--------DIDKT-GY-------------I-------IFSKQ------------------

------GPNLVSWSARKQATVSR-------------SSTEAEYKALANATAEI----MWI

QTLLQEL---------GVK-GPRA-AK----------VWCDNI--AKYLTAN-PVF----

HARTKHIEVDIHF-VR-ERVAH-----KLLEV----------------------------

-------------------------------------------

>Melmoth

--------------------------------------KGFADS-----DWASCQD----

--------SRRSTTSF-------------T-------MF--V------------------

------GDSLISWRSKKQHTVSR-------------SSAEAEYRALALATCEM----VWL

FTLLVSL----------QA-SPPV-PI----------LYSDSTA-AIYIATN-PVF----

HERTKHIKLDCHT-VR-ERLDN-----GELKLLHV---R-TED----------QV-----

-ADILTK------------------------------------

>Vitico12

--------------------------------------VGYCDA-----DYTGDHD----

--------TRRSTTGY-------------V-------FM--L------------------

------GSRAISWCSKRQPTVSL-------------STTEAEYRAAAMATQES----TWL

IXLMNDL---------HQL-VDYA-VP----------LYCDNQL-AVHLAEN-PVF----

HARTKHVEVHYHF-IR-EKVLE-----EEVELK-I---K-SGD----------QV-----

-ADLFTK------------------------------------

>Oryco11

--------------------------------------IGYTDS-----DWAGCLD----

--------DMKGTSGY-------------A-------FS--L------------------

------GSGMCSWSTKKQNIVAL-------------SSAEAEYVAASKAVSQV----VWL

RRIMEDL---------GEK-QYQP-TT----------IYCDSKS-AIAISEN-PVS----

HDRTKHIAIKYHY-IR-EAVDR-----QEVKLEFC---R-TDE----------QL-----

-ADIFTK------------------------------------

>Poco

------------------------------------------DG-ADKVDEA--------

--------IYRSLIGC-------------L-------MY--LTATR--------------

----PDIMHAVSLLSKKQEIIAQ-------------STAEAEYVAATAAANQV----LWL

RKILADL---------NME-QKKA-TR----------VNVDNQA-AIAISNN-PIF----

HGKTKHFKLKYYF-LR-EVQKN-----EEIQLIYC---R-TED----------QL-----

-ADILTK------------------------------------

>Vitico11

AGTYDFGIWYGHVQEFK--------------------LVGYTDS-----DWAGCLE----

--------DRKNTSSY-------------M-------FS--LGS----------------

---------------------------------------------------------VWL

RRILADI---------SQE-HEES-TI----------IYCDNKA-AIAMTKN-PAY----

HGRTKHVDIRVHF-IR-DLVVE-----GKVVLQYC---N-TNE----------QV-----

-ADVLTK------------------------------------

>Araco

--------------------------------------VGYSDS-----DWGGDVD----

--------DRKSTSGF-------------V-------FY--I------------------

------GDTAFTWMSKKQPIVTL-------------STCEAEYVAATSCVCHA----IWL

RNLLKEL---------SLP-QEEP-TK----------IFVDNKS-AIALAKN-PVF----

HDRSKHIDTRYHY-IR-ECVSK-----KDVQLEYV---K-THD----------QV-----

-ADIFTK------------------------------------

>Oryco12

--------------------------------------VVYSDS-----DLAGDLD----

--------EQKSTSGQ-------------I-------FF--V------------------

------NGGPVTWQSSKQKVVAL-------------SSCEAEYIAVAAATCQS----VWL

AWLLAEV---------MGD-EVAA-PL----------LKVDNQS-TISLIKN-PVH----

HDRSKHIDVKYHY-IR-ECAEK-----KLIEVMSV---G-TAE----------QL-----

-GDIFTK------------------------------------

>Endovir11

--------------------------------------VGYCDA-----DWGGNLD----

--------DRRSTTGG-------------V-------FF--L------------------

------GSNLISWHSKKQNCVSL-------------SSTQSEYIALGSCCTQL----LWM

RQMGLDY---------GMT-FPDP-LL----------VKCDNES-AIAISKN-PVQ----

HSVTKHIAIRHHF-VR-ELVEE-----KQITVEHV---P-TEI----------QL-----

-VDIFTK------------------------------------

>SIRE14

--------------------------------------VGYCDA-----DWAGSAD----

--------DRKSTSGG-------------C-------FY--L------------------

------GNNLISWFSKKQNCVSL-------------STAEAEYIAAGSSCSQL----VWM

KQMLKEY---------NVE--QDV-MT----------LYCDNMS-AINISKN-PVQ----

HSRTKHIDIRHHY-IR-DLVDD-----KVITLKHV---D-TEE----------QI-----

-ADIFTK------------------------------------

>Opie2

--------------------------------------VGYSDS-----DYAGCKV----

--------DRKSTSGS-------------C-------QM--L------------------

------GRSLVSWSSKKQNFVAL-------------FIAEAEYVSAGSCCAQL----LWM

KQILLDY---------GIS--FTK-TP----------LLCENDS-AIKIANN-PVQ----

HSRTKHIDIRHHF-LR-DHVAK-----CDIVISHI---R-TED----------QL-----

-ADIFTK------------------------------------

>TSI9

--------------------------------------CGYSDA-----DFAGCRL----

--------DRKSTSGT-------------C-------QF--L------------------

------GSSLVSWSSRKQSNVAQ-------------STTEAEYVAAASCCSQL----LWM

IATLRDF---------GLS--FSR-VP----------LLCDSTS-AISVAKN-PVL----

HSKTKHIEVRHHF-LR-DHVEK-----GDIELKYI---D-TSQ----------QL-----

-ADILTK------------------------------------

>ToRTL1

--------------------------------------VGFADA-----DFAGYQV----

--------DRKSTSGM-------------A-------HF--L------------------

------GSSLISWGTKKQNSVAL-------------STAEAEYVAAAACCSQL----LWI

RQHLEDF---------GIH--IKA-FL----------LMCDNTS-AVSMGKN-SFH----

HKRTKHIDVRHHF-LR-DHVEK-----GNIVLTYC---P-TEE----------QI-----

-ADIFTK------------------------------------

>Fourf

--------------------------------------EGYCDA-----NWISDAD----

--------ELYATSGY-------------V-------FL--F------------------

------GGGAVSWKSCKQTILTK-------------STMEAELAALDTAGAEA----EWL

RDFLLDLP--------VVEKPIPA-IS----------MNCDNQT-VITKVNS-SNN----

MKSTRHVKRRLKS-VR-KLKNS-----GVITVDYV---H-TSN----------NL-----

-ADQFTK------------------------------------

>Batata

--------------------------------------TGYTDS-----DMAGDID----

--------TRKSTSGY-------------L-------IT--Y------------------

------AGGAVSWQSRLQKCVDL-------------STTEAEFIASVEASKEM----LWM

KKFLQEL---------GFV--QD-------------------------------------

--RSKHIDTRYHW-IR-DILEC-----KMLELEKI---H-TDD----------NG-----

-SDMMTKALPRGKFEEAMGECCNGFLRTVILEMEEVVEDDLSK

>Sto4

--------------------------------------VGYADA-----DWGGCRD----

--------TLKSTSGY-------------V-------FM--L------------------

------SGGAISWKSCKQTARAS-------------STMHAEFVATYEATGQA----IWI

KKFVPGLR--------VVDSIERP-LR----------IYCDNEP-AVFFSHN-NKS----

SGSAKYIDIKCYI-VK-EKILD-----HTIQVEHI---R-THQ----------ML-----

-ADPLTK------------------------------------

>Tork4

--------------------------------------TGYSDS-----DYAGDVD----

--------TRRSMTGY-------------V-------FT--L------------------

------GGSVVSWKATLQPTVTL-------------STTEAEYMALTEAAKEG----IWL

KGLVSDL---------GLH--HDQ-AT----------VYCDSLS-AICLAKD-QVH----

HERTKHIDVRYHF-LR----SE-----KRIKVKKV---G-TAD----------NP-----

-ADMFTK------------------------------------

>Tto1

--------------------------------------VGYTDA-----DMAGDVD----

--------SRKSTSGY-------------L-------IN--F------------------

------SGGAVSWQSKLQKCVAL-------------STTEAEFIAATEACKEL----IWM

KKFLTEL---------GFS--QDG-YQ----------LFCDSQS-AIHLAKN-ASF----

HSRSKHIDVRYNW-IR-DVLEK-----KMLRLEKI---H-TDE----------NG-----

-SDMLTK------------------------------------

>RTvr2

--------------------------------------VGYSDS-----NMAGDID----

--------SRKSTSGY-------------L-------IQ--F------------------

------AGGALAWQSKLQKCVAL-------------STTEAEFIALTEAFKEL----LWV

KKFL-EL---------GFV--QDK-YL----------LYCDSQS-IIHFGKN-PTF----

NSKSKHIDVRYHW-IR-DVLDA-----KLGELAKV---H-TDD----------NG-----

-ADMMTK------------------------------------

>Tnt1

--------------------------------------KGYTDA-----DMAGDID----

--------NRKSSTGY-------------L-------FT--F------------------

------SGGAISWQSKLQKCVAL-------------STTEAEYIAATETGKEM----IWL

KRFLQEL---------GLH--QKE-YV----------VYCDSQS-AIDLSKN-SMY----

HARTKHIDVRYHW-IR-EMVDD-----ESLKVLKI---S-TNE----------NP-----

-ADMLTK------------------------------------

>V12

--------------------------------------VGCTDA-----DMVGDVD----

--------SRKSTSGY-------------L-------IT--F------------------

------SGGAVSWQSRLQKCVAL-------------STIEAEYIAITEASKEL----LWM

KKFLQEL---------GLQ--QER-YL----------LYCDSQS-AIHLSKN-PTF----

HSRSKHIDVRYHW-IR-DALEM-----KLFCLEKI---H-TDE----------NG-----

-SDMMTK------------------------------------

>Humnum

--------------------------------------TGYVDA-----DWASNSV----

--------DRRSYTGM-------------C-------FL--L------------------

------SNGIISWECRKQKCVAL-------------SSTESEYVGVSEACREV----LYL

RSLQFEI---------TNK--MYT-FS----------LFNDNQG-VQKLCAN-PVF----

HKRTKHIDIKHHF-CR-DLVKD-----NIVKLVYL---P-SAD----------MP-----

-ADILTK------------------------------------

>Mtanga

--------------------------------------EAYVDA-----DWAGDHQ----

--------DRKSNSGF-------------I-------FH--L------------------

-------GGPISWSARKQQCVTL-------------SSTEAEYVALAEACREL----LWL

QKLMKDV---------GEK-TTGP-IV----------IREDNQS-CLAMLPA-EGG----

CRRTKHIDTRYNF-IR-DLVNN-----NVIQVQYC---P-SER----------MI-----

-ADALTK------------------------------------

>Copia

--------------------------------------IGYVDS-----DWAGSEI----

--------DRKSTTGY-------------L-------FK--M------------------

-----FDFNLICWNTKRQNSVAA-------------SSTEAEYMALFEAVREA----LWL

KFLLTSI---------NIK-LENP-IK----------IYEDNQG-CISIANN-PSC----

HKRAKHIDIKYHF-AR-EQVQN-----NVICLEYI---P-TEN----------QL-----

-ADIFTK------------------------------------

>Koco

--------------------------------------VGYVDS-----DWGGDEV----

--------DRKSTTGY-------------L-------FK--M------------------

-----FDSNLICWNTKKQNSVAA-------------SSTEAEYMALFEAVRES----LWL

KSLHNSV---------NIQ-IESP-IK----------IYEDNQG-CISIANN-PSC----

HKRAKHIDIKYHF-TR-EQVEI-----NTIFIEYV---P-TEN----------QI-----

-ADIFTK------------------------------------

>Yokozuna

--------------------------------------IAYSDA-----DWAADRT----

--------DRKSTSGC-------------A-------IYQ--------------------

------GQNLITWFSRKQTCTAL-------------STAEAEYVAGAHSMSEL----IHI

KGILSDIL--------STS--IIS-AH----------LLIDNQS-TIKLIQN-QMN----

TKASKHIEIKYHY-IK-DLIYK-----KIVSISYV---P-TNE----------NI-----

-SDIFTK------------------------------------

>Tricopia

--------------------------------------RAYVDA-----DWGNCTE----

--------DRRSFTGF-------------I-------FL--L------------------

------NGSAIS-DTKKQRTVAL-------------STTEAEYMAMAECAKEA----IYL

RRFIQEL---------GFD-KLAD-VK----------IYCDNQS-AIRLAGN-PVF----

HARSKHIDVRHHF-VR-EVLRD-----KQVSLEHI---P-TEQ----------QV-----

-ADFLTK------------------------------------

>Hydra12

--------------------------------------VAYCDA-----DFASDLN----

--------DRRSTTGY-------------C-------FS--LS-----------------

-----ANGPLISWKSKKQSTVAL-------------STCEAEYMALTVTTQES----MYL

VQLLDSM---------ENDCMYMP-VQ----------IFEDNQG-AIALSKN-PVC----

RQRCKHVDVRYHF-IR-SALSD-----GKVTVEYC---P-TED----------MV-----

-ADVMTK------------------------------------

>Hydra11

--------------------------------------YAFCDA-----DWASSLE----

--------DRHSISDY-------------C-------FS--LC-----------------

-----TDGPVVSWKSKKQSSVAL-------------STCEAEYMSISAACQEI----SYL

AKLLREL---------LEV-KIEP-VT----------LRNDNQG-AIALAKN-PIK----

HMKSKHIDIRYHF-IR-EYHQQ-----GRILLEYI---Q-SNE----------NY-----

-ADIFTK------------------------------------

>1731

--------------------------------------TGFVDA-----DWGGDRL----

--------DRKSYTGY-------------V-------FF--L------------------

------SGGPVSWRSEKQQSVAL-------------SSTEAEYMALTTACKEA----IAL

RRLIVEIV-------CG-D-LKTP-TV----------MHGDNLKCAAQLAKN-PVH----

HSRTKHIDIRYH---R-EVMKE-----GHVVLEYT---S-TNE----------MI-----

-ADINTK------------------------------------

>Xanthias

--------------------------------------MGFADA-----DWANDLS----

--------DRKSYSGY-------------A-------FF--L------------------

------GGSAFSWTSAKQSVVAM-------------SSTEAEYVALSTAAKEA----VYL

RRLLLEI---------GWS-LDGP-IT----------ICGDNIS-SHHIAKN-PVH----

HKRTKHIDIKYHF-VR-EKVEC-----NEIILEYV---P-TDK----------NV-----

-ADVLTK------------------------------------

>pCretro6

--------------------------------------VGYSDA-----DWGANPD----

--------DQKSISGY-------------V-------FL--L------------------

------GGAPVCWASRKQKSVAL-------------SSMEAEYMAGSTAASQA----LWC

RMLLEEL---------GFA-QPNP-TL----------LYMDNQS-ALALARN-TGT----

QGRAKHIDIRYHF-LR-DKISS-----KEISVAHC---P-GED----------NP-----

-ADIFTK------------------------------------

>pCretro3

--------------------------------------TGYSDA-----DW-AQAH----

--------DRRSVSGY-------------A-------YL--L------------------

------AGGIVSWNSKKQPTVAL-------------STMEAEYIALSHAAKEA----VWL

RRLLTEL---------GFP-PGAP-TV----------LHTDNQA-AISFAHD-TQF----

HARSKHIDIRHHF-IR-ERITD-----GDIKVIHC---A-SAD----------NI-----

-ADMFTK------------------------------------

>PyRE1G1

--------------------------------------EGWVDS-----DFAGDTQ----

--------SRKSTTGF-------------V-------VT--L------------------

------HGGAISWRSRMQRLVAT-------------STAIAEYVAAAEAVKDS----LWL

RRLAGDL---------GE--YAGP-VT----------LKEDNQA-CIAMANN-EGM----

SPRTKHVDVCYHL-IR-DCVAT-----QQVVVVYV---P-TGE----------QL-----

-ADGFTK------------------------------------

>Osser

--------------------------------------HGYCDS-----DYAACVD----

--------TRRSTTGY-------------V-------FLN--------------------

------AGGAVSWSSRLQPTVAT-------------STAEAEYMASGAATKEA----LWY

RHLARDL---------QMR--VSS-VP----------ILCDSQA-AIKIINN-PIS----

SARSKHIDVLHHF-VR-ERVAR-----GEIVFSYC---K-SAD----------MV-----

-ADCLTK------------------------------------

>Tse1

--------------------------------------HAITDA-----AFANLD-----

--------GYGSQVGY-------------F-------IR--L------------------

------NNKIIGGSSSRAKLTCT-------------SSTEAEIYAVSRAIPML----DSL

KLLIPKI-------------SPTK-LNA--------EIKSDSMS-TINIATSDDDK----

KFRNRFFGTKAMR-IR-DEVQQ-----LGLNIKYI---N-TEE----------NT-----

-ADVLTK------------------------------------

>Ty1B

--------------------------------------VVISDA-----SYGNQP-----

--------YYKSQIGN-------------I-------YL--L------------------

------NGKVIGGKSTKASLTCT-------------STTEAEIHAISESVPLL----NNL

SYLIQEL-------------DKKP-ITK--------GLLTDSKS-TISIIISNNEE----

KFRNRFFGTKAMR-LR-DEVSG-----NHLHVCYI---E-TKK----------NI-----

-ADVMTK------------------------------------

>Tkm1

--------------------------------------TAVSDA-----AFAGNQ-----

--------DFKSQSGT-------------L-------YLR--------------------

------NNKPIAAKSRKIKLTCI-------------SSTEAEIYAISESLPIL----RGL

EHLVNKLQ------------DIKATVK----------VKTDSQP-SMAIINGTDDS----

ACLKKHIGSRAMR-IR-DECDD-----LGLTLEYI---P-TKE----------NN-----

-ADVLTK------------------------------------

>pCal

--------------------------------------ECFSDA-----SFAPGL-----

--------DRKSISGT-------------L-------IY--V------------------

------NGNLVQWATKKQTVIAQ-------------SSAACEMLALNYTMLKA----IEI

KNHLMDL---------GFE--VGK-IH----------CHQDNQA-VIKVLRN-N-Y----

CHPHRPIDICYKF-LR-QLIND-----KVFSISYV---K-TND----------NY-----

-ADCMTK------------------------------------

>Ty2

--------------------------------------VAISDA-----SYGNQP-----

--------YYKSQIGN-------------I-------FL--L------------------

------NGKVIGGKSTKASLTCT-------------STTEAEIHAVSEAIPLL----NNL

SHLVQEL-------------NKKP-IIK--------GLLTDSRS-TISIIKSTNEE----

KFRNRFFGTKAMR-LR-DEVSG-----NNLYVYYI---E-TKK----------NI-----

-ADVMTK------------------------------------

>Tdh2

--------------------------------------QTFSDG-----SFAPTK-----

--------DRRSITGY-------------T-------VY--L------------------

------NGNLINWSTIRQKVITD-------------SSAACEINALHSAVRST----LKS

RQAILDL---------NLV--IDE-IT----------LFEDNAA-VIANCNN-EGT----

SYSRRMVDIKLKF-IR-QLVSE-----GILKLKYV---N-TSI----------NI-----

-ADMLTK------------------------------------

>Ty4

--------------------------------------IAITDA-----SVGSEY-----

--------DAQSRIGV-------------I-------LWY--------------------

------GMNIFNVYSNKSTNRCV-------------SSTEAELHAIYEGYRDS----ETL

KVTLKEL---------GEG-DNND-IV----------MITDSKP-AIQGLNR-SYQ----

QPKEKFTWIKTEI-IK-EKLK------RSITVKIT-----GKG----------NI-----

-ADLLTN------------------------------------

>Zeco1

--------------------------------------VVFSDA-----SLGN-L-----

--------PDGGTQGG-------------------TLIG--LM-------GEGGKF----

--------SPLCWQSKKIRRVVR-------------STLAGETLALSDGIDNAIFLTTLF

SELTI-----------GNAELNTPPLI----------CVTDNHS-LFDALKSTKQV----

-SEKR---------LR-----------LEISSIKD-LMQ-SKKIKKVLWSDTKTQ-----

LADCLTK------------------------------------

>GalEa1

--------------------------------------EYFSDA-----SFANL------

--------AGGGSQGG-------------------FVVF--LR-----DD--SGRR----

--------CPIYWQSKKIWRVVK-------------STLSAETLALLDYAEAAVYLVRIL

DEISDC----------GSLR-----VK----------CYVDNKS-LVDALQSYKGV----

-EDRR---------LR-----------IDIAVLRN-MLE-RKEIGEVDWVDASRQ-----

LADCLTK------------------------------------

>Cico1

--------------------------------------AVFADA-----AFGN-L-----

--------PDGGSQGG-------------------YLVF--LV-------GQDGQC----

--------NLLSWQSRRIRRVVR-------------STIAAETLAMSDGIEAAIYINSLF

CEILF-----------GNKHERRLPIE----------AITDNRS-LNDAVKSTKYV----

-KDKR---------LR-----------IEISLIKD-LVS-SKEITSLKWIPTEKQ-----

LADILTK------------------------------------

>Zeco2

--------------------------------------VVFSDA-----SLVI-F-----

--------PDGGTQGG-------------------TLIG--LM-------GEGGKF----

--------SPFCWQSKKIRRVVR-------------STLAGETLALSDGIDNAIFLTTLF

SELTT-----------GNAELNTPPLI----------CVTDNLS-LFDALKSTKQV----

-SEKR---------LR-----------LEISSIKE-LMQ-SKKIKKVLWSDTKTQ-----

LADCLTK------------------------------------

>Olco1

--------------------------------------MVFSDA-----SFGN-L-----

--------SDGGTQGG-------------------HLIV--LM-------GEHGKF----

--------SPLSWQSKRVKRIVR-------------STLAGETLAMSDGIDNAYFLAILF

SELT-----------TGSVE-HAPAII----------CVTDSRS-LADALKSTKSV----

-SEKR---------LR-----------LEISAIKE-LIQ-AQRVERVLWYNTKEQ-----

LADCLTK------------------------------------

>CoDi4.4

--------------------------------------NAFVDA-----DHAGDQV----

--------TRRSRTGV-------------L-------IF--V------------------

------NKAPVIWFSKRQNTVET-------------STFGSEFVAMKIATELL----IGL

RYKLRMM---------GLP-LDGP-AN----------VFGDNQS-VVTNASR-SES----

VLKKKHVSICYHR-VR-EACAA-----DIIRIAHE---S-TKT----------NL-----

-ADLLTK------------------------------------

>CoDi3.1

--------------------------------------SVFVDA-----AHADCHV----

--------TRRSTTGI-------------L-------AF--I------------------

------NGTPIRWYSKRQNTVEA-------------STYGSEFVAMRIASEMI----ITL

RYNLRVL---------GIP-IDGP-AN----------VFCDNMS-VVTSATI-PSS----

VLKKKHNAISYHK-VR-ESIAS-----GAMRIAHE---P-TGS----------NL-----

-ADTLTK------------------------------------

>CoDi2.4

--------------------------------------TSYVDA-----NLYHDMV----

--------TGRSVTGV-------------L-------HL--V------------------

------NQTPFEWYSKRQATVET-------------ATYGSEFVAARIAVEQI----IDI

RTTLRFL---------GVP-IRGK-SV----------LFGDNQS-VIISSTE-PQS----

PINKRHNALSYHR-VR-EAIAA-----GIVDFRKI---L-GAE----------NV-----

-ADVLSK------------------------------------

>CoDi4.1

--------------------------------------TCFVDA-----DHAGCRL----

--------TRRSHTGV-------------L-------IY--V------------------

------NRAPILWYSKRQNTVES-------------STYSSEFCAMRTAIDMI----EGL

RYKLRML---------GVG-LDGP-TC----------VLCDNQS-VVISSTA-PET----

ALKRKHNAINYHR-TR-EAQAA-----GIVLIAKE---P-TET----------NI-----

-SDFLTK------------------------------------

>CoDi4.5

--------------------------------------RLYVDS-----DHAGDKV----

--------TRRSRTGY-------------I-------IY--L------------------

------NSAPIQWLSKKQSTVET-------------SVFGAEFVAMKHGIETV----RGI

RYKLRMM---------GIE-VDNP-TY----------VYGDNMS-VVTNSSK-PES----

QLKKKCNSICYHA-VR-ESVAM-----GESLVSHI---S-TDK----------NP-----

-ADLMTK------------------------------------

>CoDi4.3

--------------------------------------TAFVDA-----DHAGDKV----

--------TRRSRTGV-------------L-------IY--L------------------

------NRAPIMWFSKRQNSVET-------------SSFGSEFVALKIATEMI----QGL

RYKLRMM---------GIP-IDGP-AR----------VLCDNMS-VVHNTTA-PES----

MLKKKSNAIAYHF-VR-ENVAM-----GVIKIAYE---P-SET----------NL-----

-ADALTK------------------------------------

>CoDi7.1

--------------------------------------TVFVDS-----DHAHDKV----

--------SRRSITGL-------------L-------IF--VGRTP--------------

----------VFYTSKRQGAIET-------------STYGAEFCGMKTAVEEL----IAV

RYMLRCL---------GVK-VEHA-SM----------ICGDNLG-VIQNATI-SES----

LLKKKHVAIAYHK-TR-EAAAA-----GICHPIKT---G-GVD----------NF-----

-ADTLTK------------------------------------

>CoDi5.2

--------------------------------------HVHSDA-----SYLSET-----

--------KARSRSGG-------------I-------FF--LSSKPIKDPKPNSEP----

----PIFNGAIHVHCSIMKSVLS-------------SATEAELGALFYNAKDA----IEL

RTTLEAM---------GHP--QLA-TP----------IQTDNEC-ASGIVNE-TVK----

QRQSKAIDMRFYW-IK-DRVKQ-----GQFNVHWR---K-GVD----------NL-----

-ADYFTK------------------------------------

>CoDi5.3

--------------------------------------HVHSDA-----SYLSES-----

--------KARSRAGG-------------I-------FF--LSSAPIKNLKPNSKP----

----PPLNGAIHTHCSIMKSVLS-------------SATEAELGALFFNAKDG----VEL

RTTLEAM---------GHP--QLA-TP----------IQTDNEC-ASGIVNN-TVK----

QRRSKAIDMRFYW-IK-DRVKQ-----GQFNVHWR---K-GTD----------NL-----

-ADYFTK------------------------------------

>CoDi5.1

--------------------------------------HIHSDA-----SYLSES-----

--------KARSRAAG-------------H-------FF--LSSRP---HDPNAAPAPTG

P--DPPNNGAIHTHSSIMSVVLS-------------SATEAELGALFYNAKDA----TAF

RVTLDEL---------GHI--QPP-TP----------IQTDNAC-ASGIANE-TIK----

QRRSKAIDMRFYW-VK-DRVEQ-----KQFIIYWR---P-GLT----------NL-----

-ADYFSK------------------------------------

>CoDi5.4

--------------------------------------ALDTDG-----SYLSEP-----

--------GAKSRAAA-------------Y-------FY--LTKKD--------EP----

----EFHNGSVLILSSIIKHIMA-------------SASETELAALFYGCKEA----IPL

RNTLEEM---------GHP--QPP-TP----------VTTDNST-AIGLTMD-TMT----

PKASKSMDMRFQW-LK-SRRAQ-----HLFRYHWA---K-GTT----------NR-----

-ADYPSK------------------------------------

>CoDi5.5

--------------------------------------AAHSDA-----SYHSEP-----

--------KARSRAGG-------------H-------FF--LSSDGLY------------

----PHNNGAILNIAQIIKTVMS-------------SAAEAELGALYINAREA----VWI

RRVLEEM---------GHT--QTK-TP----------MQTDNST-AEGVVNN-KIQ----

PKRTKAMDMRFYW-LR-DQEAK-----NQFRFYWA---P-GAT----------NY-----

-ADYWTK------------------------------------

>CoDi5.6

--------------------------------------NVHSDA-----AYLVAS-----

--------RARSRAAG-------------H-------FF--LGWTP-----QNNKP----

----IKLNGAFYTTCELLKFVAG-------------SAAEAELGALFLNAQKI----KIF

RRTLEEM---------GHP--QPP-TP----------THCDNTT-AVGIANN-TVK----

RQRSRAMEMRYFW-VA-DQVAN-----KQLSVQYH---P-GQE----------NL-----

-ADYTTK------------------------------------

>CoDi6.1

--------------------------------------ECYVDA-----DFAGAWNRVFA

ATDPSTA---KSRGGW-------------I-------VF--Y------------------

------AGCPIIWASKLQTQVAL-------------STTEAEYIAMSMALRDV----IPI

MELVREMKNRKFEVICTEPLVY---CK----------VFEDNSG-ALELARL-PKL----

RPRSKHINMCYHH-FR-EHVRK-----GLIKIFPV---F-TDD----------QV-----

-ADALTK------------------------------------

>CoDi6.7

--------------------------------------EVYADA-----DFSGNWDREEA

TDDPDTA---RSRTGF-------------A-------IF--Y------------------

------AGCPVTWQSKLQTEISL-------------STTESEFVSLSTALRTA----LPL

IGLAKEMSSLGFDISVTVPTVH---CK----------AFDDNMG-AIEIALV-PKM----

RPRTKHINVKYHH-FR-QHVDN-----GDITIQHV---D-SED----------QI-----

-ADFLTK------------------------------------

>CoDi6.6

--------------------------------------DCYADA-----DFAGLWN----

AKQPNVVGNLRSRTGY-------------L-------LT--L------------------

------GNTPVVWASKLQGVIAL-------------STMESEFISLSAALKAL----IPL

-------RNTHFRISEALSLPYEPESR----------IFEDNQA-CITLATTDPRM----

TPRSKHIAIRYFW-FR-EHLRT----CKALKIVYI---P-TTH----------QR-----

-ANHLTK------------------------------------

>CoDi6.4

--------------------------------------IIYADS-----DYASDKK----

--------DRKSISGD-------------L-------ST--I------------------

------GKTLVNWRSKKQTGVTL-------------SSTEAEYVALSHAATEA----KFL

LMLLEETT--------GTF--HGP-AI----------IHEDNQG-AIFIANN-DSL----

GQRTKHIDIRYRY-TN-QLIQE-----GLIVLKYI---K-TDE----------NY-----

-ADLETK------------------------------------

>CoDi6.3

--------------------------------------VCYSDS-----DYAGDPD----

--------TRRSVSGY-------------I-------LY--V------------------

------KGVPICWRSKAQRSITL-------------SSSEAEWIALSEATKEI----MFV

LQLLESL---------YIK-VQLP-IT----------VRVDNIG-AIWMSQN-VNT----

SSRTKHVDIRTKY-VN-EYCED-----GVLKIIFV---K-SAD----------ND-----

-SDIMTK------------------------------------

>CoDi6.2

--------------------------------------VVYSDS-----DYATDPD----

--------TRRSTSGY-------------I-------LY--L------------------

------RDVPIAWKSKAQQSVSL-------------SSTEAEWIALSEAVKEI----KFV

VNLLESM---------KIK-VNYP-IK----------CRVDNIG-AIFMSQN-VTT----

TSRAKHIDIRTKF-VR-EYVED-----GKIKIVFV---R-SGD----------ND-----

-SDIMTK------------------------------------

>CoDi6.5

--------------------------------------VANVDS-----KYATNKD----

--------DRRSVSGA-------------F-------FT--V------------------

------GGTLTNWMSKTQALVAL-------------SSCEAEYVAVALATQEL----LFM

QMLMTEL---------GEC--EYP-GI----------ILEDNTG-AIFLVKN-QQV----

GQRTKHIDVRYHF-IR-EHYEN-----GEIDITHT---R-SEN----------NE-----

-ADIGTK------------------------------------
