# Supplementary material for: Genome-wide characterization of LTR retrotransposons in the non-model deep-sea annelid Lamellibrachia luymesi
Source: BMC Genomics. 2021 Jun 23;22:466. doi: 10.1186/s12864-021-07749-1 (PMC8220671; doi:10.1186/s12864-021-07749-1)
Supplement: Supplementary file 4 — Additional file 4. Reverse transcriptase sequence alignment file. [file 12864_2021_7749_MOESM4_ESM.docx]

>NC-256_ Flow

------------------------------------------------------------

-----------------------------------------------W------------

-----------------LLPHHPVLSP------H-KPL------------------P--R

VVFDSA-AVHDG-ICLNDCLEAG------------PSL----------HNDL-PGV---L

M----RFRER------PVA-LAGDVSDMFCHVRL---RTEDRKYHRYLW-RDL----ET-

----DKP-PD------------------VYEINCLVFGDKSSPCEANFA-----VQRTAE

DNKEQ---WPD--AA-------------------------AVVKRDIFVDD-LYTSCE-D

V------GE---AVKLREDITALMAEGGFPMRKWMSSSAEVLATVPETE-------RAVR

NETLE--------MGELPQGRALGVRWDPSD-----------------------------

------------------------------------------------------------

--------

>NC-228_Dan

------------------------------------------------------------

----------------------------------------------SW------------

-----------------YLPHHIVTHN------G----------------------K-HR

VVFNCS-YTYNG-ENLNKQLLPG------------PTL----------GPTL-LGV---L

I----RFREH------RVA-ISGDIKGMFHQVRL---LPKDKPLLRFLW-RDL----NP-

----ENP-PD------------------VYEWQVLPFGTTCSPCCATFA-----VQRHVF

SHCQQ---GDK--LR-------------------------QSVEHSFYVDN-CLQSTS-S

A------VE---ARELIDGLRSILASGGFEIRQWASNEATVVRHLPKEA-------KSEN

TELKWISQSQ-----ADPHEMTLGLSWHCVPD----------------------------

------------------------------------------------------------

--------

>NC-290_Dan

------------------------------------------------------------

----------------------------------------------SW------------

-----------------YLPHHLVHHN------N----------------------K-AR

LVFNCS-YQYRG-LALNEHLLPG------------PIL----------GPSL-LGV---L

L----RFREH------TVA-ISGDIKAMFHQIRL---LPEDRSLLRFLW-RDM----KK-

----NEE-PN------------------VFEWQVLPFGTTCSPCCATYA-----LQRHVK

DNCVG---YED--VQ-------------------------MSVEQAFYVDN-CLQSLN-C

P------EK---ARSLIDRMRSHLSSGGFEIRQWASNIPQVIEHLPIEA-------RAAS

TEL-WVSEHS-----SDPQEPALGLRWNCLTD----------------------------

------------------------------------------------------------

--------

>NC-219_Dan

------------------------------------------------------------

----------------------------------------------SW------------

-----------------YIPHHVVHHN------N----------------------K-DR

IVFNCS-FQHQG-QSLNSQLLPG------------PTL----------GPSL-LGV---L

L----RFRQH------AVA-VSGDIKGMFHQVRL---LPKDKQVLRFLW-RDM----CR-

----DRE-PD------------------IYEWQVLPFGTTCSPCCAIFA-----LQHHAQ

GHKGD---MPK--LV-------------------------NIVENSFYVDN-CLYSTP-T

A------NE---ARDVVDGLRQLLAEGGFDLRQWACNVPSVIQHLPAEA-------LSTN

SER-WLTKGS-----TDLQEPTLGLRWNCLND----------------------------

------------------------------------------------------------

--------

>NC-183_Dan

------------------------------------------------------------

----------------------------------------------SW------------

-----------------FIPHHMVHHN------G----------------------K-DR

IVFNCS-FQHHG-QSLNDQLLPG------------PTL----------GPSL-LGV---L

L----RFRQH------TVA-ISGDIKGMFHQVRL---LPGDKSVLRFLW-RGM----CR-

----EVE-PE------------------IYEWQVLPFGTTCSPCCAIHA-----LQHHVQ

EHKEG---KTE--LV-------------------------DIVEHSFYVDN-CLHSVP-T

A------SE---AKVIVDGLRQLLSEGGFEIRQWACNVPSVTEHLPSDA-------RSAS

SER-WLAQSS-----TDLQEPTLGLRWDCIND----------------------------

------------------------------------------------------------

--------

>NC-1118_Dan

------------------------------------------------------------

----------------------------------------------SW------------

-----------------FVPLHLVKHN------E-KY----------------------S

LVLNCS-YVFKG-DSLNTHLLPG------------PTL----------GSSL-LGV---L

L----RFREN------PVA-ISCDIKSMFHQVRL---LPSDKPLVRFLW-RNM----RK-

----EEP-AD------------------VYEWHVLPFGTTCSPCCATYA-----LQKHVR

DLSEN---CED--LK-------------------------DSITKAFYVDN-CLQSVS-D

I------QK---GKELVEKLRAHLKEGGFEIRQWASNVLEVVEVLPSDM-------RSAH

TEL-WLSQDCV-----DPSEGMLGLTWHCAKD----------------------------

------------------------------------------------------------

--------

>Jule

------------------------------------------------------------

--------------------------------------KS------EW------------

-----------------ASPIVTVPKT------D---K------------------T-IR

ICGDYK-VS------IN-------QCIEEKHYP-LPN-----------AEDL-FAT---L

E-GG------------TSF-SKLDLSHAYQQLEL---DEESEKYLVI-------------

----NTH-KG------------------LYKYSRLSYGVSSAPAIFQSV-----MDQ---

-------ILQG--M----------------------------DHVTCFLDD-ILITAS-S

E------K----HLQRLEEVLTRLEKHGLRVK--------------LAKCRF--------

---------------LQSSVEYLGHRI---------------------------------

------------------------------------------------------------

--------

>PYG1

------------------------------------------------------------

--------------------GTISPV-----------EFS------EW------------

-----------------ATPIVPIVKS------D---K------------------S-IR

ICGDYK-VT------VN-------KVSKLDNYP-IPK-----------TEVL-YAT---L

G-GG------------TDY-SKLDLSQAYQQLEL---DDSLKPYTTI-------------

----NTH-KG------------------LFVYNRLPYGVASAPGIFQRT-----MEN---

-------LLQG--I----------------------------PQVIVRVDD-ILVTGK-N

R------RQ---HLENLEAVLNRLDKAGVKLK--------------RSKCYF--------

---------------LRKEVEYLGHRI---------------------------------

------------------------------------------------------------

--------

>JRE

------------------------------------------------------------

------------------------------------------------------------

---------------------------------E--------------------------

---------------LDDS-----KTT---------------------------------

------------------------------------------------------------

----ITH-KG--------------------------YRLYVASPGFQRT-----------

-------LLQG--P--------------------------------QVVDD-LVTGRR--

---------------QLNEVLNRLDKAGVKLK--------------KDKCEF--------

---------------MEKEVIFLGHKV---------------------------------

------------------------------------------------------------

--------

>Steamer

------------------------------------------------------------

---------------------IITKV----------ETPT------DW------------

-----------------VSSLVVVKKP------S---G------------------K-LR

ICIDPK-P-------LN-------KALKRSHYP-LPI-----------IEDL-LPE---L

S-EA------------KVF-SKCDVKNAFWHVKL---DEESSYLTTF-------------

----ETP-FG------------------RYRWNKMPFGISPAPEYFQQF-----LEK---

-------NLEG--L----------------------------DGVKPIADD-ILIYGK-G

ETFQDAVKD---HDRKLEKLLKRCKERNIKLN--------------KDKFEL--------

---------------HKTEMPFIGHL----------------------------------

------------------------------------------------------------

--------

>LLGY1

------------------------------------------------------------

-------------------------------------LGV------PY------------

-----------------CRP------P------K-KHG------------------S-IR

LCIDMR-E-------PS-------KAIIRELHN-MPT-----------LDEL-IHD---L

N-GA------------TVF-SKLDLSSGYHQLEL---HPSSRYITTF-------------

----RTH-NG------------------LYMYKRLNFGISSASEIFQEH-----VRN---

-------VISY--I----------------------------ESTRNISDD-IIIYGK-G

E------NAEEMHDRALADTLEALYTNGLTLN---------------DKCKV--------

---------------NLPSIEYYGMIF---------------------------------

----SKTGVSSDPSKVQALKELPPPSNVAK--LMSFLGMMS---YL----------SRFI

KDC-----

>LLGY2

------------------------------------------------------------

----------------------------------------------DW------------

-----------------VNSLVIAEKS------N---G------------------K-MR

LCIDPK-D-------LN-------KEIKREHFQ-IPT-----------KEEI-IGK---L

A-NA------------TCF-SKLDATAGFHQIQL---DRPSSLLTTF-------------

----NTP-FG------------------RYRYLRLPMGICSAPEVFHKT-----VHQ---

-------FLED--I----------------------------EGVSVYMDD-IIVWGS-T

A------AE---HDERLMKTLQRLSEVGLGLN--------------MEKCVF--------

---------------RQPEISYLGEVV---------------------------------

----TQDGVKPDPEKIQAITDMPTPTNATE--LQRVLGMVT---YL----------GRYI

PNLS----

>LLGY3

------------------------------------------------------------

-------------------QGVIIAV----------NEPT------DW------------

-----------------VNSLVKTEKR------D---G------------------S-LR

LCLNPK-H-------MN-------KDIRREHFQ-IPT-----------FTEI-STQ---L

G-GA------------RLF-TILDQKDSYWQVEL---DKDSSLLCCF-------------

----NTT-Y---------------------RFVRMPFGITSASEVLQKW-----THE---

-------TFGD--I----------------------------PNVHIVADD-MLIAVQ-T

E------AE---HDSTLRKVMER-----VKFN--------------MKKTQL--------

---------------KKSEVFYMGTMN---------------------------------

----SADGML--------------------------------------------------

--------

>LLGY4

------------------------------------LG----------------------

---------------KLEKAGVITPV-----------RSS------DW------------

-----------------ATGVVVVPKK------N---G------------------A-IR

LCGNYK-TT------VN-------PQLKTVSPP-NIN-----------IDDI-LAD---L

A-GG------------VKL-SELDLANAYTST----------------------------

------------------------------------------------------------

------------------------------------------------------------

------------------------------------------------------------

------------------------------------------------------------

------------------------------------------------------------

--------

>LLGY5

------------------------------------------------------------

------------------------------------------------------------

------------------------------------------------------------

------------------------------------------------------------

----------------KKK-TVFDAWNGYHSVPI---RECDRHLTTF-------------

----ITP-WG------------------RYRYCTTPQGYIASGDGYTRR-----FDE---

-------IVAD--F----------------------------PNKTKCIDD-TCIWGD-T

I------EE---CFVQACAWLDQCGRNGITLN--------------PEKFRF--------

---------------AQDIVEFAGFTI---------------------------------

----TPDEVRPCSKYLDAIMQFPVPRNITD--VRSWFGLVNQVSY---------------

--------

>LLGY6

------------------------------------------------------------

------------------------------------GTPV------TW------------

-----------------CHRMVICAKK------N---G------------------K-PR

RTVDFQ-A-------LNKH---AFRETHHTQSP-FHQ----------------ARR---I

P-NG------------KRK-TVFDAWNGYHSVPL---HEDDRHKTTF-------------

----ITP-WG------------------RYRYLSAPQGYIASGDGYTRR-----YDE---

-------IVAD--I----------------------------GDKTKCVDD-TLLWSD-T

I------EG---SYFQAVQCLDICGRNGIILN--------------PEKFVF--------

---------------SAPTVDFAGFTI---------------------------------

----TMTDVRPCSRYLEAIRDFPQPRNITD--IRSWFGLVNQVAYAFSMAERMHPFRKLL

KNGKR---

>LLGY7

------------------------------------------------------------

------------------------------------DHPT------KW------------

-----------------CHPLVVVPKS------R---G------------------G-VR

LCVDLT-Q-------LN-------KHVRRPIHP-MKT-----------PKEA-VSN---I

TPGS------------KYF-SSLDAKHGYWQIAL---SPECQELTTF-------------

----LTP-WG------------------RYQFLRSPMGLSSTGDEYCRR-----GDI---

-------AIAG--L----------------------------NNIQKVMDD-VIVFDE-N

F------ER---HVERVRTLLQRCREHGITLN--------------ADKFVF--------

---------------AENELNYVGYKV---------------------------------

----NADGVTTDPEKLKAIAEFPAPNCLTE--LRSFMGLVN---QL----------GDFT

--------

>LLGY9

------------------------------------LE----------------------

---------------RLQKEGIISPV-----------EYS------QW------------

-----------------ASGVVPVAKR------E---T-----------------KD-VR

LCGDYK-VT------IN-------PVLREDKYP-LPR-----------IEDI-FAK---M

A-GG------------KRF-SKIDLKNAYLQMEV---EEDSKKYLTI-------------

----NTH-KG------------------LFKYNRLPFGIKTAPSIWQRA-----MEQ---

-------TLQG--I----------------------------PGVEVMLDD-IIVTGK-S

D------AE---HLENLEAVLRRLAEKDLRIN--------------AKKCRF--------

---------------FMERIEYCGHEI---------------------------------

----DHDGLHKTKAKIEAVQKAPHPQDVSS--LR-V------------------------

--------

>LLGY10

------------------------------------------------------------

-----------------------------------------------W------------

-----------------VSRIMTPHKP------K-KPD------------------E-IR

LCVDMR-E-------AN-------KAILRTRHV-TPT-----------LDEL-ITA---F

N-GA------------TVF-SKIDLRSGYHQLVL---HPSSCHITTF-------------

----STH-VD------------------LYRYKRLSSGINAAAEVFQHE-----IKT---

-------VIHG--V----------------------------SGAINISD----VFGV-D

Q------CS---HDKALDDVQHKLQTAGLTAN--------------LEKCEF--------

---------------RKKKIEFFGLIF---------------------------------

----SGEGVSPDPKKVADLHRAAEPKNASE--VRSFLGMAQ---VH----------Q---

--------

>LLGY11

------------------------------------------------------------

-----------------LHDEIIRP------------SNS------AY------------

-----------------SSPILLVPKK------D---G------------------D-SR

LCVDYR-K-------LNA------VTE-QDAYP-LPI-----------IQDI-FDL---V

G-GS------------SIY-STLDLKSSYWQMPV---AEADIHKTAF-------------

----RCH-AG------------------HFEFTKVPFGLKSAPNFFQKE-----MNT---

-------ILAD--L--------------------------IGKCVFVYIDD-ILVFSK-N

E------TD---HIRHLQLVFDRLRNAGLKLK--------------PTKCAF--------

---------------GLPEVKLLGYVL---------------------------------

----NADGIKTDPDKVAVIANLLPPTTVKE--TRSFLGMCN---YY----------RNSL

PKYA----

>LLGY12

------------------------------------------------------------

------------------------------------------------------------

------------------------------------------------------------

-----K-P-------LN-------KAIKRERYE-IPT-----------PADV-QS----L

G-DK------------QIF-NVIDMKDGYWHVKL---TEESSHLCTF-------------

----YAP-WG------------------RKRFKRMPFGISSASEVIQKR-----NEE---

-------TFAD--I----------------------------PGVRIIAED-MIISAK-D

E------TE---HDAIVRMV----------------------------------------

------------------------------------------------------------

------------------------------------------------------------

--------

>LLGY13

------------------------------------------------------------

------------------EQKVIAKV----------DIPT------PW------------

-----------------ISNCLAIRKS------N---G------------------T-VR

VCIDPT-D-------LN-------KAIQRNHFP-LPT-----------IEEV-LPK---L

K-DA------------KIF-SLVDAKDGFLQVKL---TNESSYLTTF-------------

----WTP-FG------------------KYRWLRMAFGLTSSPEEFQRR-----LQL---

-------ALDG--L----------------------------DGIFIVADD-ILIIGR-G

ETDEEARRD---HDENLARLLQRAREQNLKLN--------------KAKMRL--------

---------------HLTEIKYIGHVL---------------------------------

----SPEGVKADPEKVSDISSMATPTDSDQ--VRRCLGFTN---YL----------AKFL

PNLS----

>LLGY14

------------------------------------------------------------

------------------------------------------------------------

------------------------------------------------------------

------------------------------------------------------------

----------------KKK-TVFDAWNGYHSVPI---RECDRHLTTF-------------

----ITP-WG------------------RYRYCTTPQGYIASGDGYTRR-----FDE---

-------IVAD--F----------------------------PNKTKCIDD-TCIWGD-T

I------EE---CFVQACAWLDQCGRNGITLN--------------PEKFRF--------

---------------AQDIVEFAGFTI---------------------------------

----TPDEVRPCSKYLDAIMQFPVPRNITD--VRSWFGLVNQVSY---------------

--------

>LLGY15

------------------------------------LD----------------------

---------------RLLADDIIEPV-----------QYS------DW------------

-----------------ATPVVPVMKA------D---K------------------S-VR

LCGDYK-LT------VN-------QVAKLDRYP-IPR-----------IEDL-YAQ---L

G-NG------------TSY-TKLDMRHAYEQIEL---HPESRKYVTI-------------

----NTP-RG------------------LFTYKRLPYGVSSAPGIYQRV-----MDS---

-------LLKG--I----------------------------KNTMVYLDD-VLVTGR-T

D------EE---HLQTLDLVMERLMSAGFCLK--------------RQKCHF--------

---------------MVEEVEYLGHRI---------------------------------

----DALGIHPSGHALVAVRDAPAPVNVAE--LRSYLGMVN---HY----------GRFV

SNLS----

>LLGY17

------------------------------------LQ----------------------

---------------RLEDLKIVTKV-----------DYS------DW------------

-----------------ATPIVVVQKP------S---G------------------K-VR

ICGDYR-AT------VN-------PCLHVQQHP-IPR-----------IEEL-FAK---L

Q-GG------------MHF-SKLDMRDAYLQIEL---DDETKQLLVI-------------

----NTH-KG------------------LYRYNRLCFGPSPAPAIFQKL-----VDN---

-------LVAG--I----------------------------PGVAAYLDD-IIVTGQ-T

K------AE---HLENLRRVFAALDNYGLKLQ--------------LDKCVF--------

---------------FAPEVSYLGYII---------------------------------

----SKDGLCASEERVQAILQYATPTDLKQ--LESFVGKLN---YY----------GKFL

PAFA----

>LLGY18

------------------------------------------------------------

------------------------------------------------------------

--------------------ISIQTKD----------A------------------S-LR

VCIDPK-P-------LN-------KVL-QELYP-LPT-----------MEEV-PPE---M

S-TA------------RVL-SKVDLQSGHWHCEL---DHESSLLTTI-------------

----ITP-FG------------------RYRWNRLPFGMNVSAEIFQRK-----LNQ---

-------TMEG--L----------------------------DGVVCVADD-IVVFGR-D

E------ED---HDKKLRLLLQRKR-----------------------------------

------------------------------------------------------------

--------------------------------------------HE--------------

--------

>LLGY19

------------------------------------------------------------

------------------------------------------------------------

--------------------CLAIRKS------N---G------------------T-VR

VCIDPT-D-------LN-------KAIQRNHFP-LPT-----------IEEV-LPK---L

K-DA------------NIF-SLVDAKDGFLQVKL---TNESSYLTTF-------------

----WTP-FG------------------KYRWLRMAFGLTFSPEEFQRR-----LQL---

-------ALDG--L----------------------------DEIFIVADD-ILIIGR--

------------------------------------------------------------

------------------------------------------------------------

------------------------------------------------------------

--------

>LLGY20

------------------------------------------------------------

--------------------------------------PT------PW------------

-----------------VSPIVVVPKP------H-NAN------------------E-IR

ICVDMR-S-------LN-------KAIIRERHI-IPT-----------TDDI-IAD---L

N-GC------------KVF-SKIDLNQGYHQLPL---HPDSRHLTTF-------------

----STH-VG------------------LYRYKRLNFGLSCAAEIFQRK-----VGD---

-------AIRG--I----------------------------PGVRNISDD-IYIGGI-D

E------AQ---HDDRLIKVLQRLKENQLTVN--------------VPKCLI--------

---------------RVPSMLFFGHVF---------------------------------

----SGEGVSPDPKKVEALRSVNAPNNVSE--VRSLLSSAA---FC----------SRFI

KDF-----

>LLGY21

------------------------------------------------------------

--------------------------------------PT------PW------------

-----------------VSPIVVVPKP------H-NAN------------------E-IR

ICVDMR-S-------LN-------KAIIRERHI-IPT-----------TDDI-IAD---L

N-GC------------KVF-SKIDLNQGYHQLPL---HPDSRHLTTF-------------

----STH-VG------------------LYRYKRLNFGLSCAAEIFQRK-----VGD---

-------AIRG--I----------------------------PGVRNISDD-IYIGGI-D

E------AQ---HDDRLIKVLQRLKENQLTVN--------------VPKCLI--------

---------------RVPSMLFFGHVF---------------------------------

----SGEGVSPDPKKVEALRSVNAPNNVSE--VRSLLSSAA---FC----------SRFI

KDF-----

>LLGY22

------------------------------------ID----------------------

---------------RLLAEDIIEPV-----------QYS------DW------------

-----------------AAPVVPVMKA------D---K------------------S-VR

LCGDYK-LT------VN-------QVAKLDRYP-IPR-----------IEDL-YAQ---L

G-NG------------TTY-TKLDMRHAYEQIQL---HPDSRKYVTI-------------

----NTP-RG------------------LFTYKRLPYGVSSAPGIFQRV-----MDS---

-------LLKG--I----------------------------PNTMVYLDD-VLVTGP-T

E------DE---HLQTLDRVLERLVQAGFRLK--------------ESKCQF--------

---------------LSDEVDYLGHRI---------------------------------

----DAEGIHPSGETLSAVRDAPAPTNITE--LRSYLGMVN---HY----------GRFL

PNLA----

>LLBP1

------------------------------------------------------------

-------------------HGYAEPV---------PAEELTIAEGRTW------------

-----------------YIPHHGVYHP------K-KPG------------------K-IR

VVFDCS-AEYRG-EVLNRHLLQG------------PDL----------TNNL-TGV---L

C----RFRQE------PVA-VSCDIESMYHQVGV---NTEDRNFLRFLWWDNC----NL-

----DSE-PK------------------EYRMTVHLFGATSSPGCANYA-----LKATAD

MFEKDCGKLAA-----------------------------DFVRSNFYVDD-GLKSVA-T

P------AE---ALQLVESSRDMCKKGGFNLHKYMCNSKEVLVAISPEL-------RAKD

AQNLDLT------CDSLPIERTLGVQWCIESDTFQFRVEVKDRPL---------------

----------TRRGILSTVSSVFDP-----------LGLVS--PYVLRG-------KHIL

QELV----

>LLGY23

------------------------------------------------------------

------------------------------------------------------------

------------------------------------------------------------

------------------------------------------------------------

----------------SWK-TVTDAWNGYHSVPL---RDSDRHLTTF-------------

----ITP-FG------------------RWRYTRAPQGFLSSGDGYNRR-----FDE---

-------IIAN--F----------------------------QRKERCIDD-TVHWDV-E

L------QN---HWWRTIDYLILVGRAGVVLN--------------PDKFQF--------

---------------AQRTVDFAGFRI---------------------------------

----SNATIEPLPKYLDAIRDFPTPTSTTD--VRSWFGLVNQVTNY--------------

--------

>LLGY24

------------------------------------------------------------

------------------------------------DGPT------PW------------

-----------------VSPIVVVPKK------T---G------------------A-VR

ICVDMR-E-------AN-------KAVKREKHL-MPT-----------IDDL-ITD---L

N-GA------------TMF-STLDLRAGYHQLEL---DPESRQITTF-------------

----STH-VA------------------LYRYKRLMFGINAASEIFQNT-----IAE---

-------LLHG--L----------------------------NGCRNISDD-IIVHGK-T

P------AE---HNANLRAVLDRLRENNVRLN--------------REKCKF--------

---------------SQPTVTFYGHVF---------------------------------

----GAHGLRADPKKLESISKARRPTNPSE--VRSLLGMAQ---YV----------SRFI

ADF-----

>LLGY25

------------------------------------------------------------

------------------------------------------------------------

------------------------------------------------------------

------------------------------------------------------------

----------------KKK-TVFDAWNGYHSVPI---RECDRHLTTF-------------

----ITP-WG------------------RYRYCTTPQGYIASGDGYTRR-----FDE---

-------IVAD--F----------------------------PNKTKCIDD-TCIWGD-T

I------EE---CFVQACAWLDQCGRNGITLN--------------PEKFRF--------

---------------AQDIVEFAGFTI---------------------------------

----TPDEVRPCSKYLDAIMQFPVPRNITD--VRSWFGLVNQVSY---------------

--------

>LLGY26

------------------------------------------------------------

----------------------------------------------DW------------

-----------------VNSLVIAEKS------N---G------------------K-MR

LCIDPK-D-------LN-------KEIKREHFQ-IPT-----------KEEI-IGK---L

A-NA------------TCF-SKLDATAGFHQIQL---DRPSSLLTTF-------------

----NTP-FG------------------RYRYLRLPMGICSAPEVFHKT-----VHQ---

-------FLED--I----------------------------EGVSVYMDD-IIVWGS-T

A------AE---HDERLMKTLQRLSEVGLGLN--------------MEKCVF--------

---------------RQPEISYLGEVV---------------------------------

----TQDGVKPDPEKIQAITDMPTPTNATE--LQRVLGMVT---YL----------GRYI

PNLS----

>LLGY27

------------------------------------------------------------

---------------------IIKEV----------NTPT------DW------------

-----------------VSNMVIVKKK------S---G------------------K-LR

LCIDPK-P-------LN-------KALKRSHYM-LPV-----------IEDI-IPE---L

A-SA------------KVF-TVCDVKIAFCHIEL---DEESRRHTD--------------

------------------------------------------------------------

------------------------------------------------------------

------------------------------------------------------------

------------------------------------------------------------

------------------------------------------------------------

--------

>LLGY28

------------------------------------------------------------

-------------------------V----------TEPT------PW------------

-----------------VSSMLVVVKP------D---K--------------------LR

ICIDPR-D-------LN-------RAICREHYQ-MPT-----------IEEV-ATR---L

T-NA------------KKF-TVLDAKDGFWQKRL---DTESSYKTTF-------------

----NTP-FG------------------RFRWNRMPFGISSAPEVWQRT-----MHE---

-------FVED--L----------------------------DGVEVIADD-FLIAGF-G

KTEDEVLRS---LEANERAFFEKCRRWNLKLN--------------RRKVKR--------

---------------CQSSVRFMGHLL---------------------------------

----TSDGLKADPEKIQAIIEMSEPGDIKA--LKRFLGMVN---YL----------SKYM

PRLS----

>LLGY30

------------------------------------------------------------

-----------------------EEV---------VDVPT------PW------------

-----------------VSPIVAQPKP------K-KPN------------------E-LR

ICVDMR-E-------AN-------RAIRRERHV-TPT-----------VDDV-IFE---L

N-GS------------SYF-TKLDLNKGYHQLEL---APESRYITTF-------------

----SAN-QK------------------LWRYKRLMFGLSSAAEVFQNA-----IQT---

-------TLQG--I----------------------------PKAFNISDD-ILVHGR-T

Q------AE---HDDNLRQVFEQVRATNLTLN--------------REKCVF--------

---------------NQRHLSFFGHVW---------------------------------

----SPEGVSADPQKLEAIRKMKTPENAEE--VRSLLGMAG---YV----------SRSI

PNFA----

>LLGY31

------------------------------------------------------------

----------------MVKMKVITRV----------TEPT------DW------------

-----------------VSSIVYSRKS------S---G------------------K-LR

ICLDPK-D-------LN-------EAVKRPHYK-TPT-----------LDEV-TYK---L

A-GA------------KVF-SKLDARHGYWSISL---DDESSRKTTF-------------

----NSP-FG------------------RFRFERLPFGLNLSQDVFQER-----MDN---

-------ILEQ--C----------------------------PGTMGMADD-VAVFGR-D

D------VE---HDRNLHNVMKVARKHGLVFN--------------VDKCDI--------

---------------HQPSIHFFGLVF---------------------------------

----DVTGVRPDPSKVNAIKRLETPRDTTQ--LQEFLGVAT---YM----------SPFI

PHLS----

>LLGY32

------------------------------------------------------------

------------------------------------------------------------

------------------------------------------------------------

------------------------------------------------------------

--QG------------KLK-TVFDAWNGYHSVPL---HPDDRHLTTF-------------

----ITP-WG------------------RYRYCVTPQGYIASGDGYSRR-----YDE---

-------IVAD--I----------------------------PQKTKCIDD-TLLWAD-T

M------EE---SFFQAIHWLDVCGKNGITLN--------------PDKFTF--------

---------------CRPEVEFAGFVI---------------------------------

----TLDNVRPCGKYLQAIRDFPTPRNITD--VRSWFGLVN-------------------

--------

>LLGY35

------------------------------------------------------------

-----------------EEAKVIRRV----------TEPT------DW------------

-----------------VSSLTFTRKR------D---G------------------G-LR

MCLDPK-D-------LN-------RAIKRCHHK-TPT-----------LEEI-THQ---F

T-GS------------KFF-LKFDAKNGYWSVTL---DNESSLLTTF-------------

----NSP-FG------------------RYCFLRMSFGLVMAQDVFQQK-----MDA---

-------ILES--C----------------------------PDTLGIADN-VAVFGR-S

E------AE---HDANLHNLMRVSREHGLVFN--------------SKKCAI--------

---------------KSPQISFFGTIY---------------------------------

----DEKGVHPDPKKVEDIQMIPTPESKTE--LQEFLGIVT---YM----------GSFV

PNLS----

>LLGY37

------------------------------------------------------------

------------------------------------------------------------

----------------------------------------------------------VH

V-----------------------------------------------------------

------------------------------------------------------------

------------------------------------------------------------

--------------------------------------------ILCQMDD-VLVFGA-T

R------PQ---HDQRLHEVLSRLQQAKVTLN--------------TKKCQF--------

---------------SVQHVTFLGQMI---------------------------------

----DASGIHPDKEKIRAILDMPQPVDVSG--VRRFLGMVN---QL----------GKFT

PHLA----

>LLGY38

------------------------------------------------------------

-----------------LDDDIICP------------SNS------AY------------

-----------------SSPILLVPKK------D---G------------------E-SR

LCVDYR-K-------LNE------VTQ-KDAYP-LPH-----------IQEI-FDL---V

G-GS------------TIY-STLDLKAGYYQMPV---AEEDKHKTAF-------------

----RCH-LG------------------HYAFNKVPFGLKSAPNFFQRE-----MNK---

-------ILAD--L--------------------------IGKCVFVYIDD-ILVYSK-T

E------HD---HIKHLQLVFDKLRDAGLKLK--------------PTKCAF--------

---------------GLPEVKLLGYVL---------------------------------

----NADGITTDPDKVYVIAKLKPPTTVKE--VRSMLGMCN---YY----------RNSL

PNYA----

>LLGY39

------------------------------------------------------------

------------------------------------------------------------

--------------------------K------T---S----------------------

------------------------------------------------------------

-------------------------RH---------------------------------

---------G------------------RFRWKRMPFGISCAPEVFQRK-----IHE---

-------LIEG--I----------------------------AGVEVVADD-FAVIGC-G

STMEAATKD---HDDNLLRFLGHCDQINVKLN--------------TEKLQL--------

---------------RKTEAPFIGHVA---------------------------------

----SGDGLKIHRDKVRVIVEMPEPEDVTA--VQRLIGMVT---YL----------TKFV

PRLT----

>LLGY40

------------------------------------LK----------------------

---------------RMEALGVISKV----------EQPT------DW------------

-----------------CAPMVVVPKS------Q---D------------------D-VR

ICVDLT-K-------LN-------ESVRRERYE-MPS-----------VDYT-LGQ---L

A-GA------------KIF-SKLDANSGFWQVPL---SEESTLLTTF-------------

----ITP-FG------------------RFAFRRLPFGISSAPEHYQRR-----MSA---

-------ILEG--I----------------------------PGVLCQMDD-VLVFGA-T

Q------PQ---HDQRLHEVLSRLQQAKVTLN--------------TKKCQF--------

---------------SVQHVTFLGQMI---------------------------------

----DASGIHPDREKIRAILDMPEPVDVSG--VRRFLGMVN---QL----------GKFT

PHLA----

>LLGY41

------------------------------------LD----------------------

---------------RLVQTNVMEPV-----------RYS------DW------------

-----------------ATPIVPVLKA------D---G------------------K-VR

VCGDYK-LT------VN-------RVSHLEQYP-IPT-----------LDDL-CEK---L

T-GG------------KQF-SKLDLSHAYSQLPL---DNKSKEYATV-------------

----NAH-RG------------------LFRYNRLPYGISSAPAIFQRT-----MEN---

-------I----------------------------------------LQD-IL------

------------------------------------------------------------

------------------------------------------------------------

------------------------------------------------------------

--------

>LLGY43

------------------------------------------------------------

----------------------IRKV----------KEHT------DW------------

-----------------CSSIVYSTKK------D---G------------------S-LR

ICLDPK-R-------LN-------EAIKRCPHK-TPT-----------LEEI-NPA---F

V-DA------------K-F-SKLDAKSGYWSVQL---DEQSQLLTTF-------------

----RTP-IG------------------RYCYQRLPFGLCVSQDIFQQR-----MDE---

-------ILEG--L----------------------------DGCVGIADD-ICVFGA-T

Q------EE---HDERLVALLEVANSSGLVFN--------------SAKCMI--------

---------------KQKSKSFFGNIY---------------------------------

----SAEGVSPDPSKVQDIHEMPV------------------------------------

--------

>LLGY44

------------------------------------------------------------

------------------------------------------------------------

------------------------------------------------------------

-------------------------------------------------ADV-QSQ---L

G-DK------------QIF-TVIDMKDGYWHVKL---TEESSHVCTF-------------

----HTP-WR------------------RKRFKCMPFGINSASEVMQKR-----NEE---

-------TFAD--I----------------------------PGVRIIADD-MIISAK-D

E------TE---HDAIVRKVMQRARERNVKFN--------------KTKVQF--------

---------------KVPNVTYMGHIV---------------------------------

----AADGLKPDPAKVETIVMMPKPENKSD--LQRLLGMVR---YL----------AQYI

PNES----

>LLGY45

------------------------------------------------------------

----------------MVKMKVITRV----------TEPT------DW------------

-----------------VSSIVYSRKS------S---G------------------K-LR

ICLDPK-D-------LN-------EAVKRPHYK-TPT-----------LDEV-TYK---L

A-GA------------KVF-SKLDARHGYWSISL---DDESSRKTTF-------------

----NSP-FG------------------RFRFERLPFGLNLSQDVFQER-----MDN---

-------ILEQ--C----------------------------PGTMGMADD-VAVFGR-D

D------VE---HDRNLHNVMKVARKHGLVFN--------------VDKCDI--------

---------------HQPSIHFFGLVF---------------------------------

----DVTGVRPDPSKVNAIKRLETPRDTTQ--LQEFLGVAT---YM----------SPFI

PHLS----

>LLGY46

------------------------------------LD----------------------

---------------RLVQTNVMEPV-----------RYS------DW------------

-----------------ATQIVPVLKA------D---G------------------K-VR

VCGDYK-LT------VN-------RVSHLEQYP-IPT-----------LDDL-C------

------------------------------------------------------------

------------------------------------------------------------

------------------------------------------------------------

------------------------------------------------------------

------------------------------------------------------------

------------------------------------------------------------

--------

>LLGY47

------------------------------------------------------------

------------------------------------------------------------

------------------------------------------------------------

------------------------------------------------------------

-------------------------------------------LSTI-------------

----TTL-KG------------------LFQYTRLCYLASSAPGIFQRA-----MEQ---

-------IVQG--I----------------------------LMVAVYLDD-ILVSGR-T

L------EK---ARANLLTVLIRLQAAGLRLR--------------IAKCSF--------

---------------MQEYCVYLGHRL---------------------------------

----DAEGIHPTNEKLFTLQNAPEPKSVSE--LRSYLGMVK---YY----------HKFL

KNLS----

>LLGY48

------------------------------------------------------------

------------------------------------------------------------

------------------------------------------------------------

-AFMHR-Q-------LNL------KTI-KDAFP-LPR-----------VDEC-LEA---L

T-GA------------KYF-STLDLAHGYYQCAI---DARDVPKTTFR------------

----VGN-SG------------------LYEFTRMPMGLCNAPATFSRL-----MDH---

-------VLGN--EN-------------------------FH-SLLIYLDD-VLVFGK-S

V------DE---MLQRLDLVFSKLRAFGLKIK--------------PQKCSL--------

---------------FRQEVKFLGHIV---------------------------------

----SAEGVATDPDKIKAVQEWQEPKSESD--LRSFLGLAG---YY----------RRYV

PSFA----

>LLGY49

------------------------------------------------------------

-----------------------EAV----------EKPT------AW------------

-----------------ISSMVVITKK------D---S------------------K-LR

ICLDPK-D-------LN-------RAIRRENYQ-LPT-----------IEDI-ATR---L

H-GA------------KVF-TVLDVRHGFWHVRL---DDRSSYLTTF-------------

----HTP-FG------------------RYRFKRMPFGISSAPEVFPKK-----MHE---

-------LIEG--L----------------------------QGIEVVADD-FVVVGY-G

NTVDEANVD---HDKRLHSFLQRCEERGVKLN--------------VDKFKL--------

---------------RQEEVRFIGHVA---------------------------------

----TSDGLSIDPTKVKAIVDMPNPTDVAG--VQRLLGLAQ---YL----------AKFL

PHLS----

>LLGY51

------------------------------------LK----------------------

---------------RMEALGVISKV----------EQPT------DW------------

-----------------CAPMVVVPKS------Q---D------------------D-VR

ICVDLT-K-------LN-------ESVRRERYE-MPS-----------VDYT-LGQ---L

A-GA------------KIF-SKLDANSGFWQVPL---SEESTLLTTF-------------

----ITP-FG------------------RFAFRRLPFGISSAPEHYQRR-----MSA---

-------ILEG--I----------------------------PGVLCQMDD-VLVFGA-T

Q------PQ---HDQRLHEVLSRLQQAKVTLN--------------TKKCQF--------

---------------SVQHVTFLGQMI---------------------------------

----DASGIHPDREKIRAILDMPEPVDVSG--VRRFLGMVN---QL----------GKFT

PHLA----

>LLGY52

------------------------------------------------------------

-----------------EEMGIIRR------------SDS------PW------------

-----------------ASPLHMVPKN------S---G------------------G-WR

PCGDYR-R-------LND------VTI-ADRYP-VPH-----------IQDF-SSQ---L

A-GA------------TMF-SKIDLVRGYHQIPV---ATDDISKTAV-------------

----ITP-FG------------------LFEFLRTPFGLKNAAQAFQRL-----MDT---

-------VCSG--L----------------------------EFVFVYLDD-ILVSSA-S

A------EQ---HKHHLRTVFHRLASHGLVIN--------------VSKCKF--------

---------------GTTTIDYLGHRI---------------------------------

----TSQGAVPLPAKVEAIRMFTRPTTVKG--LQQFAGMVN---FY----------HRFV

PNAA----

>LLGY53

------------------------------------ID----------------------

---------------RLESAGVLYRV-----------DNS------EW------------

-----------------ASPTVNVPKM------K---HGK---------------MS-VR

VCGDYK-L-------VN-------VTIEDDKYP-LPT-----------AQDL-FAN---L

A-HKG--------KKPTVF-SILDLSGAFNQLEV---DEKSSPLLTL-------------

----NTH-KG------------------LYRTRRLAYGVKTTPSVFQAT-----MDK---

-------ILAG--I----------------------------KNVMCFVDD-ILVTGN-T

E------QE---HLKTLEQVLQMLDQYNVRLN--------------KAKCQF--------

---------------MKSQVQYLGHTV---------------------------------

----NANGIQPIQDKVEAIRKAPPPTNITE--LQSFLGVVQ---YY----------AKFV

PNLS----

>LLGY54

------------------------------------------------------------

-----------------LHDEIIRP------------SNS------AY------------

-----------------SSPILLVPKK------D---G------------------D-SR

LCVDYR-K-------LNA------VTE-QDAYP-LPI-----------IQDI-FDL---V

G-GS------------SIY-STLDLKSSYWQMPV---AEADIHKTAF-------------

----RCH-AG------------------HFEFTKVPFGLKSAPNFFQKE-----MNT---

-------ILAD--L--------------------------IGKCVFVYIDD-ILVFSK-N

E------TD---HIRHLQLVFDRLRNAGLKLK--------------PTKCAF--------

---------------GLPEVKLLGYVL---------------------------------

----NADGIKTDPDKVAVIANLLPPTTVKE--TRSFLGMCN---YY----------RNSL

PNYA----

>LLGY55

------------------------------------LK----------------------

---------------RMEALGVISKV----------EQPT------DW------------

-----------------CAPMVVVPKS------Q---D------------------D-VR

ICVDLT-K-------LN-------ESVRRERYE-MPS-----------VDYT-LGQ---L

A-GA------------KIF-SKLDANSGFWQVPL---SEESTLLTTF-------------

----ITP-FG------------------RFAFRRLPFGISSAPEHYQRR-----MSA---

-------ILEG--I----------------------------PGVLCQMDD-VLVFGA-T

Q------PQ---HDQRLHEVLSRLQQAKVTLN--------------TKKCQF--------

---------------SVQHVTFLGQMI---------------------------------

----DASGIHPDREKIRAILDMPEPVDVSG--VRRFLGMVN---QL----------GKFT

PHLA----

>LLGY56

------------------------------------LD----------------------

---------------RLQSDNIIEPV-----------TYS------QW------------

-----------------ASPIVPILKS------D---G------------------T-VR

ICGDYK-VT------VN-------RFAHIKQYP-LPT-----------SEDL-FAT---L

A-GG------------VMF-SKLDMAHAYQQILI---DDDCKQYLMI-------------

----NTH-RG------------------LFVYNRLAFGVSSAPAIFQRV-----IEN---

-------LLAG--V----------------------------PHTVVYLDD-ILVTGA-F

E------EE---HQANLKEVLRRLAEAGLRLK--------------KDKCNF--------

---------------SVDAVEYLGHRI---------------------------------

----SKDGLATLDKRVRAVVEAPTPTDVTQ--VKSFLGMLT---FY----------LRFL

PNLA----

>LLGY57

------------------------------------LD----------------------

---------------RLQKEKTISPV-----------EYS------QW------------

-----------------ASGVVPVAKR------E---T------------------D-VR

LCGHYK-VT------IN-------P------------------------ENS-FAK---M

A-GG------------KRF-GKLYIKNVYLQMKV---EEDSKQYFTI-------------

----NTH-KG------------------LFEYNRLPFGIKTAPSIWQRA-----MEQ---

-------TLQG--I----------------------------LGVEVMLDD-IIVTGK-S

D------AE---HLENLEAVLRRLAER---------------------------------

------------------------------------------------------------

------------------------------------------------------------

--------

>LLGY58

------------------------------------------------------------

------------------------------------------------------------

------------------------------------------------------------

------------------------------------------------------------

----------------KKK-TVFDAWNGYHSVPI---RECDRHLTTF-------------

----ITP-WG------------------RYRYCTTPQGYIASGDGYTRR-----FDE---

-------IVAD--F----------------------------PNKTKCIDD-TCIWGD-T

I------EE---CFVQACAWLDQCGRNGITLN--------------PEKFRF--------

---------------AQDIVEFAGFTI---------------------------------

----TPDEVRPCSKYLDAIMQFPVPRNITD--VRSWFGLVNQVSY---------------

--------

>LLGY60

------------------------------------------------------------

-----------------EEMGIIRR------------SDS------PW------------

-----------------ASPLHMVPKN------S---G------------------G-WR

PCGDYR-R-------LND------ITV-PDKYP-VPH-----------IQDF-SSQ---L

A-GA------------TLF-SKIDLVRGYHQIPV---APTDISKTAV-------------

----ITP-FG------------------LFEFLRTPFGLKNAAQTFQRL-----MDT---

-------VCKG--L----------------------------TFVFVYLDD-ILVSSA-T

A------ED---HVSHLRTVFERLASHGLVIN--------------ESKCQF--------

---------------GTPTIDYLGHHI---------------------------------

----TREGAIPLPAKVDAIRTFERPTTVKG--LQQFAGMVN---FY----------HRFV

PNAA----

>LLGY61

------------------------------------------------------------

------------------------PV----------NEPT------KW------------

-----------------VSHMATVRKA------N---G------------------K-LR

LCIDPQ-P-------LN-------VALMREHYK-LPT-----------FDDV-LPK---L

H-NA------------KVF-SKIDIKEAYWHVKL---DEQSSKLTTM-------------

----ITP-SG------------------RYRWARLPFGLKVSSELFQKR-----LHH---

-------AIAD--L----------------------------NGVVCVADD-IIIVGC-G

TTQELADRD---HAKNLEILLERCRKCNIRVN--------------EEKMAL--------

---------------KQTEVEFLGHKI---------------------------------

----TRDGIEASQKKVQAIVEMPAPTDVTG--VRRLCGMVQ---YL----------ARYT

PNLA----

>LLGY63

------------------------------------LG----------------------

---------------KLEKAGVITPV-----------TGS------DW------------

-----------------ATGVVVVPKK------N---G------------------A-IR

LCGNYK-TT------VN-------PQLKTVSQP-NIN-----------IDDI-LAD---L

A-GG------------VKF-SKLGLTNAYNQMEV---SEESREYLTI-------------

----ATH-NG------------------LFQQNRLVFGTTTAPAIWQNA-----IEK---

-------VLQG--L----------------------------PGVKVYLDN-ILVSGR-T

E------SE---HLSNLGRVFERLT-FGLKLN--------------RDKCEF--------

---------------SRDSLEYLGHVI---------------------------------

----DAQGIHKS------------------------------------------------

--------

>LLGY64

-------------------------------------E----------------------

---------------RLENEGTFKPV-----------EFS------DW------------

-----------------ATPIVPIVKS------D---S------------------S-VR

ICGDYK-LT------VN-------KASRMDSYP-IPK-----------VDEL-FAK---L

A-GG-------------KY-SELDLSHAYEQILL---DESSCECVTI-------------

----NTH-RG------------------LFQYQRLPYGVSSSPGIFQRI-----MEC---

-------LFQG--M----------------------------TDVAPYLDN-VIITGK-D

D------EE---HLKNLALVLEKISQAGLCLK--------------RSKCQF--------

---------------MKEKIVALGHVL---------------------------------

----SGKGIQPCKAKVEAIQNAPAPTNVTE--LRAYLGLIN---YY----------HKYL

RNLS----

>LLGY65

------------------------------------------------------------

-----------------LNRGVIRE------------SHS------AW------------

-----------------ASPIVLVRKK------D---D------------------S-LR

MCVDYR-L-------LNA------KTH-RDAFP-LPR-----------IDES-FDA---M

S-GA------------NWF-TTLDLASGYHQIAM---SEDDREKTAF-------------

----TTP-MG------------------LYEFNRMPFGLCNSPATFQRL-----MQR---

-------CFGD--L--------------------------CYQTVLCYLDD-VIIFSK-T

F------DS---HLAQLEQVLQRLQRIGLKLK--------------TSKCHF--------

---------------LQREVLYLGHHV---------------------------------

----SADGIATDPAKIDAVKDWAVPNTVKQ--LRSFVGFAS---YY----------RKYV

QGFS----

>LLGY66

------------------------------------------------------------

------------------------------------------------------------

------------------------------------------------------------

------------------------------------------------------------

------------------------------------------------------------

------------------------------------------------------------

-------------------------------------------NVHIVADD-MLIAAK-T

E------AE---HDSTMRKVTERARGRGVKFN--------------MKKTQL--------

---------------KKSEVFYMGTMI---------------------------------

----SADGMRPDDAKIKAIVSMPEPTDKDG--VRRIIGMLN---YL----------LPFI

PNKA----

>LLGY70

------------------------------------ID----------------------

---------------RLESAGVLYRV-----------DNS------EW------------

-----------------ASPTVNVPKM------K---HGK---------------MS-VR

VCGDYK-L-------VN-------VTIEDDKYP-LPT-----------AQDL-FAN---L

A-HKG--------KKPTVF-SILDLSGAFNQLEV---DEKSSPLLTL-------------

----NTH-KG------------------LYRTRRLAYGVKTAPSVFQAT-----MDK---

-------ILAG--I----------------------------ENVMCFVDD-ILVTGN-T

E------QE---HLKTLEQVLQMLDQYNVRLN--------------KAKCQF--------

---------------MKSQVQYLGHTV---------------------------------

----NANGIQPIQDKVEAIRKAPPPTNITE--LQSFLGVVQ---YY----------AKFV

PNLS----

>LLGY71

------------------------------------------------------------

----------------------IRKV----------KEHT------DW------------

-----------------CSSIVYSTKK------D---G------------------S-LR

ICLDPK-R-------LN-------EAIKRCPHK-TPT-----------LEEI-NPA---F

V-GA------------KWF-SKLDAKSGYWSVQL---DEQSQLLTTF-------------

----RSP-IG------------------RYCYQRLPFGLCVSQDIFQQR-----MDE---

-------ILEG--L----------------------------DGCVGIADD-ICVFGA-T

Q------EE---HDERLVALLEVANSSGLVFN--------------SAKCMI--------

---------------KQKSKSFFGNIY---------------------------------

----SAEGVSPDPSKVQDIHEMPV------------------------------------

--------

>LLGY72

------------------------------------------------------------

-------------------QQIIRTI----------EEPT------DW------------

-----------------VSSLTYVTKR------D---G------------------S-IR

VCLDPR-Q-------LN-------KALIRPRDE-APT-----------LDEL-NHK---F

A-NA-------------FF-SKLDTKAGYWSIKL---DDESQKLTTF-------------

----QTP-FG------------------RYCFLRLPFGLSVSQDIFQLE-----MDR---

-------ILEK--C----------------------------NGVCGIADD-FVVYGT-T

E------IE---HDRNLLQFMDIAKQHGLALN--------------SAKCDI--------

---------------KCNKVSFFGQLY---------------------------------

----TSEGIKPDPQKVNDLRAMPVPTTKAE--LQQFLGFIT---YL----------SRFM

KAFS----

>LLGY73

------------------------------------------------------------

------------------------------------------------------------

------------------------------------------------------------

----------------------------SEYYP-MPN-----------IEEV-STR---L

K-NA------------RLF-TVLDDKNGLWQIPL---DKTSSMLTCF-------------

----NTP-FG------------------RYRWLWTPFGINSA------------------

------------------------------------------------------------

------------------------------------------------------------

------------------------------------------------------------

------------------------------------------------------------

--------

>LLGY74

------------------------------------------------------------

------------------------------------------------------------

------------------------------------------------------------

------------------------------------------------------------

----------------SWK-TVTDAWNGYHSVPL---RDSDRHLTTF-------------

----ITP-FG------------------RWRYTRAPQGFLSSGDGYNRR-----FDE---

-------IIAN--F----------------------------QRKERCIDD-TVHWDV-E

L------QN---HWWRTIDYLILVGRAGVVLN--------------PDKFQF--------

---------------AQRTVDFAGFRI---------------------------------

----SNATIEPLPKYLDAIRDFPTPTSTTD--VRSWFGLVNQVTNY--------------

--------

>LLGY75

------------------------------------------------------------

------------------QDKVIKRV----------TEPT------EW------------

-----------------VSSLAFSRKK------D---G------------------S-LR

ICLDPK-D-------LN-------RAIRRCHHK-TPT-----------LEEI-THK---F

T-GA------------KHF-SKLDAKNGYWSVKL---DQESSLLTTF-------------

----NSP-FG------------------RYCFTRMPFGLVMSQDVFQQK-----MDA---

-------ILDN--C----------------------------PGTVGIADD-VAVFGR-T

E------AE---HDANLRNLMSVAREYGLMFN--------------SKKCVV--------

---------------KASQISFFGILY---------------------------------

----DDKGAHPDPKKVEDIQMIPAPTNRTE--LQEFLGIVT---YM----------GPFV

PNLS----

>LLGY76

------------------------------------------------------------

---------------NMERLGVISKV----------TEPT------DW------------

-----------------VSSLVYSRKS------N---N------------------K-LR

ICLDPK-D-------LN-------TAIKRPHYK-TPT-----------LDEL-THK---L

A-GA------------TVF-SKLDARHGYWSVSL---DEPSSFLTTF-------------

----NSP-FG------------------RYRFQRLPFGLNLSQDVFQER-----MDH---

-------ILEN--C----------------------------EGTIGIADD-VAVFGK-N

E------AD---HDANLHNLMKTARQHGLVFN--------------ADKCHI--------

---------------KQPRMQFFGLIF---------------------------------

----DTDGVHPDPGKITAIKQLQAPQDATQ--LKEFLGIAT---YM----------SPFT

PNLS----

>LLGY77

------------------------------------------------------------

-------------------KDNIRP------------STS------PY------------

-----------------SSPVVLVRKK------S---G------------------E-LR

MCVDYR-G-------LNA------KTI-KDAYP-LPR-----------IEES-LDA---L

N-GA------------VLF-STMDLQSAW-------------------------------

------------------------------------------------------------

------------------------------------------------------------

------------------------------------------------------------

------------------------------------------------------------

---------------------W--------------------------------------

--------

>LLGY79

------------------------------------------------------------

-----------------LEMDIIRP------------SNS------PW------------

-----------------SSPLHMVAKP------S---G------------------G-WR

ACGDYR-A-------LNA------VSE-DDRYP-IPH-----------MQDF-AGQ---L

E-GK------------SIF-SKIDLVRAYNQVPM---SATDIAKTAI-------------

----ITP-FG------------------LFEYTRMPFGLKNAAQTFQRF-----MDH---

-------VFRD--F----------------------------PFAYVYLDD-ILVASA-S

A------DE---HRIHLRQLFSRLADYGLVVN--------------PQKCVL--------

---------------GQPSLEFLGHCV---------------------------------

----TAFGVRPLLERVKHITDFPRPSSTKS--LKEFLGMLN---FY----------RRFV

PHAA----

>LLGY80

------------------------------------------------------------

-----------------------EAV----------EKPT------AW------------

-----------------ISSMVVITKK------D---S------------------K-LR

ICLDPK-D-------LN-------RAIRRENYQ-LPT-----------IEDI-ATR---L

H-GA------------KVF-TVLDVRHGFWHVRL---DDRSSYLTTF-------------

----HTP-FG------------------RYRFKRMPFGISSAPEVFPKK-----MHE---

-------LIEG--L----------------------------QGIEVVADD-FVVVGY-G

NTVDEANVD---HDKRLHSFLQRCEERGVKLN--------------VDKFKL--------

---------------RQEEVRFIGHVA---------------------------------

----TSDGLSIDPTKVKAIVDMPNPTDVAG--VQRLLGLAQ---YL----------AKFL

PHLS----

>LLGY81

------------------------------------------------------------

-----------------LKADIIEP------------SIS------EY------------

-----------------ASSHVVVRKP------D---G------------------S-VR

YCIDFC-R-------LNA------KTV-VDAEP-IPN------------QEVILNK---M

G-------DD------DFI-SRIDLTKGFWQVPI---REEDRKYTAF-------------

----PMD-QG------------------LMQNKYMPFGLMNALAIFCRM-----VRK---

-------LLHD--V----------------------------PNVDSYVDD-IVPHTV-T

W------DD---HVSAIRQVLERLR-HGLTAK--------------PSKCEI--------

---------------GHAELDLLGHVL---------------------------------

----GGGSTKPD-KKLEKILGTRRPETKKE--LRSFLGTIG---YY----------QKFI

DNYS----

>LLGY82

------------------------------------LQ----------------------

---------------RLEDLKIVTKV-----------DYS------DW------------

-----------------ATPIVVVQKP------S---G------------------K-VR

ICGDYR-AT------VN-------PCLHVQQHP-IPR-----------IEEL-FAK---L

Q-GG------------MHF-SKLDMRDAYLQIEL---DDETKQLLVI-------------

----NTH-KG------------------LYRYNRLCFGPSPAPAIFQKL-----VDN---

-------LVAG--I----------------------------PGVAAYLDD-IIVTGQ-T

K------AE---HLENLRRVFAALDNYGLKLQ--------------LDKCVF--------

---------------FAPEVSYLGYII---------------------------------

----SKDGLCASEERVQAILQYATPTDLKQ--LESFVGKLN---YY----------GKFL

PAFA----

>LLGY83

------------------------------------------------------------

---------------NMERLGVISKV----------TEPT------DW------------

-----------------VSSLVYSRKS------N---N------------------K-LR

ICLDPK-D-------LN-------TAIKRPHYK-TPT-----------LDEL-THK---L

A-GA------------TVF-SKLDARHGYWSVSL---DEPSSFLTTF-------------

----NSP-FG------------------RYRFQRLPFGLNLSQDVFQER-----MDH---

-------ILEN--C----------------------------EGTIGIADD-VAVFGK-N

E------AD---HDANLHNLMKTARQHGLVFN--------------ADKCHI--------

---------------KQPRMQFFGLIF---------------------------------

----DTDGVHPDPGKITAIKQLQAPQDATQ--LKEFLGIAT---YM----------SPFT

PNLS----

>LLGY84

------------------------------------------------------------

---------------NMERLGVISKV----------TEPT------DW------------

-----------------VSSLVYSRKS------N---N------------------K-LR

ICLDPK-D-------LN-------TAIKRPHYK-TPT-----------LDEL-THK---L

A-GA------------TVF-SKLDARHGYWSVSL---DEPSSFLTTF-------------

----NSP-FG------------------RYRFQRLPFGLNLSQDVFQER-----MDH---

-------ILEN--C----------------------------EGTIGIADD-VAVFGK-N

E------AD---HDANLHNLMKTARQHGLVFN--------------ADKCHI--------

---------------KQPRMQFFGLIF---------------------------------

----DTDGVHPDPGKITAIKQLQAPQDATQ--LKEFLGIAT---YM----------SPFT

PNLS----

>LLGY85

------------------------------------------------------------

------------------------------------------------------------

------------------------------------------------------------

------------------------------------------------------------

----------------KKK-TVFDAWNGYHSVPI---RECDRHLTTF-------------

----ITP-WG------------------RYRYCTTPQGYIASGDGYTRR-----FDE---

-------IVAD--F----------------------------PNKTKCIDD-TCIWGD-T

I------EE---CFVQACAWLDQCGRNGITLN--------------PEKFQF--------

---------------AQDIVEFAGFTI---------------------------------

----TPDDVRPCSKYLDAIMQFPVPRNITD--VRSWFGLVNQVSY---------------

--------

>LLGY86

------------------------------------ID----------------------

---------------RLLAEDIIEPV-----------QYS------DW------------

-----------------AAPVVPVMKA------D---K------------------S-VR

LCGDYK-LT------VN-------QVAKLDRYP-IPR-----------IEDL-YAQ---L

G-NG------------TTY-TKLDMRHAYEQIQL---HPDSRKYVTI-------------

----NTP-RG------------------LFTYKRLPYGVSSAPGIFQRV-----MDS---

-------LLKG--I----------------------------PNTMVYLDD-VLVTGP-T

E------DE---HLQTLDRVLERLVQAGFRLK--------------ESKCQF--------

---------------LSDEVDYLGHRI---------------------------------

----DAEGIHPSGETLSAVRDAPAPTNITE--LRSYLGMVN---HY----------GRFL

PNLA----

>LLGY87

------------------------------------------------------------

---------------------IIEEV---------SNTPT------TW------------

-----------------LSPLVVVPKP------D---G------------------D-VR

ICVDVR-R-------AN-------EAIVRERHP-IPT-----------IEEV-LQD---L

S-GS------------TVF-SKLDLKWGFHQVEL---AEESREITTF-------------

----VT--HR------------------LYRYRRLMFGIASAPENYQKI-----VKD---

-------VLRD--C----------------------------KGAANIADD-VIVHGR-G

V------KE---HDENLFAMLNRLKECGLTLN--------------VGKCKF--------

---------------RLPRLTFYGHDL---------------------------------

----GKQGITPSEEKIDAIVNAQSPNNASE--VRSFLGLVQ---YS----------S---

--------

>LLGY88

------------------------------------------------------------

------------------------------------------------------------

------------------------------------------------------------

------------------------------------------------------------

------------------------------------------------------------

------------------------------------------------------------

------------------------------------------------------------

-----------------EDIMRRMQEVGLCLN--------------LDKCIV--------

---------------KSRRIKFFGNYL---------------------------------

----SSNGLEPDLGKIATTIDMSQPTSAQK--LQSFLGMVN---YL----------G---

--------

>LLGY89

------------------------------------------------------------

------------------------------------------------------------

------------------------------------------------------------

------------------------------------------------------------

------------------------------------------------------------

------------------------------RFTRMPFGISSASKVMQKR-----NED---

-------TFAD--I----------------------------PGVRIIAGD-MIISAK-D

E------TE---HNAIVRKVMQRAHERNVKFN--------------KTKVQF--------

---------------KVPNVTYMGHVV---------------------------------

----AADGLKPDPAKVEAIVMMPKPENKSD--LQRLLGMVR---YL----------AQYI

PNES----

>LLGY91

------------------------------------------------------------

------------------------------------------------------------

--------------------------A------E---G------------------S-LR

ICLDPR-P-------LN-------RAIKRERYE-IPT-----------PADG-QSQ---L

G-DN------------QIF-TVIDMKDGYWHVKL---TEESSHMCTF-------------

----HTP-----------------------------------------------------

------------------------------------------------------------

------------------------------------------------------------

------------------------------------------------------------

------------------------------------------------------------

--------

>LLGY92

------------------------------------------------------------

------------------------------------------------------------

------------------------------------------------------------

-C----------------------------------------------------------

------------------------------QLVL---YPSSRHITTF-------------

----STH-VG------------------LYRYKRLSFGINAAAEVFQHE-----IQT---

-------VIQG--V----------------------------SGAINISDD-IAVFGV-D

Q------CS---HDKALDDVLHKLQAAGLTAN--------------LEKCEF--------

---------------RKKKIEFFGLIF---------------------------------

----SGDGVSPDPKKVADLHRAAEPKNASE--VRTFLGMAQ---YS----------ARYI

KDFA----

>LLGY93

------------------------------------------------------------

------------------------------------------------------------

------------------------------------------------------------

-----------------------------------------------------------L

G-KA------------SVF-SKLDANSGFWQLPL---DEESKLLTTF-------------

----ITP-QG------------------RFAFNRIAFGISSAPEIFTRT-----ICV---

-------ILQG--L----------------------------DGVICHMDD-ILMFAS-N

E------AE---YDDRLRKVLQRLQGAGLTLN---------------EKCEF--------

---------------GKKSIRFLGHDI---------------------------------

----DGTGVHADEQKLEAMQKFPAPTNTTE--LRRFTGMVN---QL----------GKFV

PNLA----

>LLGY95

------------------------------------------------------------

-----------------VEQKVIAPV----------DQPT------PW------------

-----------------VSNVLVTTKK------T---G------------------E-LR

VCIDPR-P-------LN-------KALKREHYQ-LPV-----------LDDI-LPE---L

T-TA------------KVF-SSIDLKSGYWHVIL---DEESSMLTTF-------------

----STP-SG------------------RYRWLRLPFGISVSSEIFQKK-----LQQ---

-------AIGD--L----------------------------QGVMCIADD-IVLYGV-G

NDHDEAMRD---HDEKLRMLLQRCRDVGIRIN--------------KDKLKL--------

---------------RKEEISFHGHLV---------------------------------

----TNMGLKLDPAKVEALQKMPPPTDVHG--VQRLGGFVN---YL----------ARFL

PRLS----

>LLGY96

------------------------------------------------------------

------------------------------------DGPT------PW------------

-----------------VSPIVVVPKK------T---G------------------A-VR

ICVDMR-E-------AN-------KAVKREKHL-MPT-----------IDDL-ITD---L

N-GA------------TMF-STLDLRAGYHQLEL---DPESRQITTF-------------

----STH-VA------------------LYRYKRLMFGINAASEIFQNT-----IAE---

-------LLHG--L----------------------------NGCRNISDD-IIVHGK-T

P------AE---HNANLRAVLDRLRENNVRLN--------------REKCKF--------

---------------SQPTVTFYGHVF---------------------------------

----GAHGLRADPKKLESISKARRPTNPSE--VRSLLGMAQ---YV----------SRFI

ADF-----

>LLGY97

------------------------------------------------------------

-----------------KEMGIIR-------------SDS------PW------------

-----------------ASPLHMVPKN------S---G------------------G-WR

PWGDYR-R-------LND------VTI-ADRYP-VPH-----------IQDF-SSQ---L

A-GA------------TMF-SKIYLVRGYHQIPV---ATDDISKTAV-------------

----IMP-FG------------------LFEFLRTPFGLENAA-AFQRL-----MDT---

-------VCSG--L----------------------------EFVFVYHDD-ILVSSA-S

A------EQ---HKHHLRTVFYCLASHGLVIN--------------VSKCKF--------

---------------GTTTIDYLGHRI---------------------------------

----TSQGAVPLPAKVEAIRMFTRPTTVKG--LQQFAGMVN---FY----------HRFV

PNAA----

>LLGY98

------------------------------------------------------------

-----------------VSQNVIRKV----------DEHT------DW------------

-----------------CSSLTFTTKK------D---G------------------S-IR

ICLDPK-R-------LN-------DSLKRCPHK-IPT-----------VEEL-NPE---F

A-HA------------TVF-SKLDAKAGYWAIHL---DEDSQLLTTF-------------

----RTP-FG------------------RYCWQRLPFGLSTSQDIFQAR-----MDE---

-------IMEG--L----------------------------NGVVSIADD-VCVHGR-N

A------AD---HDANLINLMNRAAEKGLVFN--------------SDKCFI--------

---------------KQESISFYGNTY---------------------------------

----TAEGIKPDPSKVRDIQNMPAPQCKED--LQRFLGMMT---YL----------SQYI

PRFA----

>LLGY99

------------------------------------------------------------

----------------MVKMKVITRV----------TEPT------DW------------

-----------------VSSIVYSRKS------S---G------------------K-LR

ICLDPK-D-------LN-------EAVKRPHYK-TPT-----------LDEV-TYK---L

A-GA------------KVF-SKLDARHGYWSISL---DDESSRKTTF-------------

----NSP-FG------------------RFRFERLPFGLNLSQDVFQER-----MDN---

-------ILEQ--C----------------------------PGTMGMADD-VAVFGR-D

D------VE---HDRNLHNVMKVARKHGLVFN--------------VDKCDI--------

---------------HQPSIHFFGLVF---------------------------------

----DVTGVRPDPSKVNAIKRLETPRDTTQ--LQEFLGVAT---YM----------SPFI

PHLS----

>LLGY100

------------------------------------------------------------

------------------------------------------------------------

------------------------------------------------------------

------------------------------------------------------------

----------------SWK-TVTDAWNGYHSVPL---RDSDRHLTTF-------------

----ITP-FG------------------RWRYTRAPQGFLSSGDGYNRR-----FDE---

-------IIAN--F----------------------------QRKERCIDD-TVHWDV-E

L------QN---HWWRTIDYLILVGRAGVVLN--------------PDKFQF--------

---------------AQRTVDFAGFRI---------------------------------

----SNATIEPLPKYLDAIRDFPTPTSTTD--VRSWFGLVNQVTNY--------------

--------

>LLGY101

------------------------------------------------------------

-----------------------EEV---------VDVPT------PW------------

-----------------VSPIVAQPKP------K-KPN------------------E-LR

ICVDMR-E-------AN-------RAIRRERHV-TPT-----------VDDV-IFE---L

N-GS------------SYF-TKLDLNKGYHQLEL---APESRYITTF-------------

----SAN-QK------------------LWRYKRLMFGLSSAAEVFQNA-----IQT---

-------TLQG--I----------------------------PKAFNISDD-ILVHGR-T

Q------AE---HDDNLRQVFEQVRATNLTLN--------------REKCVF--------

---------------NQRHLSFFGHVW---------------------------------

----SPEGVSADPQKLEAIRKMKTPENAEE--VRSLLGMAG---YV----------SRSI

PNFA----

>LLGY102

------------------------------------------------------------

------------------------KV----------EHPT------QW------------

-----------------ISSLQPVRKP------N---G------------------K-IR

LCIDPH-N-------LN-------KAIRRNHFP-MPT-----------LDDV-LPE---L

A-QA------------KIF-SLCDAKDGFLQVKL---SERSSDLTCF-------------

----WTP-FG------------------RYKWKRMPFGITSAPEEFQRR-----LSN---

-------ALAG--L----------------------------PGVTIVADD-ILIYGK-G

DTMKEARED---HDNNLKQLLERSCQINLKLN--------------LSKCRC--------

------------------------------------------------------------

------------------------------------------------------------

--------

>LLGY104

------------------------------------------------------------

------------------------------------DEPT------DW------------

-----------------VHNLVITEKR------N---G------------------S-LR

ICLDPR-P-------LN-------KAIKRERYE-IPT-----------PADV-QSQ---L

G-DK------------QIF-TVIDMKDGYWHVKL---TEKSSHLCTF-------------

----HTP-WG------------------RKRFKRMPFGIGSASEVKQKR-----NEE---

-------TFAD--I----------------------------PGVRIIADD-MIISAK-D

E------TE---HDAIVRKVMQM-------------------------------------

------------------------------------------------------------

------------------------------------------------------------

--------

>LLGY105

------------------------------------------------------------

-------------------------------------EPT------QW------------

-----------------VSSMIAVRKK------N---T------------------NKLR

ICLDPR-D-------LN-------KAIQRSHYP-LPT-----------LEDV-ATR---L

T-NA------------KVF-SVLDAKSGFWQVKL---EKDSSYLTTF-------------

----NTP-FG------------------RYRWLRMPFGINSAPEEWQRR-----AHE---

-------VVEG--L----------------------------KGVEVVADD-FLCIGF-G

DTIEEATRD---HDANLRALLERARTCHLVLN--------------PDKVQL--------

---------------RSKSVPYHS------------------------------------

------------------------------------------------------------

--------

>LLGY106

------------------------------------LK----------------------

---------------RMEALGVISKV----------EQPT------DW------------

-----------------CAPMVVVPKS------Q---D------------------D-VR

ICVDLT-K-------LN-------ESVRRERYE-MPS-----------VDYT-LGQ---L

A-GA------------KIF-SKLDANSGFWQVPL---SEESTLLTTF-------------

----ITP-FG------------------RFAFRRLPFGISSAPEHYQRR-----MSA---

-------ILEG--I----------------------------PGVLCQMDD-VLVFGA-T

Q------PQ---HDQRLHEVLSRLQQAKVTLN--------------TKKCQF--------

---------------SVQHVTFLGQMI---------------------------------

----DASGIHPDREKIRAILDMPEPVDVSG--VRRFLGMVN---QL----------GKFT

PHLA----

>LLGY107

------------------------------------------------------------

------------------------------------------------------------

------------------------------------------------------------

------------------------------------------------------------

--------------------TVCDAWNGYHAVPL---HPDDRQLTTF-------------

----ITP-WG------------------RYRYRSAPQGYVASGDGFTRR-----FDE---

-------IVAH--I----------------------------PNKTKCIED-TLLWAD-N

I------EQ---AFWQAVQWLDTCGRNGITQN--------------PDKFVF--------

---------------AKDTIEFAGFEI---------------------------------

----SPTSVKPCSKVLQAIKDFPIPRNVTD--IRSWFGLVN-------------------

--------

>LLGY108

------------------------------------LE----------------------

---------------RLENEGTIKPV-----------ELS------DW------------

-----------------ATPIVPIVKS------D---S------------------S-VR

ICGDYK-LT------VN-------KASRMDSYP-IPK-----------VDEL-FAK---L

A-GG------------QKY-SELDLSHAYEQILL---DESSCECVTI-------------

----NTH-RG------------------LFQYQRLPYGVSSSPGIFQRI-----MEC---

-------LFQG--M----------------------------TDVAPYLDN-VIITGK-D

D------EE---HLKNLALVLEKISQAGLRLK--------------RSKCQF--------

---------------MKEKMVALGHVL---------------------------------

----SGKGIQPCKAKVEAIQNAPAPTNVTE--LRAYLGLIN---YY----------HRYL

RNLS----

>LLGY110

------------------------------------------------------------

------------------EQKVIAKV----------DIPT------PW------------

-----------------ISNCLAIRKS------N---G------------------T-VR

VCIDPT-D-------LN-------KAIQRNHFP-LPT-----------IEEV-LPK---L

K-DA------------KIF-SLVDAKDGFLQVKL---SNESSYLTTF-------------

----WTP-CG------------------KYR-LRMAFGLTSSPEEFQRR-----LQL---

-------ALDG--L----------------------------DGIFIVADD-ILIIGR-G

ETDEEARRD---HDKNLDRLLQRAMEQNLKLN--------------KAKMRL--------

---------------HLTEIKYIGHVL---------------------------------

----SPEGVKADPEKMSDISSMATPTDSDQ--VRRFW-----------------------

--------

>LLGY111

------------------------------------------------------------

-----------------LSRGIIKE------------SHS------AW------------

-----------------ASPIVLVRKK------D---G------------------S-LR

MCVDYR-Q-------LNA------KTH-MDAFP-FPR-----------IDES-FDA---M

R-GA------------KWF-TTLDLAFGYHQIAM---EKKDQEKTAF-------------

----VTP-MG------------------IYEYTLMPFGLCNAPATFQRL-----MQR---

-------CLGD--Q--------------------------CYQTVLCYLDD-VIVFSE-T

F------DG---NIERLDMVLQRLQKIGLKLK--------------SSKCPF--------

---------------WQNEVIYLGHRV---------------------------------

----SADGISTDPEKIVAVQRWPVPNTVKQ--LRSYLGYIS---YY----------RKYV

QGFS----

>LLGY112

------------------------------------------------------------

------------------------------------------------------------

------------------------------------------------------------

------------------------------------------------------------

--QG------------KLK-TVFDAWNGYHSVPL---HPDDRHLTTF-------------

----ITP-WG------------------RYRYCVTPQGYIASGDGYSRR-----YDE---

-------IVAD--I----------------------------PQKTKCIDD-TLLWAD-T

M------EE---SFFQAIHWLDVCGKNGITLN--------------PDKFTF--------

---------------CRPEVEFAGFVI---------------------------------

----TLDNVRPCGKYLQAIRDFPTPRNITD--VRSWFGLVN-------------------

--------

>LLGY113

------------------------------------LD----------------------

---------------RLLADDIIEPV-----------QYS------DW------------

-----------------ATPVVPVMKA------D---K------------------S-VR

LCGDYK-LT------VN-------QVAKLDRYP-IPR-----------IEDL-YAQ---L

G-NG------------TSY-TKLDMRHAYEQIEL---HPESRKYVTI-------------

----NTP-RG------------------LFTYKRLPYGVSSAPGIYQRV-----MDS---

-------LLKG--I----------------------------KNTMVYLDD-VLVTGR-T

D------EE---HLQTLDLVMERLMSAGFCLK--------------RQKCHF--------

---------------MVEEVEYLGHRI---------------------------------

----DALGIHPSGHALVAVRDAPAPVNVAE--LRSYLGMVN---HY----------GRFV

SNLS----

>LLGY114

------------------------------------------------------------

-----------------------APV----------DEST------PW------------

-----------------VSQVVVVKKG------S---G------------------A-LR

VCIDPH-E-------LN-------KALQREHYT-LPI-----------LEDV-LHE---L

Q-GA------------TVF-SKAHLSSGYWHVKL---DYESSLLTTF-------------

----HAC-FG------------------RYRWCRLPFGTTVSSEVFQKH-----LLE---

-------ALRG--L----------------------------SGIICIADD-IVIYGK-T

S------EE---HDANLRKFFERCLETGIKLN--------------KDKLDI--------

---------------ELQEVTFMGHRI---------------------------------

----TKDGLRVDPTKVSAISEMQPPTNISE--LRRFMGMAN---YL----------ARFL

PHL-----

>LLGY115

------------------------------------------------------------

------------------------------------------------------------

------------------------------------------------------------

------------------------------------------------------------

----------------KKK-TVFDAWNGYHSVPI---RECDRHLTTF-------------

----ITP-WG------------------RYRYCTTPQGYIASGDGYTRR-----FDE---

-------IVAD--F----------------------------PNKTKCIDD-TCIWGD-T

I------EE---CFVQACAWLDQCGRNGITLN--------------PEKFRF--------

---------------AQDIVEFAGFTI---------------------------------

----TPDEVRPCSKYLDAIMQFPVPRNITD--VRSWFGLVNQVSY---------------

--------

>LLGY116

------------------------------------------------------------

---------------NMERLGVISKV----------TEPT------DW------------

-----------------VSSLVYSRKS------N---N------------------K-LR

ICLDPK-D-------LN-------TAIKRPHYK-TPT-----------LDEL-THK---L

A-GA------------TVF-SKLDARHGYWSVSL---DEPSSFLTTF-------------

----NSP-FG------------------RYRFQRLPFGLNLSQDVFQER-----MDH---

-------ILEN--C----------------------------EGTIGIADD-VAVFGK-N

E------AD---HDANLHNLMKTARQHGLVFN--------------ADKCHI--------

---------------KQPRMQFFGLIF---------------------------------

----DTDGVHPDPGKITAIKQLQAPQDATQ--LKEFLGIAT---YM----------SPFT

PNLS----

>LLGY117

------------------------------------------------------------

-------------------------------------GTT------PW------------

-----------------VSPIVVVPKP------H-NAN------------------E-IR

ICVDMR-S-------LN-------KAIIRERHI-IPT-----------TDDI-IAD---L

N-GC------------KVF-SKIDLNQGYHQLPL---HPDSRHLTTF-------------

----STH-VG------------------LYRYKRLNFGLSCAAEIFQRK-----VGD---

-------AIRG--I----------------------------PGVRNISDD-IYIGGI-D

E------AQ---HDDRLIKVLQRLKENQLTVN--------------VPKCLI--------

---------------RVPSMLFFGHVF---------------------------------

----SGEGVSPDPKKVEALRSVNAPNNVSE--VRSLLSSAA---FC----------SRFI

KDF-----

>LLGY119

------------------------------------------------------------

------------------------------------------------------------

------------------------------------------------------------

------------------------------------------------------------

-------------------------------------SERSSDLTCF-------------

----WTP-FG------------------RYKLKRMPFGITSAPEEFQRR-----LSN---

-------ALDG--L----------------------------PGVTIVADD-ILIYGK-G

DTMKDARED---HDNNLKQLLERSCQINFKLN--------------LNKCRF--------

---------------HMTEIPYIGHIL---------------------------------

----TSEGVKPDPGKVSAIQQMEPPRNSAE--VICFFGHVS---YL----------SKFL

PNLS----

>LLGY120

------------------------------------------------------------

------------------------------------------------------------

------------------------------------------------------------

------------------------------------------------------------

----------------SWK-TVTDAWNGYHSVPL---RDSDRHLTTF-------------

----ITP-FG------------------RWRYTRAPQGFLSSGDGYNRR-----FDE---

-------IIAN--F----------------------------QRKERCIDD-TVHWDV-E

L------QN---HWWRTIDYLILVGRAGVVLN--------------PDKFQF--------

---------------AQRTVDFAGFRI---------------------------------

----SNATIEPLPKYLDAIRDFPTPTSTTD--VRSWFGLVNQVTNY--------------

--------

>LLGY121

------------------------------------------------------------

----------------MVKMKVITRV----------TEPT------DW------------

-----------------VSSIVYSRKS------S---G------------------K-LR

ICLDPK-D-------LN-------EAVKRPHYK-TPT-----------LDEV-TYK---L

A-GA------------KVF-SKLDARHGYWSISL---DDESSRKTTF-------------

----NSP-FG------------------RFRFERLPFGLNLSQDVFQER-----MDN---

-------ILEQ--C----------------------------PGTMGMADD-VAVFGR-D

D------VE---HDRNLHNVMKVARKHGLVFN--------------VDKCDI--------

---------------HQPSIHFFGLVF---------------------------------

----DVTGVRPDPSKVNAIKRLETPRDTTQ--LQEFLGVAT---YM----------SPFI

PHLS----

>LLGY122

------------------------------------------------------------

-----------------VDLGVIAKV----------TEPT------DW------------

-----------------VSSLVYSRKS------N---G------------------R-LR

VCLDPK-D-------LN-------KAIKRPHYR-TPT-----------LDEI-THK---L

A-GA------------SMF-SKLDARHGYWSVKL---DDESSILTTF-------------

----NSP-FG------------------RYCFKRLPFGLNLSQDVFQER-----MDN---

-------ILEM--C----------------------------PGTISIADD-VGVFGR-D

A------AE---HDANLHHLMKTAQRHGLVFN--------------DAKCEI--------

---------------KRTTIKFFGLVF---------------------------------

----DADGVHPDPERIEDIRRMKKPENATE--LKEFLGIAT---YM----------SPFI

PNLS----

>LLGY123

------------------------------------------------------------

-----------------VEQKVIAPV----------DQPT------PW------------

-----------------VSNVLVTTKK------T---G------------------E-LR

VCIDPR-P-------LN-------KALKREHYQ-LPV-----------LDDI-LPE---L

T-TA------------KVF-SSIDLKSGYWHVIL---DEESSMLTTF-------------

----STP-SG------------------RYRWLRLPFGISVSSEIFQKK-----LQQ---

-------AIGD--L----------------------------QGVMCIADD-IVLYGV-G

NDHDEAMRD---HDEKLRMLLQRCRDVGIRIN--------------KDKLKL--------

---------------RKEEISFHGHLV---------------------------------

----TNMGLKLDPAKVEALQKMPPPTDVQG--VQRLGGFVN---YL----------ARFL

PRLS----

>LLGY124

------------------------------------------------------------

-----------------EKLGIVRR------------SSS------TW------------

-----------------ASPLHMVPKK------S---G------------------D-WR

PCGDYR-R-------LND------VTI-PDRYP-IPH-----------IQDF-AAQ---L

D-GK------------IIF-SKIDLVKGYHQIPV---APDDIHKTAI-------------

----ITP-FG------------------LFEFVRMPFGLRNSAQSFQRL-----MDN---

-------VLQG--I----------------------------DFVFVYLDD-ILVASS-S

A------TE---HQQHLRQLFERLTAHGLVVN--------------TTKCTF--------

---------------GIDAVDFLGHHV---------------------------------

----TAHGIKPLADRVESITHLTRPVDKKK--LQEYVGMLN---FY----------HRFV

PHVA----

>LLGY125

------------------------------------------------------------

---------------------VICPI----------EEPT------EW------------

-----------------SNHISVQTKK------D---A------------------S-LR

VCIDPR-P-------LN-------KVLQR-LYP-LPT-----------MEEV-LPE---M

S-TA------------RVL-SKVDLQSGYWHCEL---DHDSSLLTTI-------------

----ITP-FG------------------RYRWNRLPFGMNVSAEIFQRK-----LNQ---

-------TLEG--L----------------------------DRVVCVADD-IVVFGC-D

E------ED---HDKKLRLLLQRCRETGMKLN--------------RNKCEF--------

---------------RLDEINFMGHRV---------------------------------

----TSEGLKPDERKIEAILKMENPTDVKG--IQRLQGTIG---YL----------SEFL

PGLS----

>LLGY126

------------------------------------------------------------

-----------------IQQGIVRP------------SHS------EY------------

-----------------ASPIVICKKK------N---G------------------N-IR

MCVDYR-R-------LNT------KTR-KDAFP-LPR-----------VDEI-FDH---L

A-GA------------KYF-STVDLKSAYNQVEI---DESDQHKTAF-------------

----TTP-MG------------------LYEYTKMPYGLCNSPATFQRL-----MHI---

-------VFRE--EM-------------------------NE-KVLIFLDD-IIIYSS-T

I------EE---HFERLALVFQRLASHGLKIE--------------PTKCHL--------

---------------FQKSVSYLGQII---------------------------------

----SDQGISADPEKVKAVSEWPVPENARE--LKIFLGTAG---YH----------RRYI

HHFS----

>LLGY127

------------------------------------------------------------

--------------------------------------PT------PW------------

-----------------VSPIVVVPKP------H-NAN------------------E-IR

ICVDMR-S-------LN-------KAIIRERHI-IPT-----------TDDI-IAD---L

N-GC------------KVF-SKIDLNQGYHQLPL---HPDSRHLTTF-------------

----STH-VC------------------LYRYKRLNFGLSCAAEIFQ-------VGD---

-------AIRG--I----------------------------PGVRNTSDD-IYIDGI-D

E------AQ---HDDRLIKVLQRLKENQLTVN--------------VPKCLI--------

---------------RVPSMLFFGHVF---------------------------------

----CGEGVSPDPKKVEVLRSINAPNNVSE--VRSLLSSAA---FC----------LRFI

KD------

>LLGY128

------------------------------------------------------------

-----------------LRKGIIKE------------SMS------PY------------

-----------------ASPIVLVRKK------D---Q------------------S-LR

LCVDYR-H-------LNA------KTV-RDAYP-LPR-----------IEES-LDR---L

H-GA------------KWF-SVMDLASGFNQVAM---EEQDREKTAF-------------

----ITP-MG------------------LFECLRMPFGLTNSPATFQRL-----MQG---

-------VLGD--QM-------------------------FQ-ILLVYLDD-IIVYSQ-T

F------EE---HLDRLDVVFSRLGKHGLRLK--------------PEKCHF--------

---------------FKREVRYLGHLV---------------------------------

----SEHGVSPDPDKVAAVANWEVPKTVKE--LQRFLGFAS---YY----------RRFI

EHFS----

>LLGY129

------------------------------------------------------------

-------------------------V----------TEPT------PW------------

-----------------VSSMLVVVKP------D---K--------------------LR

ICIDPR-D-------LN-------RAICREHYQ-MPT-----------IEEV-ATR---L

T-NA------------KKF-TVLDAKDGFWQKRL---DTESSYKTTF-------------

----NTP-FG------------------RFRWNRMPFGISSAPEVWQRT-----MHE---

-------FVED--L----------------------------DGVEVIADD-FLIAGF-G

KTEDEVLRS---LEANERAFFEKCRRWNLKLN--------------RRKVKR--------

---------------CQSSVRFMGHLL---------------------------------

----TSDGLKADPEKIQAIIEMSEPGDIKA--LKRFLGMVN---YL----------SKYM

PRLS----

>LLGY130

------------------------------------------------------------

------------------------------------------------------------

----------------------FTTKT------D---G------------------S-NR

ICLDPK-R-------LN-------DSLKRCPHK-IPA-----------VEEL-NPE---F

A-HA------------TVF-SKLDAKAGYWAIHL---DEDSQLLTTF-------------

----RTQ-FS------------------RYCWQRLPFGLSTSLDIFQAR-----MDE---

-------IMEG--L----------------------------NGVVSIADD-VCVHGR-N

A------AG---RDANLINLMNRAAEKGLVFN--------------SDKCFI--------

---------------KQESISFYGNTY---------------------------------

----TAEGIKPDPSKVGGIQNMPAPQYKED--LQRFLGMMT---YL----------SQYI

PRFA----

>LLGY131

------------------------------------------------------------

------------------------------------------------------------

------------------------------------------------------------

-----Q-R-------LN-------DSLKRCPHK-IPT-----------VEEL-NPE---F

A-HA------------TMF-SKLDAKAGYWAIHL---DEDSQLLTTF-------------

----RTP-FG------------------RYCWQRLPFGLSTSQDIFQAR-----MDE---

-------IMEG--L----------------------------NG-VSIADD-VCVHGR-N

A------AD---RDANLINLMNRAAEKGLVFN--------------SDKCFI--------

---------------KQESISFYGNTY---------------------------------

----TAEGIKPDPSKVRDIQNMPAPQGKED--LQRFLGMMT---YL----------SQYI

PRFA----

>LLGY132

------------------------------------------------------------

-----------------EEMGIIRR------------SDS------PW------------

-----------------ASPLHMVPKN------S---G------------------G-WR

PCGDYR-R-------LND------ITV-PDKYP-VPH-----------IQDF-SSQ---L

A-GA------------TLF-SKIDLVRGYHQIPV---APTDISKTAV-------------

----ITP-FG------------------LFEFLRTPFGLKNAAQTFQRL-----MDT---

-------VCKG--L----------------------------TFVFVYLDD-ILVSSA-T

A------ED---HVSHLRTVFERLASHGLVIN--------------ESKCQF--------

---------------GTPTIDYLGHHI---------------------------------

----TREGAIPLPAKVDAIRTFERPTTVKG--LQQFAGMVN---FY----------HRFV

PNAA----

>LLGY133

------------------------------------------------------------

------------------------------------------------------------

------------------------------------------------------------

------------------------------------------------------------

------------------------------------------------------------

------------------------------------------------------------

------------------------------------------------------------

----------------------------LKLN--------------LSKCRF--------

---------------HMTEIPYIGHAL---------------------------------

----TSEGVKPDPGKVSAIQQTEPPRNTAE--VRRFFGHVN---YL----------SKFL

PNLS----

>LLGY134

------------------------------------------------------------

------------------ALGVISKV----------EQPT------DW------------

-----------------CAPMVVVPKS------Q---D------------------D-VR

ICVDLT-K-------LN-------ESVRRERYE-MPS-----------VDYT-LEQ---L

A-GA------------NIF-SKLDANSGFWQVPL---SEESTLLTTF-------------

----ITP-FG------------------RFAFRRLPFGISSAPEHYQRR-----MSA---

-------ILEG-------------------------------------------------

------------------------------------------------------------

------------------------------------------------------------

------------------------------------------------------------

--------

>LLGY135

------------------------------------------------------------

------------------------------------------------------------

------------------------------------------------------------

------------------------------------------------------------

------------------K-TVFDAWNGYHSVPI---RECDRHLTTF-------------

----ITP-WG------------------RYRYCTTPQGYIASGDGYTRR-----FDE---

-------IVAD--F----------------------------PNKTKCIDD-TCIWGD-T

I------EE---CFVQACAWLDQCGRNGITLN--------------PEKFRF--------

---------------AQDIVEFAGFTI---------------------------------

----TPDEVRPCSKYLDAIMQFPVPRNITD--VRSWFGLVNQVSY---------------

--------

>LLGY136

------------------------------------------------------------

-----------------IQQGIVRP------------SHS------EY------------

-----------------ASPIVICKKK------N---G------------------N-IR

MCVDYR-R-------LNT------KTM-KDAFP-LPR-----------VDDI-FDH---L

A-G---------------------------------------------------------

------------------------------------------------------------

------------------------------------------------------------

------------------------------------------------------------

------------------------------------------------------------

---------------------------------------------------------RY-

--------

>LLGY137

------------------------------------------------------------

---------------------IIEEV---------VDVPT------PW------------

-----------------VSPIVVQPKP------K-KPN------------------E-LR

ICVDMR-E-------AN-------RAIRRERHV-TPT-----------VDDV-IFE---L

N-WS------------SYF-TKLDLNKGYHQLEL---APESRYITTF-------------

----SAN-QK------------------LWRYKPLMFGLSSAAEVFQNA-----IQT---

-------TLQG--I----------------------------PKAFNISDD-ILVHGR-T

Q------AE---HDDNLRQAFEQVRATNLTLN--------------REKCVF--------

---------------NQRHLSFFGHVW---------------------------------

----SPEGVSADPQKLEAIKK---------------------------------------

--------

>LLGY138

------------------------------------------------------------

------------------QDKVIKRV----------TEPT------EW------------

-----------------VSSLAFSRKK------D---G------------------S-LR

ICLDPK-D-------LN-------RAIRRCHHK-TPT-----------LEEI-THK---F

T-GA------------KHF-SKLDAKNGYWSVKL---DQESSLLTTF-------------

----NSP-FG------------------RYCFTRMPFGLVMSQDVFQQK-----MDA---

-------ILDN--C----------------------------PGTVGIADD-VAVFGR-T

E------AE---HDANLRNLMSVAREYGLVFN--------------SKKCVV--------

---------------KASQISFFGILY---------------------------------

----DDKGAHPDPKKVEDIQMIPAPTNRTE--LQEFLGIVT---YM----------GPFV

PNLS----

>LLGY139

------------------------------------------------------------

----------------------------------------------EW------------

-----------------AAPIVPVCKS------N---G------------------Q-VS

ICGDHK-VT------IN-------QGMAEDKYP-LPR-----------VNDL-HAG---L

N-GG------------ETF-SKLDLSQAYLQLTP---DEDSLNYVTI-------------

----NTH-KG------------------LFRYTRLLYGLTVA------------------

------------------------------------------------------------

------------------------------------------------------------

------------------------------------------------------------

------------------------------------------------------------

--------

>LLGY140

------------------------------------------------------------

--------------------GIIRKV----------KGHT------DW------------

-----------------CSSIVYSTKK------D---G------------------S-LR

ICLDPK-R-------LN-------EAIKGCPHK-TPT-----------LEEI-NPA---F

V-GA------------RWF-SKLDAKSGYWSVQL---DEQSQLLTTF-------------

----RTP-IG------------------RYCYQRLPFGLCVSQDIFQQR-----MDE---

-------ILEG--L----------------------------DGCVGIADD-ICVFGA-T

Q------EE---HDERLVALLEVANSSGLVFN--------------SAKCTI--------

---------------KQKSISFFGNIY---------------------------------

----SAEGVSPDPSKVHDIHEMPVPQDKED--L---------------------------

--------

>LLGY141

------------------------------------------------------------

------------------------------------DGPT------PW------------

-----------------VSPIVVVPKK------T---G------------------A-VR

ICVDMR-E-------AN-------KAVKREKHL-MPT-----------IDDL-ITD---L

N-GA------------TMF-STLDLRAGYHQLEL---DPESRQITTF-------------

----STH-VA------------------LYRYKRLMFGINAASEIFQNT-----IAE---

-------LLHG--L----------------------------NGCRNISDD-IIVHGK-T

P------AE---HNANLRAVLDRLRENNVRLN--------------REKCKF--------

---------------SQPTVTFYGHVF---------------------------------

----GAHGLRADPKKLESISKARRPTNPSE--VRSLLGMAQ---YV----------SRFI

ADF-----

>LLGY142

------------------------------------------------------------

-----------------------EAV----------EKPT------AW------------

-----------------ISSMVVITKK------D---S------------------K-LR

ICLDPK-D-------LN-------RAIRRENYQ-LPT-----------IEDI-ATR---L

H-GA------------KVF-TVLDVRHGFWHVRL---DDRSSYLTTF-------------

----HTP-FG------------------RYRFKRMPFGISSAPEVFKKK-----MHE---

-------LIEG--L----------------------------QGIEVVADD-FVVVGY-G

NTVDEANVD---HDKRLHSFLQRCEERGVKLN--------------VDKFKL--------

---------------RQEEVRFIGHVA---------------------------------

----TSDGLSIDPTKVKAIVDMPNPTDVAG--VQRLLGLAQ---YL----------AKFL

PHLS----

>LLGY143

------------------------------------LG----------------------

---------------KLKKAGVITPV-----------TSS------DW------------

-----------------AMGVVVVPKT------N---G------------------A-IR

LCGNYK-TT------VN-------PQLKTVSPP-NIN-----------IDDI-LAD---L

A-GG------------DKF-SKLDLANASNQMEV---SEESREYLTI-------------

----ATH-NG------------------LFRQNRLVFGITTAPAI---------------

--------SQG--L----------------------------PGVKVYLDD-ILVSGR-T

E------SE---HLSNLGRVFERLTEFGLKLN--------------RDKCEF--------

---------------SRD------------------------------------------

------------------------------------------------------------

--------

>LLGY144

------------------------------------------------------------

----------------------------------------------DW------------

-----------------VNSLVIAEKS------N---G------------------K-MR

LCIDPK-D-------LN-------KEIKREHFQ-IPT-----------KEEI-IGK---L

A-NA------------TCF-SKLDATAGFHQIQL---DRPSSLLTTF-------------

----NTP-FG------------------RYRYLRLPMGICSAPEVFHKT-----VHQ---

-------FLED--I----------------------------EGVSVYMDD-IIVWGS-T

A------AE---HDERLMKTLQRLSEVGLGLN--------------MEKCVF--------

---------------RQPEISYLGEVV---------------------------------

----TQDGVKPDPEKIQAITDMPTPTNATE--LQRVLGMVT---YL----------GRYI

PNLS----

>LLGY145

------------------------------------------------------------

---------------NMERLGVISKV----------TEPT------DW------------

-----------------VSSLVYSRKS------N---N------------------K-LR

ICLDPK-D-------LN-------TAIKRPHYK-TPT-----------LDEL-THK---L

A-GA------------TVF-SKLDARHGYWSVSL---DEPSSFLTTF-------------

----NSP-FG------------------RYRFQRLPFGLNLSQDVFQER-----MDH---

-------ILEN--C----------------------------EGTIGIADD-VAVFGK-N

E------AD---HDANLHNLMKTARQHGLVFN--------------ADKCHI--------

---------------KQPRMQFFGLIF---------------------------------

----DTDGVHPDPGKITAIKQLQAPQDATQ--LKEFLGIAT---YM----------SPFT

PNLS----

>LLGY146

------------------------------------------------------------

------------------------------------DEPT------DW------------

-----------------VHNLVITEKR------N---G------------------S-LR

ICLDRR-P-------LN-------KAIKRERCE-IPT-----------PADV-QSQ---L

G-DK------------LIF-TVIDMKDGYWHVKL---TEESSHLCTF-------------

----HTP-WG------------------RKRFTRMPFGISSASDVMQ-R-----NEE---

-------TFAD--I----------------------------PGVRIIADD-MIISAK-D

E------TE---HDAIVRKVMQRARERNVKFN--------------KTKVQF--------

---------------KVPNVTYMGHIV---------------------------------

----AADGLKPDPAKVEAIVMMPKPENKSD--LQRLLGMVR---YL----------AQYI

PNES----

>LLGY147

------------------------------------------------------------

------------------------------------DHPT------KW------------

-----------------CHPLVVVPKS------R---G------------------G-VR

LCVDLT-Q-------LN-------KHVRRPIHP-MKT-----------PKEA-VSN---I

TPGS------------KYF-SSLDAKHGYWQIAL---SPECQELTTF-------------

----LTP-WG------------------RYQFLRSPMGLSSTGDEYCRR-----GDI---

-------AIAG--L----------------------------NNIQKVMDD-VIVFDE-N

F------ER---HVERVRTLLQRCREHGITLN--------------ADKFVF--------

---------------AENELNYVGYKV---------------------------------

----NADGVTTDPEKLKAIAEFPAPNCLTE--LRSFMGLVN---QL----------GDFT

--------

>LLGY148

------------------------------------------------------------

----------------------VLER---------VDGPT------PW------------

-----------------ESLIVVAPKP------K-RLS------------------E-VH

ICVDMR-E-------AN-------HAIKRERHP-SST-----------MDDI-VHR---L

N-YT------------RVF-SKVDLKSGYHQLVL---AEKSRYITTF-------------

----SMH-DG------------------LWRYKCLNFGISSESEMFQNV-----I-----

--------------------------------------------------D-V-------

------------------------------------------------------------

------------------------------------------------------------

------------------------------------------------------------

--------

>LLGY149

------------------------------------------------------------

------------------------------------------------------------

------------------------------------------------------------

---------------------------------------------------I-KP-----

--------------------------------------------ATF-------------

----ATP-FG------------------HYHWRRLCFGLKVSSEIFQKR-----LQQ---

-------ALEG--L----------------------------DNVHCVADD-IIIHGS-D

D------SD---LCIKVQRLLQRCMEHGIKLN--------------LEKCRF--------

---------------NVDEIPFLGHVV---------------------------------

----TADGLKPDPSKVEAVLKMERPGDKEA--VERLRGTVT---YL----------ARYV

PELI----

>LLGY150

------------------------------------LG----------------------

---------------KLEKAGVITPV-----------TIS------DL------------

-----------------ATGVVVVPKK------N---G------------------A-IR

LCGNYK-TT------VN-------PQLKTVSQP-NIN-----------IDDI-LAD---L

A-GG------------VKF-SKLDLANAYNQMEV---SDESREYLTI-------------

----ATH-NG------------------LFRQNRLVFGITTAPAI-QNA-----IEK---

-------VLQG--L----------------------------PGVKVYLDD-ILVSGR-T

E------SE---HLSNLGRVFERLTEFGLKLN--------------RDKYEF--------

---------------SRDSLEYLGHVI---------------------------------

----DVQGIHKSADKIAVILYAPRPTEVNS--LRSFIGMAN---YY----------RKFV

PDFA----

>LLGY151

------------------------------------------------------------

-----------------IQQGIVRP------------SHS------EY------------

-----------------ASPIVICKKD------N---G------------------N-IR

MCVDYR-R-------LNT------KTR-KDAFP-LPR-----------VDEI-FDH---L

A-GA------------KCF-STVDLKSAYNQVEI---DESDQHKTAF-------------

----TTP-MG------------------LYEYTKMPYGLCNSPATFQRL-----MHI---

-------VFRE--EM-------------------------NE-KVLIFLDD-IIIYSS-T

I------EE---HFERLALVFQRLASHGLKIK--------------PTKCHL--------

---------------FQKSVSYL-QII---------------------------------

----SDQGISADPEKVKAVSEWPVPENARE--LKIFLGTAG---YH----------RRYI

HHFS----

>LLGY152

------------------------------------------------------------

-----------------EKLGIVRR------------SSS------TW------------

-----------------ASPLHMVPKK------S---G------------------D-WR

PCGDYR-R-------LND------VTI-PDRYP-IPH-----------IQDF-AAQ---L

D-GK------------IIF-SKIDLVKGYHQIPV---APDDIHKTAI-------------

----ITP-FG------------------LFKFVRMPFGLRNSA-SFQRL-----MDN---

-------VLQG--I----------------------------DFVFVYHDY-ILVASS-S

A------TE---HQQHLRQLFERLTAHGLVVN--------------TTMCTF--------

---------------GIDAVDFLGHHV---------------------------------

----TAHGIKPLADHIESITHLTRPVDKKK--LQEYVGMLN---FY----------HCFV

PHVA----

>LLGY153

------------------------------------LG----------------------

---------------RLENEGTIKPV-----------EFS------DW------------

-----------------ATPIVPIVKS------D---S------------------S-VR

IYGDYK-LT------VN-------KASRMDSYP-IPN-----------VDEL-FAK---L

A-GG------------QIY-SELDLSHAYKQILL---DESSCECVTI-------------

----NTH-RG------------------LSQYQRLPYGVSSSPGIFQRI-----MEC---

-------LFQG--M----------------------------TDVAPYLDN-VIITGK-D

D------EE---HLKNLALVLEKISQAGLRLK--------------RSKCEF--------

---------------MTEKMVALEHVL---------------------------------

----SGKGIQPCKAKVEAIQNTPATTNVTE--LRAYLGLIN---YY----------HRYL

RNLS----

>LLBP2

------------------------------------------------------------

-------------------HGYAEPV---------PAEELTIAEGRTW------------

-----------------YIPHHGVYHP------K-KPG------------------K-IR

VVFDCS-AEYRG-EVLNRHLLQG------------PDL----------TNNL-TGV---L

C----RFRQE------PVA-VSCDIESMYHQVGV---NTEDRNFLRFLWWDNC----NL-

----DSE-PK------------------EYRMTVHLFGATSSPGCANYA-----LKATAD

MFEKDCGKLAA-----------------------------DFVRSNFYVDD-GLKSVA-T

P------AE---ALQLVESSRDMCKKGGFNLHKYMCNSKEVLVAISPEL-------RAKD

AQNLDLT------CDSLPIERTLGVQWCIESDTFQFRVEVKDRPL---------------

----------TRRGILSTVSSVFDP-----------LGLVS--PYVLRG-------KQIL

QELV----

>LLGY154

------------------------------------------------------------

-----------------------EAV----------EKPT------AW------------

-----------------ISSMVVITKK------D---S------------------K-LR

ICLDPK-D-------LN-------RAIRRENYQ-LPT-----------IEDI-ATR---L

H-GA------------KVF-TVLDVRHGFWHVRL---DDRSSYLTTF-------------

----HTP-FG------------------RYRFKRMPFGISSAPEVFPKK-----MHE---

-------LIEG--L----------------------------QGIEVVADD-FVVVGY-G

NTVDEANVD---HDKRLHSFLQRCEERGVKLN--------------VDKFKL--------

---------------RQEEVRFIGHVA---------------------------------

----TSDGLSIDPTKVKAIVDMPNPTDVAG--VQRLLGLAQ---YL----------AKFL

PHLS----

>LLGY155

------------------------------------------------------------

------------------------------------------------------------

------------------------------------------------------------

------------------------------------------------------------

----------------KKK-TVFDAWNGYHSVPI---RECDRHLTTF-------------

----ITP-WG------------------RYRYCTTPQGYIASGDGYTRR-----FDE---

-------IVAD--F----------------------------PNKTKCIDD-TCIWGD-T

I------EE---CFVQACAWLDQCGRNGITLN--------------PEKFRF--------

---------------AQDIVEFAGFTI---------------------------------

----TPDEVRPCSKYLDAIMQFPVPRNITD--VRSWFGLVNQVSY---------------

--------

>LLGY156

------------------------------------------------------------

-----------------MRNGTIVP------------SKS------NH------------

-----------------ASAIVLVRKK------N---G------------------D-LR

MCIDYR-A-------LNN------KTI-KDAHP-LPR-----------IVES-LDA---M

T-GA------------QYF-TTLDLQSAYNQVQM---EPDDQHKTAF-------------

----TTP-FG------------------LYEFTRMPYGLCNAAATFQRL-----MQM---

-------TFSA--EM-------------------------FE-ILLVYLDD-IVIFSK-S

I------EE---HLKRLDAVFTKLRQFGLKLE--------------LKKCNF--------

---------------FKKEVLYLGHLI---------------------------------

----GADGVATDPSKIAVVEKWPIPKTLKD--LRSFLGFAS---YY----------RRYV

PNFT----

>LLGY157

------------------------------------------------------------

-----------------------EAV----------EKPT------AW------------

-----------------ISSMVVITKK------D---S------------------K-LR

ICLDPK-D-------LN-------RAIRRENYQ-LPT-----------IEDI-ATR---L

H-GA------------KVF-TVLDVRHGFWHVRL---DDRSSYLTTF-------------

----HTP-FG------------------RYRFKRMPFGISSAPEVFQKK-----MHE---

-------LIEG--L----------------------------QGIEVVADD-FVVVGY-G

NTVDEANVD---HDKRLHSFLQRCEERGVKLN--------------VDKFKL--------

---------------RQEEVRFIGHVA---------------------------------

----TSDGLSIDPTKVKAIVDMPNPTDVAG--VQRLLGLAQ---YL----------AKFL

PHLS----

>LLGY158

------------------------------------------------------------

--------------------------------------PT------PW------------

-----------------VNNLVITEKR------N---G------------------S-LR

LCLDPK-P-------LN-------RAIKREQFE-IPT-----------PEDV-QSR---L

A-GK------------KVF-SVIDMSMAYWHVRL---SDESSYLCTF-------------

----HTP-WG------------------RKRFLRMPFGISSASEVLQKR-----MQL---

-------TFGD--I----------------------------KGAYVIADD-IIIAGD-D

E------QD---HDAIMRAVFQRARERNVKFN--------------SSKVQL--------

---------------KVNEVLYMGHVV---------------------------------

----SEAGLSPDPSKVRAIVDMPLPEDKRA--VLRFLNIVK---YQ----------AKFI

PRES----

>LLGY159

------------------------------------LQ----------------------

---------------RLEDLKIVTKV-----------DYS------DW------------

-----------------ATPIVVVQKP------S---G------------------K-VR

ICGDYR-AT------VN-------PCLHVQQHP-IPR-----------IEEL-FAK---L

Q-GG------------MHF-SKLDMRDAYLQIEL---DDETKQLLVI-------------

----NTH-KG------------------LYRYNRLCFGPSPAPAIFQKL-----VDN---

-------LVAG--I----------------------------PGVAAYLDD-IIVTGQ-T

K------AE---HLENLRRVFAALDNYGLKLQ--------------LDKCVF--------

---------------FAPEVSYLGYII---------------------------------

----SKDGLCASEERVQAILQYATPTDLKQ--LESFVGKLN---YY----------GKFL

PAFA----

>LLGY160

------------------------------------LE----------------------

---------------RLQKEGIISPV-----------EYS------QW------------

-----------------ASGVVPVAKR------E---T-----------------KD-VR

LCGDYK-VT------IN-------PVLREDKYP-LPR-----------IEDI-FAK---M

A-GG------------KRF-SKIDLKNAYLQMEV---EEDSKKYLTI-------------

----NTH-KG------------------LFKYNRLPFG--TAPSIWQRA-----MEQ---

-------TLQG--I----------------------------PGVEVMLDD-IIVTGK-S

D------AE---HLENLEAVLRRLTEKDLRIN--------------AKKCRF--------

---------------FMERIEYCGHEI---------------------------------

----DHDGLHKTKAKIEAVQKAPHPQDVSS--LR-FLGLVN---YY----------HRFL

PNLA----

>LLGY161

------------------------------------------------------------

--------------------------------------PP------PW------------

-----------------CAP-----KK------P---D------------------Q-VR

ICIDLK-Q-------LERG-----RGRGRERYI-IPA-----------ISQV-LAK---L

T-GS------------VRF-SKLDASGGYWQLAL---DEESSNLTTF-------------

----VTP-LG------------------RFCMTRVPF---------QRK-----MGE---

-------LLEG--L----------------------------RGVECYQDD-IIVHGR-T

V------EE---HNSVLQKVLQRIRV----------------------------------

------------------------------------------------------------

------------------------------------------------------------

--------

>LLGY162

------------------------------------------------------------

------------------------------------TEAT------DW------------

-----------------VSSIVIVQKP------S---G------------------Q-IR

IRIDPK-D-------LN-------TAIRREYYP-MPT-----------IEEV-STR---L

K-NA------------RLF-TVLDAKNGFWQIPL---DEKSSMLTCF-------------

----NPP-FG------------------RYRWLRMPFGINSAPEIWQRT-----INQ---

-------LVEG--L----------------------------TGTQVIHDD-FLIVGC-G

TTDKEAEID---HDKILRAFLDRAGERNLRLN--------------AEKVNL--------

---------------KMTEVPYIGHLL---------------------------------

----TREGLRVDPKKVEAIEKMPEPEDAKA--VQRLLGSVN---YL----------AKFI

PHLS----

>LLGY163

------------------------------------------------------------

----------------MVKMKVITRV----------TEPT------DW------------

-----------------VSSIVYSRKS------S---G------------------K-LR

ICLDPK-D-------LN-------EAVKRPHYK-TPT-----------LDEV-TYK---L

A-GA------------KVF-SKLDARHGYWSISL---DDESSRKTTF-------------

----NSP-FG------------------RFRFERLPFGLNLSQDVFQER-----MDN---

-------ILEQ--C----------------------------PGTMGMADD-VAVFGR-D

D------VE---HDRNLHNVMKVARKHGLVFN--------------VDKCDI--------

---------------HQPSIHFFGLVF---------------------------------

----DVTGVRPDPSKVNAIKRLETPRDTTQ--LQEFLGVAT---YM----------SPFI

PHLS----

>LLGY164

------------------------------------------------------------

-----------------VEKGIITPV----------NEPT------DW------------

-----------------VSALVVVKKK------D---T------------------NDIR

ICMDPG-D-------LN-------RAIKRPHYP-MRT-----------IEEV-VEN---I

P-NA------------KYF-TVMDAKQAFYHIPL---ENDSSYLTTF-------------

----GTP-FG------------------RFRYLRMPMGISSASEVYQRG-----IEH---

-------LLAG-------------------------------YPCAVIMDD-ILVSGA-S

E------ME---HDTNLEKVLKRLREINLKLA--------------PRKCKY--------

---------------KLQEIPYIGHVL---------------------------------

----SKDGLKPDPRKVQAITEMPEPENTTD--LLRFMGMVK---YL----------SKFV

PDLS----

>LLGY165

------------------------------------LQ----------------------

---------------RLEDLKIVTKV-----------DYS------DW------------

-----------------ATPIVVVQKP------S---G------------------K-VR

ICGDYR-AT------VN-------PCLHVQQHP-IPR-----------IEEL-FAK---L

Q-GG------------MHF-SKLDMRDAYLQIEL---DDETKQLLVI-------------

----NTH-KG------------------LYRYNRLCFGPSPAPAIFQKL-----VDN---

-------LVAG--I----------------------------PGVAAYLDD-IIVTGQ-T

K------AE---HLENLRRVFAALDNYGLKLQ--------------LDKCVF--------

---------------FAPEVSYLGYII---------------------------------

----SKDGLCASEERVQAILQYATPTDLKQ--LESFVGKLN---YY----------GKFL

PAFA----

>LLGY166

------------------------------------------------------------

-------------------QQIIRTI----------EEPT------DW------------

-----------------VSSLTYVTKR------D---G------------------S-IR

VCLDPR-Q-------LN-------KALIRPRHE-APT-----------LDEL-NHK---F

A-NA------------KFF-SKLDAKAGYWSIKL---DDESQKLTTF-------------

----QTP-FG------------------RYCFLRLPFGLSVSQDIFQLE-----MDR---

-------ILEK--C----------------------------NGVCGIADD-FVVYGT-T

E------IE---HDRNLLQFMDIAKQHGLALN--------------SAKCDI--------

---------------KCNKVSFFGQLY---------------------------------

----TSEGIKPDPQKVNDLRAMPVPTTKAE--LQQFLGFIT---YL----------SRFM

KAFS----

>LLGY167

------------------------------------LD----------------------

---------------RLLADDIIEPV-----------QYS------DW------------

-----------------ATPVVPVMKA------D---K------------------S-VR

LCGDYK-LT------VN-------QVAKLDRYP-IPR-----------IEDL-YAQ---L

G-NG------------TSY-TKLDMRHAYEQIEL---HPESRKYVTI-------------

----NTP-RG------------------LFTYKRLPYGVSSAPGIYQRV-----MDS---

-------LLKG--I----------------------------KNTMVYLDD-VLVTGR-T

D------EE---HLQTLDLVMERLMSAGFCLK--------------RQKCHF--------

---------------MVEEVEYLGHRI---------------------------------

----DALGIHPSGHALVAVRDAPAPVNVAE--LRSYLGMVN---HY----------GRFV

SNLS----

>LLGY168

------------------------------------------------------------

-----------------LHDEIIRP------------SNS------AY------------

-----------------SSPILLVPKK------D---G------------------D-SR

LCVDYR-K-------LNA------VTE-QDAYP-LPI-----------IQDI-FDL---V

G-GS------------SIY-STLDLKSSYWQMPV---AEADIHKTAF-------------

----RCH-AG------------------HFEFTKVPFGLKSAPNFFQKE-----MNT---

-------ILAD--L--------------------------IGKCVFVYIDD-ILVFSK-N

E------TD---HIRHLQLVFDRLRNAGLKLK--------------PTKCAF--------

---------------GLPEVKLLGYVL---------------------------------

----NADGIKTDPDKVAVIANLLPPTTVKE--TRSFLGMCN---YY----------RNSL

PNYA----

>LLGY169

------------------------------------LE----------------------

---------------RLQKEGIIFPV-----------EYS------QW------------

-----------------ASGVVPVAKR------E---T-----------------KD-VR

LCWYYK-VT------NN-------PVLREDKYP-LPR-----------IEDI-LSK---M

A-GG------------KRF-SKIDLKNAYLQMEV---GEDSKKYLTI-------------

----DTH-KG------------------LFKYNRLPFGIKTAPSIWQRA-----MEQ---

-------TLQG--I----------------------------PGVEVMLDD-IIVTGK-S

D------AE---HLENLGAVLRRLAEKDLRIN--------------AKKCRF--------

---------------FMERIEYCGHEI---------------------------------

----DHDGLHKTKAKIEAVQKAPHPQDVSS--LRGFLGLVN---YY----------HRFL

PNLA----

>LLCO1

------------------------------------------------------------

-------------------QIEYKSLMDHG----------------TW--------ELV-

------PLPPDKK--LVGSRWVFKAKH------D-ESG----------AVERY---K-AR

FVAQGF-TQVFG---EDYN----------QTFS--PVVRWES------VRTI-ISL----

---AAQ-Y--------DMEIHQMDVETAFLNGWL---DEE-----IYM------------

----KQP-EGFASPG---------QQNLVCKLKRSLYGLKQSPRCWNEV-----LHE---

-------QLVD--MQFVQSAADQC-LYI-----GKICGSLV--FVAIYVDD-ILIASK--

------------EIQVVQKTKELFAKK-FKVKD-------------MGKLH---------

--------------------YFLRVRIH--------------------------------

-----------------------------Q--SDGAFWLGQ-EKYA----------ENIL

TKFK----

>LLGY170

------------------------------------------------------------

-----------------------AKV----------EELT------EW------------

-----------------VNPIVNVLKP------N---K------------------Q-LR

ICLDPQ-K-------LN-------ETIMREHYA-LPV-----------AADI-FAR---I

S-GA------------KVY-STLDATAGFHQIEL---EEQSSYYTTF-------------

----ITA-YG------------------RYRYLRLPFGVKYAPEVMHKA-----ISQ---

-------MFDS--I----------------------------EGVECYIDD-ILVWGR-D

Q------AE---HDERLRKVLQKCRVEQLTLN--------------KDKCKI--------

---------------SQPTVKFIGHEL---------------------------------

----SK-GLAPSRDRVQSVVEMTTPQTKED--VQRFIGFAT---YL----------SKFC

PRLS----

>LLGY171

------------------------------------------------------------

--------------------DVIEKV----------NGPT------QW------------

-----------------VNPVVTVEKP------N---G------------------D-VR

VCLDMR-E-------AN-------KAIIRERQP-IPT-----------VEET-VQE---M

G-EM------------KVF-TKLDLNMAFHQVEL---HPESRDITTF-------------

----AAP-NG------------------VYRYKRLVFGLNMASEKFNHA-----TRQ---

-------VVQD-------------------------------------------------

------------------------------------------------------------

------------------------------------------------------------

------------------------------------------------------------

--------

>LLGY172

------------------------------------------------------------

------------------------------------------------------------

------------------------------------------------------------

------------------------------------------------------------

------------------------------------------------------------

------------------------------------------------------------

------------------------------------------------------------

------------------------------------------------------------

-------------------VVYLSHKI---------------------------------

----DQHGLHLIEDKVETIQKARATENVLE--LQAFLGLLN---YY----------GRFI

ANLS----

>LLGY173

------------------------------------------------------------

------------------------------------------------------------

------------------------------------------------------------

------------------------------------------------------------

----------------KKK-TVFDAWNGYHSVPI---RECDRHLTTF-------------

----ITP-WG------------------RYRYCTTPQGYIASGDGYTRR-----FDE---

-------IVAD--F----------------------------PNKTKCIDD-TCIWGD-T

I------EE---CFVQACAWLDQCGRNGITLN--------------PEKFRF--------

---------------AQDIVEFAGFTI---------------------------------

----TPDEVRPCSKYLDAIMQFPVPRNITD--VRSWFGLVNQVSY---------------

--------

>LLGY174

------------------------------------------------------------

-----------------MRNGTIVP------------SKS------NH------------

-----------------ASAIVLVRKK------N---G------------------D-LR

MCIDYR-A-------LNN------KTI-KDAHP-LPR-----------IVES-LDA---M

T-GA------------QYF-TTLDLQSAYNQVQM---EPDDQHKTAF-------------

----TTP-FG------------------LYEFTRMPYGLCNAAATFQRL-----MQM---

-------TFSA--EM-------------------------FE-ILLVYLDD-IVIFSK-S

I------EE---HLKRLDAVFTKLRQFGLKLE--------------LKKCNF--------

---------------FKKEVLYLGHLI---------------------------------

----GADGVATDPSKIAVVEKWPIPKTLKD--LRSFLGFAS---YY----------RRYV

PNFT----

>LLGY175

------------------------------------------------------------

------------------------------------------------------------

------------------------------------------------------------

--------------------------LKREHYR-LHV-----------LDDI-LPK---L

V-NS------------KKF-AVCDLQQGYLHCEL---DDELSLLTTF-------------

----ATP-FG------------------RYRWRRLPFGLKVSSEIFQKR-----LQQ---

-------ALEG--L----------------------------DNVHCVADD-IIIHGS-D

D------SD---LCTKVQRLIQRCMEHGIKLN--------------LEKCRF--------

---------------NVDEIPFLGHVV---------------------------------

----TADGLKPDPSKVEAVLKMERPGDKEA--VERL------------------------

--------

>LLGY176

------------------------------------------------------------

-------------------------I----------EEPT------EW------------

-----------------CNQISIQTKK------D---A------------------S-LR

VCIDPR-P-------LN-------KVLQRELYP-LPT-----------MEEV-LPE---M

S-TA------------RVL-SKVDLQSGYWHCEL---DHESSLLTTI-------------

----ITP-FG------------------RYRWNRLPFGMNVSAEIFQRK-----LNQ---

-------TLEG--L----------------------------DGVVCVADD-IVVFGR-D

E------ED---HDKKLRLLLQRCRETGMKLN--------------RNKCEF--------

---------------RLDEINFMGHRV---------------------------------

----TSEGLKPDERKIEAILKMENPTDVKG--IQRLQGTIG---YL----------SKFL

PGLS----

>Moose

------------------------------------------------------------

-------------------LGHMVPV---------SPDNLDAA--TCC------------

-----------------YLPHHPVFKE-----TS-STT------------------K-MR

VVFDGS-APTSTGHSLNDALLVG------------PVI----------QDDL-LSL---I

I----RFRKF------QVA-LVPDLEKMYRQVLV---HPEDRPLQRYG------------

---------G------------------AYELRTVTYGLAPSSFLATRT-----LQQLAE

DEGDA---FPT--AK-------------------------DTLKKQLYMDD-LIAGSN-S

V------DG---AIQLREELSALAQRGGFTFRKWCSNSLAVLSDVPAE------------

----QLATKSSLRFDDKETISTLGICWEPEIDTFQFNISITTKSER--------------

---------DTMRTILSMIAELYDP-----------LGLIS--PVIITA-------KVLM

QSLW----

>Max

------------------------------------------------------------

-------------------LGHMKEV---------PPTHNSP----SY------------

-----------------HLPHHAVVKP-----ES-TTT------------------K-LR

VVFNAS-SPSANGISLNDILHAG------------PVL----------QSDL-TIQ---V

L----KWRYF------QYV-FSADITKMYRQIWV---DPKHTPFQRIL-FRNK-------

----EGD-IR------------------DFELKTVTFGVNCAPFLAIRV-----LQQLAE

DIQVP---FPN--AS-------------------------RIIQQHMYVDD-VLAGAN-S

V------NE---AQSSIRELQAALSASGFPLRKWTSNNKSVLKDVPAEHLLH--------

--------SEFLDIDAESTAKTLGIRWRAKSDEFYFVPPDIVVEAS--------------

---------YTKREVLSQIARLFDP-----------AGWLA--PFIIRS-------KIFM

QEIW----

>Tribel

------------------------------------------------------------

-------------------LEHMQEI----------KTMTDETNNNGY------------

-----------------YFLHHPVVND----KQD----------------------K-IR

VVFDGS-AQTDCGLSLNDCQMVG------------PTI----------QNEL-LSI---I

L----RFRKY------PYV-LTADIEKMYRMVLM---HPDFHRYQRIYWRNQS-------

----DEE-MK------------------TYELTTVTYGTSAAAFLAIRC-----LYQVAL

DLKSD---DPE--LS-------------------------KVIENDFCVDD-LMTGAQ-S

M------EA---AMELKDRLCKALNQVGFNLTKWRTN----FDHVPEETETI--------

-------------MLGDNESKKLGTLWNSRQDTINYEIVFGEPPRS--------------

---------TTKRVIISEVSKIFDP-----------LDLLG--PVVIKA-------KILM

QKLW----

>Nabel

--------------------------------TKV-IE----------------------

---------------EYLKLGH------------------------GF------------

-----------------YLPHHGVPKL-----DS-LTT------------------M-LR

VVFNASVTPSDGGCSLNDCLLVG------------PTI----------QDDL-ITL---L

L----RFRTH------TYV-ILADIEKMYRQFLV---RDSDRKYQKILWFVND-------

----E---IR------------------EFVLNTVTFGVAAAPFLAIRC-----LHQLAD

DEQER---FPL--AA-------------------------KILKRDMYVDN-MLTGTN-S

V------EE---ARSICTQMTQVLRTAGMNMRQWAANDPNILSDIESKNL----------

--------DANFDLSNDNTLKTLGIRWRAKTDSFVYKIKPISVTER--------------

---------FTKRKILSEIAKIFDP-----------LGLLG--PIILFA-------KKIM

QDIW----

>Roo

------------------------------------------------------------

-------------------LGHMIEV---------SDE-------GKY------------

-----------------FLPHQAVIRD-----SS-LTT------------------K-LR

VVFDAS-AKTTNNKSLNDIMWVG------------PRV----------QKDI-FDI---I

I----KWRKW------EFV-VSADIEKMYRQIKI---DNNDQKYQYILWRNSP-------

----KEK-IK------------------TYKLTTVTYGTASAPYLATRV-----LVDIAD

KCKNQ---VIS--A---------------------------IIRNDFYMDD-LMTGAD-S

V------EE---ANKLITLIPHELQKVGFNLRKWISNNSKILTTVEDTGD----------

--------NKVLNIIENECVKTLGLKWEPQKDLFKFSVNCNDESKN--------------

---------INKRVVLSTLAKIFDP-----------LGWLA--PVTVSG-------KLFI

QKLW----

>Bel

------------------------------------------------------------

-------------------RGHMEKL---------TSAQVEESPDTCF------------

-----------------YLPHHAVIKL-----DS-LTT------------------K-CR

VVFDGS-GKDSSGVSLNDRLHIG------------PPI----------QRDL-FGV---C

L----RFRQH------QYV-LCADVEKMFRGIKV---FKPHTNFQRIVWRTTE-------

----NEP-LL------------------HFRLLTVTYGLAPSPFLAVRV-----LKQLAD

DHGHE---YPA--AA-------------------------HALLHDAYVDD-IPTGAN-T

F------EE---LMILKDELIALLDKGKFKLRKWSSNSWRLLKSLPEEDR---------C

FEPIQLLNKSA----ADSPVKVLGIQWNPGKDVLYLNLKGCDATIS--------------

---------PTKRELLSQLSRIYDP-----------LGLVA--PVTVLL-------KLIF

QESW----

>Cer7

------------------------------------------------------------

-------------------KNILEEV-------DVSKDTEGMR---IH------------

-----------------YNPHSPVLTP-----QK-TTT------------------K-CR

VVIDGS-AHFKNEPSLNDAIYQG------------PTI----------LPDS-VDT----

-----RFRSG------KTV-LLADVEKAFLQVHL---NESDRDVTRVLWVKNP----DLP

PT--REN-LR------------------VLRFTRVLFGLNVSPFLLGAT-----ILFHLD

RMEDKK-------LA-------------------------NTIARNLYVDN-LIIATD-D

D------SE-A-MFKLYNKVKTVFNGLSMNIREFQSNDQSFTDLLDECDKT---------

---------------SESEVKVLGVKWSTKTDQITSSTTDIDILEN--------------

----------SRRTVSSAIASIYDP-----------MGLLV--PLLLPL-------KLFQ

RKLW----

>Mabel

------------------------------------------------------------

-------------------SNIIEKV---------SSEMNEVGI--IH------------

-----------------YLPHHEVITP-----NK-TTT------------------N-LR

IVYDAS-AHCKGMKSLNDVLYRG------------PIT----------LPDL-VGV---L

L----RFRTM------KNV-IIADVEKAFLQIEL---HPTDRNCTRFLWLKDI----RSP

VN--EEN-VS------------------CYRFQRVPFGIVSSPFLLSAT-----LNYHLE

TCKSK--------IA-------------------------LELRKNLYVDN-IIIPTK-G

T------NE---ALSNYKEIKIIFNEASMNVREFLSNDQEVNEKLPVQDRAE--------

---------------VSSIKKILGLNWYHKQDIIQVTLKP--------------------

----WFGKKLTKRIILQFVASQYDP-----------LGFIV--PILIRF-------KLFL

QTLW----

>Spirobel

------------------------------------------------------------

-------------------NGFVEEV---------NDV--QGRPGRTW------------

-----------------YLPHHTVLRE-----DK-SST------------------K-CR

IVFDGS-AQFRN-DSLNRQLDPG------------PPL----------QKDL-VQI---L

L----RFRRF------RVG-LQADISKMFLQIGL---HEKDRDVTRFLW----------R

ELGSQET-PR------------------IFRFKRVCFGLTCSPFLAMSV-----TRHHAL

NHLQG---FPQ--AA-------------------------NQVLENMYVDD-IVFSVD-E

D------DV---ARETVRQLVSLMKKGGFHLTKWVSNLSTVLADVPSED-----------

-----------IMGKNTNTSKILGIVWDSANDELAYSVLSDVDPWS--------------

--------RDTKRQLISVTAKVYDP-----------LGHLS--PYLIKA-------KVLF

QKLW----

>Tas

------------------------------------------------------------

-------------------CGVIEEV---------PQEELKPTFNIVH------------

-----------------YLPHQAVINP-----HK-TTT------------------K-I-

VVFDGS-SKSRGTKSINEAIMRG------------PVL----------LPNL-GGL---L

L----RFRSM------PIA-IIGDLEKAFLQVAL---NKPDRDAARFLWLSDI----NSS

PS--HSN-L-------------------VFRFARVPFGLNASPFLPTAT-----IKTHLD

SY--G---DVA--LA-------------------------EEIWKNIYVDN-VLISAR-T

T------KE---AFNKFESIKKLFEDARMNIREFLSNDATFNKTIPERDRHE--------

---------------ATN-AKLLGLRWNTTADYIQWKVSPPKQPMM--------------

----------TKRQLLSYIAAQYDP-----------TGHIP--PLFTPL-------KVIL

Q-------

>Hydra31

------------------------------------------------------------

-------------------SGVIEKV---------NNY--DSSIGEVH------------

-----------------YLPHRPVCRK-----DK-TST------------------K-VR

IVFDAS-STLSG-PSLNDCINAG------------PSL----------ATPL-FFI---L

L----RFRAN------KYA-FIADIEKAFLQIAL---DKNDRNYLRLIWFDDIYNINNSN

LF--SSA-LA------------------TYRICRVPFGVTSSPFLLNAT-----LIYHAE

RYCLN---DNN--IS-------------------------SKLLQSLHIDD-LISSCV-T

I------EE---GILFFNKCKDILKEGGFNLRKFESNSSAFEKQINGDNYER--------

---------------QTN-TRVLGLKWNKTEDSIIYSFEDLLNVASI-------------

--------VPTKREVMSFIASIYDP-----------IGLIN--PVVVTC-------KNLF

QRIC----

>Cer101

------------------------------------------------------------

-------------------AGIVERVW----PNMIGADDV------KY------------

-----------------YTPHRAVIKE-----TS-NTT------------------K-LR

IVLDAS-SPPGPEPSLNDCLYAG------------DNL----------VTPL-YGI---L

L----RGRIY------PYV-VVADIEKAFHQVRL---PTEFRNASLFLWLKDI-------

----TKP-PTPDN-------IC------TYRFTRIPFGVASSPWLLAAS-----ILHFLD

RNPNP--------LN-------------------------QRVRENLYVDN-CLIGTF-D

K------SE---ITEIIKNSKDIFMKMKMNLREYVTNSEEHMVAIEVED-QA--------

---------------KHRVIKLLGCEWDSHEDIDTLSVALAVLDIDH-------------

---------PTKRQVASKMAETFDP-----------LGLIT--PLIVSF-------KRLM

QNLW----

>Ninja

------------------------------------------------------------

-------------------KGYARRL---------QPEEVAVRSDRLW------------

-----------------YLPHFSVENP------N-KPG------------------K-VR

LVFDAA-AKVGG-TSLNSELDKG------------PQH----------YKPL-PAV---L

F----HFREG------AVG-VCGDIKEMFHQVLI---RPEDRCSQRFLW-RDG----DD-

----ERD-PD------------------VYEMNVMTFGAACSPSAAHYV-----KTMNAL

KYRDS---DPR--AV-------------------------KAITDYHYVDD-YVDSFA-T

E------SE---AISVSTRVKEIHKDAGFELCQFSSSSPTVETALGPGRVKSVGW-----

---------------GEAEEKILGMRWQVATDDFRFNVEYHRVPSS--------------

--VLSGDRVPTKREYLSLVMSTFDP-----------LGFLC--CLMVTA-------KLLL

REIW----

>Tamy

------------------------------------------------------------

-------------------SGYAEEA---------PSGTT---PGRTF------------

-----------------YLPHFAVVHPM----KK----------------------K-IR

IVFDAA-SRYEG-KSLNDALLPG------------PDL----------LQSL-FGV---L

L----RFRQG------PVA-VVADIKEMFLQIKI---REQDRDSLRFLW-RGE----DR-

----ASK-PR------------------EYRMTSVIFGAASSPATAIFV-----KNRNAE

EHQAS---HPE--AV-------------------------KAIVRNHYMDD-YLQSFA-T

I------EE---AIDTAATVDSIHKEAGFELRQWASNEQRVLDGLQREEASPTEV-----

-------------AIGSNEEKTLGLRWLIKDDALAFNGGLRNAPKE--------------

--IIEGNRVPTKREVTSAVMSTFDP-----------LGLIA--PLLIKG-------KRML

QDIW----

>Cubel

------------------------------------------------------------

-------------------KGYIRKL---------TDAEERADRDRVW------------

-----------------YLPIFVVTNP------N-KPG------------------K-LR

IVWDAA-AEVRG-ISLNSVLLKG------------PDQ----------VTSL-VDV---L

L----RFREY------RTA-VTGDIKEMFHQVRV---HLDDQHCQRFLW-NNG----AP-

----GST-PS------------------VYVMQVMTFGASCSPSCAQFV-----KNINAE

RFAED---SPA--AA-------------------------KAIINDHYVDD-MLSSVE-T

E------QE---AIKLASSVRDIHGQGGFEIRGWRSNSKAVLAALNSQECEDKNLSEASQ

F----------------STEKVLGMWWDSSTDTFTF-----RLPTKPDKDLLNGL-----

-------RVPTKKEVVRVLMSIFDP-----------LGLLA--NVLMFL-------KVLI

QEIW----

>Zebel

------------------------------------------------------------

-------------------AGYVAKI---------TAEEANKSNE-SW------------

-----------------YIPHHVVHHN------N----------------------K-DR

IVFNCS-FQHQG-QSLNSQLLPG------------PTL----------GPSL-LGV---L

L----RFRQH------AVA-VSGDIKGMFHQVRL---LPKDKQVLRFLW-RDL----CR-

----DRE-PD------------------IYEWQVLPFGTTCSPCCAIFA-----LQHHAQ

GHKGD---MPK--LV-------------------------NIVENSFYVDN-CLYSTP-T

A------KE---AKDVVDGLRQLLAEGGFDLRQWACNVPSVIQHLPAEA-------LSTN

SER-WLTKAS-----TDLQEPTLGLRWNCLNDSLGYNL----------------------

--RSAEPLEPTMRNMYKTLASQYDP-----------LGFII--PFTTRA-------KVLI

QDLW----

>Sinbad

------------------------------------------------------------

-------------------KGYIIEA---------SKEGFD-RDAVCW------------

-----------------YIPHHPVINP------K-KPG------------------K-LR

IVFDCA-AVYQG-FSLNNQFLRG------------PNT----------VNSL-FGV---L

L----RFRLG------NIA-LAADIEEMFLQVRI---PRQDRGAFRLLWWEDG----DM-

----KRT-AK------------------EYCLTVHPFGAVSSPFCANFA-----LKKTVD

IFGKE---FNRD-IQ-------------------------EVVDNSFYVDD-YLASID-N

V------QD---AIELAKTLGLLLRKGGFRLTKWISSCLQVLESIHPEE-------RAEA

VGEIDFE--------RLPTERTLGLFWNTMVDSFDFKVHIPKRPL---------------

----------TRRGILSSVASLYDP-----------LGLLA--PFILPM-------KQLL

QRLG----

>Saci6

------------------------------------------------------------

-------------------KSYIIEA---------SKEGFD-RDAVCW------------

-----------------YIPHHPVINP------K-KPG------------------K-LR

IVFDCA-AVYQG-FSLNDQLLRG------------PNS----------VNSL-LGV---L

L----RFRLG------NVA-LAADIEEMFLQVRI---PRQDRGAFRLLWWEDG----DI-

----KRT-AK-----------------------------------ARVS-----LLK---

------------------------------------------------------------

V------QT---IPRLELTAAVLAARMGSQLQS---------------------------

------------------------------------------------------------

------------------------------------------------------------

--------

>Kobel

------------------------------------------------------------

-------------------NGYAEKV----------PDGVTAKKGKEW------------

-----------------YIPHHGVYHP------R-KPN------------------K-IR

VVFDCA-AKCDG-TSLNDVLLPG------------PNL----------MNDL-KGV---L

M----RFREF------PVA-FASDIECMFYQVKV---PESQRDLLRFLWLPKG----DL-

----DQK-PK------------------VYRMAVHLFGAVSAPSCANYS-----LRRTGI

DNICN--------AR---------------------EETVNTLHNNFYMDD-GLKSVE-N

V------EQ---AIQLVKELKALCKEGGFNLTKWSSNEREVLREIPLDD-------RAKE

LMDLDLE------SSQLPTERTLGVLWNAETDEFQFKCQIKKTEV---------------

----------TRRSMLSEVSSIYDP-----------LGFIA--PLLIPA-------KSVL

QEMC----

>Purbel

------------------------------------------------------------

-------------------MGFAKKL---------EKDEMTMYKGPVH------------

-----------------YISHHAVIRP-----DN-KST------------------P-LR

IVFNSS-AIYKG-HCLNEYWKKG------------PDL----------LNNL-FGV---L

L----RFREH------PVA-ICADISKMYHRILI---PERDQHVHRFLW-RDM----NQ-

----EKE-PD------------------VYMMKVVTFGDKPAAAMAQIG-----VRLTAE

EGEAK---YPE--AA-------------------------AILKRNIYMDD-ICDSVE-T

E------DT---AKKRIAEVDELLEAGGFKVKGWQSNKP---------------LDGGNK

QEKMKL-------LEEIAEEKVLGVVWERHNDTFSYKVKLQETDKETEK-----------

---------LTKRKILGKVARIYDP-----------IGFAA--PIIIKA-------KTGL

QKLW----

>Suzu

------------------------------------------------------------

-------------------RGAAAKL---------SDSTIAEWTGPIW------------

-----------------YISHLIAPNP-----HS-VTT------------------P-VR

LVWNSS-QRFKG-VSLNDLLLKG------------PDV----------LNQI-RAV---L

L----RFRSG------VYA-AIGDVRKMYNSVWL---EDREVHLHRFLWRNSA-------

----EEE-IQ------------------EYAVTRVNIGDKPAGCIAQLA-----MRETAS

LPQFS--HLTA--ER-------------------------EVLHRNAYVDD-ILTSHN-D

L------KQ---LQATTKGVEDILKAGGFALKPWVFSGQSGRGDHIDRQ-------ETVQ

KDAFVL-----PNQMSDDDNKALGLGYIVKEEKLHVMVAINFSKRR--KKMHLGKDLEPQ

QVRFCTPSPLTRRELLSQVSGLYDP-----------VGLVA--PVKQKG-------AILV

RRAF----

>Gabel

------------------------------------------------------------

-------------------RRAAMKL---------TKDVLREWTGPVW------------

-----------------YISHLIAPNP-----HS-VTT------------------P-VR

LVWNSS-QKCRG-VSLNDLLLKG------------PDV----------LNSI-RAV---L

L----KFRRG------GFA-ALGDVKKMYNSVWL---EDQEVHLHRFLWRDSE-------

----EEE-LA------------------EYAVTRVNIGDKPAGCIAQLA-----MRETAN

LPQFS--HLEE--ER-------------------------RVLQEDSYVDD-LLTSHD-N

L------DQ---LKVITGNVEQILKAGGFELKPWVFTGQSRRESSGNDQ--------AVT

PRTVIL-----PNQLKEEDNKALGLGYTLEDDKLHVMVGINFSRRK--RKMRLGQDLRLE

QVRAQTPDPLTQRELLSQVSGLYDP-----------IGLTT--PVKQKG-------AILV

RRAF----

>CoDi4.4

------------------------------------------------------------

------------------IEKEMANV------------MP------AF--------KFLE

GD---ENLPVGCQ--KIDCHIIFDVK------LDLTR-------------------K-AR

YVAGGHMTEAPA----------------ALTYS--SVVSRES------VRIA-FMA-AAL

N---------------GLDILAADAQNAYLNADC---RES-----VYT------------

---IAGPEFG------------AARQGLRVLIVRALYGLKSSGAAWHAH-----LAQ---

-------TMSD--LKFRPCVADPD-VWLRPA-VKG-NGDKYYEYVLIYVDDILAVSEK--

----------------PDRIMETLSSL-YKFKE------------DPKTRKK--------

---------------YGPPDRYLGANVGKYKL----------------------------

P-GATKEHWYMSSD-----------------------------DYV----------KAAV

TNVE----

>CoDi3.1

------------------------------------------------------------

------------------IRKEMDTL----------ESMK------TF--------TIKD

KG---ERAPSDYK--RIPMWIIFDVK------MDFRR-------------------K-AR

LVAGGHVTDPPD----------------EDTYS--SVASRES------VRLG-FLL-ASL

N---------------NLDLVSVDIGNAYVNADC---REK-----VFS------------

---IAGPEFG-------------EHEGKVVLIAKALYGLKSSGAAWHSH-----LAQ---

-------NLRF--LGFVSSKADPD-MWFRKA-QRL-DKTEYYEYIISYVDDLTIVSAD--

----------------TKAILKALENIPYTLK--------------GGSA----------

------------------PRTFLGATTGVHTF----------------------------

NDG--TKAWYMSAK-----------------------------QYL----------TNAI

LNIE----

>CoDi2.4

------------------------------------------------------------

------------------ESIERAQL----------HEYE------TFVD-IGKATTLGK

HV---TNAPKGYK--KIRVHTVYDVK------HD---G-------------RH---K-AR

MVAGGHLTPVPT----------------ESVYS--GVVSLRS------LRIV-VFL-AEL

N---------------GLKLWGADIGNAYLEAKT---KEK-----VFI------------

---VAGPEFA-------------ELEGHILVINKALYGLRSSGLRWHER-----FAD---

-------TLRD--LGFTASKADSG-VWMR-------ENSGVYEYIAVYVDD-IAVAAQ--

---------------DPEGIIRQLKEK-YKYK--------------LKGVGP--------

------------------LQYHLGCTFERDK-----------------------------

-----DGTLSYH------------P-----------------RKYI----------SRMM

EQYE----

>CoDi4.1

------------------------------------------------------------

------------------IAKEMKNV------------MP------AF--------EFRD

-D---DRMPVGHK--EITCHMIFDIKA-----FSLQR-------------------K-AR

LVAGGHTTDPPK----------------DMTFA--SVVSRDS------VRIA-FLL-AAL

N---------------DLDILAADVQNAYLNAAT---KEK-----VWT------------

---RAGKEFG-------------SNAGRPVVIVRALYGLKSSGARWRDH-----MAA---

-------TLRE--ADYTSSRADPD-VWMRPA-TKP-DGFKYWEYVLVYSDDILVVSHD--

----------------PKRTMDFLESK-NTLK--------------NGTVAE--------

------------------P-----------------------------------------

------------------------------------------------------------

--------

>CoDi4.5

------------------------------------------------------------

------------------IKKEMQNV------------RI------AF--------DILP

DG---TTAPIGYQ--HVRCHMIFDVKM-----EDFRR-------------------K-AR

LVAGGHTTEAPP----------------TLTYA--SVVSRET------VRIA-LTM-AAL

H---------------GLPIMAADVMNAYVTAPN---KEK-----IWT------------

---TLGPEFG-------------SDCGKKAIIVRALYGLKSAGAAFRSH-----LGE---

-------CMRN--LGYKPCLADPD-LWMKPE-YDPSDSFKYWSYILCYVDDILVIHHQ--

----------------PEDVIKKIDKY-FPLK--------------PGSVGK--------

------------------PDMYLGTKLREITF----------------------------

TNG--EKAWAMSPS-----------------------------KYV----------QESV

SNCI----

>CoDi4.3

------------------------------------------------------------

------------------IRKEMKNV------------MP------AF--------KVLE

PN---ASKPVGYT--WIPCHMIFDIK------MDFTR-------------------K-AR

FVAGGHVTDPPS----------------SITYA--SVVSRDS------VRIA-LLI-AAL

N---------------SMQILGADAQNAYLNAPV---REK-----VYT------------

---TCGPEFG------------NSTEGRYAIIVRALYGLKSSGAAWRAH-----LAA---

-------TMEE--LMFTSCIADPD-VWLRPF-QNE-GGLKFYEYVLIYTDDFLCISHD--

----------------PKSILDTIGKH-FKLK--------------PESIKT--------

------------------PDTYLGANLGHFSL----------------------------

PDKPDKPRWSMSST-----------------------------NYV----------KQAV

ANVE----

>CoDi7.1

------------------------------------------------------------

------------------IKLEITSL----------IDLE------CF--------EFKP

SD---FSPGNEFQ--KTTLMTVFDVK------QDLRR-------------------K-AR

LVAGGHLVDALD----------------HDIYS--STVKGIS------VKLL-HVI-AHK

A---------------NLKQLCGDVANAYVNAYT---NEK-----VYA------------

---KAGPEFG------------SDLVGSIVIIRKALYGLRSSSERWHAH-----FAD---

-------TLRA--LQFKQSRYDKD-VWIRLG-----NESLFYEYVCTHVDDFMIVSKT--

----------------PEKIMESIKAI-YSVK----------------SVGP--------

------------------PDYYLGNDYKKDR-----------------------------

-----KGRWCIG--C---------------------------KKYL----------VEAI

KRVE----

>CoDi5.2

------------------------------------------------------------

------------------AANEMGRLSQGVQPHMPTGTNT-----IHF---IPH------

-----TEKPHDRK--ATYLKIVAAIKP-----HKAEKY------------------R-IR

FTVGGD--------RIEYSGPTS-----------TPTAALPA------IKILVNSV---I

STDGAHF-------------MTCDLKDFYLGTPL---PVY-----KYMRIPAK-------

----HIP-ACIMEQYKLAPL--VHNDNVLVEIRKGMYGLPHAGRIANDR-----LLQ---

-------HLAL--DGYHQAKHTPG-FFTHES---------RPISFSLVVDD-FGVKYV--

------------GKEHAEHLVQCLEKL-YTVTT---------------------------

---------------DWTGSLYCGLTFTW-------------------------------

--DYNARHVDMAMP-----------------------------GYI----------EKAL

QQFQ----

>CoDi5.3

------------------------------------------------------------

------------------AANEMGRLSQGVKPNMPTGTDT-----MHF---IPH------

-----TAKPHDRK--ATYLKIVAAIKP-----HKAEKY------------------R-IR

FTVGGD--------RIEYNGPTS-----------TPTAALPA------IKILVNSV---I

STKGARF-------------MTCDLKDFYLGTPL---PVY-----EYMRIPAV-------

----HIP-DCIMEQYKLAPL--IHKGNVLVEIRKGMYGLPHAGRIVNDR-----LID---

-------HLAL--DGYHQAKHTPG-FFTHET---------RPISFSLVVDD-FGVKYV--

------------GREHAEHLLHCLEKL-YTVTT---------------------------

---------------DWTGALYCGLTFTW-------------------------------

--NYEQRHVDMAMP-----------------------------GYV----------DKAL

QRFQ----

>CoDi5.1

------------------------------------------------------------

------------------AANEIGRLTQGNPPHSTTGSQT-----MHF---IAH------

-----NAIPPGRK--ATYLRIVASIRP-----QKSEPK------------------R-IR

FTVGGN--------LVQYPGKVS-----------TPTADITT------AKILFNSV---L

STPAAKF-------------MCIDIKDFYLGTPM---ARY-----EYMRIPVP-------

----DIP-PTIMAHYQLAPL--IHNNSITVEIRKGMYGLPQAGILAHDR-----LVE---

-------HLAA--HGYIKTKHTAG-LFRHVT---------RPIQFTLVVDD-FGVKYT--

------------GTEHAQHLIDTLQAL-YTITI---------------------------

---------------DWDGTRYLGLTLAW-------------------------------

--DYERRTLDMSMP-----------------------------DYI----------DQAL

TRFQ----

>CoDi5.4

------------------------------------------------------------

------------------YANELGRLTQG-IRDI-PGTNT-----MFY---ITK------

-----SEIPEDRRKDITYGRIVVVVRP-----QKKEQE------------------R-TR

LTVGGN--------LIDYPWEVA-----------TPTADLTT------AKLLFNSV---I

STPGAVF-------------VVLDCKNFYLQTPM---KRP-----EFMRLKLS-------

----LIP-QEIIDKYKLNDKV-DDRGWIYVRIEQGMYGLPQAGRLANEL-----LAK---

-------RLDK--EGYYQCQYTPG-LWRHKW---------RPITFSLVVDD-FGIKTV--

------------GLSHAKHLKNALEKH-YEVTT---------------------------

---------------DWKGNLFCGIKLTW-------------------------------

--DYKNRTVDLSMP-----------------------------DYI----------PKAL

TRFQ----

>CoDi5.5

------------------------------------------------------------

------------------CANEIGRLAQGIGKRI-DGTNT-----MFF---INK------

-----QDIPNDRFKDITYAKFVCDYRP-----GKSEPN------------------R-TR

LTMGGD--------RINYPGEVG-----------TPTADLLL------VKILFNSI---I

STHGARC-------------MTADIKNFYLNTPM---QRY-----EYLRIRLS-------

----DIP-QEVITEYGLLNKV-AADGYVYLEVRKGMYGLPQAGLLAQEL-----LEK---

-------RLAE--HGYFQSKIIPG-LWKHVT---------RPVCFTLVVDD-FCVKYI--

------------GKQHAEHLMGVLKQH-YEITE---------------------------

---------------DWKGEKYCGLTIDW-------------------------------

--DYNNKKVHLSMP-----------------------------GYV----------NKAL

KRFG----

>CoDi5.6

------------------------------------------------------------

------------------FGKEIGSLAQGDDLTGTKGTDT-----IVFLDRIGI------

-----KNIPKDRV--ITYARIVVDYRP-----QKEDPN------------------R-VR

ITAGGN--------LINYPGELT-----------TRTADLTT------AKLMWNSV---I

STDNARY-------------ACYDVKNFYLGTPL---DRY-----EYMRIPLH-------

----LIP-QHIIDQYDLTNK--ALNGFVYVEIRKGIYGLPQAGMLANKL-----LKE---

-------RLAP--HGYLEVRDTPG-LFIHKT---------RPIMFTLVVDD-FGVKYV--

------------GKEHADHLVSVLKRY-YTLSE---------------------------

---------------DWTGSLYCGVSLKW-------------------------------

--NYKERWVDISMP-----------------------------GYI----------KNAL

QRYN----

>Retrofit

------------------------------------------------------------

------------------METEYNALIKND----------------TW--------HLV-

------PYEKGQN--IIGCKWVYKIKR------K-ADG----------TLDRY---K-AR

LVAKGF-KQRYG---IDYE----------DTFS--PVVKAAT------IRII-LSI----

---AVS-R--------GWSLRQLDVQNAFLHGFL---EEE-----VYM------------

----QQP-PGFESSS---------KPDYVCKLDKALYGLKQAPRAWYSR-----LSK---

-------KLVE--LGFEASKADTS-LFF-----LNKGGILM--FVLVYVDD-IIVASS--

------------TEKATTALLKDLNKE-FALKD-------------LGDLH---------

--------------------YFLGIEVT--------------------------------

-----------------------------K--VSNGVILTQ-EKYA----------NDLL

KRVN----

>Koala

------------------------------------------------------------

------------------MDEEYQALVTNK----------------TW--------HLV-

------PPNK--N--IIDCKWVYKVKR------K-QDG----------TLDRY---K-AR

LVAKGF-RQRYG---IDYE----------DTFS--PVIKMTT------IRII-LAI----

---AVS-K--------GWFLRQLDVKNAFLHGIL---EEE-----VYM------------

----YQP-PGYEDKQ---------HPNYVCKLDKALYGLKQAPRAWFAR-----LSH---

-------KLNQ--LGFQESKADTS-LFF-----YNREGLTV--FLLIYVDD-IIVVSS--

------------KSEAIPILLQNLQQD-FALKD-------------LGNLH---------

--------------------YFLGIEVN--------------------------------

-----------------------------Q--SPNGIVLTQ-AKYA----------NDLL

RRSG----

>Hopscotch

GDTSPGPHHDAAGSSAAPEVIAATEQGDINARPKTRLQSGIRKKKIYTDGTVKYGCFTSS

GEPQNLAEALGDRNWKEAMDKEYYALMKNE----------------TW--------HLV-

------PPKKGIN--IIDCKWVYKIKR------K-ADG----------SLDRY---K-GR

LVAKGF-KQRYG---IDYE----------DTFS--PVVKAAT------IRTV-LSL----

---VAS-K----------------------------------------------------

------------------------------------------------------------

---------------------DTS-LFF-----YDKNGVTM--FMLVYVDD-IIVSSS--

------------SEKATSALLQDLNQE-FALKD-------------LGDLH---------

--------------------FFLGIEVK--------------------------------

-----------------------------K--VNDGLVLTQ-EKYA----------SDVL

RRVG----

>Melmoth

------------------------------------------------------------

------------------VDAEIGAMEKTN----------------TW--------EIT-

------TLPKGKK--AVGCKWVFTLKF------L-ADG----------NLERY---K-AR

LVAKGY-TQKEG---LDYT----------DTFS--PVAKMTT------IKLL-LKV----

---SAS-K--------KWFLKQLDVSNAFLNGEL---EEE-----IFM------------

----KIP-EGYAERKGI-----VLPSNVVLRLKRSIYGLKQASRQWFKK-----FSS---

-------SLLS--LGFKKTHGDHT-LFL-----KMYDGEFV--IVLVYVDD-IVIAST--

------------SEAAAAQLTEELDQR-FKLRD-------------LGDLK---------

--------------------YFLGLEVA--------------------------------

-----------------------------R--TTAGISICQ-RKYA----------LELL

QSTG----

>Vitico12

------------------------------------------------------------

--LETFAKAFQNLDWSKAMKEEIAALKRNS----------------DL--------GA--

-----------------------RAKA------K-XYG----------SIERH---K-AH

LVARGF-SQQYG---LDYD----------ETFS--PVAKLTI------------------

----------------------MDVKNAFLHKEL---DRE-----IYM------------

----NQL-MGFQSQG---------HPEYVCKLRKALYGLKQAPRAWYGK-----IAE---

-------FLTQ--SGYSVTHADSS-XFV-----KANGGK-------LAIEN---------

-----------------------LSVR-FEMKE-------------LGQLK---------

--------------------HFLGLEVD--------------------------------

-----------------------------C--THEGIFLCQ-QKCA----------KDLL

KKFG----

>Oryco11

------------------------------------------------------------

------------------MEDEIHMIEKNN----------------TW--------ELV-

------DRPRDRE--VIGVKWVYKTKL------N-LDG----------SVQKY---K-AR

LVAKGF-KQKPG---IDYY----------ETYA--PVARLET------IRTI-IAL----

---AAQ-K--------RWKIYQLDVKSAFLNGYL---DEE-----IYV------------

----EQP-EGFSVQG---------GENKVFRLKKALYGLKQAPRVWYSQ-----IDK---

-------YFIQ--KGFAKSISEPT-LYV-----NKTGTDIL--IVSLYVDD-LIYTGN--

------------SEKMMQDFKKDMMHT-YEMSD-------------LGLLY---------

--------------------YFLGMEVH--------------------------------

-----------------------------Q--SDEGIFISQ-RKYA----------ENIL

KKFK----

>Vitico11

------------------------------------------------------------

------------------XKEEIAAIEKNE----------------TW--------ELV-

------ELLEDKN--VIGVKWVFRTKY------L-ADG----------SIQKH---K-AQ

LVAKGY-AQQHG---VDYD----------DTFS--PIALFET------VRTL-LAL----

---AAH-M--------HWCVYQFDVKSAFLNGEL---VEE-----VYV------------

----SQP-EGFIVPG---------KEEHVYRLKKTLFGLKQAPRAWYSK-----IDS---

-------YFVE--NGFERSKSDPN-LYL-----KRQ-------------DD-MIYMGS--

------------SSFLINEFKACMKKK-FEMSD-------------LGLLH---------

--------------------FFLGLEAE--------------------------------

-----------------------------D--D------TE-RADA--------------

RRFRSLVG

>Araco

------------------------------------------------------------

------------------MDEEIKSIQKND----------------TW--------ELT-

------SLPNGHK--AIGVKWVYKAKK------N-SKG----------EVERY---K-AR

LVAKGY-SQRAG---IDYD----------EVFA--PVARLET------VRLI-ISL----

---AAQ-N--------KWKIHQMDVKSAFLNGDL---EEE-----VYI------------

----EQP-QGYIVKG---------EEDKVLRLKKALYGLKQAPRAWNTR-----IDK---

-------YFKE--KDFIKCPYEHA-LYI-----KIQKEDIL--IACLYVDD-LIFTGN--

------------NPSMFEEFKKEMTKE-FEMTD-------------IGLMS---------

--------------------YYLGIEVK--------------------------------

-----------------------------Q--EDNGIFITQ-EGYA----------KEVL

KKFK----

>Poco

------------------------------------------------------------

------------------MNEELRMIEKNQ----------------TW--------KLV-

------DMSEHKK--PIGVKWVYRTKL------N-ADG----------TINKH---K-AR

LVVKGY-AQIFG---VDFS----------ETFA--PVARLDT------IRML-LAV----

---AAQ-K--------GWKIFQLDVKSAFLNGYL---QEG-----IFV------------

----EQP-KGFVVRG---------EEEKVYLLKKALYGLKQAPRAW-SR-----IDE---

-------HLLK--LDFKKSLSEST-LYI-----RNSNSDYI--VVSLYVDD-LFVTGN--

------------NQSMIDNFKAEMMKV-FEMTD-------------LGEMA---------

--------------------YFLGMEVQ--------------------------------

-----------------------------Q--NQHGIFICQ-QKYA----------KEIL

KKFK----

>Oryco12

------------------------------------------------------------

------------------MQDELDAIVDND----------------TW--------SLT-

------DLPHGHR--AIGLKWVYKLKR------D-EQG----------AIVRY---K-AR

LAAKGY-VQRQG---GGLRRG-------LHTCR--P------------------------

----AG-I--------DWQVHHMDVKSAFLNGKL---LEE-----VYV------------

----SQP-PGFVDDN---------HKNKVYRLHKALYGLRQAPRAWNAK-----LDS---

-------SLLS--FGFHRSSSEHG-VYT-----RTRGGRRL--TVGVYVND-LIITGD--

------------HDDEIRSFKGEMMKL-FKMSD-------------LGHSDTT-------

-------------SASRLPWTVMGSRWGK-------------------------------

----------------------------LH--MPARFLRGQ-D-----------------

--------

>Endovir11

------------------------------------------------------------

------------------MEEELEEFSRHQ----------------VW--------DLV-

------PRPPQVN--VIGTKWIFKNKF------D-EVG----------NITRN---K-AR

LVAQGY-TQVEG---LDFD----------ETFA--PVARLEC------IRFL-LGT----

---ACG-M--------GFKLHQMDVKCAFLNGII---EEE-----VYV------------

----EQP-KGFENLE---------FPEYVYKLKKALYGLKQAPRAWYER-----LTT---

-------FLIV--QGYTRGSVDKT-LFV-----KNDVHGII--IIQIYVDD-IVFGGT--

------------SDKLVKTFVKTMTTE-FRMSM-------------VGELK---------

--------------------YFLGLQIN--------------------------------

-----------------------------Q--TDEGITISQ-STYA----------QNLV

KRFG----

>SIRE14

------------------------------------------------------------

------------------MQEELEQFKRNE----------------VW--------ELV-

------PRPEGTN--VIGTKWIFKNKT------N-EEG----------VITRN---K-AR

LVAQGY-TQIEG---VDFD----------ETFA--PVARLES------IRLL-LGV----

---ACI-L--------KFKLYQMDVKSAFLNGYL---NEE-----VYV------------

----EQP-KGFADPT---------HPDHVYRLKKALYGLKQAPRAWYER-----LTE---

-------FLTQ--QGYRKGGIDKT-LFV-----KQDAENLM--IAQIYVDD-IVFGGM--

------------SNEMLRHFVQQMQSE-FEMSL-------------VGELT---------

--------------------YFLGLQVK--------------------------------

-----------------------------Q--MEDSIFLSQ-SRYA----------KNIV

KKFG----

>Opie2

------------------------------------------------------------

------------------MQEELEQFKRNE----------------VW--------ELV-

------PRPEGTN--VIGTKWIFKNKT------N-EEG----------VITRN---K-AR

LVAQGY-TQIEG---VDFD----------ETFA--PVARLES------IRLL-LGV----

---ACI-L--------KFKLYQMDVKSAFLNGYL---NEE-----VYV------------

----EQP-KGFADPT---------HPDHVYRLKKALYGLKQAPRAWYER-----LTE---

-------FLTQ--QGYRKGGIDKT-LFV-----KQDAENLM--IAQIYVDD-IVFGGM--

------------SNEMLRHFVQQMQSE-FEMSL-------------VGELT---------

--------------------YFLGLQVK--------------------------------

-----------------------------Q--MEDSIFLSQ-SRYA----------KNIV

KKFG----

>TSI9

------------------------------------------------------------

------------------MHEELENFERNQ----------------VW--------VLV-

------DPPPSCK--PIGTKWVFKNKQ------G-EDG----------HVVRN---K-AR

LVAQGF-CQKEG---IDYG----------ETFA--PVARLEA------IRIL-LAM----

---AAS-H--------GYKLYQMDVKSAFLNGFI---EEE-----VYV------------

----KQP-PGFEHPN---------FPDRVFKLQKALYGLKQAPRAWYAR-----LKT---

-------FLLK--NGFKMGSVDKT-LFL-----LRQGNDTL--IVQIYVDD-IIFGGS--

------------SHVLMKKFADVMSKE-FEMSM-------------MGELK---------

--------------------FFLGLQIK--------------------------------

-----------------------------Q--TSEGTFVHQ-GKYT----------KDVL

QKFA----

>ToRTL1

------------------------------------------------------------

------------------MQEELHQFERSK----------------VW--------YLV-

------PRPKGRT--IIGTRWVFRNKL------D-ENG----------VITRN---K-SR

LVVQGY-NQEEG---IDYD----------ETFA--PVARMEA------IRIL-IAF----

---AAF-M--------GIKLYQMDVKSAFLNEDL---KEE-----VYV------------

----KQP-PGFEDAE---------LPNHVFRLNKALYGLKQAPRAWYER-----LSK---

-------FLLK--NGFKRGKIDNT-LFL-----LKREQELL--IIQVYVDD-IISGTT--

------------SEHLCEEFSSLMGRE-FEMSM-------------MGELT---------

--------------------FFLGLQIK--------------------------------

-----------------------------Q--SSNGTSICQ-EKYI----------KELL

KKFN----

>Fourf

------------------------------------------------------------

------------------VRSEMESIMSNG----------------TW--------EVV-

------DRPYGCQ--PIGCKWIFKKKL------R-PDG----------TIERY---K-AR

LVAKGY-TQKEG---EDFF----------DTYS--PVARLTT------IRTL-IAV----

---AAS-Y--------GLIIHQMDVKTAFLNGEL---DEE-----IYM------------

----DQP-EGFIADG---------QENKVCRLIKSLYGLKQAPKQWHEK-----FDN---

-------TLTA--AGFVVNESDTC-VYY-----RYGGGESV--MLCLYVDD-ILIFGS--

------------NLNVIEEVKNLLSSN-FEMKD-------------LGEAD---------

--------------------VILNIKLVR-------------------------------

-----------------------------K--ADGGVTLLQ-SHYV----------EKVL

SRFG----

>Batata

------------------------------------------------------------

------------------MQEEMNSLYVND----------------TF--------ELV-

------KAPKNRK--ALKNRWVYRVKH------E-EGT----------SVPRF---K-AR

LVVKGF-SQKKG---IDFD----------EIFS--PVVKFSS------IRVV-LGL----

---AAR-L--------DIEIEQMDVKTAFLHGDL---DEE-----IYM------------

----EQP-EGFKVKG---------KEDYVCRLKKSLYGLKQAPRQWYKK-----FTS---

-------VMSK--HGYKKTSSDHC-VFV----NRYSDDDFV--ILLLYVDD-MLIVGR--

------------NASRIQELKQELSKS-FSMKD-------------MGPAK---------

--------------------QILGMKIIR-------------------------------

----------------------------DR--QNKKLWLSQ-EKYI----------EKVL

ERFH----

>Sto4

------------------------------------------------------------

------------------MEDELESMRMNK----------------VW--------DLE-

------VIPHGAK--TVGCKWVYKTKR------D-SRG----------NIERY---K-AR

LVAKGF-TQREG---IDYH----------ETFS--PVSTKDS------FRII-MAL----

---VAH-F--------DLELHQMGVKTAFLNGEL---EEN-----VFM------------

----AQP-KGFVVSG---------KEHMGCHLRRSIYGLKQASRQWYIK-----FDQ---

-------TIRK--FGFEENKEDNC-IYA-----KFRKGKYI--FLVLYVDD-ILLASS--

------------DKDLLAETKGFLSSN-FDMKD-------------MGEAS---------

--------------------YVLGIEIHR-------------------------------

----------------------------DR--QKGVLGLSQ-KSYI----------ENVL

KRYN----

>Tork4

------------------------------------------------------------

------------------MGDEMESLHKNQ----------------TW--------DLV-

------IQPSGRK--IITCKWVFKKKE------GISPA----------EGVKY---K-AR

VVARGF-NQREG---VDYN----------EIFS--PVVRHTS------IRVL-LAI----

---VAH-Q--------NLELEQLDVKTAFLHGEL---EEE-----IYM------------

----TQP-DGFQVPG---------KENHVCKLKKSLYGLKQSPRQWYKR-----FDS---

-------YMVK--LGYTRSSYDCC-VYY----NRLNDDSFI--YLVLYVDD-MLIAAK--

------------KKYDIQKLKGLLSAE-FEMKD-------------LGAAR---------

--------------------KILGMEIIR-------------------------------

----------------------------DR--ERRKLFLSQ-RSYI----------QKVL

ARFG----

>Tto1

------------------------------------------------------------

------------------MQDEIKSLHENK----------------TF--------ELV-

------KLPKGKR--ALKNKWVFKMKH------D-EHN----------SLPRF---K-AR

LVVKGF-NQRKG---IDFD----------EIFS--PVVKMTS------IRTV-LGL----

---AAS-L--------NLEVEQMDVKTAFLHGDL---EEE-----IYM------------

----EQP-DGFQQKG---------KEDYVCRLRKSLYGLKQAPRQWYKK-----FES---

-------VMGQ--HGYKKTTSDHC-VFA----QKFSDDDFI--ILLLYVDD-MLIVGR--

------------NVSRINSLKEQLSKF-FAMKD-------------LGPAK---------

--------------------QILGMRIMR-------------------------------

----------------------------DR--EAKKLWLSQ-EKYI----------EKVL

QRFN----

>RTvr2

------------------------------------------------------------

------------------MQDKMKSLYDNH----------------TC--------DFV-

------NLPKGKR--ALENRWIFRVKQ------E-SNS----------TSTRY---K-AR

LVVKGF-RQRKG---VDFN----------EIFS--SVVRMTS------I-TV-LSL----

---AAT-L--------DLEVKQMDVKTTFLHGDL---EEE-----IYM------------

----KQP-DDFLIEG---------KEDYVCRLRKSLYGLKQAPRQWYKK-----FES---

-------VMCE--QGYKKTTFDHC-VFV----RKFSENDFI--ILLLYVDD-MLIVGK--

------------DVSKIDRLKKQLGES-FAMKD-------------MGAAK---------

--------------------KILGISITR-------------------------------

----------------------------DR--KEKKLWLSQ-KHYI----------QKVL

QRFQ----

>Tnt1

------------------------------------------------------------

------------------MQEEMESLQKNG----------------TY--------KLV-

------ELPKGKR--PLKCKWVFKLKK------D-GDC----------KLVRY---K-AR

LVVKGF-EQKKG---IDFD----------EIFS--PVVKMTS------IRTI-LSL----

---AAS-L--------DLEVEQLDVKTAFLHGDL---EEE-----IYM------------

----EQP-EGFEVAG---------KKHMVCKLNKSLYGLKQAPRQWYMK-----FDS---

-------FMKS--QTYLKTYSDPC-VYF----KRFSENNFI--ILLLYVDD-MLIVGK--

------------DKGLIAKLKGDLSKS-FDMKD-------------LGPAQ---------

--------------------QILGMKIVR-------------------------------

----------------------------ER--TSRKLWLSQ-EKYI----------ERVL

ERFN----

>V12

------------------------------------------------------------

------------------MHEDMKSLHKNN----------------TY--------ELM-

------ELPKGKR--ALKNKWVLKRKP------E-PNR----------SQPRY---K-AR

LVVKGF-SQKKG---IDFE----------EIFS--PVVKMSS------IRVV-LGL----

---AAS-M--------NLEIEQLDVKTAFLHGDL---EEE-----IYM------------

----EQL-EGFTIKG---------KEHLVCRLKKSLYGLKQAPRQWYKK-----FDS---

-------FMVE--HGYDRTASDHC-VFV----KKFSDGEFI--ILLLYVDD-MLIVGR--

------------DTGKIDKLKKELSKS-FEMKD-------------LGSTS---------

--------------------QILGIKISR-------------------------------

----------------------------DR--TNGKLWLSQ-ESYI----------EKVL

DKFN----

>Humnum

------------------------------------------------------------

------------------VQDELLSFEKNS----------------AW--------ELV-

------DVPKDGT--IVQCKWVLRKKY------D-SEN----------KV-HY---R-AR

LVAKGF-TQKHG---VDYT----------ETFS--PVVRHTT------LRLL-FAL----

---SVK-L--------DLNVTHLDVKTAFLNGDL---EET-----IYM------------

----KLP-DCYNSSS--------SSDCKVLKLKKAIYGLKQASRAWNKK-----VDN---

-------CLVS--NGYKRSKIEPC-MYV-----KDISKCKI--IVTVYVDD-FFIFSN--

------------DKVETENLKQILSNK-FSIKD-------------LGQVK---------

--------------------QCLGMNVTF-------------------------------

----------------------------NR--EKGYVTLSQ-ETYV----------DQLL

SKFQ----

>Mtanga

------------------------------------------------------------

------------------MEEEIKSLHENA----------------TW--------EIA-

------SLPKDRK--AVGSKWVFKRKM------D-GDG----------KIVQY---K-AR

LVAKGF-SQVYG---ADYD----------EVFA--PVAKQTT------FRTL-LSI----

---AAR-R--------KLIVKHVDVKSAYLYGDL---AET-----IYM------------

----KQP-TGF-EIG---------SKNDVCLLKKSLYGLKQAGRVWNQT-----ITE---

-------VLRS--LGFHSSEADPC-LFV-----KNKRDRWS--FILLYVDD-MLVACS--

------------EDREYEDIENTLKRH-FKITT-------------LGDVR---------

--------------------NYLGIRIER-------------------------------

-----------------------------G--QNGEYLLDQ-ASYI----------RRIA

KRFG----

>Copia

------------------------------------------------------------

------------------INTELNAHKINN----------------TW--------TIT-

------KRPENKN--IVDSRWVFSVKY------N-ELG----------NPIRY---K-AR

LVARGF-TQKYQ---IDYE----------ETFA--PVARISS------FRFI-LSL----

---AIQ-Y--------NLKVHQMDVKTAFLNGSL---KEE-----IYM------------

----RLP-QGISC-----------NSDNVCKLNKAIYGLKQAARCWFEV-----FEQ---

-------ALKE--CEFVNSSVDRC-IYIL---DKDNINENI--YVLLYVDD-VVIATG--

------------DMTRMNNFKRYLMEK-FRMTD-------------LNEIK---------

--------------------HFIGIRIE--------------------------------

-----------------------------M--HEDKIYLSQ-SAYV----------KKIL

SKFN----

>Koco

------------------------------------------------------------

------------------IKTELNAHDINN----------------TW--------TIM-

------QKPENKN--IVDSRWVFSVKY------D-ELG----------APIKY---K-AR

LVARGF-TQKYQ---VDYD----------ETFA--PVARIAS------FRLL-LSL----

---AVQ-Y--------NLKVHHMDVKTAFLNGTL---KEE-----IYM------------

----QPP-QGVAC-----------NVGYVCKLNKAIYGLKQAARCWFQV-----FEQ---

-------ALKE--CSFVNSPVDRC-IYIL---EKGDIKKNI--YVLLYVDD-VVIATG--

------------DMEGMINFKSFLNKK-FRMTD-------------LDEIR---------

--------------------HFIGIRIE--------------------------------

-----------------------------T--YEDKICLSQ-AAYV----------ERIL

NKFN----

>Yokozuna

------------------------------------------------------------

------------------IKTELNSQVKLE----------------TW--------EEA-

------TLPMGSK--AIDTKWVFRTKQ------N----------------GTK---R-AR

LVAKGF-QQQ------NND----------NHYA--PVAKLST------IRLM-MSL----

---AVQ-L--------DLSLKQLDVPTAFLNGKL---NDN-----VYI------------

----KCP-KGMEI-----------SEGKVLKLKRALYGLKEAPKCWNQR-----FHN---

-------FVTQ--KGFVQSQHDLC-LYG---------KGKI--WILLYVDD-ILYLGN--

------------SNEMIKE----LEKE-FKVKN-------------LGEVH---------

--------------------QYLGLEVT--------------------------------

-----------------------------R--TEDSLEIRQ-TEII----------KRLL

EKYH----

>Tricopia

------------------------------------------------------------

------------------ITSEITSILKND----------------TF--------ELV-

------DRPKEGN--VIGSV-ILRNKF------K-SNG----------MLERR---K-AR

LVAQGF-SQKPG---IHFN----------ETFA--PVTRFSS------IRLL-AAL----

---AVE-H--------GMRIQQFDVTTAYLNGEI---EEE-----IFM------------

----EPP-KNFEQIL---------SGDKVCRLKKSLYGLKQAGRNWYEK-----LSS---

-------TLKE--TGAVPTSSDPC-FFR-----LGSGEDIT--FIAVYVDD-ILVASR--

------------NRNMISRVKNCLSSR-FDLKS-------------LGDVK---------

--------------------SCLGVEFD--------------------------------

-----------------------------Q--RDGQVTMHQ-RGYI----------NEIL

ARFG----

>Hydra12

------------------------------------------------------------

------------------MEDEISSLQENN----------------TF--------TLT-

------TLPEGKH--AVGGRWVYSIKS------N--ID----------ETETY---K-AR

YVAKGY-SQVLG---VDYI----------ETFS--PTANITS------IRAL-MQM----

---AAQ-Y--------DLELHQMDVKTAYLHAPI---DCK-----IYM------------

----EQP-KGFEVKSNR-------GDKLVCKLNKSLYGLKQLGRNWNKI-----LHV---

-------YLTE--NDLIQNLADYC-VYS-----KRSGKDRV--IIIIWVDD-LIIAAS--

------------DNSLLKDVKEMLTSK-FKMKD-------------LGKLK---------

--------------------HFLGIDFD--------------------------------

-----------------------------Q--SKGTVRVNQ-KRYI----------LRIL

ERFN----

>Hydra11

------------------------------------------------------------

------------------MDEEIQSLTNND----------------TF--------IIT-

------ELPANKK--VVGGRWIYTIKG------N--NN----------KI-IY---K-AR

YVAKGY-NQIQG---IDYL----------ETFS--PTARMES------VRIL-MQI----

---SAQ-Y--------NLILHQMDVKSAYLHAPI---EHE-----IYV------------

----NQP-PGYE-KTHN-------NKQLVWKLNKSLYGLKQSGRNWQNV-----LSD---

-------FLKE--IQFIQSNADNC-VFV-----RRSNTEVA--MILVWVDD-IIIAAN--

------------SNELLIKVKKKLSKR-FKMKD-------------LGPLT---------

--------------------SFLGIQFK--------------------------------

-----------------------------S--TSNCVTMNQ-SDYL----------QNVL

QKFG----

>1731

------------------------------------------------------------

------------------MGLEYKALLANE----------------TW--------KLA-

------DLPRNRR--CVACKWVYSLKR------D-VSG----------RIERF---K-AR

LVAKGC-SQKFG---VDYF----------ETFS--PVCRLES------VRLI-LAL----

---AAE-M--------QLYLHHMDVCTAYLNSEL---KDT-----VYM------------

----KQP-QGFTDAA---------NPDQVLLLRKAIYGLKQSGREWNSK-----LDG---

-------VLKD--LGFKACNHEPC-LYQ-----QSGQGNLM--LILVYVDD-LILACQ--

------------SREDMEDLKAKISES-FECTD-------------KGPLH---------

--------------------LFLGMEVQR-------------------------------

----------------------------DG--DLGEITLGH-SQYI----------KELL

RDYG----

>Xanthias

------------------------------------------------------------

------------------MQKEYDALVSNN----------------TW--------TLC-

------DLPPGQK--AIGSKWVFRVKR------D-KEG----------NIQKF---K-SR

LVAQGC-GQKMG---VNYS----------ETFS--PVIRYET------IRML-FAI----

---AAE-K--------QLCMHQVDISNAYLNGRL---QEE-----VYM------------

----RQP-QNFIDEK---------HPNKVLKLQKAIYGLKQSGRVWNDT-----LDE---

-------VLKS--IGFKRSKNEAC-LYA-----KQQQQQHS--YIAVYVDD-LIIISY--

------------DENEISAIKRKIANK-FDIHD-------------GGQLN---------

--------------------YFLGMEIQR-------------------------------

----------------------------ES--TRGSISLCQ-KQFI----------INLL

DKYG----

>pCretro6

------------------------------------------------------------

------------------MQREYDQLTKLG----------------CW--------DLV-

------DLPAGRK--AIGCKWVYRIKR------N-FSG----------AIIKY---K-AR

LVAQGF-SQVPG---VDYD----------ETYA--PVMRPES------LHIL-AAI----

---AVI-L--------NLEWDIENAVGAYLNSQL---KLT-----IYM------------

----RQP-EGFDD-----------GSGRVCKLNLALYGLKQSGREWNLL-----LDE---

-------FLRG--IGFRASSVDPC-VYL-----RIDEGSPT--FLAVHVDD-FSLFAK--

------------TREIMDKLKGELSSR-FEMTD-------------LGPVR---------

--------------------QILGYEVIR-------------------------------

----------------------------ER--DQRTLMLRQ-AAYI----------RKVL

DRFN----

>pCretro3

------------------------------------------------------------

------------------MDAEMAQLEANG----------------TW--------KKG-

------ELPPGRK--AIGSKWVFAIKR------H-QDG----------SIDKY---K-AR

LVAQGF-SQIAG---QDYF----------DTFS--PVVRQET------FRVA-TAL----

---AAT-E--------NLDSDALDIVGAYLHGPL---EEE-----IYM------------

----RQA-PGYDD-----------GSGQVYVLIKALYGLKQAGRVWNHL-----LNH---

-------VLTSL-MGWTRSEADPC-LYF------KHEGKLN--MALVHVDD-TALYGE--

-----------R--SILDRFKADVAKH-FAITT-------------NGTLS---------

--------------------SFVGLQVT--------------------------------

-----------------------------R--KNGAISILQ-TRYL----------ETIL

ERFG----

>PyRE1G1

------------------------------------------------------------

------------------LEDERQSMITHK----------------VW--------TKK-

------KAPPGAR--RQGTRVIFECKA------D-QRG----------VLQRR---K-CR

LVGRGD-RQKPG---NDYL----------QSWAAMPAA--AT------TRAF-FAT----

---AAA-R--------GWTVHHIDVKTAYLYAPM---DVE-----VYI------------

----VIP-EGFE------------GAGKDALLQQAMYGTKQAGNLWGKH-----LDG---

-------KLTQ--RGGVQSKADKC-LYT-----FAMKGTTV--YVNVHVDD-ILPGGP--

------------DAEAVADVKRRIARH-FECRD-------------MGEVT---------

--------------------SYLGMQVEW-------------------------------

----------------------------DK--AAGTVTLSN-PRHT----------ADLL

KEYE----

>Osser

------------------------------------------------------------

------------------MDEELASIRANE----------------VW--------RLE-

------IPPKSVR--PLPVKWVFSLKK------D-EHG----------EIVRY---K-AR

LVAKGF-AQVEG---RDYE----------EVWA--PVSKHTT------LRAL-LSV----

---AAR----------DLELHQLDVKTAFLNGEL---EET-----VYI------------

----QQP-PGYVE----------GEPYLACKLEKALYGLKQAPRAWYAR-----LRS---

-------ELEA--MNFTVSQADPG-LFY-----RDVLGERV--YLLVYVDDLLLIAAK--

------------DINIVRQLKDKLKSI-LMCVT-------------WVRPV---------

--------------------CFLGFEIER-------------------------------

----------------------------NR--AERTMKVSQ-KRYA----------KGLV

EKYG----

>CoDi6.1

------------------------------------------------------------

------------------VVKEVNGHIENN----------------HW--------QLVP

R----SEVPPDAE--VVPSVWAMRRKR------NLTTN----------EITKY---K-AR

LNMHGG-KQTYG---VNYY----------ETFA--PVVSWFG------IRLL-VVF----

---AIV-F--------KWSLRQVDFVMAYTQAPI---EMD-----MYM------------

----ELP-AGLSTKH-------GDSKSHVLKLLANLYGQKQAGRVWNEY-----LVG---

-------KLRS--IGFEQSKVDDC-IFY-----RGD------VVFIVYVDDGMFLGRC--

------------DRQLTSIIKELVGLG-LDIED-------------QGHPA---------

--------------------DYVGVNIRK-------------------------------

-----------------------------L--QDGSYEFTQ-RAII----------DSVI

ADVG----

>CoDi6.7

------------------------------------------------------------

------------------MQEEVQAHTENK----------------LW--------ELYP

R----RLVPQGTP--IIPAVWSMKRKR------RISTR----------EVYKW---K-AR

LAFDGS-KQIHG---VNFW----------ETYA--PVASWPT------IRYI-LTL----

---ALI-N--------RWHMQQIDFVLAYTQAEA---ECE-----MFM------------

----KIP-KGFTVEH-------DNSEEYVLRIKKNYYGLKQAGRVWNQH-----LVS---

-------KLQE--CGFKQSEHDQC-LFY-----RGRS------VYVLYTDD-SILAGP-D

L-----------EE-LEQIKIDMANSG-LKLTS-------------EPGVS---------

--------------------DFLGVKID--------------------------------

-----------------------------R--KGDEIHLTQ-PHLI----------NSIL

DDLR----

>CoDi6.6

------------------------------------------------------------

------------------MNKELQQLHDKG----------------TY--------EIVA

K-----DTVQGK---IIPTTWVFKRKR-----L--PDG----------TIYKY---K-AR

LCIRGD-LQAPQ---LDRN----------DTYA--PVASWDT------IRLM-FSL----

---CVQ-H--------GIKSRQIDFANAFVQADR---DEP-----IYL------------

----SLP-PGFSQ----------TSSTHCMKVTKSLYGDARAPRMWYDH-----LTT---

-------ALVA--LGFTPSPIDPC-LFLRKDC-----------IFLFWVDD-AIICSH--

------------DDSTIVSVIDDLRQRNFNIDN--DT--------GVGSME---------

--------------------NYLGIKLAP-------------------------------

---------------------------DNS--ISGSIHLTQ-PHLI----------SRIV

DSTC----

>CoDi6.4

------------------------------------------------------------

------------------AAKEMESIRDRN----------------VL--------GGLI

KL---SSLPTGTR--VLGTRWIFKWKQ------D---G---------FDHDKYT-EK-AR

LVVKGY-EQISG---VDYT----------ESYS--PVANETT------VNLG-IALGLYY

K-HVLK-Q--------DWIEHVIDVETAFLHAERLNSDKK-----TYI------------

----KVP-SGFEELTGI-----KTGTDDVIELTGVLYGEVDAPLAWAIT-----FKK---

-------ILTK--IGFKQSLIDPC-LYI-----MQDKKMTLEVLMMVHVDD-CKITGS--

------------RE-KVMWIKKEIKKH-VPIKD-------------LGELR---------

--------------------KFLGVNYKA-------------------------------

--------------------------GTDK--IGPYIGVDM-TEYV----------EEII

RDYE----

>CoDi6.3

------------------------------------------------------------

------------------IRKEFRDMVKRK----------------VW--------RRVK

K----SSIPSNRR--CVKSKWVFKIKR------N----------------GVF---R-AR

LVACGY-SQIPG---VDFS----------ESYS--PVANDIT------IRLL-LVA----

---MIL-F--------GLSAKIVDVETAFLYGEL---EEE-----VYM------------

----ENP-EGLED----------SNDDEALLLLTTIYGLVQAARQYYKK-----ARG---

-------ILRK--IGFTGGDVDPC-LFV-----KKSSLGIV--FIALYVDD-NLLVGH--

------------PKAIECAIEQMKRHG-LILKV-------------EDDLK---------

--------------------DYLSCEIQF-------------------------------

----------------------------SK--DKTKAWLGQ-PHLI----------SNLM

SKFG----

>CoDi6.2

------------------------------------------------------------

------------------IMKEWNDMKKRN----------------VW--------IVQK

R----CDMPKDRR--CVKSKWVFKLKR------N----------------GVF---R-AR

IVACGY-SQIPG---VDFE----------ESYS--PVMNDIT------LRIL-LVI----

---WIV-M--------TLKAIIADVETAFLYGKL---LEV-----IFM------------

----ECP-PGMMG----------TTKDDVLRLLMCIYGLVQAAARYYAY-----MAK---

-------TLRS--MGFKGGDVDPC-LFV-----KWINGRVC--FVGLYVDD-NLIIGH--

------------PELVDDTIKQLRQKG-LILKI--------------SDLD---------

--------------------DYLSCHIVL-------------------------------

----------------------------SK--DKRRAWLGQ-PHLI----------ASIV

NKFG----

>CoDi6.5

------------------------------------------------------------

------------------AVNEIMNFISRG----------------SW--------KKVP

R---SQARKSGKT--ILPTKWVFKKKD------E-QDG-----------TTRY---K-SR

IVTKGF-MQIPG---VDYS----------ESFS--PVANDTS------VRIG-IA-----

K-TLAN-N--------DWVIEVIDIEAAFLEGQL---DRD-----TYI------------

----EWP-EGMVKLGFITA---DESERACIQLTKSMYGNVDAARRFYIE-----YKK---

-------HLTGVPMGMESSEVDPCVFYR-----KRGNKVCL--VAMTHVDD-TILFGP--

------------KQE-IEWFKVGVKKR-FNYSD-------------LGKLK---------

--------------------KHLGVWYEWK------------------------------

-------------------------ENNSE--ERHLIG-SM-PKLV----------KEII

ESFE----

>Tse1

------------------------------------------------------------

------------------YDKEINQLMKMN----------------TW--------DNNQ

LYDA-KDIPS-KK--IINSMFIFTTKR------D-GTR------------------K-CR

FVARGD-QQHP------------------STYD--ENAIANT------VHHY-ALM-TSL

S-LALD-S--------KKYIVQLDISSAYLYADL---SEE-----LYI------------

----RTP-PHMS------------KRGKVMRLNKSLYGLKQSGANWYNT-----IKE---

-------YLIKK-CKLQEVKGWSC-VF------RNKD---L--TVCLFVDD-MVVTSS--

------------NRELANKFIDTLKKK-FETKVVN-----------TGEIDNQ-------

---------------GYAYYDILGLEIEY-------------------------------

-----------------------------K--FGSKMKIG------------------ME

KLQ-----

>Ty1B

------------------------------------------------------------

------------------YHKEVNQLLKMK----------------TW--------DTDK

YYDR-KEIDP-KR--VINSMFIFNRKR------D-GTH------------------K-AR

FVARGD-IQHP------------------DTYD--SGMQSNT------VHHY-ALM-TSL

S-LALD-N--------NYHITQLDISSAYLYADI---KEE-----LYI------------

----RPP-PHLG------------MNDKLIRLKKSLYGLKQSGANWYET-----IKS---

-------YLIKQ-CGMEEVRGWSC-VF------ENSQ---V--TICLFVDD-MVLFSK--

------------NLNSNKRIIDKLKMQ-YDTKIIN-----------LGESDEE-------

-----------------IQYDILGLEIKY-------------------------------

-----------------------------Q--RGKYMKLG------------------ME

N-------

>Tkm1

------------------------------------------------------------

------------------YQKEIAQLTKMN----------------TW---------NEE

LIDA-STLPK-KK--ILNSMFIFTTKR------D-NSK------------------K-CR

LVARGD-QQAA------------------DTYD--TELKANT------VDNL-ALM-TVL

A-LTLD-Y--------NLTAFQLDISSAYLYADL---KEE-----LYI------------

----RAP-PHMN------------AKNKVLRLNKSLYGLKQSGANWYEL-----IRS---

-------FLIKK-CDLIEDRMWKC-VF------RDKEPLKL--IICLFVDD-MLVVGN--

------------DVKYIKKFISKLSKR-FDTKIVN-----------DGSHRPE-------

-------------D-GVNEYDILGIELEY-------------------------------

-----------------------------K--KKEYMKFG------------------MQ

K-------

>pCal

------------------------------------------------------------

------------------MNAELEKFRSKD----------------VY--------EEV-

------PIPTGVK--PISMGWVHTEKI------D--SL----------KGVVR---K-SR

CVVHGN-RQKEK---LDYD-P-------FSVSS--PVIDLVT------IRLL-TII----

---GCE-L--------GMTIQHLDVESAYLNASI---THSN---PIYV------------

----FPP-KSVPL-----------KKNHCWLLKRSVYGLKQSGLEWYHT-----IKR---

-------VLED--IGFTQVLHNDG-LFH----IEYEEGSVI--YLGLYVDD-ILMVGS--

------------SQKVIDNFVDQLRDH-FEVKV-------------FGEIS---------

--------------------NYLGIEFR--------------------------------

-----------------------------K--TESGYILSQ-EKFK--------------

--------

>Ty2

------------------------------------------------------------

------------------YHKEISQLLKMN----------------TW--------DTNK

YYDR-NDIDP-KK--VINSMFIFNKKR------D-GTH------------------K-AR

FVARGD-IQHP------------------DTYD--SDMQSNT------VHHY-ALM-TSL

S-IALD-N--------DYYITQLDISSAYLYADI---KEE-----LYI------------

----RPP-PHLG------------LNDKLLRLRKSLYGLKQSGANWYET-----IKS---

-------YLINC-CDMQEVRGWSC-VF------KNSQ---V--TICLFVDD-MILFSK--

------------DLNANKKIITTLKKQ-YDTKIIN-----------LGERDNE-------

-----------------IQYDILGLEIKY-------------------------------

-----------------------------Q--RSKYMKLG------------------ME

K-------

>Tdh2

------------------------------------------------------------

------------------IQRELDTFKKYE----------------VY--------TVV-

------KNPKNVK--PIPTTWVHTHKI------N--DL----------KEVQY---K-SR

CVVQGF-RQIAN---EHYD-T-------SKVSS--PVIELSI------IRLL-TAI----

---AVE-Y--------EWPIHHLDISSAYLHADI---DYEK---SIFV------------

----KPP-PGSNI-----------DSGKCWQLNKSVYGMKQAGYMWYQC-----ITK---

-------VLMD--LNFEPDTAISG-MFC----KYFGENKKL--IVALYVDD-MFLTSS--

------------NITILNDFKLELAKH-FDLKY-------------FADIS---------

--------------------EFLGIEFI--------------------------------

-----------------------------Q--IAGGYRLSQ-HNFL--------------

--------

>Ty4

------------------------------------------------------------

------------------YHKELQNLKDMK----------------VF--------DVDV

KYSR-SEIPDN---LIVPTNTIFTKKR------N---GI-------------Y---K-AR

IVCRGD-TQSP------------------DTYSVITTESLNHN----HIKIF-LMM----

-------QTT------EICLWTLDINHAFLYAKL---EEE-----IYI------------

----PHP------------L----IGDVYVKLNKALYGLKQSPKEWNDH-----LRQ---

-------YLNG--IGLKDNSYTPG-LYQ-------TEDKNL--MIAVYVDD-CVIAAS--

------------NEQRLDEFINKLKSN-FELKI-------------TGTLID--------

---------------DVLDTDILGMDLVY-------------------------------

----------------------------NK--RLGTIDLTL-KSFI----------NRMD

KKYK----

>Zeco1

------------------------------------------------------------

------------------KQAEIFNWYKNN----------------VF--------EEVE

DA--------GQK--CVSTRWVCSLKE------T-QNG----------IV-----PK-AR

LVARGF-EELNI---HELQK---------DS----PTCASDS------LRLL-LAV----

---ICQ-N--------KWQVHSMDIKSAFLQGMQL--SRE-----IYV------------

----RPP-PEVG------------KENVLWKLNKCVYGLADASLYWYIK-----VK----

-------IMLS--TGSKLSKVDPA-VFY-----WLDEQCKVTGVLACHVDD--FLWAG--

------------SQNFSTNVIPILKSA-LNVGR---------------------------

--------------EEHEHFCYVGMDFVT-------------------------------

--------------------------------INGVVHVHQ-HRYI----------ENLQ

PIQ-----

>GalEa1

------------------------------------------------------------

------------------KEKEITNWKDNK----------------VY--------KEVE

DV--------GQR--ALSVRWVVTEKV------K-DGQ----------TV-----VK-AR

LVARGF-EE-ET---GNLRK---------DS----PTCSKEA------VRLA-LSV----

---AAT-C--------GWVCYSLDVKAAYLQGDQI--DRD-----VYL------------

----CPP-PEF-------------NDGSLWKLKRTVYGLCDAARHWYLR-----VRS---

-------QLLD--LGAIASSLDPV-LFS-----W-RCGRNLEGVICVYVDD--LIWAG--

------------TNSFKEQVIDRLSQI-FRMGN---------------------------

--------------SESKAFKYVGLNIVS-------------------------------

-----------------------------Y--SDGSITLDQ-NQYA----------ATLT

PIS-----

>Cico1

------------------------------------------------------------

------------------KAAELQSWKQNK----------------VY--------EEVP

NI--------GQK--CISVRWVCTVKD------H-ECG----------PK-----PK-AR

LVARGF-EERNT---QDLDK---------DS----PTCSTDA------LRVT-ITI----

---IEH-N--------NWKLNSIDVKTAFLQGELL--DRN-----IFV------------

----KPP-AAAKV-----------SKDVIWKLKKCVYGLADASKSWYKR-----VQS---

-------FLIS--IGAQITKVDQS-VFY-----W-HFKGKLQGVVALYVDD--ILWGG--

------------TAIFEESVIQLLRNQ-FTIGK---------------------------

--------------ESSEKFKYIGLEVKQ-------------------------------

--------------------------------SMNHTEICQ-DDYI----------KNVT

QIV-----

>Zeco2

------------------------------------------------------------

------------------KQAEIFNWYKNN----------------VF--------EEVE

DA--------GQK--RVSTRWVCSLKE------T-QNG----------IV-----PK-AR

LVARGF-EELNI---HELQK---------DS----PTCASDS------LRLL-LAV----

---ICQSD--------KWQVHSMDIKSAFLQGMQL--SRE-----IYV------------

----RPP-PEVG------------KENVLWKLNKCVSGL-DVNTMMQMHHCIGTTKE---

-------IMLS--TGSKMSKVDPA-VFY-----WLYEQCKVTGVLA-HVDD--FLWAG--

------------SQNFSTNVIPILKSA-LHVGR---------------------------

--------------EEHEHFCYVGMYFVT-------------------------------

--------------------------------INGVVHVHQ-HSYI----------ENLQ

PIQ-----

>Olco1

------------------------------------------------------------

------------------KVKEIKNWRDND----------------VF--------VEVQ

DE--------GQK--YISTRWVCTLKE------T-HTG----------LM-----PK-AR

LVARGF-EELQV---SELQK---------DS----PTCASES------LRLL-VAV----

---ICQ-R--------QWSLNSMDIKAAFLQGAEL--SRD-----LYI------------

----RPP-PEAD------------SKGTLWKLKKCVYGLADASLYWYNR-----VKE---

-------IMQT--LGGKVSKVDPA-VFY-----WLDEKNTVIGVLACHVDD--FIWGG--

------------TQSFSTTVIPELRSA-FQVGL---------------------------

--------------EQHGSFCYVGIDFVS-------------------------------

--------------------------------LKEKIQLHQ-ENYI----------QHLQ

PIM-----

>Athila41

------------------------------------------------------------

-----------------LDAGVIYPI-----------SDS------TW------------

-----------------VSPVHCVPKK------G---GMTVVKNEKDELIPTRTITG-HR

MCIDYR-K-------LN-------AASRKDHFP-LPF-----------IDQM-LER---L

A-NH------------PYY-CFLDGYSGFFQIPI---HPNDQEKTTF-------------

----TCP-YG------------------TFAYKRMPFGLCNAPATFQRC-----MTS---

-------IFSD--L--------------------------IEEMVEVFMDD-FSVYGP-S

F------SS---CLLNLGRVLTRCEETNLVLN--------------WEKCHF--------

---------------MVKEGIVLDHKI---------------------------------

----SEKGIEVDKGKVEVMMQLQPPKTVKD--IRSFLGHAG---FY----------RRFI

KDFA----

>Diaspora

------------------------------------------------------------

-----------------LQAGIIYPI-----------SDS------QW------------

-----------------VSPVQVVPKK------T---GLTVIKNEKEELIPTRVQNN-WR

VCIHYR-R-------LN-------QVTKKDHFP-LPF-----------IDQI-LEC---L

A-GK------------SHY-CFLDGFSGYMQITI---ALEDQEKTTF-------------

----TCL-FG------------------TFAYRRMSFGLCNAPGTFQRC-----MIS---

-------IFSD--F--------------------------LENCIEEFMDD-FTVYGS-S

F------DG---CLDSLEKVLNRRIETNLVLN--------------FEKCHF--------

---------------MVEQGIVLGHII---------------------------------

----SNKGIEVDPAKISVISQLPYPSCVE---VRSFLGHAG---FY----------RCFI

RDFS----

>Cyclops2

------------------------------------------------------------

-----------------LDARMIYPI-----------SDS------PW------------

-----------------VSPVHVVPKK------G---GNTVIRNDKDELIPTKVATG-WR

MCIEYR-R-------LN-------TATRKDHFP-LPF-----------MDQM-LER---L

S-GQ------------QYY-CFLDGYSGYNQIAV---DPADH-KTAF-------------

----TCP-FG------------------VFAYRKMSFGLCNAPTTFQRC-----VQA---

-------IFAD--L--------------------------NEKTMEVFMDD-FSVFGV-S

F------SL---CLANLKTVLERCVKTNLVLN--------------W-KCHF--------

---------------MVTEGIVLGHKV---------------------------------

----SSRGLEVDRAKVEVIEKLPPPVNVKG--IRSFLGHVG---FY----------RHFI

KEFS----

>Bagy2

------------------------------------------------------------

-----------------LEAGIIYPV-----------AHS------DW------------

-----------------VSPVHCVPKK------G---GITVVPNDKNELIPHRIVTG-YR

MVIDFR-K-------LN-------KATRKDHYP-LPF-----------IDKM-LER---L

S-N-------------THF-CFLDGYSGFSQISA---AQSDQEKTTF-------------

----TCP-FG------------------TFAYRRMAFGLCNAPATFQRC-----MMA---

-------ISSD--F--------------------------CEKIIEVFMDD-FSVYGS-S

F------DD---YLSNLDRVLQRCKDTNLVLN--------------WEKCHF--------

---------------MVNEGIVLGHKI---------------------------------

----SAKGIEVDKAKVDAIEKMPCPIDIRG--IRSFLGHAG---FY----------RRFI

KDFS----

>Calypso

------------------------------------------------------------

-----------------VARSRPHPI-----------SDS------AW------------

-----------------VSPVQVVLKK------G---GMTVIKNDKDELISTRTVTG-WR

MCIDYR-K-------LN-------NATWKDHYP-LPF-----------MDHM-LER---L

A-RQ------------SYY-CFLDGYSSY-NIAI---DIKDQEKTTF-------------

----TFP-FG------------------VFAYRCMPFGLCNALATFQRC-----MMA---

-------IFSD--M--------------------------VEKCIEVFMDD-FSVFGP-S

F------DG---CLSNLERVF-RCEESNLVLN--------------WEKCHF--------

---------------MVQEGIVLGHKI---------------------------------

----SVRGIEVDKVKIDVIEKLPPPMNVKR--MRSFLGHDG---FY-----------RLI

KDFS----

>Gloin

------------------------------------------------------------

-----------------LQCGIIRP------------SKS------PF------------

-----------------SSLVLLVKKK------D---G------------------S-WR

FCVDYR-A-------LNR------VTV-LDKFP-IPM-----------IDEL-LDE---L

H-GT------------TIF-SKLDLCLGYHQIRM---REDDIEKTAF-------------

----RTH-DG------------------HFEFLVMPFGLTNAPASFQSL-----MNE---

-------LFGP--F--------------------------LGKFVLVFFDD-ILIYSN-N

L------TN---HVKHLTLVMEVLAKHQLFAN--------------RKKCLL--------

---------------RQSQIDYLGHVI---------------------------------

----SAHGVATDPSKTEAMIHWPTPKSVKE--LFGFLG------YY----------RHFV

KGYG----

>Ifg7

------------------------------------------------------------

-----------------LEAGIIQP------------SQS------SF------------

-----------------SDPVVLVHKK------D---G------------------S-WC

MCPDYR-E-------LNK------LTI-KDKFP-IPV-----------IDEL-LDE---L

H-GS------------IYF-TKLDLRSGYHQIRM---KTEDIPKTTF-------------

----RTH-EG------------------HYEFFVMPFGLTNTPSTFQGL-----MNS---

-------IFKP--F--------------------------LRKFVLVFFDD-ILIYNK-S

W------KD---HVEHVDRVLQLLEEKKLYAK--------------RSKCFF--------

---------------VVQEVEYLGHIV---------------------------------

----S-EGVKVYPNKIKAIKEWKIPTSIKH--LRGFLGLTG---CY----------RKFA

KNYG----

>Reina

------------------------------------------------------------

-----------------LEQGVIQH------------SSS------PF------------

-----------------ASPVLLVKKK------D---G------------------E-WR

LCVDYR-R-------LNA------HTV-KNRYP-MPV-----------FDEI-VDE---L

C-GT------------KIF-TKLDHRSGYHQIRI---KEGDEFKTAF-------------

----QTH-NG------------------HYEYRVMPFGLTGAPATFQDF-----MNK---

-------ILTP--F--------------------------LRKCVVVFLDD-VLIYSR-D

M------EE---HVLQVKQVFQKLKDHQLKLK--------------LSKCRF--------

---------------AQTTLEFLGHII---------------------------------

----SAEGIATDPEKVQVIRDWPIPNNVKE--VRSFLGMAG---YY----------RRFV

AHYA----

>Gimli

------------------------------------------------------------

-----------------LESGIIQP------------SSS------SF------------

-----------------ASPVVLVKKK------D---G------------------S-WR

LCVDYR-K-------VNG------ITV-KNCFP-IPL-----------IEDL-MDE---L

G-GA------------VIF-SKFDLRAGYHQVRM---VEKDIPKTAF-------------

----KTH-SG------------------HFEYLVMPFGLTNALATFQGL-----MNS---

-------VFQK--F--------------------------LRKFVLVFFDD-ILIYSK-S

K------KE---HLQHLKLVLEVMREHKLFAK--------------RSNCAF--------

---------------ATSRVEYLGHFI---------------------------------

----DRDGLSTDPSKVLAVKEWPAPTNLKQ--LRGFLGLAN---YY----------RRFV

KNFG----

>Monkey

------------------------------------------------------------

-----------------LSGGLIRS------------SKA------PF------------

-----------------GAPVLFQKKQ------D---G------------------S-LR

LCVDYR-A-------LNK------VTV-KNKYP-IPL-----------IADL-FDQ---L

G-KA------------KYF-SKLDLRSGYWQVCI---AEGDEAKITC-------------

----VTR-YG------------------AFEFLVMPFGLTNAPATFCTL-----MNQ---

-------LFKE--Y--------------------------LDKFVVVYLDD-IVVYSQ-T

L------EE---HVKHLWMIFKVLRENTLFVK--------------REKCYF--------

---------------AQTEILFLGHRI---------------------------------

----GDGSIWMDKSKVQVVAEWRTPKKVPE--LRSFLGFVN---YY----------RRFI

AGYS----

>Tntom1

------------------------------------------------------------

---------------ENARHRYHRT------------LQV------PI------------

-----------------RVPCA-IPKE------T---W------------------Q-FT

TLCDYR-A-------LNI------ITV-KNKYP-IPL-----------MAYL-FDR---L

G-GA------------MVF-TKIDLMTGYWQVRI---AEGDEHKMTC-------------

----VTR-YG------------------SYDFLVMPFGLTNAPAIFCTL-----MNE---

-------VFQE--Y--------------------------IDEFMVVYLDD-IVVNIH-T

L------EE---HLEHLRKVLARLREH-LYAK--------------LYKCSF--------

---------------AQKQIDFIGHVI---------------------------------

----EEGRIKMDQQKIQAITNWPPPKDIHA--LRSFLVLN----FY----------WLFV

KNYS----

>Galadriel

------------------------------------------------------------

-----------------LDAGLIQP------------SKA------PY------------

-----------------GAPVLFQKKQ------D---G------------------T-MR

MCVDYR-A-------LNK------ATI-KNKYS-VPL-----------VQDL-MDR---L

S-KA------------CWF-TKLDLRAGYWQVRI---AEGDEPKTTC-------------

----VTR-YG------------------SYEFLVMPFGLTNAPATFCNL-----MNN---

-------VLFD--Y--------------------------LDDFVVVYLDD-IVIYSR-T

L------EE---HVNHLSLVLSQLRKYTLYVK--------------MEKCEF--------

---------------AQQEIKFLGHLV---------------------------------

----SKNQVRMDPKKVQAIVDWQAPRHVKD--LRSFLGLAN---YY----------RKFI

AGYS----

>Del

------------------------------------------------------------

-----------------LNKGFIRG------------STS------PW------------

-----------------GAHVLFDPKK------D---D------------------S-KR

MCIDYK---------LNS------VTV-KNKYP-LPR-----------IDDL-FDQ---L

N-GA-------------YF-SKIDLRFRYHQLRI---R-ADIPKTAF-------------

----RTR-YG------------------HYEFLVMPFGLTNVPTAFMNL-----MNR---

-------VFRE--Y--------------------------LDKFIVVFVDY-VLIYSR-T

Q------KD---HEHHLRISLQLLRNNQLYAK--------------LSKCEF--------

---------------WMEKVKFLGHVV---------------------------------

----SREGIVVDPVKVKAVMNWELPKNIFE--IRSFLGLAG---YY----------RRFI

KGFA----

>Peabody

------------------------------------------------------------

-----------------LDKKFIRP------------SVS------PW------------

-----------------GAPVLLVKKK------E---G------------------T-MR

LCVDYR-Q-------LNK------VTI-KNRYP-LPR-----------IDDL-MDQ---L

V-GA------------SVF-SKIDLRSGYHQIRV---KTEDIQKTAF-------------

----RTR-YG------------------HYEYSVMPSGVTNAPGVFMEY-----MNR---

-------IFHP--Y--------------------------LDKFVVVFIDD-ILVYSK-S

E------EE---HVEHLRVVLGVLREKKLFAK--------------LSKCEF--------

---------------WLEEVSFLGHVI---------------------------------

----SRGGVAVDPSKIEAVSKWEAPKSVAE--IRSFLGLAG---YY----------RKFI

EGFS----

>Retrosat2

------------------------------------------------------------

-----------------LQKGYIRP------------STS------PW------------

-----------------GAPVIFVEKK------D---K------------------T-KR

MCVDYR-A-------LNE------VTI-KNKYP-LPR-----------IDDL-FDQ---L

K-GA------------KVF-SKIDLRSGYHQLRI---REEDIPKTAF-------------

----TTR-YG------------------LYECTVMSFGLTNAPAFFMNL-----MNK---

-------VFME--F--------------------------LDKFVVVFIDD-ILIYSK-S

E------EE---HEQHLRLVLEKLKEHQLYAK--------------FSKCDF--------

---------------WLTEVKFLGHVI---------------------------------

----TAQGVAVDPSNVESVTKWTPPKTVSQ--IRSFLGLAG---YY----------RRFI

ENFS----

>Bagy1

------------------------------------------------------------

-----------------LNKGFIRP------------SSS------P-------------

-----------------SCPVLFVKKK------D---G------------------M-DR

MVVDYR-P-------VNL------VTI-KNKYP-LPR-----------INDL-YDQ---L

T-GS------------SFF-SKMDLRLGYHQIKI---KNGDIPKKAF-------------

----VTR-YG------------------QYEYTVMSFGLTNAPATFSRL-----MNS---

-------IFME--Y--------------------------LDKFVVVYLDD-ILIYSM-N

E------RE---HAEHLRLVLMKLREHRLYAK--------------FSKCE---------

---------------------FLGHVI---------------------------------

----SGKGIAVNPERVQAVLDWTQPESVKQ--VRSFLGLAS---YC----------RRFV

ENFS----

>Tma

------------------------------------------------------------

-----------------LGKGFIRP------------STS------P-------------

-----------------GAPVLFVKKK------D---G------------------S-FR

LCIDYR-G-------LNW------VTV-KNKYP-LPR-----------IDEL-LDQ---L

R-GA------------TCF-SKIDLTSGYHLIPI---AEADVRKTAF-------------

----RTR-YG------------------HFEFVVMPFGLTNAPAAFMRL-----MNS---

-------VFQE--V--------------------------LDEFVIIFIDD-ILVYSK-S

L------EE---HEVHLRRVMEKLREQKLFAK--------------LSKCSF--------

---------------WQREMGFLGHIV---------------------------------

----SAEGVSVDPEKIEAIRDWHTPTNATE--IRSFLGLAG---YY----------RRFV

KGFA----

>Legolas

------------------------------------------------------------

-----------------LDKGFIRP------------SSS------PW------------

-----------------GAPVLFVKKK------D---G------------------S-FR

LCIDYR-G-------LNK------VTV-KNKYP-LPR-----------IDEL-MDQ---L

G-GA------------QWF-SKIDLASGYHQIPI---EPTDVRKTAF-------------

----RTR-YD------------------HFEFVVMPFGLTNAPAAFMKM-----MNG---

-------VFRD--F--------------------------LDEFVIIFIND-ILVYSK-S

W------EA---HQEHLRAVLERLREHELFAK--------------LSKCSF--------

---------------WQRSVGFLGHVI---------------------------------

----SDQGVSVDPEKIRSIKEWPRPRNATE--IRSFLGLAG---YY----------RRFV

MSFA----

>Cereba

------------------------------------------------------------

-----------------LDKGYIRE------------SLS------PC------------

-----------------DVPIILVPKK-----------------------------G-WY

IAYVCL-L-------EAL------IIL-LFVIV-ILF-----------LGMICIDE---L

S-GY------------TIF-SKVDLRSGYNQICM---KLGDEWKTAF-------------

----KTK-FG------------------LYEWLVMPFGLTNAPSTFMRL-----MNE---

-------VLRA--F--------------------------IGRFVVLYFDD-ILIYSR-S

L------EE---HLDHLRVVFLALRDARLFGN--------------LGKCTF--------

---------------CTDRVSFLGYVV---------------------------------

----TPQGIEVDKAKIEAIESWPQPKTVTQ--VRSFLGLAG---FY----------RHFV

RDFS----

>CRM

------------------------------------------------------------

-----------------LDKGYVRE------------SLS------PC------------

-----------------AVPVILVPKK------D---G------------------T-WR

MCVDCR-A-------INN------ITI-RYRHP-IPR-----------LDDM-LDE---L

S-GA------------IVF-SKVDLRSGYHQIRM---KLGDEWKTAF-------------

----KTK-FG------------------LYEWLVMPFGLTNAPSTFMRL-----MNE---

-------VLRA--F--------------------------IGKFVVVYFDD-ILIYSK-S

M------DE---HVDHMRAVFNALRDARLFGN--------------LEKCTF--------

---------------CTDRVSFLGYVV---------------------------------

----TPQGIEVDQAKVEAIHGWPMPKTITQ--VRSFLGLAG---FY----------RRFV

KDFS----

>Beetle1

------------------------------------------------------------

-----------------LAKGFVQE------------SLS------PC------------

-----------------AVPVILVPKK------D---G------------------T-MR

MCVDCR-A-------INN------ITI-KYRYP-IPR-----------LDDL-LDE---L

H-GA------------TIF-SKIDLRSGYHQIRI---KEGDEWKTAF-------------

----KTK-SG------------------LYEWRVMPFGLTNAPSTFMRL-----MHE---

-------VLRP--F--------------------------IGSFVVVYFDD-ILVYSN-S

E------QD---HLIHLKKVFLKLREKKLYAK--------------MEKCEF--------

---------------FTSSVSFLGFII---------------------------------

----SSQGISMDESKVEAIKSWPIPKSITE--VRSFHGLAS---FY----------RRFI

RGFS----

>REM1

------------------------------------------------------------

-----------------LAKGMIQP------------STS------PY------------

-----------------GAPVIFVEKA------D---G------------------S-LR

MVLDYR-A-------LNK------ITR-KRRYP-MPN-----------ITEL-FDQ---L

A-GA------------KVF-SSLDLQQGYNQIRI---HPDDVPKTGF-------------

----IAPGMG------------------QFEYKVLCFGLTNAPATFQSV-----MNN---

-------MFGP--H--------------------------IGKFVLVYLDD-ILVFSK-N

A------EE---HKEHLRTVLEILRKNQFKAK--------------RSKCDF--------

---------------NRPELHFLGHIV---------------------------------

----SREGLKVDDRKIRVIREWEVPQDLHK--LRAFLGLAN---YF----------RRFI

QGYS----

>GRhodo

------------------------------------------------------------

-----------------YSHGLARD------------SLS------EY------------

-----------------ASPVTLAPKP------D---G------------------T-WR

FCTDYR-A-------LNS------ITQ-EAKFP-LPR-----------IDDS-LDQ---L

R-GA------------RYF-SKIDLRSGYWQVRI---EEKDIHKTAF-------------

----RTP-FG------------------HHEWLVMPFGLQGAPSTFQRM-----MNH---

-------YLRQ--Y--------------------------LGDFVICYLDD-VLIYSK-T

K------ED---HLEHIRKVLEILRKHKLYAK--------------ASKCDF--------

---------------GRTQVQYLGFIV---------------------------------

----RQGQVDKDPKKIEAIRDWPLPSTVRE--VRSFLGLAG---FY----------RKFV

EGFA----

>Sushiichi

------------------------------------------------------------

-----------------LASGIIRP------------SSS------PL------------

-----------------AAGFFFVAKE------D---G------------------G-LR

PCIDFR-K-------LNN------ITV-KNKYP-LPL-----------MSST-FEP---L

T-HA------------RVF-TKLDLRNAYHLVQI---RKGDEWKTAF-------------

----NTH-LG------------------HFEYLVMPFGLSNAPAVFQEL-----VND---

-------VLRD--M--------------------------INVFVVVYLDD-ILIFSR-T

M------EE---HHQHVRLVLQRLLENRLFIK--------------AEKCIF--------

---------------HSASVGYLGYIV---------------------------------

----EEGRVRADPAKIQAVVEWPRPTDRTQ--LRRFLGFAG---FI----------RRFI

KGFA----

>Amnichi

--------------------------------LREFIE----------------------

---------------TNLRKGFIRP------------SQS------PA------------

-----------------ASPVMFVKKK------S---G------------------D-LR

LVVDYR-A-------LNN------ITK-RNSYP-LPL-----------ISDL-LDR---L

R-GA------------KVY-TKLDLRGAYNLVRI---REGDEWKTAF-------------

----QTK-FG------------------LFESRVMNYGLCGAPATFQHF-----VND---

-------IFQD--Y--------------------------LDRFLIIYLDD-FLVFSR-S

Q------SE---HENHVKMVLQRLRDHGLYAK--------------LEKCAF--------

---------------DLQEVDFLGYRI---------------------------------

----SPLGLSMDPAKVSAVLE-RAPTNKKE--VQRFLGFAN---YY----------RKFI

PDFA----

>Amnsan

--------------------------------MKEYIS----------------------

---------------ENLQRGFIRP------------STS------PA------------

-----------------GAGFFFVEKK------D---G------------------G-LR

PCIDYR-G-------LNK------ITV-KNRYP-LPL-----------ISEL-FDQ---L

K-GA------------KIF-SKLDLRGAYNLIRI---REGDEWKTAF-------------

----NTR-DG------------------HYEYLVMPFGLCNAPAVFQEF-----VND---

-------IFRD--L--------------------------LGKSVVVYLDD-ILIFSQ-D

L------ET---HRSQVKEALSRLRENFLFAK--------------LEKCTF--------

---------------EVPKISFLGYII---------------------------------

----SSRGFEMDPAKVSAIQKWPLPQSTKA--IQRFIGFAN---YY----------RQFI

KDFS----

>Amnni

--------------------------------MESYIE----------------------

---------------ESLAAGIIRQ------------STS------PA------------

-----------------GAGFFFVGKK------D---G------------------G-LR

PCIDYR-G-------LNK------ITI-RNRYP-LPL-----------MSTA-FEI---L

Q-EA------------SIF-TKLDLRNAYHLVRI---KQGDEWKTAF-------------

----NTP-TG------------------HYEYLVMPFGLTNAPAVFQAL-----IND---

-------VLRD--M--------------------------LNKFVFVYLDD-ILIFSS-S

L------QE---HIFHVRKVLQRLLNNHLYVK--------------PEKCQF--------

---------------HVTQVKFLGFII---------------------------------

----KPGQIQMDPQKIQAMVDWPSPSSVKE--VQRFLGFAN---FY----------RKFI

LNFS----

>Dane1

------------------------------------------------------------

-----------------LRKGFIRP------------SSS------PT------------

-----------------ASPVLFVKKP------G---G------------------G-LC

FCVDYQ-R-------LNW------ILV-KDQYP-LPL-----------VKET-LNN---L

K-GM------------RYF-TKIDIISAFNNIRI---KKGQEYLTAF-------------

----RTC-LG------------------LYESLVMPFGLTGAPATFQHY-----IND---

-------TLRD--Y--------------------------LDIFCTAYLDN-ILIYSQ-T

R------SE---HIQHVRKVLQKLREAGLFAK--------------LVKYEF--------

---------------TVHETKFLGLIV---------------------------------

----ARDRIKIDPEKVQTIAAWATPTCITD--IQAFIRFAN---FY----------RRFI

KDFS----

>Maggy

------------------------------------------------------------

-----------------LKKGFIRP------------SSS------SV------------

-----------------ASPVLFVKKQ------G---G------------------G-LR

FCVDYR-A-------LNN------ITV-KDRYP-LPL-----------VRET-LNN---L

A-GM------------KFF-SKIDIVSAFNNIRI---KKGEEYLTAF-------------

----RTR-FG------------------LYESLVMPFGLTGAPATFQRY-----IND---

-------SLRE--Y--------------------------LDVFCTAYLDD-ILIYSR-T

R------TE---HEEHLKLVLEALRKAGLYAN--------------AAKCEF--------

---------------FVTETKFLGLLV---------------------------------

----GVEGVKMDPEKITAVLDWQTPKKLTD--VQAFLGFGN---FY----------RRFI

RDFA----

>marY1

------------------------------------------------------------

-----------------LASGRIRP------------SKS------PM------------

-----------------ASPFFFVKKK------D---G------------------S-LR

PVQDYR-R-------LNN------ITV-KNRYP-LPL-----------ISEL-VNQ---L

H-GA------------RYF-TKLDVRWGYNNVRI---KEGDEWKAAF-------------

----RTN-RG------------------LFELLVMFFGLTNSPATFQTM-----MND---

-------IFHD--LI-------------------------LEGVVCIYLDD-ILIFTR-M

V------EE---HRRITRLVLERLRRYKLYLH--------------QDKCEF--------

---------------ERTKIEYLGLII---------------------------------

----SEGQVEMDPIKVNRVTAWPTPTNKKE--VQSFLGFIN---FY----------RRFI

KDFS----

>Cgret

------------------------------------------------------------

-----------------LKKGYIRE------------STS------PA------------

-----------------GSPILFVPKK------N---G------------------K-LR

LCVDYR-M-------LNE------MTI-KNRYP-LPL-----------IDEL-QRL---L

H-GA------------NWF-TALDLKGAYNLIRM---KEGEEWKTAF-------------

----RTR-KG------------------HFEYLVMPFGLTNAPATFQNM-----INQ---

-------VLRK--F--------------------------VDIFVVVYLDD-ILIFSP-T

L------KQ---HKEHVHLVLQALQNAKLLVE--------------PEKSKF--------

---------------HAQEVEYLGFTI---------------------------------

----TPGHIHMSKDKVRSIQEWPTPTNLKE--VQSFLGLVN---FY----------RKFI

KYYG----

>Cft1

------------------------------------------------------------

-----------------LAKGWIRR------------STS------SA------------

-----------------GTPCMFVPKA------N---G------------------K-LR

LVQDYR-K-------LNE------ITI-KNRYP-LPN-----------IEEA-QDR---L

T-GS------------DWY-TKIDLRDAFYAIRM---AEGEEWKTAF-------------

----RTR-YG------------------LYEFLVMPMGLTNAPASCQDL-----VNE---

-------TLRD--L--------------------------LDVCVVAYMDD-ILVYTKGS

L------QE---HTKQVQDVFERLTKSGFKTA--------------PEKCEF--------

---------------HKKEVKFLGFII---------------------------------

----STTGITIDPAKTQSIREWPEPKTVKD--VQSFLGLAN---YN----------RKFI

KDYS----

>Pyret

------------------------------------------------------------

-----------------FKKDYIRP------------SIS------PA------------

-----------------GYPILFVPKK------N---G------------------K-LR

LCVDYR-Q-------LND------ITI-KNCYP-LPL-----------IREF-RNM---L

Y-QT------------QWF-TTLNLKGAYNLIRI---KEGEKWKTAF-------------

----RTK-RG------------------YYEYLIMPFGLTNAPATFQTI-----INH---

-------VLRE--C--------------------------LNIFVVIYLDD-ILVFSK-T

L------EK---HKQHVHTILQKLQNAKLLVE--------------PEKYLF--------

---------------HSKQINFLGYII---------------------------------

----APGEIRMEKSKIQAVKEWPQPQNWTEQTQQAFEQLRD--AIT----------RE--

--------

>Skippy

------------------------------------------------------------

-----------------IRKGYIRP------------SKS------SA------------

-----------------GFPVMFVPKP------N---SN-----------------K-LR

LVVDYR-Q-------LNE------ITE-KDRTS-LPL-----------ITEL-KDR---L

F-GK------------KWF-TALDLKSAYNLIRI---KEADEWKTAF-------------

----RTK-YG------------------LFEYLVMPFGLTNAPAVFQRM-----ITN---

-------VLRE--Y--------------------------LDIFVVCYLDD-ILIFSD-T

E------EE---HTEHVHKVLKALQDANMLVE--------------PTKSHF--------

---------------HQSQVTYLGHEI---------------------------------

----SHNEIRMDRRKIAAVAEWKVPTSVKE--TQSFLGFAN---YY----------RRFI

KDFA----

>Real

------------------------------------------------------------

-----------------LEKGFIRV------------SSS------PA------------

-----------------AAPVLFAKKP------G---G------------------G-LR

LCIDYR-A-------LNA------ITK-KDRYP-LPL-----------IRET-LNN---L

S-KA------------KWF-TKLDVIAAFHKIRV---AEGDEWKTAF-------------

----RTR-FG------------------LFEWLVTPFGMANSPSTFQRY-----INW---

-------TLRE--F--------------------------LDDFCSAYLDD-VLIYTDGS

L------KQ---HQEHVRKVLRKLQDAGLQVD--------------IKKCEF--------

---------------EVKSTKYLGFIIK--------------------------------

----AGKGISMDPAKVAAIREWEAPQTVKG--VRSFLGFAN---FY----------RKFI

KNFS----

>MGLR3

------------------------------------------------------------

-----------------LQKGFIEP------------GST------PW------------

-----------------AAPILFARKG------D---G------------------G-LR

FCVDYR-K-------LNA------LTK-KDVCP-LPL-----------IEET-LAR---I

S-KA------------RFF-TKIDIRQAFHRIRM---NPEHRDYTTF-------------

----RTR-YG------------------TFRYNVLPFGLTNGPATFQKF-----INE---

-------ILME--Y--------------------------LDDFCSAYMDD-ILIWSE-T

E------EE---HQTHVRQVLERLKKAGLQAD--------------IKKCEF--------

---------------HVTETRFLGFII---------------------------------

----GTKGVAVDPDKVAAVKQWAPPTTVKG--VQSFLGFCN---FY----------RKFV

PEYS----

>Grasshopper

------------------------------------------------------------

-----------------MDKGWIRA------------SSS------SA------------

-----------------AAPVLMVRKA------S---G------------------G-WR

LCVDYR-A-------LNS------ITM-QDRYP-LPL-----------IKET-IRS---L

T-GA------------RWF-TKVDVRAAFHKLRI---AEGDEHLTAF-------------

----RTR-FG------------------LFEWLVCPFGLAGAPATFQRY-----VNG---

-------VLGD--T--------------------------LGDYASAYLDD-ILIYSSGS

K------SD---HWSKVTRVLDKLAAAGLNLD--------------LDKSAF--------

---------------AVKEVKYLGFIVK--------------------------------

----AGEGVQADPEKIKAIRDWEAPTRLRG--LRGFLGFAN---FY----------RDFI

DGYA----

>Pyggy

------------------------------------------------------------

-----------------LDKGFIRV------------SSL------LA------------

-----------------FLLVLFAKKL------E---G------------------G-LR

LCIDYW-A-------LNA------ITRGKNRYL-LPL-----------IRET-LNN---I

S-KA------------KWF-TKLDVIAAFYKIRV---AEGDEWKTAF-------------

----RTR-FS------------------LYEWLVTPFGMANSLSTFQRY-----INW---

-------TLRE--Y--------------------------LDEFCSAYLDD-VLIYTDGS

L------EQ---HQDHVRKVLRKLQESGLNVD--------------IKKCEF--------

---------------GVKSTKYLGLIID--------------------------------

----AEKGIRMDPEKVKAIMEWE-PLRLRA--YVHFWDSQT---ST----------GDLS

RDFS----

>Tse3

------------------------------------------------------------

-----------------IASGNVIP------------SES------PY------------

-----------------AAPVIFVQKK------D---G------------------T-KR

LCVDYR-G-------LND------ITI-KSKFP-LPL-----------IEDV-LDQ---L

S-GA------------TIF-SKLDLISGYHQVAI---ADEDQYKTAF-------------

----TTH-RG------------------QYSWRVMPFGLTNAPATFQRL-----MNY---

-------VLRD--Y--------------------------INKICVVYLDD-ILIYSK-N

E------KE---HSEHVSTIINVLRKHQLYAK--------------KSKCEF--------

---------------YVPKIQFLGHEL---------------------------------

----SAKGITPDKEKILAIKDWPTPKTYKD--AQSFIGLAG---YY----------RRFI

KDFS----

>Ty31

------------------------------------------------------------

-----------------LDNKFIVP------------SKS------PC------------

-----------------SSPVVLVPKK------D---G------------------T-FR

LCVDYR-T-------LNK------ATI-SDPFP-LPR-----------IDNL-LSR---I

G-NA------------QIF-TTLDLHSGYHQIPM---EPKDRYKTAF-------------

----VTP-SG------------------KYEYTVMPFGLVNAPSTFARY-----MAD---

-------TFRD--L----------------------------RFVNVYLDD-ILIFSE-S

P------EE---HWKHLDTVLERLKNENLIVK--------------KKKCKF--------

---------------ASEETEFLGYSI---------------------------------

----GIQKIAPLQHKCAAIRDFPTPKTVKQ--AQRFLGMIN---YY----------RRFI

PNCS----

>Skipper

------------------------------------------------------------

-----------------IDLGIIKR------------SES------NY------------

-----------------SSPIMLLKKR------D----------------------S-WR

VVHDYR-Q-------LNK------VTV-RDDHP-FTP-----------VDSL-LNQ---C

K-DS------------KLF-SKFDMIMGYFQVLI---NPEHAKYTAF-------------

----ITH-IG------------------KFEYTRMPQGLVNSPSTFARL-----MVE---

-------IFGK--I----------------------------KSLLQYFDD-LLVHSK--

-------LDYMVHFIEIIRMLLYCRKYLLFIS--------------REKSEM--------

---------------LKTEVDFLGFHI---------------------------------

----HKDGISPRAAKVRAISELPEPRNAKE--AEAALGLFG---FF----------RRHI

ENYA----

>TF2

------------------------------------------------------------

-----------------LKSGIIRE------------SKA------IN------------

-----------------ACPVMFVPKK------E---G------------------T-LR

MVVDYK-P-------LNK------YVK-PNIYP-LPL-----------IEQL-LAK---I

Q-GS------------TIF-TKLDLKSAYHLIRV---RKGDEHKLAF-------------

----RCP-RG------------------VFEYLVMPYGISTAPAHFQYF-----INT---

-------ILGE--A--------------------------KESHVVCYMDD-ILIHSK-S

E------SE---HVKHVKDVLQKLKNANLIIN--------------QAKCEF--------

---------------HQSQVKFIGYHI---------------------------------

----SEKGFTPCQENIDKVLQWKQPKNRKE--LRQFLGSVN---YL----------RKFI

PKTA----

>TF1

------------------------------------------------------------

-----------------LKSGIIRE------------SKA------IN------------

-----------------ACPVMFVPKK------E---G------------------T-LR

MVVDYK-P-------LNK------YVK-PNIYP-LPL-----------IEQL-LAK---I

Q-GS------------TIF-TKLDLKSAYHLIRV---RKGDEHKLAF-------------

----RCP-RG------------------VFEYLVMPYGISTAPAHFQYF-----INT---

-------ILGE--A--------------------------KESHVVCYMDD-ILIHSK-S

E------SE---HVKHVKDVLQKLKNANLIIN--------------QAKCEF--------

---------------HQSQVKFIGYHI---------------------------------

----SEKGFTPCQENIDKVLQWKQPKNRKE--LRQFLGSVN---YL----------RKFI

PKTS----

>Tor4a

------------------------------------LD----------------------

---------------QFLENKLIEP------------CSS------PW------------

-----------------NSPSLLVRKK------D---G------------------R-FR

LVIDYR-R-------LND------ATL-QMHHP-LPN-----------LEDS-ISY---L

E-KS------------RVY-SMCDMIKGFHQIDL---DEDSKQKTAF-------------

----SNE-FG------------------QFQYTRMPMGCKNAPSFFMRI-----MDK---

-------ALLG--V--------------------------KKTEIIAYMDD-LLCHSQ-T

E------VE---HIKILEKLFQILAVNNLRIN--------------SKKAAF--------

---------------FTDSVNFCGYDI---------------------------------

----SNGKICPSQDKIEAIQKLKIPRTKDA--AQSLFGALN---YH----------RRFI

RNFA----

>Tor2

--------------------------------VKAQID----------------------

---------------NMAKDGIIEPC----------PDGK------GF------------

-----------------HSPLVIVRKK------T---G------------------G-LR

ICSDFK-SS------LNQCLDET-----TDIWP-LPQ-----------MDHL-FAN---I

EHGH------------RIF-TSLDVSKAYWNLLI---DPRDRHKTNF-------------

----TFD-NK------------------CWMYVRLPFGLKFSGDAFCRS-----ISS---

-------MLDR--VS-------------------------LKSNFCNYVDD-VLAYSS-D

C------ET---HVKVLQQIFSACRHFGARLG--------------AKKCTF--------

---------------GQASTAFMGRII---------------------------------

----SAEGISIPPENMETILALKPPTTRKQ--VQSLIGNFC---WL----------KSWV

SANL----

>Tor1

------------------------------------------------------------

----------------MEDAGIIEKA-----------SGS------SF------------

-----------------NAPLQLVRKS------S---G------------------G-YR

ICVDMR-S-------LN-------NRLAESKWP-LPS-----------LAET-LES---L

A-GT------------AFF-SCVDIRQAFFHMAL---TDESKHLTAF-------------

----SAL-NC------------------QYQFRRLPMGLKISPSVYQMA-----MKE---

-------TLGN--D--------------------------LGNKAVVYLDD-VLVTGR-T

E------DE---HLEALDVVLDRLRKAGFLLN--------------PDKCIL--------

---------------GVKKTTFLGHEV---------------------------------

----TTEGYYPKTDNLAAIREFPKPTNKKA--LRRFIGMTA---FY----------STLV

PKLQ----

>Cer1

------------------------------------------------------------

-----------------LNQKVIRE------------SKS------PW------------

-----------------SSPVVLVKKK------D---G------------------S-IR

MCIDYR-K-------VNK------VVK-NNAHP-LPN-----------IEAT-LQS---L

A-GK------------KLY-TVFDMIAGFWQIPL---DEKSKEITAF-------------

----AIG-SE------------------LFEWNVLPFGLVISPALFQGT-----MEE---

-------IIGD--L--------------------------LGVCAFVYVDD-LLIASK-D

M------EQ---HLQDVKEALTRIRKSGMKLR--------------ASKCHI--------

---------------AKKEVEYLGHKV---------------------------------

----TLDGVETQEVKTDKMKQFSRPTNVKE--LQSFLGLVG---YY----------RKFI

LNFA----

>Cer2

------------------------------------------------------------

-----------------IQSERIVE------------SNT------PW------------

-----------------TSPIVLVKKK------N---G------------------S-LR

VCLDFR-K-------LNE------VTI-PDNYP-LPR-----------IDAI-IEK---V

G-GS------------RYF-SVLDMANGYLQLRL---DAESSYKCGF-------------

----ITE-NK------------------VYAYTHLPFGLKSAASYFQRA-----LRQ---

-------VLDG--L----------------------------EDVMVYIDD-VLIYSK-T

F------ED---HIRTLEMVLERFRKFNLKAS--------------PNKCEF--------

---------------FKESIVFLGHEI---------------------------------

----SRDNYSPNRVNVETIRSMPTPTNVNE--VRRFVGMSG---FF----------RKFI

PNFS----

>Cer3

------------------------------------------------------------

-----------------IQSGRIVE------------SNT------PW------------

-----------------TSPIVLIKKK------N---G------------------S-LR

VCLDFR-K-------LNE------VTV-PDNYP-LPR-----------IDSI-IEQ---I

G-GS------------KYF-TSLDMANGYLQLRL---DAESSDKCGF-------------

----ITE-NK------------------VYAYTHLPFGLRSAASYFQRD-----LKQ---

-------VLG------------------------------LEKEVTVYIDD-VLIFSK-T

F------EE---HLNSLRKVLERFRKFNLKVS--------------PNKCEF--------

---------------FKQSITFLGHEI---------------------------------

----NADNYKPNRINVDAIVNLPTPRNVGD--VRRFIGMSG---FF----------RKFL

PNFS----

>Cigr1

------------------------------------------------------------

-----------------LEEEIIEP------------SNS------PW------------

-----------------RAQVLVAREG------S---K------------------K-PR

MVIDYS-QT------VN-------VFTELDAYP-LPS-----------IESI-VNK---V

T-------QD------KVY-SSLDLRSAYHQIPL---KDSDRPFTAF-------------

----EAE-GQ------------------LYQYKRLPFGVTNGVSAFQRI-----INR---

-------FIQD--N--------------------------HLNKVYAYLDD-ITVTGE-T

L------EE---HDRNLKKLLVAAKRHNLTIN--------------EEKSKL--------

---------------RMTSIDFLGYRI---------------------------------

----SYGDARPDPERLRPLLNLPPPDTPRE--LKRIQGMFA---YY----------AKWI

SRFS----

>CsRN1

------------------------------------------------------------

-----------------LELDIIRT------------SSS------HW------------

-----------------SSPLHMVPKK------S-K-G------------------D-WR

PCGDYR-S-------LNN------ATI-PDRYP-IPH-----------IHDF-AST---S

C-HT------------NIF-SKLDLVRAYYHIPI---APDDIPKTAI-------------

----ATP-FG------------------LFEFTRMPFDLRNAAQTFQRF-----MDE---

-------VLRG--L----------------------------PFVYAYLDD-VLIAST-S

P------ME---HAAHLRAVFERLSTYNIRLN--------------IDKCLF--------

---------------GATNLDFLGHHI---------------------------------

----DSTGISPLPDRILALESFPIPTTLTQ--LRRFIGIIN---YY----------RRFI

PHCA----

>Kabuki

------------------------------------------------------------

-----------------QELGICRP------------SKI------AW------------

-----------------ASPLHIVPKK------N---G------------------D-LR

PCGDYR-Q-------LNA------ITK-PDRYP-IPR-----------RHDF-TYI---L

D-DK------------KIF-SKLDINRAFHCIDV---APEDIEKTAI-------------

----ITP-FG------------------LFEFPKMTFGLRNAPQTFQRF-----MNNT--

-------VLHG--L----------------------------EFLFSYLDD-VIIASE-S

P------TQ---HNEHLRIVFERFNSYGITIN--------------LDKCCF--------

---------------GQPKVEFLGHEV---------------------------------

----SVNGITPLKNKVEAIINFPKPETVSD--LRRFLGMVN---FY----------RPHL

PNSA----

>Boudicca

------------------------------------------------------------

-----------------RSRELIRS------------SNS------PW------------

-----------------SSPLHMVPKK------D-S-N------------------D-WR

PTDVYR-R-------LNA------KTI-PDRYP-LPH-----------IHDL-TAT---L

K-GT------------TVS-SKIDLVKAYDQIPM---ATNEIPKTAI-------------

----ITL-FG------------------LYEFLQMPFGLRNAAQTFQRF-----IDD---

-------VFRG--L----------------------------NFVHAYVDD-CLIASP-D

R------ES---HLKHLDIVFDRLQRHGITVN--------------IQKCQI--------

---------------GTNSLDFLGHTI---------------------------------

----DAQGIRPLRSKMVAILDYPEPTTIKQ--LRTFNGLVS---FY----------RRFI

PKCA----

>Osvaldo

------------------------------------------------------------

-----------------LVKGCIEP------------SKS------PH------------

-----------------TRTYSNGQGR-----KN---G------------------K-WR

LCVDFR-Q-------LNS------RSI-KDAYP-LPR-----------VHHI-LDQ---L

R-EA------------RYI-TSLDLKDGYWQIPM---EKSSRPLTAF-------------

----TVPGKG------------------LFQWKVMPFGLHSAPATFQRA-----LDQ---

-------VIGP--D--------------------------MMPHAFAYLDD-IIVIGR-T

R------QE---HMDNLREVFRRLRAANLRIN--------------IDKCDF--------

---------------FKKELKYLGHKV---------------------------------

----TENGIRTDPEKVAAIAQLKPPTNVKE--LRQYVGVAS---WY----------RRFV

PDFA----

>Woot

------------------------------------------------------------

-----------------LDLGVIKR------------EAS------PY------------

-----------------ASPMTVGKKK------D---G------------------T-VR

ICLDAR-M-------INS------KMI-ADCES-PPA-----------ADEL-LRR---F

H-EI------------RYM-STIDLRSSYWQIPL---SPESRQYTAF-------------

----LYN-GR------------------SYTYQVLPFGLKTAVGSFSRA-----MDV---

-------VLGT--E--------------------------VREFVVNYIDD-LLVASE-T

L------NE---HLEHLRQVFEKLKQARMTIN--------------LEKSNF--------

---------------IQKEVKFLGHIL---------------------------------

----TINGIKADPEKISAIRNFPVPQKTKH--VRAFLGLCN---FY----------RKFC

ARYA----

>Ulysses

------------------------------------------------------------

-----------------LKLGIIEE------------SDS------PW------------

-----------------SNRTTVVMRP----------G------------------K-NR

FCLDAR-K-------LNS------VTV-KDAYP-LPC-----------IEGI-LSR---S

T-----------RLILSLA-STLSSRSG--NRDG---GEEQGVYGVY-------------

----CTR-RP------------------LYQFRHMPFGLCNAAQHFEAH-----MDK---

-------VIPA--N--------------------------LRSNVFVYLDD-LLIISA-D

F------PT---HLKYLELVAECLRNANLTIG--------------MAKSKF--------

---------------LFRNLNYLGFIQL--------------------------------

----RRRTWRMDPGRVEAIRNIPNPRTVKE--LRSFLGTAG---WY----------RRFI

KNFA----

>Circe

------------------------------------------------------------

----------------------VKSI----------------------------------

----------------------------------------------------------VR

LRWMLK-RH------LHR----------LGHLQ-LPCISNQARCDFVSMQEN-MQS---L

Q-TA---KSS--NLTSKMFRSRIPCQDCFHRS----------------------------

--------KA------------------PLSVQKNPFGLS--------------------

-------IHKP--CA-------------------------VCTSLYPINNR-MYSTSK--

---ICWFCQ---TISKTIFCICPKRKAGLTIN--------------VQKSQF--------

---------------CLKTVDYLGYLV---------------------------------

----GEGTLQVNPNKIAAVGDFPVPKTKQ---LRRFLGMTG---WY----------QRLI

SNYS----

>Gmr1

------------------------------------------------------------

-----------------LKLGVIEE------------SRS------AW------------

-----------------ASPIVLVPKP------D---G------------------T-HR

FCNDFR-R-------LNE------VSD-FDSYP-MPR-----------VDEL-IER---L

G-PA------------RYL-STLDLTKGYWQVPL---APSSREKTAF-------------

----ATP-GG------------------LFQYTVLPFGVHGAPATFQRM-----MDQ---

-------VLRP--H---------------------------SSYAAAYIDD-IIIHSA-S

W------DE---HVKHVRAVLNGLRAAGLTAN--------------PAKCRL--------

---------------GREETAYLGYRV---------------------------------

----GRGNVRPQEDKVAAIREWPQPQTKKQ--VRSFLGLVG---YY----------QRFI

PGYA----

>rGmr1

------------------------------------------------------------

-----------------LKLGVIEP------------SRS------PW------------

-----------------SSPIVMVPKP------D---G------------------T-LR

FCNDFR-K-------LNE------ASS-FDGYP-MPR-----------VDEL-LDR---L

G-SA------------RFI-STLDLTKGYWQVPL---APGAKEKTAF-------------

----TTP-SG------------------HWHYRVLPFGLHGAPATFQRM-----MDI---

-------LLRP--H---------------------------QSYAAAYLDD-VVVHSM-C

W------EE---HLTRLRRVLLELRRAGLTAN--------------PKKCHL--------

---------------GLAEAKYLGYHI---------------------------------

----GRGLIQPQQAKVEALQKTPRPTNKSQ--VRAFLGLAG---YY----------RCFI

PNFS----

>RetroSor1

------------------------------------------------------------

-----------------LDAKVIREV-----------IYP------EW------------

-----------------LANVVLVPKK------N---G------------------K-MR

MCIDFT-D-------LN-------KACVKDSFP-LPR-----------IDTS-VDK---A

A-GC------------QRF-SLLDCFSGYHQIWL---KKEDEGKASF-------------

----TTP-FG------------------TYCYTRMPEGLKNAGATFSRM-----MGK---

-------VLGS--Q--------------------------LQRNIIAYVDD-VVVMSK-R

K------ED---HIKDLQETFVNLRSAGLKLN--------------PEKCVF--------

---------------GVSKGKMLGYII---------------------------------

----SSEGIRANPDKTKAIMSMAEPSNKKE--VQRLTGRIA---AL----------NRFI

SRSA----

>Cinful1

------------------------------------------------------------

-----------------LSAGVIREV-----------KYP------EW------------

-----------------LANTVMVKKA------N---G------------------K-WR

MCIDFT-D-------LN-------KACPKDEFP-LPR-----------IDSL-VDA---T

A-SS------------ELM-SLLDCYSGYHQIWM---KREDEPKTSF-------------

----ITP-SG------------------TYCYLRMPEGLKNAGGSFSRM-----TAK---

-------VLQS--Q--------------------------IGRNVLTYVDD-IIVKST-K

Q------EN---HIADLQETFASFRQAGLKLN--------------PEKCVF--------

---------------GVKKGKFLGCLV---------------------------------

----STKGIEANPSKIEAILRMEPPTTKKG--AQRLTGRLA---SL----------NRFI

SRSA----

>B1147A04.5

------------------------------------------------------------

------------------------------------------------------------

------------------------------------------------------------

-LVSFL-R-------ANAD---VFAWRPADMPG-VPRE---------VIEHR-LAV---R

S-GARPVRQKVRRQAPERQ-AFIR--SGYHQIRM---AREDEEKTAF-------------

----ITP-IG------------------TYCYTTMPFGLKNAGPTFQRT-----TRI---

-------SLGS--Q--------------------------IGRNVEAYVDD-LVVKTR-N

Q------ET---LLSDLAETFESLRSARIKLN--------------PDKCVF--------

---------------GVPAGKLLGFLV---------------------------------

----SARGIEANPEKIRAIERMRPPSKLRD--MQCVTGCMA---AL----------SRFI

SRLG----

>Ogre

------------------------------------------------------------

-----------------WDAGFLAVT-----------SYP------PW------------

-----------------MANIVPVPKK------D---G------------------K-VR

MCVDYR-D-------LN-------RASPKDDFP-LPH-----------IDVL-VDN---T

A-QS------------SVF-SFMDGFSGYNQIKM---APEDMEKTTF-------------

----ITP-WG------------------TFCYKVMPFGLKNAGATYQRA-----MTT---

-------LFHD--M--------------------------MHKEIEVYVDD-MIAKSQ-T

E------EE---HLVNLQKLFDRLRKFKLRLN--------------PNKCTF--------

---------------GVRSGKLLGFIV---------------------------------

----SEKGIEVDPAKVKAIQEMPEPKTEKQ--VRGFLGRLN---YI----------ARFI

SHLT----

>Grande14

------------------------------------------------------------

-----------------VAAGFIREV-----------LHP------EW------------

-----------------LPTLFLYSKR------I---K------------------W-IG

ACASTI-L-------IST------NTVRRDPFG-LPR-----------IDQV-VDS---T

A-GC------------SML-SFLDCYSWYHQISL---AKEDEEKTAF-------------

----ITP-FG------------------AFCYTSMSFGLKNGRATYQRA-----IQT---

-------CLAN--H--------------------------WGKRVEAYVDD-VVIKIE-N

S------EN---FIEDLQLVFNSLRRYRWKLN--------------PEKCVF--------

---------------GVPAGKLLGFIV---------------------------------

----SHRGIEANPDKIEAIMKMEAPRSQKK--VQRLTGCMA---AL----------SRFI

SRLG----

>Tat41

------------------------------------------------------------

-----------------LGAGSIVEV-----------KYP------EW------------

-----------------LANPVVVKKK------N---G------------------K-LR

VCIDFT-D-------LN-------KACPKDSFP-LPH-----------IDRM-VEA---T

T-GN------------ELL-SFMDAFSGYNQIPM---HKDDQEKTSF-------------

----ITD-RG------------------TYCYKVMPFGLKNAGARYQRL-----VNQ---

-------MFAP--Q--------------------------LGKTMEVYIDD-MLVKSK-K

S------AD---HIEHLTACFETLNKYNMKLN--------------PAKCSF--------

---------------GVTSGEFLGYIV---------------------------------

----TKRGIEANPKQIQAILDLKSPRNKKE--VQRLTGRIA---AL----------NIFI

ARST----

>Tft2

------------------------------------------------------------

-----------------LKIGSIREV-----------QYP------DW------------

-----------------LANTVVVKKK------N---G------------------K-RI

VCIDFT-D-------LN-------KACPKDSFP-LPH-----------IDNL-VES---T

A-GN------------ELL-SFMDALSGYNQIMM---NPEDQEKTSF-------------

----ITD-RG------------------IYCYKVMPFGLKNAGATYQRL-----VNK---

-------MFNE--H--------------------------LGKTMEVYIDY-MLVKSL-K

K------ED---HVKHLEECFAILNQYQMKLN--------------PAKCTF--------

---------------GVPSGEFLSYIV---------------------------------

----RKRGIEANPNQINAFLNMSSPKNFKE--VQRLTGRIA---AL----------NRFI

SRST----

>RIRE2

------------------------------------------------------------

-----------------LAAGFIKEV-----------LHP------DW------------

-----------------LANPVLVRKK------T---G------------------Q-WR

MCVDYT-D-------LN-------KSCPKDPFG-LPR-----------IDQV-VDS---T

A-GC------------ELL-SFLDCYSGYHQIRL---KESDCLKTSF-------------

----ITP-FG------------------AYCYVTMPFGLKNAGATYQRM-----IQR---

-------CFST--Q--------------------------IGRNVEAYVDD-VVVKTK-Q

K------DD---LISDLEETFASIRAFRMKLN--------------PEKCTF--------

---------------GVPSGKLLGFMV---------------------------------

----SHRGIQANPEKVTAILNMKPPSTQKD--VQKLTGCMA---AL----------SRFV

SRLG----

>Mag

------------------------------------------------------------

-----------------LAAGVIKPV-----------DHS------DW------------

-----------------ATPLVVVRKA------D---G------------------G-LR

ICADYK-VT------LN-------KVLAIDRFP-VPK-----------MEDL-FSN---L

S-GN------------KFF-TKLDLSQAYNQIVL---SERSSEYTVI-------------

----NTH-RG------------------LFKYSRLVYGLASSPGIFQKL-----MVN---

-------MFKN--V----------------------------PNVVVFYDD-ILIRNQ-D

L------DS---HLKSIKEVLDILERYGLKIK--------------RSKCEF--------

---------------MVTEVRYLGFII---------------------------------

----DQNGVRVDPEKVKSIATMPHPNNVTE--LKSFIGMVN---FY----------SKFI

QDLA----

>Gulliver

------------------------------------LE----------------------

---------------RLY-MNVISPV-----------SYS-------V------------

-----------------ATPLVFIKKA------D---G------------------K-LR

VCGDFS-TG------LN-------KALQDHLYP-LPI-----------PEDL-FTI---L

N-GC------------VVF-SKIDFSDAYFQVEV---AEESRLLLTV-------------

----NTH-RG------------------LFQYNRLPFGVKCAPAIFQQI-----MDT---

-------MLAD--L----------------------------PFSMAYMDD-IIVTSK-N

E------KE---HYAHSQKVFRRLSEYGFCMK--------------LEKCSF--------

---------------FMSSIKYLGIII---------------------------------

----DLNGGRPNPEKVNAVVHMSIPSNVQK---PSILGMIN---YY----------NSLC

LTCT----

>DRM

------------------------------------LD----------------------

---------------RLEKAGIITPV-----------KYS------EW------------

-----------------AAPIVPVVKK------D---K------------------T-LR

LCGDYK-LT------VN-------QAVTTEIYP-LPR-----------IEEL-MAT---L

S-GG------------TIF-SKIDLASAYQQVLL---EDESKELLTI-------------

----NTH-RG------------------LFVYNRLPFGVSSAPSIFQRI-----MEN---

-------LMRD--L-----------------------------DVIVYLDD-LLVTGK-T

E------QE---HLQRLQAVLKRLQENGLRVK--------------KSKCEF--------

---------------GKSQIEYLGFVL---------------------------------

----NSQGLHPSPDKVDAVKNAPVPTCVKE--LKAFLGLVN---YY----------GRFL

PNQS----

>CFG1

------------------------------------LD----------------------

---------------RLVAEGTISPV-----------EFS------EW------------

-----------------ATPIVPIVKS------D---K------------------S-IR

ICGDYK-VT------VN-------KVSKLDNYP-IPK-----------TEDL-YAT---L

G-GG------------TDY-SKLDLSQAYQQLEL---DDSSKPYTTI-------------

----NTH-KG------------------LFVYNRLPYGVASAPGIFQRT-----MEN---

-------LLQG--I----------------------------PQVIVRVDD-ILVTGK-N

R------RQ---HLENLEAVLNRLEKAGVKLK--------------RSKCYF--------

---------------LRKEVEYQGHRI---------------------------------

----NSEGMQPIEGKVMAIKDAPAPTNVKE--LQAFLGMLN---YY----------SCYL

PRLS----

>Hydra21

------------------------------------LD----------------------

---------------RLISSGIYRPV-----------SHS------RW------------

-----------------AAPIVPVLKK------D---G------------------T-IR

LCGDYK-QT------VN-------QAAMCDSYP-LPR-----------TEDL-FAT---L

A-GG------------QKF-TKLDLAFAYQQLLL---DKSSCELLTV-------------

----NTH-RG------------------LFEPTRLQFGVHSASGIFQRE-----IEK---

-------LIGN--L----------------------------PFTKVRVDD-ILVSGR-S

D------EE---HLKNVETVLLILEKAGLKLK--------------ENKCVF--------

---------------MSPEVEYLGFKL---------------------------------

----TKDGVVPLEDKLNAIRNAPEPKDTTQ--VKSFLGMIN---YY----------HRHL

PNLA----

>SPM

------------------------------------LD----------------------

---------------RLGKAGVIKKV-----------KNS------RW------------

-----------------AAPIVTVPKS------D---G------------------S-VR

LCGDYK-QT------VN-------KVLEEDVYP-LPT-----------CEDL-FAN---L

A-GG------------KVF-SKIDLSNAYLQLEL---TDKSKELLTI-------------

----HTH-KG------------------LYQYERLPFGVSTAPAVFQSV-----MDR---

-------ILGG--M----------------------------KGVCCYLDD-ILISSE-N

E------ED---HIKTLNEVLGRLQEYGVKAK--------------KEKCSF--------

---------------MVSRVIYLGHEI---------------------------------

----DGKGVHPTKEKVEGICDAKRPENKDE--LRTFIGIVV---YY----------AKFV

PNLA----

>SURL

------------------------------------------------------------

----------------YERLDVITPV----------DEPT------DW------------

-----------------VSSLVVVMKK------N---G------------------Q-LR

VCLDPR-D-------LN-------RAIKREHYQ-LPS-----------RAEI-TAH---F

A-GA------------KYF-SKLDASSGFWQIQL---DDESSKLCTF-------------

----ITP-YG------------------RYKFLRLPFGICSAPEVYHKI-----VHQ---

-------MFAH--I----------------------------PGVNTMMDD-VIVWGT-T

Q------QE---HDNRLREVLSIARRMNLKLN--------------KDKCEF--------

---------------SVKKLTFIGDLI---------------------------------

----SDQGVQPDPKKVSAILNMERPKCRKD--VQRFLGMIN---YQ----------GKFI

PDLA----

>Cer4

------------------------------------------------------------

-----------------LDLEVLEPI-----------THS------DW------------

-----------------AAPIVVIRKK------D---TG-----------------K-VR

VCADFK-CS-----GLN-------NSLIEEIHP-LPT-----------SDDL-FGT---L

Q-G-------------CIF-SKIDLKDAYLQIAL---DSESQKLAVI-------------

----NTH-KG------------------LFKYRRMTFGLKPAPAKFQKI-----IDK---

-------MIAG--L----------------------------PGVAAYLDD-VIVSAN-S

L------EE---HEKVLHELLKRIKDYGFRIS--------------PEKCNF--------

---------------AQSEITFLGFII---------------------------------

----DKRGRRPDPKKTSVIRSMKAPTDQKQ--LMSFLGAIC---FY----------GRFV

PKMS----

>Cer5

------------------------------------------------------------

-----------------QEMGVIVPI-----------TYA------KW------------

-----------------AAPIVVIKKK------G---TG-----------------K-IR

VCADFK-CS-----GLN-------AALKDEFHP-LPT-----------SEDI-FSR---L

K-G-------------TVY-SQIDLKDAYLQVEL---DEEAQKLAVI-------------

----NTH-RG------------------IFKYLRMTFGLKPAPASFQKI-----MDK---

-------MVSG--L----------------------------TGVAVYLDD-IIISAS-S

I------EE---HEKILRELFERIKEYGFRVS--------------AEKCAF--------

---------------AQKQVTFLGFIV---------------------------------

----DEHGRRPDPKKTEVIRSMKAPTDQKQ--LASFLGAVS---FY----------SRFV

PKMS----

>Cer6

------------------------------------------------------------

-----------------KDMGVITPI-----------TYS------KW------------

-----------------AAPIVVIRKK------E---SN-----------------K-IR

VCADFK-CS-----GLN-------SALKDEIHP-LPT-----------CEDL-FAK---L

K-G-------------SVF-SKIDFRDAYLQIEL---DDESQELAVI-------------

----NTH-LG------------------LFKYVRMAFGLKPAPAVFQKI-----VDK---

-------LISG--I----------------------------PGVSAYLDD-LIIAAS-S

Y------DE---HEAILRQLFDRIREYGFRVS--------------PEKCMF--------

---------------AVSEISFLGFIV---------------------------------

----DEKGRRPDPNKASKIRSMKAPGDQKQ--LSSFLGAVC---FY----------SRFV

PKMS----

>412

------------------------------------------------------------

-----------------IKDKIVEP------------SVS------QY------------

-----------------NSPLLLVPKK-SSPNSD-K-K------------------K-WR

LVIDYR-Q-------INK------KLL-ADKFP-LPR-----------IDDI-LDQ---L

G-RA------------KYF-SCLDLMSGFHQIEL---DEGSRDITSF-------------

----STS-NG------------------SYRFTRLPFGLKIAPNSFQRM-----MTI---

-------AFSG--I--------------------------EPSQAFLYMDD-LIVIGC-S

E------KH---MLKNLTEVFGKCREYNLKLH--------------PEKCSF--------

---------------FMHEVTFLGHKC---------------------------------

----TDKGILPDDKKYDVIQNYPVPHDADS--ARRFVAFCN---YY----------RRFI

KNFA----

>Mdg1

------------------------------------------------------------

-----------------IKDGIVEQ------------SIS------EY------------

-----------------NSPLLLVPKK-SLPNSE-E-K------------------R-WR

LVVDYR-Q-------INK------KLL-ADKFP-LPR-----------IEDI-LDQ---L

G-RA------------KYF-SCLDLMSGFHQIEL---DERSRNITSF-------------

----STS-TG------------------AYRYTRLPFGLKIAPNSFQRM-----MTL---

-------AFSG--L--------------------------TPSQAFLYMDD-LVVIGC-S

E------KH---MLKNLTDVFKLCRQHNLKLH--------------PEKCTF--------

---------------FMKEVTYLGHKC---------------------------------

----TDKGILPDDSKYEVIKNYPKPVNADE--ARRFVAFCN---YY----------RRFI

KNFA----

>HMSBeagle

------------------------------------------------------------

-----------------LHDGIIRP------------SRS------PY------------

-----------------NSPVWIVPKK-LDSSGK---K------------------K-YR

VVIDYR-K-------LNM------VTV-ADRYP-IPD-----------INEV-LAQ---L

G-DN------------KIF-SVLDLKSGFHQILL---KESDIEKTAF-------------

----SIN-NG------------------KYEFTRLPFGLKNAPSIFQRA-----LDD---

-------ILHE--H--------------------------IGKICFIYIDD-IIIFSK-D

D------ET---HYQNLDTIFRTLQQANMKCQ--------------LDKCEF--------

---------------MKRKVEFLGFVV---------------------------------

----SDKGIETSPTKVQAISDFPIPRTLKE--LRSFLGLSG---YY----------RRFI

PNYA----

>Yoyo

------------------------------------------------------------

-----------------LEDGIIRP------------SRS------PY------------

-----------------NSPVWIVDKK-PDSLGN---K------------------Q-YR

LVIDYR-K-------LNS------VTI-ADRYP-IPE-----------INEV-LSH---L

G-SN------------TFF-SVIDLKSGFHQIPL---KNSDIEKTAF-------------

----SIN-NE------------------KYEFTRLPFGLKNAPSIFQRT-----LDD---

-------ILRD--Y--------------------------IGQCCYVYIDD-IIIFSR-N

E------KE---HSTHLKNIFTTLEKANMKVQ--------------LDKCKF--------

---------------FEKEVEFLGFIV---------------------------------

----TPEGIKTNPSKIEAIQNFPIPRNLKE--LRSFLGLSG---YY----------RRFV

KDYA----

>Gypsyvir

------------------------------------------------------------

-----------------LDDGIIRP------------SRS------PY------------

-----------------NSPTWVVDKKGTDSYGN---P------------------K-KR

LVIDFR-K-------LNE------KTI-PDRYP-MPR-----------IPMI-LAN---L

G-KA------------KYF-TTLDLKSGYHQIYL---AEHDREKTSF-------------

----SVS-SG------------------KYEFCRLPFGLRNASSIFQRA-----IDD---

-------ILRE--H--------------------------IGKICFVYVDD-VIIFSK-N

E------TE---HLQHINIVLKCLIDANMRVG--------------PEKTRF--------

---------------FKESIEFLGFIV---------------------------------

----TKDGATSDPKKVKAIQEFPEPKNVYS--VRSFLGLAN---YY----------RVFI

KDGA----

>Burdock

------------------------------------------------------------

-----------------LKDGIIRP------------SSS------PY------------

-----------------NNPVWVVDKKGTDEEGN---T------------------K-KR

LVIDFR-K-------LNL------KTI-DDKYP-IPN-----------VVWI-LSN---L

G-KA------------RFF-TTLDLKSAFHQILL---AEKDRAKTAF-------------

----SVG-NG------------------KYEFCRLPFGLKNAPSIFQRA-----IDD---

-------VVRD--R--------------------------IGKSCYVYVDD-VIIFSN-G

I------ED---HVNDVAWVLDRLSGANMRVS--------------KEKSFF--------

---------------FKESVEYLGFMV---------------------------------

----SSGGITTSPSKVEAIQKYNQPTNLFS--VRSFLGLAS---YY----------RCFI

KDFA----

>Nomad

------------------------------------------------------------

-----------------LQDGIIRP------------SNS------PY------------

-----------------NSPIWIVPKK-PKPNGE---K------------------Q-YR

MVVDFK-R-------LNT------VTI-PDTYP-IPD-----------INAT-LAS---L

G-NA------------KYF-TTLDLTSGFHQIHM---KESDIPKTAF-------------

----STL-NG------------------KYEFLRLPFGLKNAPAIFQRM-----IDD---

-------ILRE--H--------------------------IGKVCYVYIDD-IIVFSE-D

Y------DT---HWKNLRLVLASLSKANLQVN--------------LEKSHF--------

---------------LDTQVEFLGYIV---------------------------------

----TADGIKADPKKVRAISEMPPPTSVKE--LKRFLGMTS---YY----------RKFI

QDYA----

>Gypsy

------------------------------------------------------------

-----------------LKDGIIRP------------SRS------PY------------

-----------------NSPTWVVDKKGTDAFGN---P------------------N-KR

LVIDFR-K-------LNE------KTI-PDRYP-MPS-----------IPMI-LAN---L

G-KA------------KFF-TTLDLKSGYHQIYL---AEHDREKTSF-------------

----SVN-GG------------------KYEFCRLPFGLRNASSIFQRA-----LDD---

-------VLRE--Q--------------------------IGKICYVYVDD-VIIFSE-N

E------SD---HVRHIDTVLKCLIDANMRVS--------------QEKTRF--------

---------------FKESVEYLGFIV---------------------------------

----SKDGTKSDPEKVKAIQEYPEPDCVYK--VRSFLGLAS---YY----------RVFI

KDFA----

>297

------------------------------------------------------------

-----------------LNQGLIRE------------SNS------PY------------

-----------------NSPTWVVPKK-PDASGA---N------------------K-YR

VVIDYR-K-------LNE------ITI-PDRYP-IPN-----------MDEI-LGK---L

G-KC------------QYF-TTIDLAKGFHQIEM---DEESISKTAF-------------

----STK-SG------------------HYEYLRMPFGLRNAPATFQRC-----MNN---

-------ILRP--L--------------------------LNKHCLVYLDD-IIIFST-S

L------TE---HLNSIQLVFTKLADANLKLQ--------------LDKCEF--------

---------------LKKEANFLGHIV---------------------------------

----TPDGIKPNPIKVKAIVSYPIPTKDKE--IRAFLGLTG---YY----------RKFI

PNYA----

>17.6

------------------------------------------------------------

-----------------LNQGIIRT------------SNS------PY------------

-----------------NSPIWVVPKK-QDASGK---Q------------------K-FR

IVIDYR-K-------LNE------ITV-GDRHP-IPN-----------MDEI-LGK---L

G-RC------------NYF-TTIDLAKGFHQIEM---DPESVSKTAF-------------

----STK-HG------------------HYEYLRMPFGLKNAPATFQRC-----MND---

-------ILRP--L--------------------------LNKHCLVYLDD-IIVFST-S

L------DE---HLQSLGLVFEKLAKANLKLQ--------------LDKCEF--------

---------------LKQETTFLGHVL---------------------------------

----TPDGIKPNPEKIEAIQKYPIPTKPKE--IKAFLGLTG---YY----------RKFI

PNFA----

>Tv1

--------------------------------PFSDLI----------------------

---------------KPIPNGL--------------------------------------

-------------------PVIIVPKR-NDAFGK---P------------------K-FR

LVIDYR-H-------FNE------LTI-NDKYP-IPI-----------MDEI-LDK---L

G-KC------------QYF-TTIDLAKGFHQIQM---DPGSIPKTAF-------------

----STK-HG------------------HYEYTRMPFGLKNAPATFQRC-----MNN---

-------LLED--L--------------------------IFKDCLVHLDD-IIIFST-S

L------EE---HILSLQKVFKKLREANLKLQ--------------LDKCEF--------

---------------MRKETEFLGHII---------------------------------

----TTEGIKPNPNKIQAIVKFPIPKTPKE--IKSFLGLCG---FY----------RKFI

PNFA----

>Ted

------------------------------------------------------------

-----------------LDQGIIRP------------SDS------AW------------

-----------------SSPIWVVPKK-IDASGK---Q------------------K-WR

LVVDFR-K-------LNE------KTI-DDKYP-IPN-----------ISDV-LDK---L

G-KC------------QYF-TTLDLASGFYQVEM---DPQDISKTAF-------------

----NVE-HG------------------HFEFLRMPMGLKNSPSTFQRV-----MDN---

-------VLRG--L--------------------------QNNICLVYLDD-IIVYST-S

L------QE---HLENLERVFQRLRESNFKIQ--------------MDKSEF--------

---------------LKLETAYLGHII---------------------------------

----SRDGIKPNPDKISAIQKYLIPKTPKE--IKQFLGLLG---YY----------RKFI

PDFA----

>Zam

------------------------------------------------------------

-----------------LDQQIIRS------------SHS------PW------------

-----------------SAPVWVVPKK-LDGTGN---R------------------K-WR

LVIDYR-K-------LND------KTI-SDRYP-IPN-----------INDI-LDS---I

G-KA------------KYF-STLDLTSGFHQIEM---NPKDIAKTAF-------------

----TVE-GG------------------HYEFTRMPFGLKNAPATFQRV-----MDS---

-------VLGD--L--------------------------NGTICLFYLDD-IIIFSP-S

L------QK---HLLDIKMVFEKLRAANFKLQ--------------PSKSEF--------

---------------LRKEIEFLGHIV---------------------------------

----TQDGVKPNPNKISAIKKFPCPTNRRA--IKSFLGLLG---YY----------RKFI

RDFA----

>Idefix

------------------------------------------------------------

-----------------IEQGIVRK------------SKS------PY------------

-----------------CSPIWVVPKK-ADASGK---Q------------------K-FR

LVVDYR-N-------LNE------ITV-NDKFP-IPR-----------MDEI-LDK---L

G-RC------------QYF-TTIDLAKGFHQIQM---DENSIAKTAF-------------

----STK-HG------------------HYEYTRMPFGLKNAPATFQRC-----MNN---

-------LLED--L--------------------------IYKDCLVYLDD-IIVYST-P

L------EE---HILSLKKVFEKLRDANLKLQ--------------LDKCEF--------

---------------MKKETEFLGHIV---------------------------------

----TTNGIKPNPNKTKAITNFPLPKTPKQ--IKSFLGLCG---FY----------RKFI

PNFA----

>Tom

------------------------------------------------------------

-----------------LEQGLIRE------------SNS------PY------------

-----------------NSPTWVVPKK-PDASGK---A------------------K-YR

VVIDYR-K-------LNE------ITI-PDRFP-IPN-----------MDEI-LGK---L

G-KC------------QYF-TTIDLARGFHQIEM---DSESIQKTAF-------------

----STK-RG------------------HYEYVRMPFGLRNAPATFQRC-----MNN---

-------ILRP--L--------------------------INKHCLVYLDD-MIIFST-S

L------DE---HLNSLQLVFEKLSESNLKLQ--------------LDKCEF--------

---------------LKKEATFLGHIV---------------------------------

----TPDGIKPNPLKVEAIASYPIPTKVKE--IRAFLGMTG---YY----------RKFI

PSYA----

>Springer

------------------------------------------------------------

-----------------LKNGIIQK------------SKS------PY------------

-----------------NNPIWVVDKKGTDDAGN---K------------------K-MR

LVLDFR-K-------LNE------RTV-PDRYP-MPN-----------ISMI-LGN---L

G-KA------------KYF-TTLDLKSGYHQITL---AERDREKTAF-------------

----AVN-GG------------------KYEFRRLPFGLRNAASIFQRT-----IDD---

-------ILRE--Q--------------------------IGKFCYVYVDD-VIIFSE-D

E------ND---HVKHVDWVLKSLYDANMRIS--------------AEKSRF--------

---------------FKKSVSFLGFIV---------------------------------

----TNNGAATDPEKVKAIKEFPEPKNVFE--VRSFLGLAS---YY----------RCFI

KDFA----

>Blastopia

------------------------------------------------------------

-----------------VEQSIVRK------------STS------NV------------

-----------------ASRIVVVRKK------D---G------------------T-LR

VCVDYR-K-------LNT------MVL-MDCFP-VPI-----------MEEV-LEK---L

Q-SA------------KWF-TTMDLQNGFFHVAV---EEASKPYTAF-------------

----VTR-EG------------------LFEFNKAPFGFKNSPAAFIRF-----VQF---

-------IFQE--LI-------------------------NSNIMQLYMDD-IIVYAA-T

P------EE---CMEKTEMVLKRAAEFGLKIK--------------WKKCNF--------

---------------MQRRIHFLGHII---------------------------------

----EGGQICPGKEKTSAVNSFGTPQNVKA--VQGFLGLTG---FF----------RKFI

PGYA----

>Micropia

------------------------------------------------------------

-----------------IRCNIVRP------------SCS------PF------------

-----------------ASPMLLVKKK------N---G------------------T-DR

LCVDFR-E-------LNS------NTI-SDKYP-LPL-----------ISDQ-IAR---L

R-GA------------NYF-TCLDMASGFHQIPI---HPESVEYTAF-------------

----------------------------------VPDGLKNAPSVFQRT-----VIN---

-------ALGD--L--------------------------ANSFVIVYMDD-IMVVSP-T

K------EL---ALERLKTVLNVLTKAGFTFN--------------LAKCSF--------

---------------LKTTVQYLGYEV---------------------------------

----RAGEIRPNVRKIASLSSLPPPQTVSG--VRQFIGLAS---YF----------RKFV

SGFA----

>Mdg3

------------------------------------------------------------

-----------------LENGFIRP------------SDS------EY------------

-----------------ASPIVLVKKK------T---G------------------D-LR

MCVDFR-K-------LNK------MTM-KDNYP-LPL-----------IDDL-LDR---M

N-EK------------TVF-TKLDLKNGFFHVHV---KKESIKYTSF-------------

----VTP-LG------------------QYEWLRMPFGLKNAPSVFQRF-----VNK---

-------IFAD--MI-------------------------RENKVVVYMDD-ILLATE-N

I------NE---HLETLKEIFKRLVENKLELR--------------IDKCEF--------

---------------MQSSIKYLGFII---------------------------------

----NKDGIMPNDKGIEAIKNFPIPNNVHT--VQSFLGLCS---YF----------RRFI

KDFS----
